# Identification of high-performing antibodies for the reliable detection of Tau proteoforms by Western blotting and immunohistochemistry

**DOI:** 10.1007/s00401-024-02729-7

**Published:** 2024-05-18

**Authors:** Michael J. Ellis, Christiana Lekka, Katie L. Holden, Hanna Tulmin, Faheem Seedat, Darragh P. O’Brien, Shalinee Dhayal, Marie-Louise Zeissler, Jakob G. Knudsen, Benedikt M. Kessler, Noel G. Morgan, John A. Todd, Sarah J. Richardson, M. Irina Stefana

**Affiliations:** 1 JDRF/Wellcome Diabetes and Inflammation Laboratory, Nuffield Department of Medicine, Centre for Human Genetics, University of Oxford, Roosevelt Drive, Oxford, UK; 2 Islet Biology Group, Department of Clinical & Biomedical Sciences, Exeter Centre of Excellence in Diabetes (EXCEED), University of Exeter, RILD Building, Exeter, UK; 3 Nuffield Department of Women’s and Reproductive Health, Women’s Centre, University of Oxford, John Radcliffe Hospital, Level 3, Oxford, UK; 4 Target Discovery Institute, Centre for Medicines Discovery, Nuffield Department of Medicine, University of Oxford, Roosevelt Drive, Oxford, UK; 5 Oxford Centre for Diabetes, Endocrinology and Metabolism, Department of Medicine, University of Oxford, Radcliffe, UK; 6 Section for Cell Biology and Physiology, Department of Biology, University of Copenhagen, Copenhagen, Denmark

**Keywords:** Tau, Splice isoforms, Phosphorylation, Antibody validation, Western blot, Immunohistochemistry

## Abstract

Antibodies are essential research tools whose performance directly impacts research conclusions and reproducibility. Owing to its central role in Alzheimer’s disease and other dementias, hundreds of distinct antibody clones have been developed against the microtubule-associated protein Tau and its multiple proteoforms. Despite this breadth of offer, limited understanding of their performance and poor antibody selectivity have hindered research progress. Here, we validate a large panel of Tau antibodies by Western blot (79 reagents) and immunohistochemistry (35 reagents). We address the reagents’ ability to detect the target proteoform, selectivity, the impact of protein phosphorylation on antibody binding and performance in human brain samples. While most antibodies detected Tau at high levels, many failed to detect it at lower, endogenous levels. By WB, non-selective binding to other proteins affected over half of the antibodies tested, with several cross-reacting with the related MAP2 protein, whereas the “oligomeric Tau” T22 antibody reacted with monomeric Tau by WB, thus calling into question its specificity to Tau oligomers. Despite the presumption that “total” Tau antibodies are agnostic to post-translational modifications, we found that phosphorylation partially inhibits binding for many such antibodies, including the popular Tau-5 clone. We further combine high-sensitivity reagents, mass-spectrometry proteomics and cDNA sequencing to demonstrate that presumptive Tau “knockout” human cells continue to express residual protein arising through exon skipping, providing evidence of previously unappreciated gene plasticity. Finally, probing of human brain samples with a large panel of antibodies revealed the presence of C-term-truncated versions of all main Tau brain isoforms in both control and tauopathy donors. Ultimately, we identify a validated panel of Tau antibodies that can be employed in Western blotting and/or immunohistochemistry to reliably detect even low levels of Tau expression with high selectivity. This work represents an extensive resource that will enable the re-interpretation of published data, improve reproducibility in Tau research, and overall accelerate scientific progress.

**Supplementary Information:**

The online version contains supplementary material available at 10.1007/s00401-024-02729-7.

## Background

**Fig. 1:**
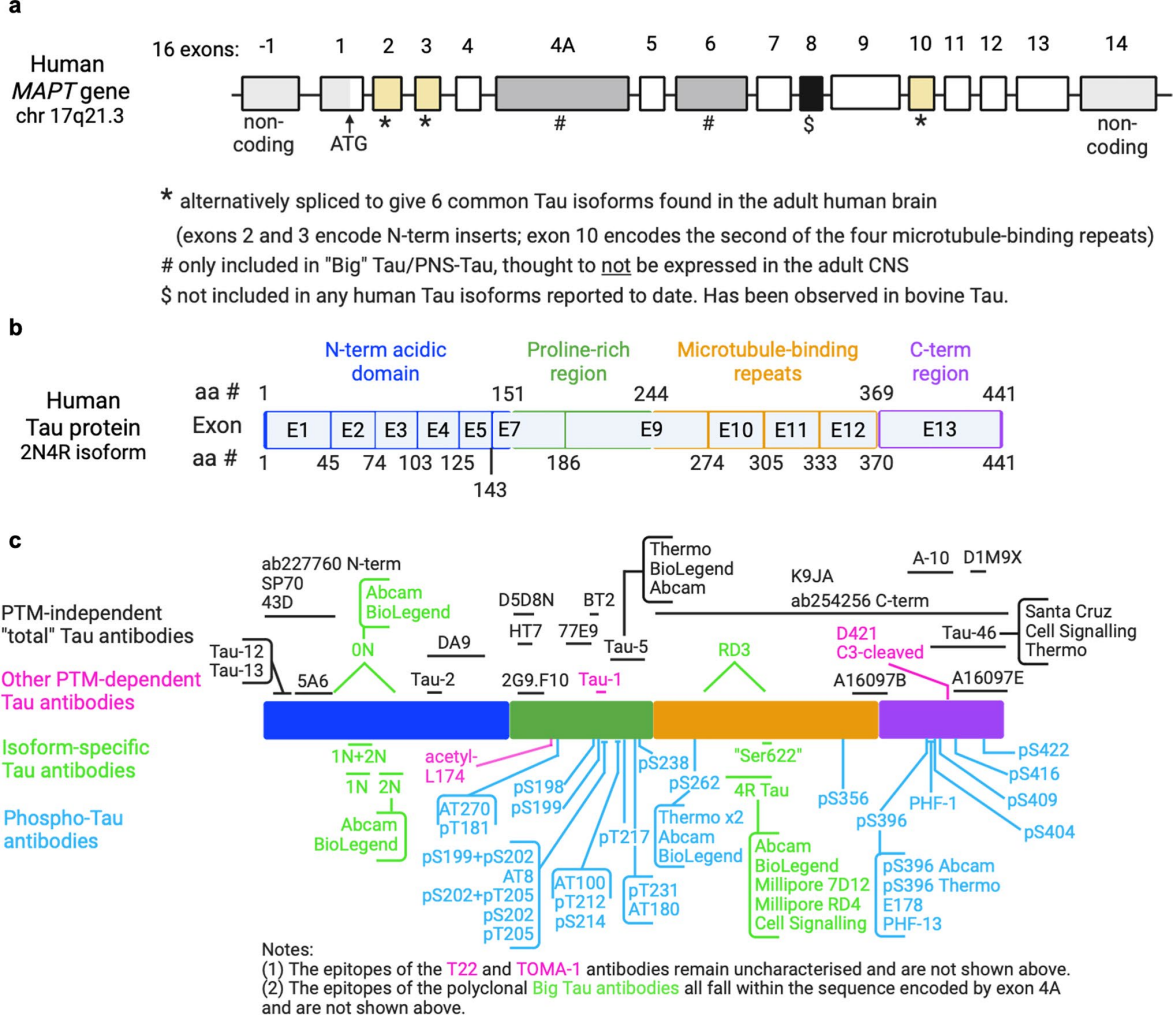
Overview of the human *MAPT* gene, Tau protein, antibody epitopes and antibody validation experimental strategies. **a** Diagram of the human *MAPT* gene structure with currently described exons depicted as rectangles and introns depicted as connecting lines. Exon numbering shown above. The canonical transcription start site (ATG) located in exon 1 is indicated (black arrow). Non-coding exonic regions are shown in light grey. Constitutively included exons (1, 4, 5, 7, 9, 11, 12 and 13) are shown in white. Alternatively spliced exons included in common brain Tau isoforms, Big Tau isoforms or not included in any human Tau isoforms described to date are shown in yellow (exon 2,3 and 10), light grey (exons 4A and 6) and black (exon 8), respectively. **b** Diagram of the human Tau protein (2N4R isoform, longest canonical Tau isoform), showing the four main protein domains: N-terminal acidic region (blue), proline-rich mid region (green), microtubule-binding repeat region (orange) and C-terminal region (purple). Amino acid residues marking the domain boundaries are shown above. Protein regions encoded by different exons are indicated. Amino acid residues marking exon–exon boundaries are shown below. **c** Schematic depiction of the epitopes targeted by the Tau antibodies included in this study: “total” Tau antibodies (above, black), phospho-Tau antibodies (below, blue), other PTM-dependent antibodies (magenta) and isoform-specific antibodies (green)

Discovered over four and a half decades ago [161], the microtubule-associated protein Tau has attracted ample research interest owing to its association with a wide range of neurodegenerative diseases, particularly tauopathies, a family of dementias marked by abnormal accumulation of protein aggregates containing hyperphosphorylated Tau [8, 168]. The Tau protein can be roughly divided into four main functional domains with different physicochemical properties: an acidic amino (N)-terminal domain, a proline-rich mid-domain, the microtubule-binding repeats (MTBR) domain, and a carboxy (C)-terminal domain (Fig. 1a). Although an intrinsically disordered protein, Tau can form local secondary structures and adopts distinct conformational folds that define different tauopathies [132]. Alternative splicing of the gene encoding Tau, *MAPT* (Fig. 1b), gives rise to six common Tau splice isoforms, distinguished by the presence of zero, one, or two N-terminal domains (0N, 1N and 2N Tau isoforms) and either three or four MTBRs (3R and 4R Tau isoforms) (Fig. 1a). The resulting unmodified protein isoforms have predicted molecular weights (MW) ranging from 36.7 to 45.9 kDa, but migrate on sodium dodecyl sulphate-polyacrylamide gel electrophoresis (SDS-PAGE) as a series of closely spaced bands with apparent MWs ranging from 58 to 66 kDa, thus showing abnormal retardation in electrophoretic mobility [64]. Truncation or, less commonly, skipping of constitutively included exons can generate Tau species with a MW below that of the shortest splice isoform [57, 68, 93, 109, 131], while inclusion of exons 4A and/or 6 gives rise to mid-MW and high-MW Tau isoforms, known as Big Tau or PNS-Tau [24, 54, 58, 65, 102]. Inclusion of exon 8 has been observed in bovine, but not human, Tau [33]. Adding further complexity to the study of Tau, each isoform can be subjected to a large number of post-translational modifications (PTMs), particularly phosphorylation with the 2N4R Tau isoform containing 85 residues that can accept a phosphate group, over 45 of which are reported to be phosphorylated in vivo or in vitro [3, 8].

Antibodies are among the most widely employed tools in the study of proteins, but few have been adequately characterised, and up to 60% may not perform as advertised [10]. Indeed, quality issues with research antibodies have been highlighted as one of the main contributors to the widely acknowledged “reproducibility crisis” in scientific research [5, 11, 25, 116, 162]. Despite an urgent need for validated reagents, and sustained efforts from both the scientific community and publishers [10, 12, 21, 22, 49, 70, 84, 116, 127, 138, 150, 151, 155, 156], no universally agreed standards exist for antibody validation, with most antibodies used in research studies not tested rigorously. Compounding this problem, antibody performance is both application-dependent and context-specific [2, 10, 96, 150]. Poorly characterised antibodies waste time, funds and biological samples (including precious patient material), and often lead to artefactual findings or the misinterpretation of research data.

Given its pathological relevance, an ever-increasing multiplicity of anti-Tau antibodies (referred to as “Tau antibodies” hereafter) have been developed (Table 1). Many of these have been widely employed in research studies, with the top 20 Tau antibody products on Citeab, representing 15 different antibody clones, amassing a total of 4,803 citations [100]. This overwhelming variety of antibody resources speaks to the widespread interest in the protein, but also highlights the difficulties of developing reliable, high-quality reagents. Given this, Tau antibody performance has been repeatedly questioned and addressed disparately at small scale (e.g., [123]; [88]), whereas the only two prior systematic attempts have tested fewer antibodies and only addressed selectivity in mouse brain samples [50, 115]. Hence, there is an urgent need for a clear understanding of Tau antibody performance to yield a validated set of reagents that can detect Tau in human samples, cover the entire length of the protein, can be used to discriminate between isoforms and target the most widely researched PTMs.

**Table 1:** Summary of existing Tau antibodies compiled from publicly available antibody databases

Database name	No. of Tau antibodies	Notes
Benchsci [9]	10,830	Search term: “MAPT [human]”
Antibodypedia [7]	6,454	53 suppliers
Alzforum [4]	572	Includes records of antibodies developed by individual research laboratories that are not currently available commercially
Labome [82]	460	29 suppliers 135 reagents have been cited in published studies 87 reagents included on the Labome “validated antibodies” list
Citeab [100]	3,502	Search term: “MAPT” + filtered for “Protein = Tau (Human)”

Here, we use a combination of approaches in both human and murine samples to characterise a panel of 79 Tau antibodies for use in Western blotting (WB), and a subset of these (35 antibodies) for use in immunohistochemistry (IHC) on formalin-fixed paraffin-embedded (FFPE) tissue sections. WB and IHC were chosen owing to their status as the two most common uses of antibodies overall [150] and the two most common applications for Tau antibodies [7, 9, 82]. To enable studies of clinical relevance, we focus on identifying antibodies that react with human Tau and include testing data for their performance in human brain samples. We highlight problematic reagents, reveal insights into widely used reagents that can aid reinterpretation of existing research data and, ultimately, identify high-quality antibodies that demonstrate selectivity towards the Tau protein. By identifying antibodies that can be used to reliably detect Tau proteoforms, including when the target proteins are expressed at physiological (often low) levels, these findings will improve reproducibility of future research studies, whilst concurrently broadening Tau research by facilitating a deeper understanding of the protein’s physiological roles in the CNS and beyond.

## Methods

Detailed information on experimental procedures is provided in Supplementary Materials and Methods.

### Antibodies

The complete list of antibodies used in this study is available in Supp. Table S1.

### Cell culture

Human embryonic kidney (HEK) 293 T cells (RRID: CVCL_0063), HEK293T Lenti-X cells (Takara Bio, cat. no. 632180; RRID: CVCL_4401), SH-SY5Y cells (European Collection of Authenticated Cell Cultures via Sigma-Merck, cat. no. 94030304; RRID: CVCL_0019), HAP1 cells (Horizon Discovery; parental/wildtype control cells cat. no. C631, RRID: CVCL_Y019; 2 bp deletion cells cat. no. HZGHC003277c009, RRID:CVCL_SX09; 14 bp deletion cells cat. no. HZGHC003277c003, RRID:CVCL_SX08) were maintained according to established cell culture protocols following the supplier’s instructions, where applicable.

### Tau overexpression

Plasmids used for tdTomato-Tau overexpression in HEK293T cells and the tdTomato-C1 control plasmid were a gift from Michael Davidson: tdTomato-MAPTau-C-10 (RRID: Addgene_58112), tdTomato-MAPTau-N-10 (RRID:Addgene_58113) and tdTomato-C1 (RRID:Addgene_54653). Control tdTomato-N1 plasmid was a gift from Michael Davidson, Nathan Shaner and Roger Tsien (RRID:Addgene_54642) [129]. For Tau overexpression in SH-SY5Y cells, the cDNA sequence of either the 441 amino acids-long human 2N4R Tau isoform or that of the 758 amino acids-long human Big Tau isoform, respectively, was cloned into the pSMPUW-IRES-Bsd vector backbone (Cell Biolabs, cat. no. VPK-219). The empty vector was used as a control. Lentiviral particles generated using these plasmids were used to create SH-SY5Y cell lines that stably overexpress Tau, as well as corresponding control cell lines.

### Recombinant proteins

Tau ladder (either Sigma cat. no. T7951, or Signal Chem cat. no. T08-07N-250), GSK3β-phosphorylated recombinant Tau (Signal Chem, cat. no. T08-50FN-20), DYRK1A-phosphorylated recombinant Tau (Signal Chem, cat. no. T08-50RN-20) and CAMK2A-phosphorylated recombinant Tau (Signal Chem, cat. no. T08-50CN-20) were used as controls for antibody validation by WB. Recombinant MAP2c protein (Abcam, #ab114686) was used to test for cross-reactivity of Tau antibodies with MAP2.

### Animals

Five month-old, female wildtype, *Mapt* knockout (*Mapt*^−/−^; [44]) and humanised Tau (hTau; [164]) mice, all maintained on a C57BL/6 J background, were used to collect brain tissue samples for WB and IHC. rTg4510 mice [120] were 9-month-old male mice and were acquired from Eli Lily through collaboration with Dr Jonathan Brown (Exeter University, UK). All housing and experimental procedures were carried out in compliance with the local ethical review panel of the Universities of Oxford and Exeter, respectively, under UK Home Office project licenses held in accordance with the Animals (Scientific Procedures) Act 1986. Animals were housed under a 12-h light/dark cycle with ad libitum access to food and water.

### Human brain tissue samples

Anonymised human post-mortem brain tissue (4 µm formalin-fixed paraffin-embedded (FFPE) brain sections for IHC, or 1 g frozen brain tissue for WB), was obtained from the Oxford Brain Bank. For all donors, human brain samples were collected from the dorsolateral prefrontal cortex (Brodmann areas 9/46), except for donor 14 (GGT donor, ID 18/061). For this latter donor, brain tissue originated from the M1 region (motor cortex). Donor details are provided in Table 2.

**Table 2:** Human brain tissue donor details

Donor #	UK BBN number	OBB number	Sex	Diagnosis	WB sample ID	Age	Cause of death	Post-mortem delay	Pathological diagnosis	Braak Stage
1	BBN_11050	12/085	F	control	ctrl 1	71	Necrotising pancreatitis	8 h	Normal aged brain	I
2	BBN004.32463	10/071	M	control	ctrl 2	68	Metastatic choriocarcinoma	48 h	Metastatic choriocarcinoma with liver metastases causing hepatic haemorrhage. No evidence of an underlying neurodegenerative disorder. Some hypoxic ischaemic damage in the CA 1 region of the hippocampus	not assessed
3	BBN004.30213	17/054	F	AD	AD 1	73	unknown	75 h	Alzheimer's disease [CERAD definite], Braak tangle stage VI, Thal phase 5	VI
4	BBN004.33683	18/071	M	AD	AD 2	80	unknown	66 h	End-stage Alzheimer’s disease with severe and widespread amyloid angiopathy. There was also some small-vessel cerebrovascular disease. Rare Lewy bodies were seen in limbic neurons but not elsewhere. There were several microscopic foci of chronic inflammatory cells in medulla and cranial nerve root, of unclear cause	VI
5	BBN004.29033	09/002	F	PSP	PSP 1	83	pneumonia	216 h	Progressive Supranuclear Palsy (PSP). Abnormal Tau pathology was present in brainstem, deep grey nuclei, and cerebral cortex	III
6	BBN_24654	14/137	M	PSP	PSP 2	80	unknown	72 h	Progressive Supranuclear Palsy (PSP). Argyrophilic grain disease (AGD)	not assessed
7	BBN_6318	95/1338	F	CBD	CBD 1	73	Pulmonary oedema	56 h	Atypical Pick's disease or corticobasal degeneration, with tangles, Pick bodies, ballooned neurones, astrocytic plaques and severe nigral degeneration	not assessed
8	BBN_11057	12/101	M	CBD	CBD 2	70	unknown	80 h	CBD—FTD (Corticobasal degeneration presenting as frontotemporal dementia clinically). Additional atypical TDP-43 pathology in a similar distribution to Tau	not assessed
9	BBN_22195	14/012	F	PiD	PiD 1	72	unknown	48 h	Microscopic changes of Pick's disease. Severe neuronal loss, gliosis, and neuropil sponginess in atrophic regions of cortex. AT8 immunostained abundant Pick bodies, Pick cells, and some astrocytes, in atrophic cortex and also in some subcortical locations including amygdala, hippocampus, entorhinal cortex, striatum, locus ceruleus, basis pontis. Cerebral white matter underlying the severely atrophic cortex shows pallor and loss of myelinated fibres	not assessed
10	BBN004.35410	19/021	M	PiD	PiD 2	62	unknown	96 h	Fronto-temporal Lobar Degeneration—non-Ad tauopathy (Pick's Disease). Braak stage 0	0
11	BBN004.34125	18/091	F	Control		38	unknown	41 h	No neurodegenerative abnormality detected. Normal age-related changes	0
12	BBN004.32631	18/025	M	AD		74	unknown	41 h	Alzheimer’s disease with severe amyloid angiopathy.Confirmed TARDBP mutation	VI
13	BBN004.28421	16/039	F	PiD		73	unknown	49 h	Fronto-temporal lobar degeneration with abnormal deposition of Tau protein, predominately as round inclusions with neuronal cytoplasm (Pick bodies)	V
14	BBN004.33641	18/061	M	GGT		82	Pneumonia	39 h	Globular glial tauopathy (GGT) type II, a type of GGT that shows predominantly motor cortex and corticospinal tract degeneration, with more restricted anatomical involvement compared to GGT types I and III	II

### Protein extraction and Western blotting

Proteins were extracted from cells and ground frozen brain tissue in lysis buffer (1 × radio immunoprecipitation assay buffer (RIPA buffer, Merck Millipore, cat. no. 20–188) containing 0.1% SDS, 1 × cOmplete™ EDTA-free Protease Inhibitor Cocktail (Roche, cat. no. 11873580001) and 1 × PhosSTOP™ phosphatase inhibitors (Roche, cat. no. 4906837001)). Lysates were spun down at 20,000 × g to pellet genomic DNA and cellular debris, and the supernatant was aliquoted into fresh, low-protein-binding tubes. Samples were stored at -80 °C until further use.

For WB, protein extracts were mixed 3:1 with 4 × Laemmli buffer (Biorad, cat. no. 1610747) containing β-mercaptoethanol and heated at 95 °C for 10 min. Proteins were separated by sodium dodecyl sulfate–polyacrylamide gel electrophoresis (SDS-PAGE) on 4–15% gradient Mini-PROTEAN TGX Stain Free Gels (Bio-Rad, cat. no. 4568084 for 10-well gels, cat. no. 4561086 for 15-well gels) and transferred to Immobilon-FL PVDF membranes (Merck Millipore, cat. no. IPFL00010). Blots were blocked in 5% Amersham™ ECL Prime Blocking Reagent (SLS, cat. no. RPN418) diluted in TBS (blocking buffer, no detergent). Primary antibodies were diluted in blocking buffer containing 0.1% Tween-20 and membranes were incubated with primary antibodies for either 2 h at RT or overnight at 4 °C. For primary antibody details, see Supp. Table S1. Secondary antibodies (for details, see in Supp. Table S1) were diluted 1:10,000 in blocking buffer containing 0.1% Tween-20 and 0.01% SDS. Membranes were imaged dry on an Odyssey CLx scanner (LI-COR Biosciences), and blot images were visualised and quantified in the Image Studio software (version 5.2.5; LI-COR Biosciences).

For detailed experimental procedures regarding protein extraction from the different sample types and further information on Western blotting, see Supplementary Materials and Methods.

### Immunofluorescence labelling of FFPE tissue sections

For IHC labelling of FFPE tissue sections, antigens were unmasked by heat-induced epitope retrieval (HIER) using either 10 mM citrate solution (pH 6.0) and/ or Tris ethylenediaminetetraacetic acid (TE) solution (pH 9.0). Tissue sections were blocked with 5% normal goat serum in phosphate-buffered saline (PBS; blocking buffer) before incubating with primary antibodies either overnight at 4 °C or for 2 h at RT (for primary antibody details, see Supp. Table S1). The resulting antigen–antibody complexes were detected using Alexa Fluor-conjugated secondary antibodies (1/400; AlexaFluor 488, 555, 647—secondary antibodies are listed in Supp. Table S1) and cell nuclei were labelled with DAPI (Invitrogen, UK). If applicable, tissue sections were then counterstained with 0.5% Thioflavin S (ThS; diluted in water; Sigma Aldrich, cat. no. T1892). Sections were mounted in fluorescence mounting media (Agilent, UK, cat. no. S302380-2) before imaging. All slides were imaged on the Akoya PhenoImager HT™ Automated Quantitative Pathology Imaging System (CLS143455) and images were processed using the InForm™ image analysis platform (Akoya Biosciences, US). Quantification analysis of the immunofluorescent signal was performed using HALO® (Indica Labs), a gold standard image analysis platform for quantitative analysis of IHC data. See Supplementary Materials and Methods for further details.

### Lambda phosphatase treatment

To dephosphorylate samples, protein extracts, WB membranes and tissue sections, as appropriate, were treated with lambda phosphatase (NEB, cat. no. 0753) according to manufacturer’s instructions. Further details are provided in Supplementary Materials and Methods.

## Results

We tested a panel of 79 Tau antibodies (Fig. 1c; antibody details provided in Supp. Table S1), broadly divided into four classes: (i) “total” Tau antibodies, (ii) phospho-Tau antibodies, (iii) other PTM-dependent Tau antibodies, and (iv) isoform-specific Tau antibodies. The latter category includes antibodies against the little-studied Big Tau isoform, whose functions remain unknown. The term “total” Tau antibodies (sometimes also referred to as “pan” Tau antibodies) denotes reagents with the presumed ability to detect all Tau splice isoforms, regardless of phosphorylation status, aggregation status, or the presence of most other PTMs. However, by definition, these antibodies cannot detect Tau variants that lack the region containing the antibody epitope, so the “total” Tau denomination does not account for truncation. Unless otherwise specified, residue numbering is based on the human 2N4R brain Tau splice isoform (441 amino acids-long; Uniprot ID: P10636-8).

**Fig. 2:**
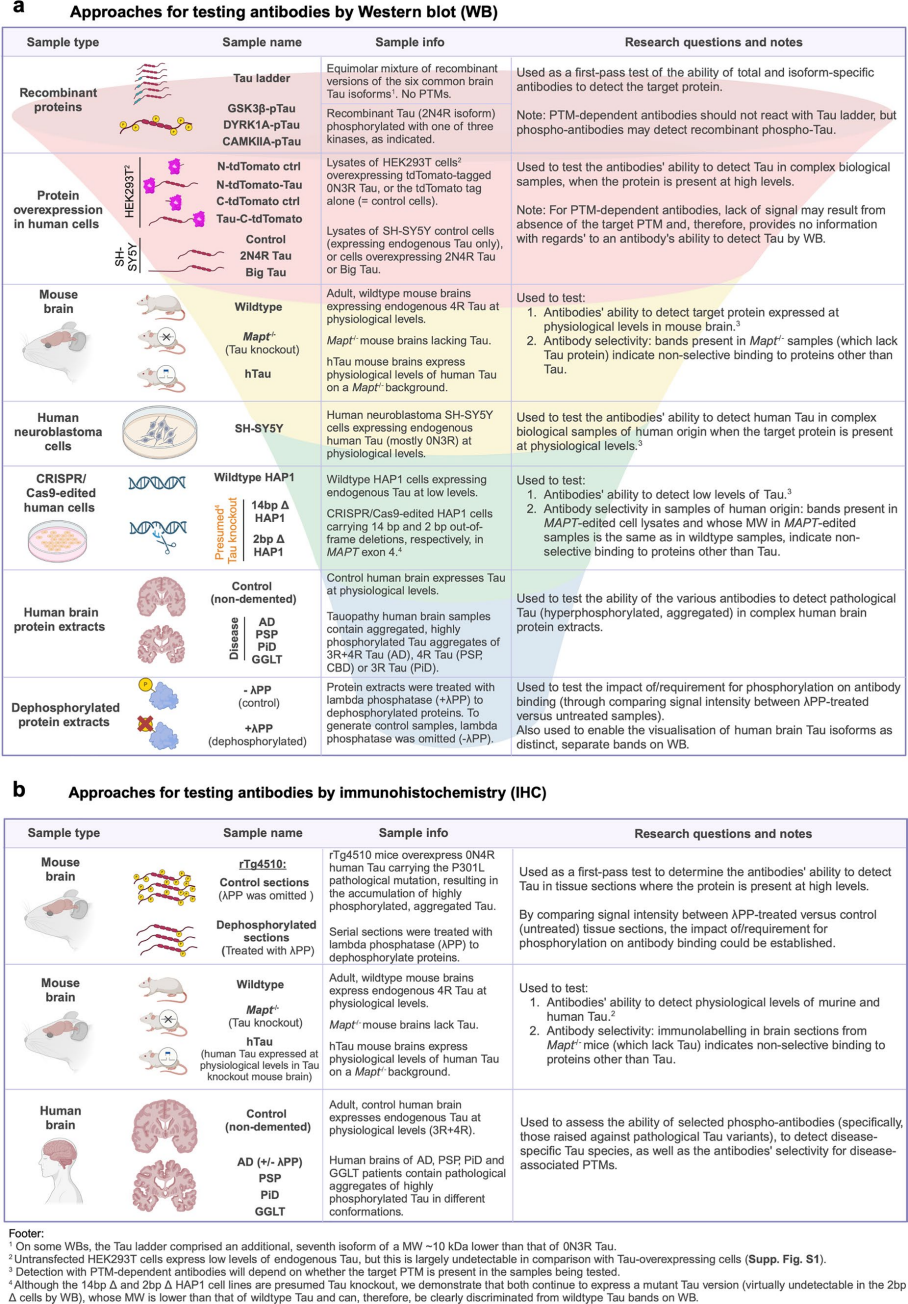
Overview of the experimental approaches employed in this study to validate Tau antibodies using WB (**a**) and IHC-IF (**b**)

**Fig. 3:**
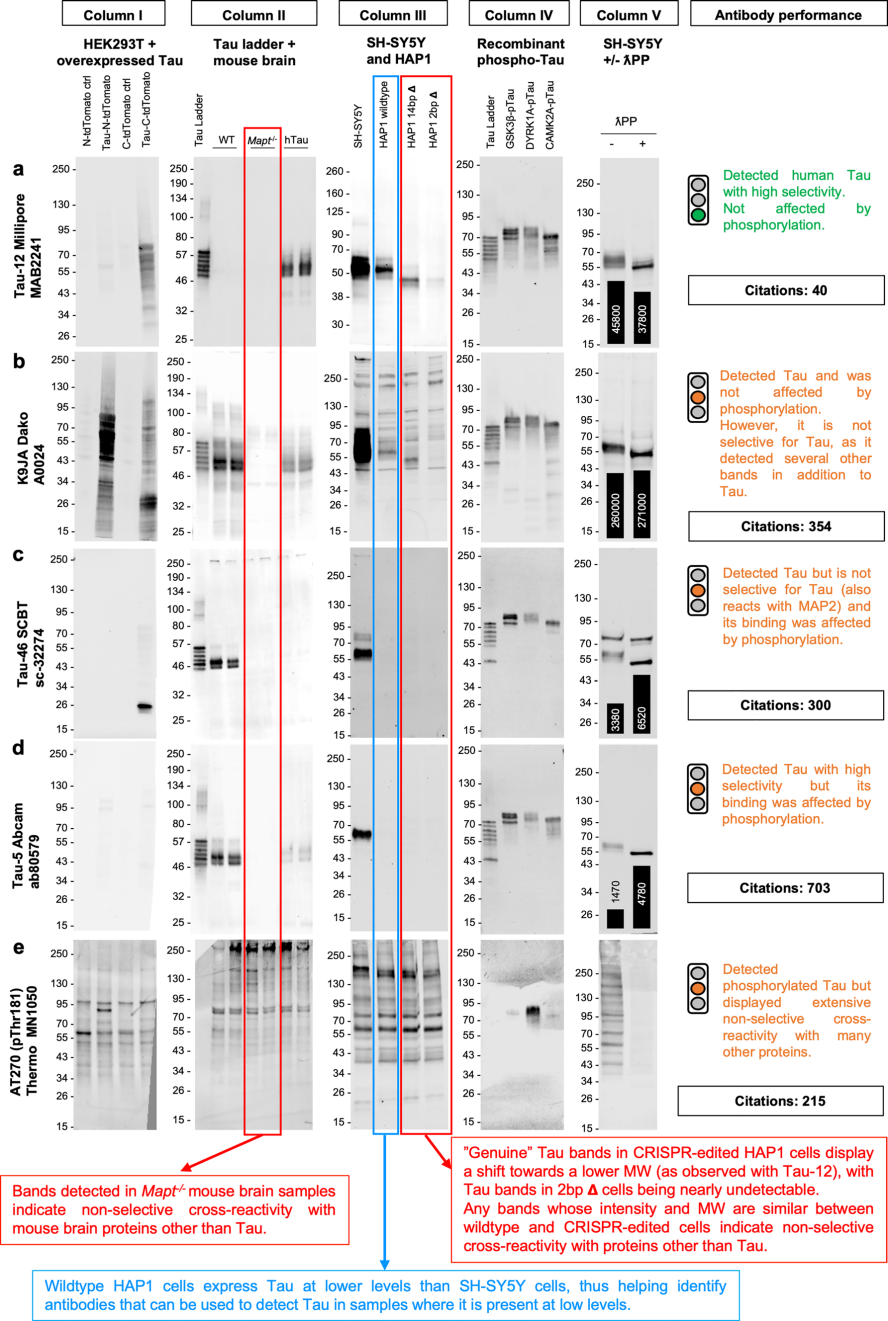
Representative antibody validation results for WB. a–e WB data illustrating the performance of different Tau antibodies: Tau-12 Millipore #MAB2241 (**a**), K9JA Dako #A0024 (**b**), Tau-46 SCBT #sc-32274 (**c**), Tau-5 Abcam #ab80579 (**d**), AT270 (pThr181) ThermoFisher Scientific #MN1050 (**e**). For each antibody, overall performance was categorised based on a traffic light system, as defined in Figs. 5–8. Number of citations for each antibody clone as of 9 Oct 2023 is indicated. WBs shown in columns I to V are as follows: WB of lysates from HEK293T cells overexpressing 0N3R human Tau and corresponding control cells (**column I**); WB of recombinant human Tau ladder (5 ng/isoform/lane), plus adult mouse brain lysates from wildtype, *Mapt*^*−/−*^ and hTau mice (**column II**); WB of lysates from SH-SY5Y neuroblastoma cells, plus HAP1 cells: parental (wildtype) and two cell lines carrying either a 14 bp deletion (14 bp Δ) or a 2-bp deletion (2 bp Δ) in *MAPT* exon 4 (**column III**); WB of recombinant human Tau ladder (50 ng/isoform/lane) plus recombinant 2N4R Tau that has been phosphorylated by one of three known Tau kinases: GSK3ꞵ, DYRK1A or CAMKIIA (**column IV**); WB of lysates from SH-SY5Y neuroblastoma cells that have been either untreated (-) or treated ( +) with λPP. Where applicable, quantifications of the Tau signal intensity for each lane are shown superimposed as a bar chart, with the respective values [a.u.] printed on or above each bar of the chart (**column V**)

**Fig. 4:**
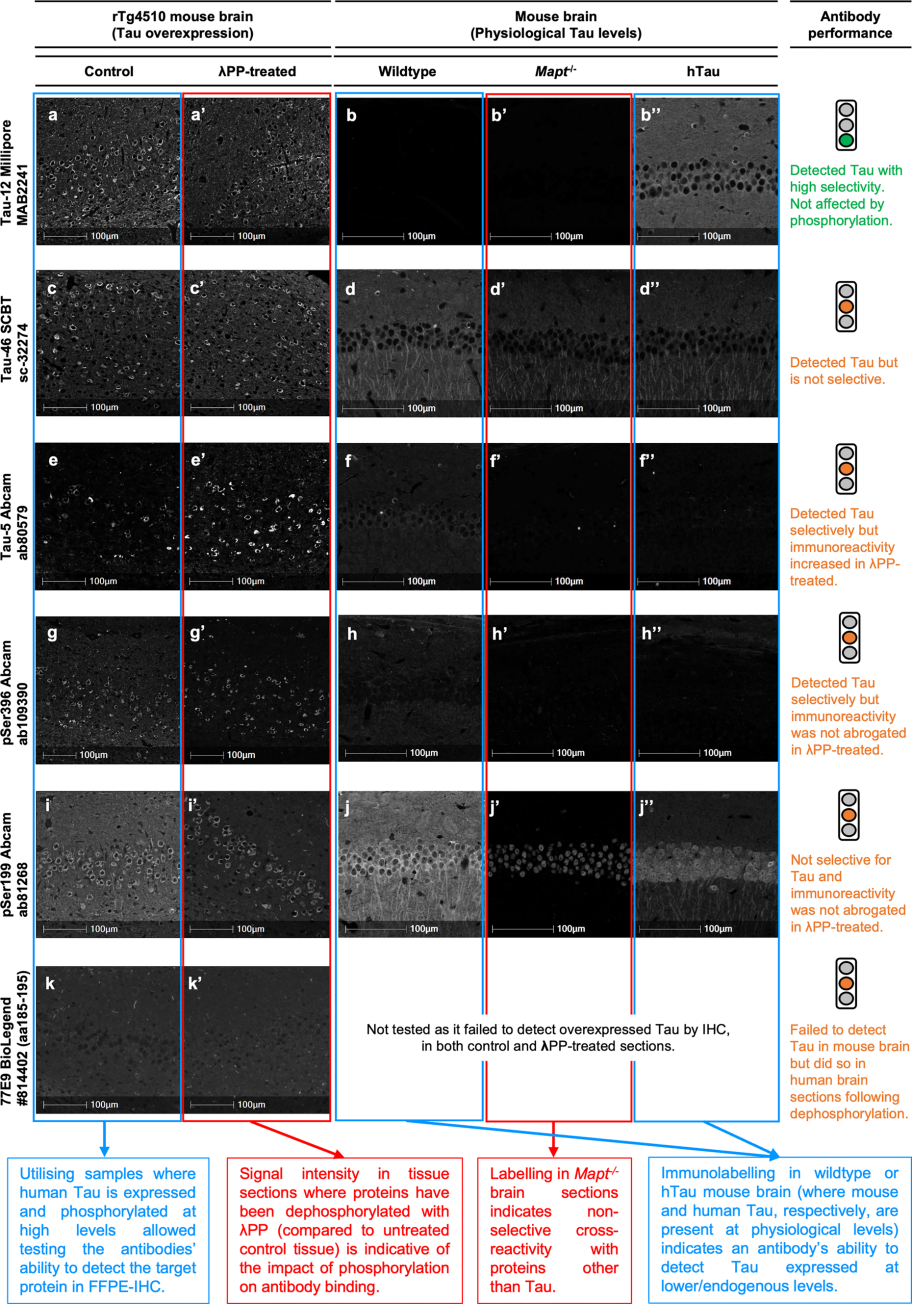
Representative antibody validation results for IHC-IF. **a–g’**: IHC-IF data illustrating the performance of different Tau antibodies: Tau-12 Millipore #MAB2241 (**a**–**b’’**), Tau-46 SCBT #sc-32274 (**c**–**d’’**), Tau-5 Abcam #ab80579 (**e**–**f’’**), pSer396 Abcam #ab109390 (**g**–**h'’**), pSer199 Abcam #ab81268 (**i**–**j'’**)**,** 77E9 BioLegend #814,402 (**k**–**k’**). For each antibody, overall performance was categorised based on a traffic light system, as defined in the text and in Figs. 5–8. **a-a’, c–c’, e-e’, g-g’, i-i', k-k':** Fluorescence micrographs of serial FFPE brain sections from 9-month old rTg4510 mice, either untreated (control; **a, c, e, g, i and k**) or treated (λPP-treated; **a’, c’, e’, g’, i' and k’**) with λPP, immunolabelled with Tau antibodies. **b-b”, d-d’’, f-f”, h–h'’, j-j'’:** Fluorescence micrographs of FFPE brain sections from 5-month-old wildtype (**b, d, f, h and j)**, *Mapt*^*−/−*^ (**b’, d’, f’ and j’**) and hTau (**b”, d”, f” and j’’**) mice immunolabelled with Tau antibodies. Brain regions imaged are cortex (**a-a’, c–c’, e-e’, g-g’, i-i' and k-k'**) and CA1 region of the hippocampus (**b-b”, d-d”, f-f”, h–h'’ and j-j'’**). Tau labelling is shown in grayscale. Scale bars = 100 µm

To assess antibody performance, we employed a variety of experimental strategies designed to establish each reagent’s ability to detect Tau, and their selectivity for Tau, in samples of murine and human origin, as well as the immunoreactivity of each reagent to phosphorylated and unphosphorylated Tau (Fig. 2a, b). where relevant, we first confirmed expression of transgenic proteins in the different cell lines (Supp. Fig. S1a), equal protein loading on WB (Supp. Fig. S1b, g, j, m, p), specificity of the secondary antibodies (Supp. Fig. S1c, d, h, i, k, l, n, o and Supp. Fig. S2), and low background autofluorescence (Supp. Fig. S1e, f and Supp. Fig. S2). Importantly, the antibodies’ ability to detect Tau was investigated not only when the target protein was present at high levels (e.g., purified recombinant proteins or protein overexpression), but also in samples expressing Tau at physiological levels, such as in brain or neuronal cell types (e.g., wildtype and hTau mouse brains, SH-SY5Y neuroblastoma cells), as well as in experimental samples where endogenous Tau is present at low levels (e.g. HAP1 cells) (Figs. 2a, b, 3, 4). We additionally tested immunoreactivity in human brain samples, to assess the antibodies’ ability to detect pathological Tau and overall performance in samples of clinical relevance (Fig. 2a, b). Antibody selectivity was established through comparing each reagent’s immunoreactivity in wildtype samples to that observed in presumed Tau “knockout” samples (Fig. 2a, b). As the latter are presumed to lack the Tau protein, signals detected in these samples can indicate that the respective antibody displays non-selective binding to proteins other than Tau. While our experimental data confirmed the Tau “knockout” status of *Mapt*^*−/−*^ mice, preliminary experiments revealed that Tau-immunoreactive bands were still detected by WB in *MAPT*-edited HAP1 cells. These bands were shifted to a lower MW compared to those observed in parental cells, suggesting that the presumed “knockout” cells continue to produce a lower MW version of Tau (Supp. Fig. S1q). Mass-spectrometry proteomic analyses of Tau immunoprecipitated from wildtype and 2 bp ∆ HAP1 cells identified Tau peptides in both cell lines, confirming that presumed “knockout” *MAPT*-edited HAP1 cells continue to express residual Tau protein (Supp. Fig. S3a). PCR-amplification of *MAPT* cDNA from wildtype and the two *MAPT*-edited HAP1 cell lines revealed that the edited cells produced a viable MAPT transcript through skipping of exon 4, the exon carrying the CRISPR/Cas9-introduced deletions in both cases (Supp. Fig. S3b), thus explaining the reduction in the MW of the detected protein. Nevertheless, HAP1 cells proved informative for antibody validation by WB due to the shift in MW displayed by “genuine” Tau bands following *MAPT* editing and the fact that residual Tau expression in the 2 bp ∆ cell line was very low, often undetectable, such that this cell line was as valuable as a true knockout would have been in practical terms. Taken together, these features allowed the clear distinction between selective and non-selective Tau-immunoreactive bands by WB, where selective (i.e. “genuine”) Tau bands: (a) displayed a shift to a lower MW in the 14 bp ∆ HAP1 cell line compared to the wildtype/parental cell line; (b) were fainter than the Tau bands detected in wildtype cells; and (c) were mostly undetectable in the 2 bp ∆ HAP1 cell line (Figs. 2a and 3, column III; Supp. Fig. S3b). In contrast, non-selective bands could be identified as bands whose signal intensity and MWs remained unaltered between wildtype compared to *MAPT*-edited HAP1 cell lines (Fig. 2a; Fig. 3, column III; Supp. Fig. S3b).

**Fig. 5:**
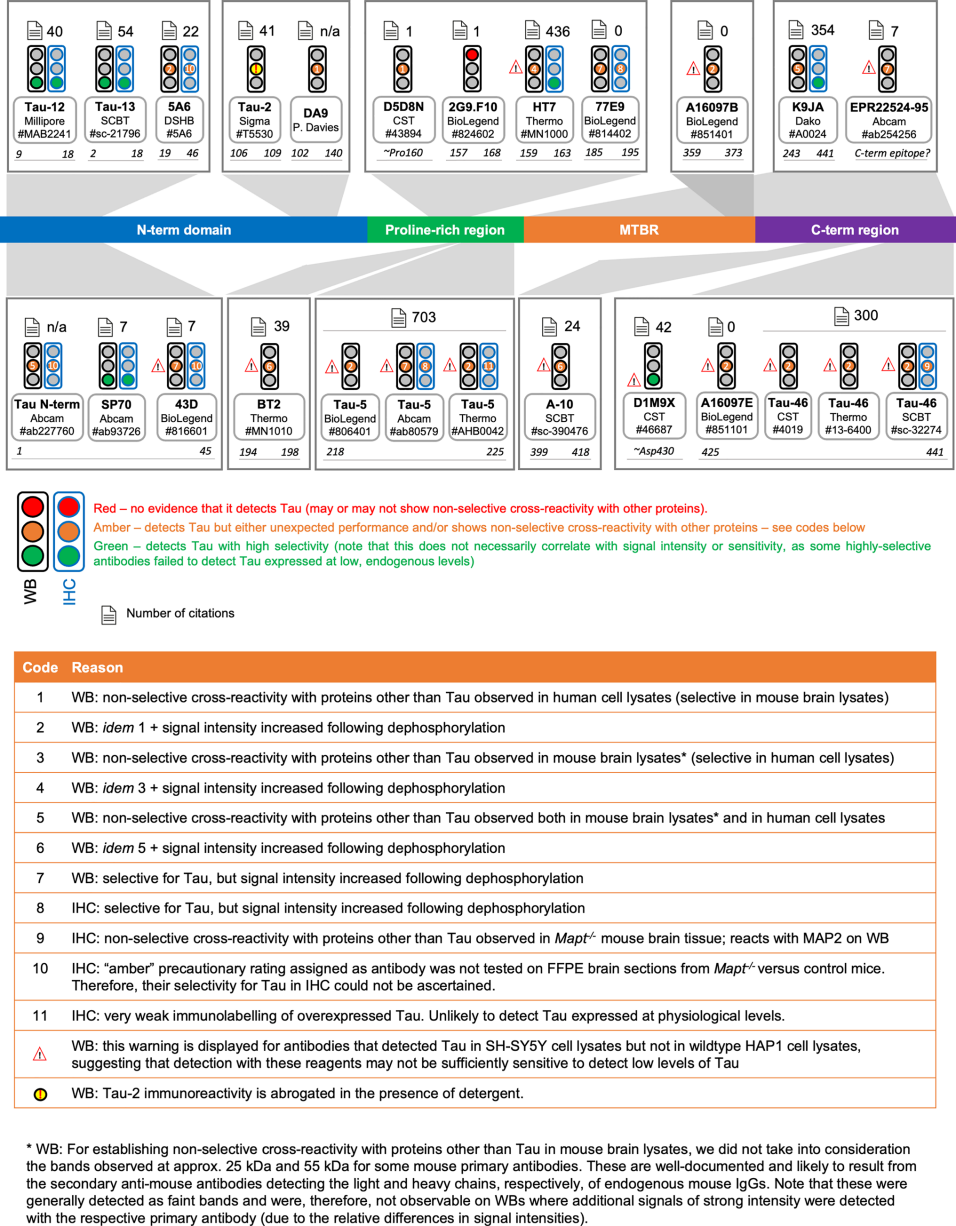
Summary of results for “total” Tau antibodies. Diagram of the Tau protein showing the locations of the epitopes of “total” Tau antibodies. In this and the following two figures: (i) residue numbering is based on 2N4R Tau (441 amino acids; Uniprot ID 10636–8); (ii) traffic lights summarise the performance of each antibody in WB (**black outline**) and IHC (**blue outline**); (iii) antibody performance was classified as either: **Green** = detects Tau with high selectivity; **Amber** = detects Tau but either unexpected performance was observed and/or antibody displayed non-selective cross-reactivity with other proteins (see codes); or **Red** = no evidence that the antibody detects Tau (may or may not show non-selective cross-reactivity with other proteins); (iv) the total number of studies citing the use of each antibody clone (aggregated for all formulations and vendors) is displayed

**Fig. 6:**
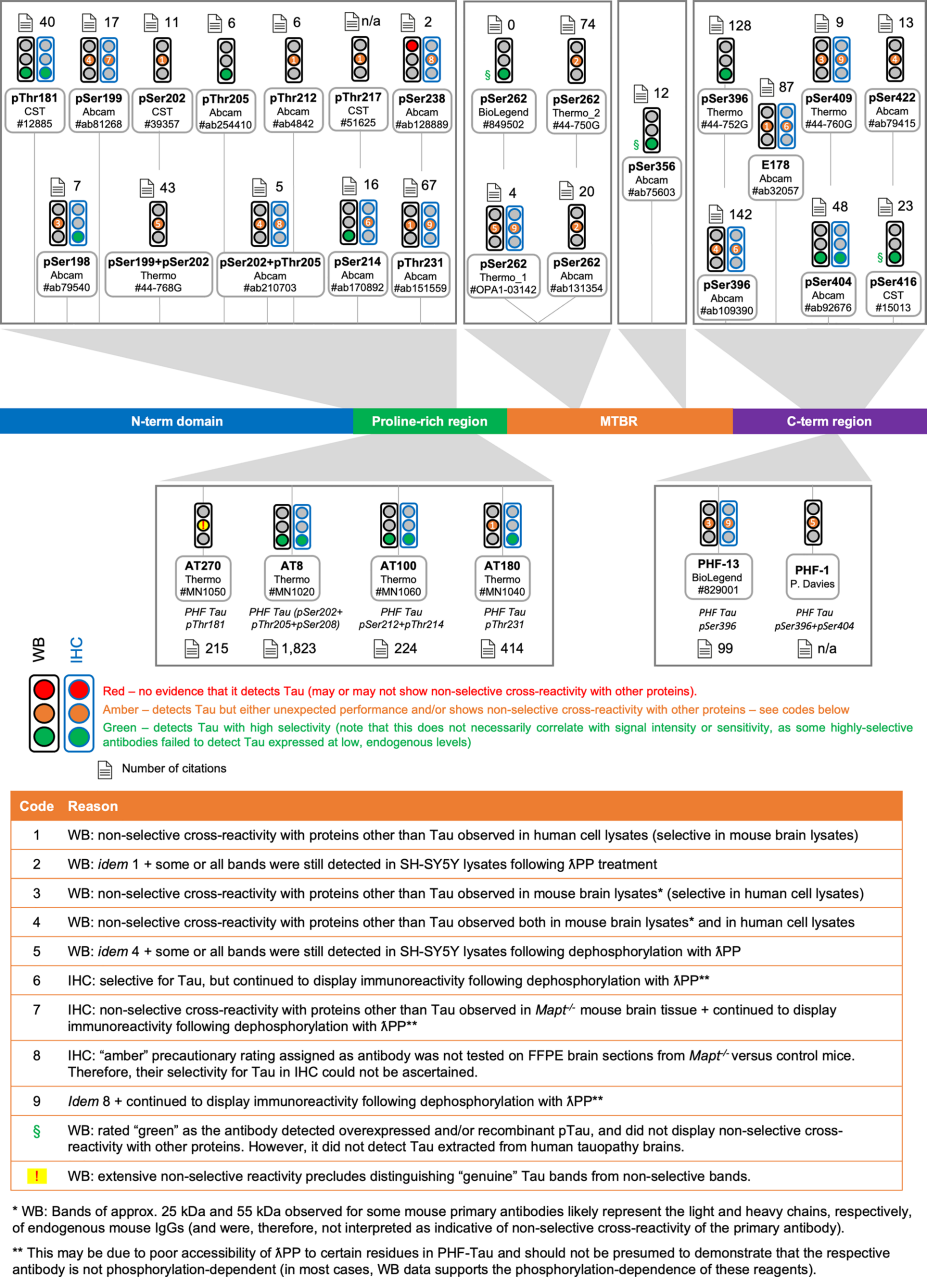
Summary of results for phosphorylation-dependent, other PTM-dependent and isoform-specific Tau antibodies. Diagram of the Tau protein showing the locations of the epitopes of phospho-Tau antibodies

**Fig. 7:**
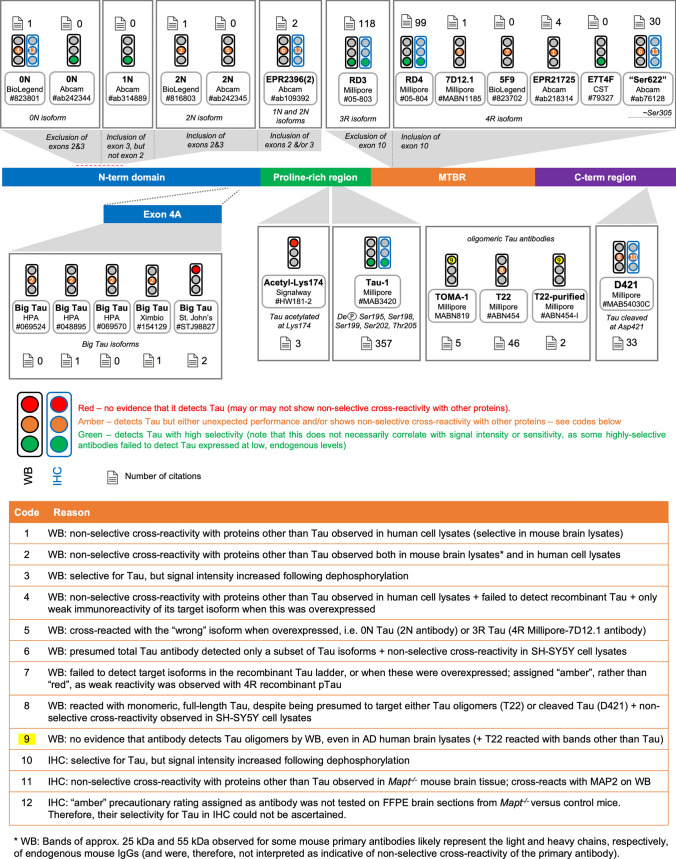
Summary of results for other PTM-dependent and isoform-specific Tau antibodies. Diagram of the Tau protein showing the locations of the epitopes of other-PTM dependent antibodies, as well as isoform-specific Tau antibodies

Taking all experimental data into consideration, each reagent’s overall performance was categorised as green, amber or red, based on an intuitive “traffic-light system” (Figs. 3, 4, 5, 6 and 7). It is important to note, however, that the conclusions presented here, while based on an extensive data set, rely largely on assessing the performance of most reagents using standardised protocols, with few exceptions, which are clearly indicated. It is possible that variations in the experimental conditions may alter antibody performance and that targeted protocol optimisation (e.g. different antigen retrieval protocols, blocking reagents, antibody concentration, length of incubation steps, etc.) may improve the performance of specific reagents. Antibodies assigned “green” were those found to detect Tau with high selectivity, although not all did so with high sensitivity. Antibody performance was classified as “amber” if the respective antibody detected Tau, but either displayed non-selective reactivity with other proteins, or its performance differed from expected. Antibodies whose performance was categorised as “red” failed to detect Tau, regardless of whether or not they reacted non-selectively with other proteins. Overall, only five antibodies failed to detect Tau altogether in any of the samples tested by WB or IHC. In these cases, it remains possible that lack of signal may reflect the absence of the target epitope(s) from the samples included in our testing, rather than the fact that a given antibody does not bind Tau or is unsuitable for use in the respective assay. To aid interpretation and selection of appropriate reagents based on experimental needs, antibodies classed as “amber” were further split into sub-categories, which are displayed as codes (with associated explanations) on the respective traffic lights. Finally, in-depth interpretations of the experimental data are provided for both WB data (Supp. Table S2) and IHC-IF data (Supp. Table S3).

### Performance of PTM-agnostic “total” Tau antibodies by WB

Of the 24 “total” Tau antibodies tested by WB (Fig. 1; antibody details provided in Supp. Table S1), only one (clone 2G9.F10) failed to detect Tau in any of the samples tested, including when the protein was overexpressed (Supp. Figs. S5d and Supp. Table S2). The remaining antibodies detected Tau by WB, although non-selective cross-reactivity with other murine and/or human proteins was also evident for many of the reagents (Supp. Figs. S4–S7; Supp. Fig. S23; Supp. Table S2). This was observed particularly with the K9JA (Dako, cat. no. A0024) polyclonal antibody in both mouse and human samples (Supp. Fig. S7f; Supp. Fig. S23i). Reducing the primary antibody concentration reduced the intensity of the signals detected with the K9JA, but this decrease was observed equally for non-selective and selective bands. Dilution of the primary antibody was not sufficient to eliminate non-selective bands, even when the primary antibody concentration was reduced to 1 in 10,000 (Supp. Fig. S8a). Furthermore, the pattern and intensity of non-selective bands detected with the K9JA antibody were similar across different blocking reagents, except for 5% skimmed milk, which resulted in overall diminished signal intensity for all bands, including the selective Tau bands (Supp. Fig. S8a).

Despite their status as “PTM-agnostic” antibodies, we found that, for many of the antibodies in this category, the signal intensity of Tau bands detected in SH-SY5Y lysates increased following dephosphorylation with lambda phosphatase (λPP) (Supp. Figs. S4–S7, column V; Supp. Table S2). This was by as much as ~ 5-fold for clone BT2 (Supp. Fig. S5f, column V), 7.7-fold for clone A16097E (Supp. Fig. S7a, column V) and ~ 13-fold for clone 77E9 (Supp. Fig. S5e, column V). In line with this, many antibodies further showed differential reactivity with recombinant pTau that has been phosphorylated by different kinases (Supp. Figs. S4–S7, column IV; Supp. Table S2). Conversely, our data suggest that N-terminal antibodies SP70, Tau-12, and Tau-13, as well as the polyclonal K9JA, raised against the protein’s C-terminal half, were least affected by Tau phosphorylation (Supp. Figs. S4–S7, columns IV, V; Supp. Table S2). Dephosphorylating proteins directly on the WB membrane yielded similar findings. While the D5D8N clone detected Tau similarly on untreated and λPP-treated membranes, signal intensity detected with the BT2 and 77E9 antibody clones increased following λPP treatment of the WB membrane (Supp. Fig. S8b). Notably, there were no cases where Tau immunoreactivity decreased following λPP treatment of the SH-SY5Y lysates (Supp. Figs. S4–S7, column V), suggesting that none of the tested antibodies has a greater affinity for phosphorylated Tau compared to the unphosphorylated protein. Taken together, our results reveal that binding of many “total” Tau antibodies is impacted by the protein’s phosphorylation status, despite the assumption that these reagents bind Tau with equal affinity, irrespective of PTM occupancy. This may be either directly through the presence of phosphate groups hindering antibody binding, or indirectly through phosphorylation events potentially altering Tau conformation.

As detected with the SP70, Tau-12 and Tau-13 antibodies, Tau protein levels in wildtype HAP1 cells were ~ 35–50% of those in SH-SY5Y cells (Supp. Fig. S9a–c). However, intensity of the Tau signal detected in wildtype HAP1 cell lysates with the D5D8N, RD3, and DA9 antibody clones, respectively, was only ~ 6%, 5%, and 3% of that detected in SH-SY5Y cell lysates (Supp. Fig. S9d–f). Furthermore, many of the antibodies that detected Tau in SH-SY5Y lysates, failed to detect the lower levels of Tau expressed in HAP1 cells (e.g., C-term ab254256, 43D, HT7, Tau-5, Tau-46, A16097E, and A16097B) (Supp. Figs. S4–S7, column III; Supp. Table S2), highlighting the importance of antibody choice when analysing samples where Tau is expressed at lower levels, as may be the case for non-brain/non-neuronal samples.

Taken together, our data demonstrate that antibodies SP70, 43D, Tau-12, Tau-13, 77E9, Tau-5 (Abcam), D1M9X, and #ab254256 can detect Tau with high selectivity in samples of both mouse and human origin (Supp. Figs. S4–S7 and Supp. Table S2). However, our data further revealed that 77E9 has much stronger immunoreactivity with Tau in dephosphorylated samples (Supp. Fig. S5f, column V; Supp. Table S2), raising the possibility that it may detect either a dephosphorylated epitope or a conformational epitope that is affected by phosphorylation, highlighting the need for further characterisation of its epitope and performance in different samples. We also found that 43D, Tau-5, D1M9X and #ab254256 failed to detect low, endogenous levels of Tau expression in HAP1 cells, suggesting that their utility may be limited to samples that express Tau at higher levels (column III in Supp. Fig. S4d, Supp. Fig. S6a–c, f, Supp. Fig. S7e; Supp. Table S2). Antibodies SP70, Tau-12 and Tau-13 were found to be selective, sensitive and to react with Tau equally regardless of its phosphorylation status (Supp. Fig. S4c, e, f; Supp. Table S2).

To assess the antibodies’ ability to detect Tau in clinically relevant human brain samples, the immunoreactivity pattern of “total” Tau antibodies was tested in human brain protein extracts from control individuals and donors diagnosed with one of four different tauopathies: AD, PSP, CBD, and PiD. To ensure that samples were comparable across donors, proteins were extracted from the prefrontal cortex of advanced-stage tauopathy donors with confirmed cortical pathology or age-matched control individuals. Given the large number of antibodies tested, we employed RIPA (supplemented with 0.1% SDS) whole-cell protein extracts, to capture both the detergent-soluble and detergent-insoluble Tau pools of brain Tau in one sample. Probing human brain extracts with 15 different “total” Tau antibodies, whose epitopes span the entire length of the protein, revealed immunoreactivity to monomeric, full-length Tau in the 50–70 kDa MW region in all samples (Supp. Figs. S4–S7, column VI; Supp. Fig. S24). In brain extracts from AD patients, many of the antibodies (e.g. 5A6, DA9, Tau-5, D1M9X, Tau-46, C-term ab254256, K9JA) also detected high-MW smears, as well as distinct bands of ~ 150–180 kDa, widely reported features of AD brain protein extracts, which can be attributed to the presence of PHF-Tau aggregates and low-order oligomers, respectively (Supp. Figs. S4–S7, column VI; Supp. Fig. S24). These findings demonstrate effective extraction not only of monomeric Tau but also of AD-associated Tau oligomers and filaments, which largely partition into the “insoluble” fraction mentioned above, thus confirming that RIPA extracts indeed capture both the soluble and insoluble AD Tau pools, as expected. Re-extraction of DNase-treated RIPA pellets through boiling in sample buffer revealed that additional monomeric and aggregated Tau could be recovered in this manner (Supp. Fig. S27), although signal intensities between the “RIPA extract” and the “RIPA pellet” cannot be compared as the dilution factors differ between the two.

**Fig. 8:**
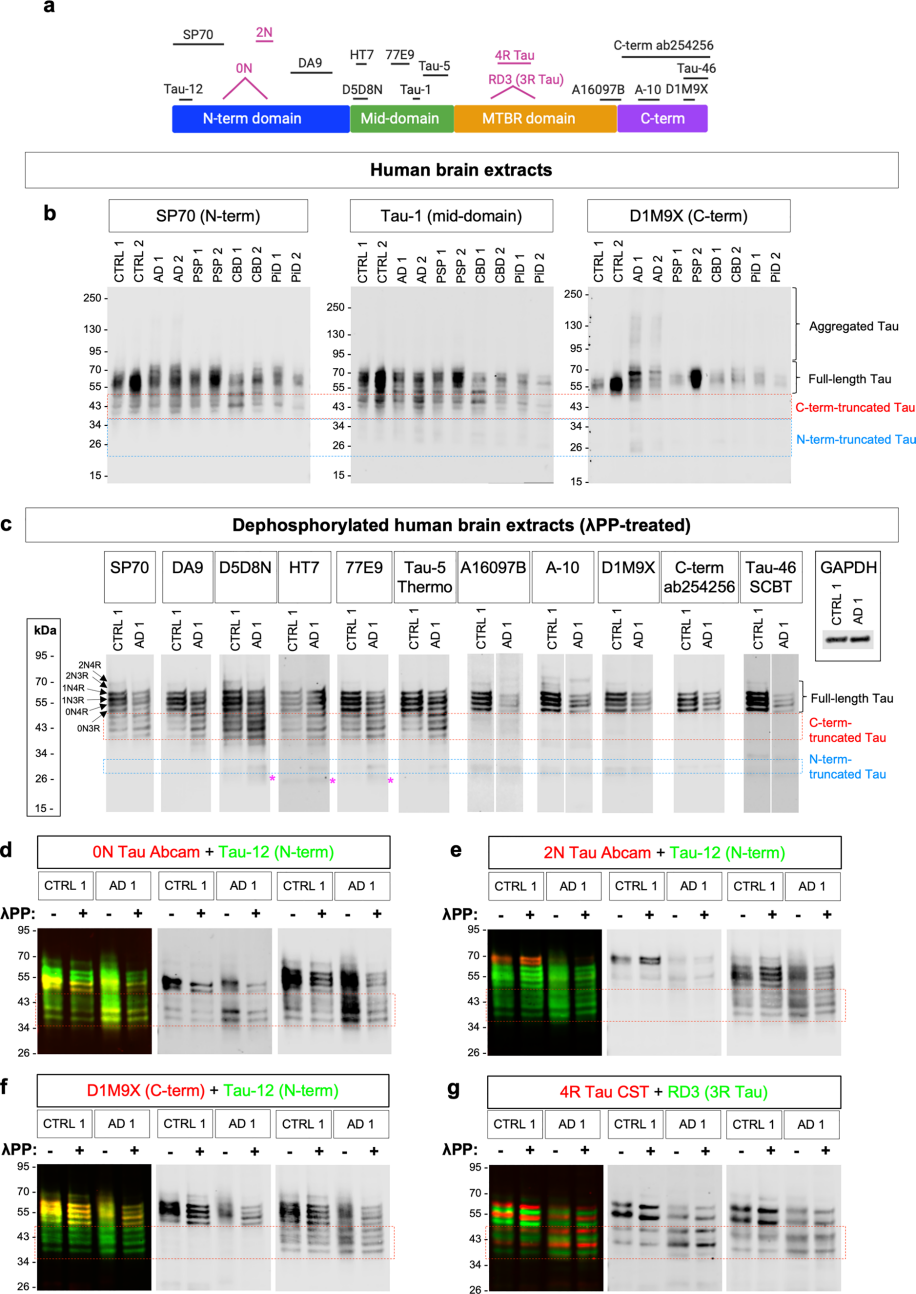
Antibodies binding to the N-term, mid- and MTBR-domains reveal presence of C-term-cleaved Tau variants in human brain extracts from both control and tauopathy donors. **a** Diagram of the main domains of the Tau protein showing the locations of the epitopes of the various antibodies. Antibodies raised against specific splice isoforms are shown in magenta.** b** Tau was detected by WB in human brain protein extracts from control and diagnosed tauopathy donors with antibodies binding close to the N-terminus (SP70), in the mid-domain (Tau-1) or close the C-terminus (D1M9X) of the protein. MW range of full-length Tau isoforms, Tau oligomers and higher order Tau aggregates are indicated. Blue and red boxes outline the MW range where N-terminal-truncated and C-terminal-truncated Tau variants were observed, respectively. **c** Tau was detected in dephosphorylated human brain extracts by WB with a panel of antibodies with epitopes spanning the entire length of the protein. The antibody used to probe each WB membrane is indicated above each respective image. Magenta asterisks indicate putative Tau fragments that lack both the N- and C-termini. **d**–**g** Human brain protein extracts were either dephosphorylated via treatment with λPP ( +) or mock-treated (-) and analysed by fluorescent WB with combinations of mouse (green) + rabbit (red) Tau antibody pairs to assess the overlap between the bands detected with each antibody: 0N Tau isoform-specific antibody (Abcam reagent) + Tau-12 “total” Tau N-term antibody (**d**); 2N Tau Tau isoform-specific antibody (Abcam reagent) + Tau-12 “total” Tau N-term antibody (**e**); D1M9X “total” Tau C-term antibody + Tau-12 “total” Tau N-term antibody (**f**); 4R Tau isoform-specific antibody (CST reagent) + RD3 (3R Tau) isoform-specific antibody (**g**). Individual channels are shown in grayscale (red channel, left; green channel, right)

Probing human brain extracts with a broad panel of antibodies that detect epitopes across the entire length of the protein (Figs. 1c and 5) revealed a striking pattern of immunoreactivity, where antibodies with epitopes falling within the N-terminal domain or Pro-rich mid-domain reacted with a diffuse ladder of overlapping bands in the ~ 38–50 kDa MW range, below the MW range of full-length monomeric brain Tau isoforms (Fig. 8a-b; Supp. Figs. S4–S7; Supp. Fig. S24). In contrast, these signals were not detected with C-term antibodies (Fig. 8a,b; Supp. Fig. S4–S7; Supp. Fig. S24). Dephosphorylating human brain extracts with λPP allowed the separation of different monomeric Tau species into closely-spaced but distinct bands, where the bottom four of the six full-length brain Tau isoforms (corresponding to 0N and 1N variants) could be clearly detected, while the top two bands (corresponding to 2N variants) were much weaker or undetectable, depending on the antibody (Fig. 8c). This is in line with the well-established lower expression of 2N isoforms compared to 0N and 1N isoforms in the adult human brain [39]. Closer inspection of the band patterns detected with different antibodies in λPP-treated control and AD brain extracts established that N-terminal and mid-domain “total” Tau antibodies all detected a series of four closely spaced, but distinct, bands migrating in the ~ 38–50 kDa MW range (Fig. 8c, outlined in red box). These experiments further confirmed that the same bands were not immunoreactive with C-terminal antibodies (Fig. 8c). Of note, the appearance and spacing of these bands mirrored those of the four main full-length Tau isoforms (corresponding to 0N and 1 N Tau variants) present on the same blots in the ~ 50–65 kDa MW range (Fig. 8c). These data demonstrate the presence of C-term-cleaved Tau variants in the brains of both control and tauopathy donors and further suggest that all brain isoforms are consistently cleaved at the same residue. Complementing the presence of C-term-cleaved variants, we further found that mid-domain and C-terminal, but not N-terminal, antibodies detected bands of ~ 30 kDa, albeit weakly, suggesting that these represent lower abundance N-term cleaved fragments (blue box in Fig. 8c). Finally, mid-term antibodies D5D8N, HT7, and 77E9 also detected faint bands of 25–27 kDa estimated MW, which were not unambiguously detected with N-terminal nor C-terminal antibodies, suggesting the presence of low levels of fragments missing both the N- and C-termini (bands marked with magenta asterisks in Fig. 8c).

### Performance of phosphorylation-dependent Tau antibodies by WB

During development and in tauopathies, Tau is hyperphosphorylated [3, 160, 165], spurring the development of many putative phospho-Tau antibodies. We tested 30 phospho-Tau antibodies, of which 25 were developed to detect Tau phosphorylated at single residues, while five (pSer199 + pSer202, pSer202 + pThr205, AT8, AT100, and PHF-1) were expected to detect double phosphorylation events (Fig. 1; antibody details provided in Supp. Table S1). This category includes the E178 antibody clone, marketed as a “total” Tau antibody until 2021 (when we contacted the vendor) and used as such in many studies to date (E178 has been cited in at least 86 published studies [36]), further highlighting the importance of detailed antibody testing.

Except pSer238, which did not detect Tau in any of the samples tested, including in protein extracts from the brains of patients diagnosed with various tauopathies (Supp. Fig. S12d; Supp. Table S2), we provide evidence that the remaining 29 phospho-Tau antibodies tested can detect the target protein by WB, with AT8, AT100 and AT180 only doing so in human brain protein extracts from tauopathy patients, in line with established knowledge that these antibodies only react with pathological Tau (Supp. Figs. S10–14; Supp. Table S2). In the case of pSer238, it was not possible to establish whether this antibody failed to bind phosphorylated Tau or whether Tau phosphorylated at Ser238 was not present at detectable levels in any of the samples tested (Supp. Fig. S12d). Although Ser238 has been reported to be phosphorylated in the brains of AD patients, it is detected in fewer than 10% of donor samples [163].

While evidence of non-selective reactivity was observed for several reagents, 15 (50%) and 12 (40%) of the 30 phospho-Tau antibodies were deemed highly selective for the detection of Tau in mouse brain and human cell extracts, respectively (Supp. Figs. S9–S12, columns II-III; Supp. Table S2). Taken together, however, only 8 (27%) of the phospho-Tau antibodies tested were highly selective for Tau in both mouse and human samples, namely: pThr205, AT8, pSer214, AT100, AT180, pSer262 (BioLegend), pSer396 (Thermo), and pSer404 (Supp. Figs. S10–S14, columns II–III; Supp. Table S2).

It has been suggested that many phospho-Tau antibodies can also react with the corresponding unphosphorylated peptide [50], raising concerns over their specificity for phosphorylated Tau. Of the 30 phospho-Tau antibodies tested here, only the pSer409 antibody reacted weakly with the unphosphorylated Tau ladder (Supp. Fig. S14d, column IV, lane 1), thus confirming that the remaining 29 reagents (97%) do not detect unmodified Tau by WB (Supp. Figs. S10–S14, columns II and IV; Supp. Table S2). Where a phospho-Tau antibody immunoreacted with SH-SY5Y protein extracts, treatment with λPP abrogated this signal, further confirming the requirement for phosphorylation (Supp. Figs. S10–S14, column V; Supp. Table S2).

Comparison of the sites that GSK3β, DYRK1A, and CAMKIIA have previously been reported to phosphorylate on recombinant Tau with the detection patterns observed for the various phospho-Tau antibodies, revealed that the reactivity patterns observed for 12 of the 30 phospho-Tau antibodies tested (namely, pThr181, pSer202 + pThr205, pSer214, AT100, pThr217, pThr231, pSer238, three of four pSer262 antibodies, pSer356 and pSer422) agreed with existing information regarding the phospho-epitopes expected to be present on each of three recombinant pTau variants (Supp. Table S4). The remaining phospho-Tau antibodies: (i) did not yield a detectable signal for one or more of the recombinant pTau variants they were expected to react with (14 antibodies); and/or (ii) reacted with recombinant pTau versions that have not previously been reported to be phosphorylated at the relevant epitopes (5 antibodies) (Supp. Table S4). In scenario (ii), however, unless a given kinase has been specifically reported not to phosphorylate Tau at a certain residue, absence of information cannot be construed as proof that the relevant kinase does not phosphorylate Tau at a given epitope. We highlight the case of the pSer262 Abcam antibody, which was the only one of four pSer262 antibodies that did not detect CAMKIIA-pTau. This antibody detected DYRK1A-pTau, instead, despite the fact that DYRK1A has been expressly reported to not phosphorylate Tau at Ser262 (Supp. Table S4) [91, 124]. These findings raise doubts with regards to the identity of the phospho-epitope detected by the pSer262 Abcam antibody.

We next assessed the phospho-Tau immunoreactivity profiles in human brain protein extracts from control individuals as well as from donors diagnosed with one of four different advanced-stage tauopathies. Given the advanced stage of disease, the latter were expected to contain most phospho-epitopes [81, 163]. In line with this, many of the antibodies displayed strong immunoreactivity with AD brain Tau, with signals detected across the entire MW range, indicating the presence of monomeric, oligomeric and PHF-Tau species, as well as Tau fragments (Supp. Figs. S10–S14, column VI; Supp. Figs. S24–S26). Out of three pSer262 antibodies, only the Abcam reagent immunoreacted strongly with Tau in human brain extracts, where it yielded a signal in nine of the ten brain samples and detected PHF-Tau in AD brain (Supp. Fig. S12f, column VI). Although Tau phosphorylation at Ser262 has been reported to increase in AD [163]—the pSer262 Thermo_2 reagent immunoreacted weakly with AD brain extracts in our testing (Supp. Fig. S13b)—we caution that data obtained with the Abcam pSer262 reagent may not necessarily arise from detection of Tau phosphorylated at Ser262, in view of our finding that it detected DYRK1-pTau, as detailed above (Supp. Fig. S12f, column IV; Supp. Table S4). In our testing, only three of the phospho-Tau antibodies that detected Tau in recombinant pTau form and when overexpressed in HEKs, did not yield detectable signals in any of the human brain samples tested. This was the case for the pSer262 BioLegend, pSer356 and pSer409 reagents (column VI in Supp. Figs S12e, S13c and 14d; Supp. Fig. S25).

Overall, we observed consistently lower immunoreactivity of phospho-Tau antibodies in PSP, CBD, and PiD RIPA brain extracts. Furthermore, phospho-Tau antibodies largely did not detect high-MW Tau aggregates in the primary tauopathy brain samples (Supp. Figs. S10–S14, column VI; Supp. Figs. S24–S26). WB analyses of the re-extracted DNase-treated RIPA pellets revealed immunoreactivity with several phosphorylation-specific antibodies in all tauopathies (Supp. Fig. S27c-h), including AT8-immunoreactive high-MW aggregated Tau (Supp. Fig. S27d). Overall, immunoreactivity signals were lower in the PSP brain samples, which may reflect the lower load/frequency of Tau lesions in these samples compared to the other tauopathies.

While most antibodies continued to show the highest immunoreactivity with the protein extracts from RIPA pellets from AD brain samples, the pSer214 antibody immunoreacted strongest with pellet proteins from the two PiD brain samples (Supp. Fig. S27e). Of note, very low to undetectable immunoreactivity was observed for pSer214 in the RIPA-soluble brain extracts (Supp. Fig. S11e, column VI; Supp. Fig. S25). Taken together, these data suggest that the phospho-epitope detected by the pSer214 antibody is specifically increased in PiD and may be a molecular feature that can distinguish this disease from other tauopathies.

### Performance of other PTM-dependent Tau antibodies by WB

Tau-1 is an antibody widely employed to detect Tau that is not phosphorylated at residues Ser195 to Thr205, located in the mid-domain of the Tau protein, a region that includes the AT8 epitope (Fig. 1; Supp. Table S1). Tau-1 reacted strongly with the unphosphorylated, recombinant Tau ladder (Supp. Fig. S15a, columns II and IV), and detected Tau in biological samples with high selectivity (Supp. Fig. S15a, columns I-III; Supp. Table S2). Treating SH-SY5Y lysates with λPP resulted in a ~ sixfold increase in the intensity of the immunoreactive signal, in agreement with the dephosphorylation-dependent nature of its epitope (Supp. Fig. S15a, column V; Supp. Table S2). Taken together, our data support the widely acknowledged status of Tau-1 as an antibody that detects the dephosphorylated versions of both murine and human Tau proteins with high selectivity. In human brain extracts, Tau-1 reacted with monomeric full-length Tau as well as C-terminal and N-terminal cleaved variants (Fig. 8b; Supp. Fig. S15a, column VI; Supp. Fig. S26).

Early stages of AD are characterised by the proteolytic cleavage of Tau at Asp421 by caspases, giving rise to ΔTau, which can adopt the MC1 pathological conformation and “seed” aggregation [122]. Whether this event contributes to pathology or is a protective response to early pathological changes remains a matter of debate [19, 104, 122]. Given the biological relevance of this PTM, we tested the reportedly highly selective D421 antibody clone, raised against Tau cleaved at Asp421 [59]. While no immunoreactivity was observed in lysates from HEK293T Tau-overexpressing cells, mouse brain, SH-SY5Y or HAP1 cells (Supp. Fig. S15b, columns I-IV; Supp. Table S2), we found that the D421 antibody: (i) detected Tau in SH-SY5Y lysates following λPP treatment (Supp. Fig. S15b, column V) and (ii) immunoreacted weakly with full-length, recombinant Tau (Supp. Fig. S15b, column IV). Taken together, our data demonstrate that binding of the D421 clone to Tau is inhibited by phosphorylation and, importantly, that this antibody is not specific to Tau truncated at Asp421, given the observed immunoreactivity to full-length recombinant Tau.

An acetyl-Lys174 antibody, raised against Tau acetylated at Lys174, did not immunoreact with any of the samples tested, including human brain extracts from late-stage AD donors, where Tau acetylation at Lys174 has been previously reported [105] (Supp. Fig. S15c; Supp. Table S2). Taken together, these data suggest that the acetyl-Lys174 antibody tested here may not be suitable for use in WB, although we cannot rule out the possibility that further protocol optimisation may be required for this reagent or that the target epitope was present at levels below the detection limit. We note that previous studies that have used antibodies to detect Tau acetylated at Lys174 have employed an in-house generated reagent [105, 117] and, importantly, that not all studies have detected this modification in AD patient brain samples, even when employing highly sensitive detection techniques [163], suggesting that it may either be present at very low levels or that its levels are highly variable across donor populations.

The Tau-2 antibody clone, originally raised against bovine Tau, has been reported to detect a conformational epitope [111, 159]. Despite the fact that the human Tau sequence harbours an amino acid substitution at a key residue within the Tau-2 epitope, it has been proposed that Tau hyperphosphorylation, as seen under pathological conditions such as AD, can alter Tau conformation and restore the Tau-2 epitope, resulting in Tau-2 immunoreactivity with pathological, but not normal, human Tau [85], In our initial testing, Tau-2 failed to detect Tau by WB in any of the samples tested, including human brain protein extracts from tauopathy donors (Supp. Fig. S15d; Supp. Table S2). Further protocol optimisation revealed weak Tau-2 immunoreactivity in human brain protein extracts from two donors when detergents were omitted entirely during probing of the WB membrane (Supp. Fig. S15e), in line with previous reports that Tau-2-immunoreactivity is abrogated by exposure to detergent [133]. Tau-2-immunoreactive bands were present in the MW range of Tau monomers but not high MW oligomers. Unexpectedly, neither of the two donors had been diagnosed with AD, with one having been diagnosed with PSP and the other being a control donor, thus raising the possibility that Tau species that are immunoreactive with Tau-2 may also be present in the brains of some control donors.

### Performance of oligomeric Tau antibodies by WB

Targeting Tau species of pathological and diagnostic relevance, the T22 and the Tau oligomer-specific monoclonal antibody 1 (TOMA-1) antibody clones were raised by using in vitro-generated recombinant 2N4R Tau oligomers as immunogens and are thought to detect Tau only when the protein exists in low-order oligomeric aggregates, but not in monomeric form nor in filamentous aggregates [29, 87]. No TOMA-1-immunoreactive bands were detected in any of the samples tested, including human brain protein extracts from tauopathy donors (Supp. Fig. S16a; Supp. Table S2). These findings support the selectivity of the TOMA-1 antibody but offer no information regarding its ability to detect Tau oligomers.

Conversely, the T22 antibody reacted with the Tau ladder and detected monomeric Tau by WB in all biological samples tested, including human brain extracts from control donors (Supp. Fig. S16b; Supp. Fig. S26; Supp. Fig. S27k; Supp. Table S2). In line with this, T22 immunoreactivity in human brain extracts largely mirrored that of “total” Tau antibodies, e.g. Tau-12 or K9JA Dako, such that the majority of the T22 signal originated from detection of Tau monomers accompanied by faint detection of high-MW Tau in AD brain samples (Supp. Fig. S16b, column VI; Supp. Fig. S26). Finally, while absence of T22-immunoreactive bands in *Mapt*^*−/−*^ mouse brain lysates support this reagent’s selectivity for Tau in mouse samples (Supp. Fig. S16b, column II), non-selective reactivity was observed in human cell lysates (Supp. Fig. S16b, column III; Supp. Fig. S23u-v). Taken together, our data demonstrate that T22 can detect monomeric Tau and is, therefore, not selective for Tau oligomers under denaturing WB conditions.

The T22 clone is also available commercially as an affinity chromatography-purified version (hereafter, T22-purified). Similar to the “original” T22 version described above, T22-purified displayed non-selective cross-reactivity to other proteins (including to low-MW bands in human brain extracts from all donors), but, in contrast to the “original” version, it did not react with monomeric Tau (Supp. Fig. S16c; Supp. Table S2). Importantly, like TOMA-1, T22-purified also failed to detect Tau oligomers in human brain extracts (Supp. Fig. S16c, column IV; Supp. Fig. S26). These data suggest that, under the experimental conditions employed here, T22-purified does not display detectable reactivity with Tau monomers, although it may bind non-selectively to proteins other than Tau. We note, however, that T22-purified has been cited in only two studies to date, with most studies citing the use of the commercial product having used the “original” version of this product described above.

We were intrigued by the unexpected finding that neither TOMA-1 nor T22-purified detected oligomeric Tau in tauopathy brain extracts, given that many of the “total” Tau and phospho-Tau antibodies, including the well-established AT8 and AT180 antibodies, immunoreacted strongly with high-MW Tau species in AD brain extracts (column VI in Supp Figs. S4-S7 and Supp. Figs. S10-S14; Supp. Figs. S24-S26). Many of these also reacted with a focalised signal of ~ 150–180 kDa (Supp. Figs. S24-S26), which coincides with the reported MW for low-order Tau oligomers, the very species that T22 and TOMA-1 have been reported to bind to [29, 87], demonstrating that aggregated Tau species, including Tau oligomers, were indeed extracted efficiently from AD human brains. Nonetheless, to address the possibility that the target Tau oligomers might have remained in the RIPA pellet, we further tested the immunoreactivity of oligomeric Tau antibodies by WB to proteins re-extracted from the DNase-treated RIPA pellets. The “original” T22 antibody once again displayed an immunoreactivity pattern similar to that observed with “total” Tau antibodies, with the strongest reactivity observed with monomeric Tau (Supp. Fig. S27k). In the case of membranes probed with the TOMA-1 and T22-purified antibodies, faint smears were observed across the entire MW range, but no distinct reactivity with high-MW oligomeric Tau was distinguishable above this background (Supp. Fig. S27l-m). These findings confirm that TOMA-1 and T22-reactive oligomers were not lost in the RIPA pellet.

Both antibodies were originally reported to preferentially detect PBS-soluble oligomeric Tau by WB under semi-denaturing conditions [29, 87], with immunoreactivity lost when samples were subjected to harsh denaturing conditions (8 M urea). Semi-denaturing WB analysis of PBS-soluble human brain extracts from one control and the two AD cases revealed immunoreactivity with the SP70 “total” Tau antibody in all three samples and with the AT8 antibody in AD samples only, as expected (Supp. Fig. S16d-e). Furthermore, AT8 detected two high-MW bands of ~ 150 kDa and ~ 180 kDa (Supp. Fig. S16e), which match the MWs reported for low-order oligomers [86]. TOMA-1 detected a multitude of bands, including a prominent band of ~ 130 kDa (Supp. Fig. S16f). However, unlike the AT8 signals, these were present in all donors (Supp. Fig. S16f), with signal intensity being strongest in the control donor, which we note was also the sample that had the highest concentration of Tau overall (Supp. Fig. S16d). In contrast, T22-purified did not yield distinguishable high-MW signals, except for weak immunoreactivity with a smeared signal observed in the control brain. These data establish that many signals, including high-MW signals, can be detected with TOMA-1 in PBS-soluble brain extracts under semi-denaturing SDS-PAGE conditions, but the identity of the high-MW bands remains unclear given their presence in both control and AD brains. The discrepancy between the data presented here and previously published results may stem from technical differences or from differences in the biological samples employed here compared to previous studies, as physiological and pathological Tau levels vary widely between donors and across brain regions.

### Performance of isoform-specific Tau antibodies by WB

The expression of Tau splice isoforms is regulated in a developmental-, tissue- and disease-dependent manner [6, 38, 39, 66, 90, 112, 143, 146]. In the adult human brain, distortion of the ratios between different isoforms has been reported to correlate with, and even to cause, neurodegeneration [27, 31, 34, 46, 62, 107, 142, 166]. There is, therefore, strong research interest in identifying antibodies that can be used to reliably detect and quantify the various Tau splice isoforms. In this category, we include antibodies purposefully developed to recognise specific Tau splice isoforms, as well as antibodies identified as targeting certain splice isoforms in the course of this work (Fig. 1; antibody details provided in Supp. Table S1).

Of the two 0N Tau antibodies tested, the Abcam reagent detected its target isoform and did so with high selectivity (Supp. Fig. S17a-b; Supp. Table S2). This reagent further immunoreacted strongly with Tau in human brain extracts (Fig. 8d; Supp. Fig. S17b, column VI; Supp. Fig. S26). Employing fluorescent WB detection to combine the 0N Tau Abcam antibody with Tau-12 demonstrated that the bottom two of the four C-term cleaved Tau variants observed with “total” Tau antibodies in human brain extracts are indeed 0N Tau variants, as hypothesised above (Fig. 8d).

The only currently available antibody against human 1N Tau displayed high selectivity in both mouse and human samples, but only weak immunoreactivity to the target isoform in the Tau ladder and in human brain extracts (Supp. Fig. S17c; Supp. Table S2), despite the fact that 1N and 0N Tau isoforms are present at comparably high levels in human brain extracts (Fig. 8c).

Both 2N Tau antibodies tested detected the target isoforms, but the BioLegend reagent reacted with prominent non-selective bands, while the Abcam reagent detected 0N Tau overexpressed in HEK293T cells (Supp. Fig. S17d-e; Supp. Fig. S19k-l; Supp. Table S2). In human brain extracts, the Abcam 2N Tau antibody detected two doublets of bands, with the strongly immunoreactive top doublet corresponding to full-length 2N Tau isoforms, while the fainter bottom doublet overlaps with 0N Tau bands (Fig. 8e; Supp. Fig. S17e, column VI; Supp. Fig. S26). Taken together, our data suggest that the Abcam 2N Tau antibody has a high affinity for 2N isoforms, but can also detect other Tau isoforms, depending on expression levels.

Despite being marketed as a “total” Tau antibody, we found that antibody Abcam #ab109392 (clone name: EPR2396(2)) detected only the 1N and 2N, but not 0N, splice isoforms of human Tau, with varying selectivity depending on the origin of the samples being tested (Supp. Fig. S17f; Supp. Fig. S19a; Supp. Table S2). Taken together, our data suggest that its epitope lies within the first N-terminal insert encoded by exon 2 (Fig. 1). We note that the same antibody clone, EPR2396(2), is marketed by other suppliers (e.g., Origene cat. no. TA307184 and GeneTex cat. no. 62576) as a phospho-Thr50 Tau antibody, a residue encoded by *MAPT* exon 2. However, as this antibody detected isoforms of the recombinant Tau ladder and reacted equally with all three recombinant phosphorylated Tau versions (Supp. Fig. S17e, column IV), we conclude that its binding is not phosphorylation-dependent.

The RD3 antibody clone, raised against 3R Tau, detected Tau with high selectivity, in both mouse and human samples, and did not appear to be affected by Tau phosphorylation (Supp. Fig. S18a; Supp. Table S2). Lack of RD3 immunoreactivity with wildtype murine brain Tau (Supp. Fig. S18a, column II) is in agreement with published data that adult wildtype mouse brains express 4R Tau exclusively [103]. RD3 immunoreactivity in human brain extracts was observed for all donors, but this was unexpectedly lowest for PiD brain extracts (Supp. Fig. S18a, column VI; Supp. Fig. S26). We hypothesised that, although AD brain Tau was extracted with high efficiency in SDS-containing RIPA, Pick’s bodies may not be solubilised efficiently with this protocol. Confirming this possibility, WB analysis of proteins re-extracted from the DNase-treated RIPA pellet revealed strong RD3 immunoreactivity with PiD Tau and AD Tau, with only faint immunoreactivity with PSP and CBD Tau (Supp. Fig. S27i).

Of the five 4R Tau antibodies tested, all of them detected the target protein isoforms in recombinant form or when the protein was overexpressed (Supp. Fig. S18b-f; Supp. Fig. S19m-p; Supp. Table S2), but only the Millipore clone 1E1/A6 (hereafter RD4), Abcam and CST reagents were able to detect Tau expressed in adult mouse brain, where all three displayed high selectivity (Supp. Fig. S18b-f, column II). Although undifferentiated SH-SY5Y cells are often presumed to express only the 0N3R isoform, expression of 4R Tau has also been reported in these cells, albeit at much lower levels than 3R Tau [1, 135, 140]. We confirmed low level 4R Tau expression in SH-SY5Y cells and, although proximity of the bands precluded precise quantification, estimated that 4R isoforms account for ≤ 10% of the “total” Tau signal detected in SH-SY5Y cells (Supp. Fig. S19b-d). Taken together, our data indicate that the RD4 reagent detects both murine and human 4R Tau with high selectivity, but yields low signal intensity, while the Abcam reagent detects murine and human 4R Tau isoforms with high sensitivity, but may also react with proteins other than Tau, and should therefore be tested carefully in each new sample type. Finally, the CST 4R Tau antibody detected its target isoform with high selectivity and yielded high signal intensities, suggesting it would be the reagent of choice for a variety of samples. Both the RD4 and CST reagents displayed immunoreactivity to Tau in human brain extracts, with signal intensity being weakest or undetectable in PiD (3R tauopathy) brain extracts, as expected (Supp. Fig. S19c, f, column VI; Supp. Fig. S26). However, neither detected aggregated 4R Tau in PSP and CBD brain extracts as expected for these two tauopathies where aggregates consist of 4R Tau exclusively (Supp. Fig. S19c, f, column VI; Supp. Fig. S26). In protein extracts from DNase-treated RIPA pellets, however, the CST 4R Tau antibody displayed strong immunoreactivity to PSP and CBD Tau, as predicted (Supp. Fig. S27j). Utilising fluorescent WB detection to combine the RD3 3R Tau antibody with the 4R CST Tau reagent, enabled us to establish that the four C-term-cleaved Tau variants, detected with “total” Tau antibodies on WB of phosphatase-treated human brain extracts, comprise of two 3R Tau bands and two 4R Tau bands (Fig. 8g). These data confirm the hypothesis that the C-term-cleaved variants mirror the isoform compositions of the full-length Tau species found in human brain.

Despite having been marketed as an antibody that “detects Tau, whether phosphorylated or unphosphorylated on Ser622” [118], our testing established that the Abcam #ab76128 reagent (clone name: EP2456Y; hereafter referred to as the “Ser622 antibody”) had a much higher affinity for 4R Tau compared to 3R Tau, and showed non-selective reactivity with other human proteins (Supp. Fig. S19e-g; Supp. Table S2). Potentially explaining this reagent’s preference for 4R Tau, the Big Tau/PNS-Tau (Uniprot ID: P10636-1) Ser622 residue corresponds to Ser305 in the 2N4R Tau isoform, the last residue encoded by the alternatively spliced exon 10, whose inclusion gives rise to 4R Tau (Fig. 1B). In light of our findings, results obtained through using this antibody should be interpreted with caution as it is neither a “total” nor a truly isoform-specific Tau antibody.

Similar to “total” Tau antibodies, isoform-specific antibodies are assumed to detect the target protein regardless of phosphorylation status. By comparing antibody reactivity to recombinant Tau that had been phosphorylated by different kinases or to SH-SY5Y cell lysates prior to or after λPP treatment, our data suggest that the binding of the Abcam 0N, BioLegend 2N, Millipore RD4, BioLegend 4R and CST 4R Tau antibodies is partially attenuated by phosphorylation (Supp. Figs. S14-S15, columns IV, V; Supp. Table. S2).

### Performance of Big Tau antibodies by WB

Inclusion of exon 4A increases considerably the MW of the resulting Tau protein variants, variably referred to as Big Tau or PNS-Tau in the literature. With predicted MWs of 78.9 kDa and 76.2 kDa for the largest Big Tau isoforms in human and mouse, respectively, these have been found to migrate on SDS-PAGE to apparent MWs in the 100–125 kDa range. Despite having been cloned for the first time in 1992 and its abundance in the PNS, retina and skeletal muscle, the functions of Big Tau have remained virtually unexplored [53, 54]. This is, in great part, due to the lack of reagents that can be used to reliably detect Big Tau variants. Of the currently available antibodies, only one was specifically developed to recognise Big Tau (Ximbio, cat. no. 154129, polyclonal antibody) (Supp. Table S1) [24]. Through analysing immunogen information for a large number of Tau antibodies, we established that four other commercially available reagents have also been raised against Big Tau-defining exon 4A sequences, although all of these are currently marketed as “total” Tau antibodies (Supp. Table S1, all polyclonal). Surprisingly, three of these reagents are the Tau antibodies developed by the Human Protein Atlas, an ambitious large-scale project that aims to map all human proteins in cells, tissues and organs, and have been used to report Tau expression in many tissues outside the brain (Supp. Table S1) [144]. Finally, a fifth Big Tau antibody was developed by St John’s Laboratories (STJ, cat. no. STJ98827).

As expected, none of the Big Tau antibodies reacted with Tau that lacks exon 4A (Supp. Fig. S20; Supp. Table S2b). While the STJ reagent failed to detect Big Tau, even when overexpressed, we provide evidence that the remaining antibodies detected the target Tau isoforms in biological samples (Supp. Figure 20a-e; Supp. Table S2b). Taken together, our data suggest that Big Tau is expressed in the wildtype mouse brain. Although bands of the predicted MW were observed in SH-SY5Y lysates, the identity of these bands could not be established due to the extensive non-selective reactivity that characterised all antibodies in this category. Big Tau antibodies with improved selectivity in human samples are urgently needed in order to allow biological studies into the physiological roles of Big Tau and its potential involvement in disease.

### Several Tau antibodies cross-react with MAP2 giving rise to mid-MW bands that may interfere with the detection of 2N Tau isoforms

The C-terminal half of Tau shares extensive sequence homology with related microtubule-associated proteins (MAPs), particularly MAP2 and MAP4 [45]. Antibodies whose epitopes lie in the C-terminal half of the protein may, therefore, cross-react with other MAPs. In line with this and in agreement with previous reports [20, 26, 69, 101], we observed that all three Tau-46 antibody clones tested detected non-specific bands whose MWs are consistent with those reported for the MAP2a/MAP2b (> 250 kDa in mouse brain and SH-SY5Y extracts) and MAP2c (75–90 kDa in SH-SY5Y extracts) isoforms, respectively (Supp. Fig. S7b-d; Supp. Fig. S22a; Supp. Table S2). While cross-reactivity with the high-MW MAP2a/b isoforms does not interfere with the detection of monomeric Tau on SDS-PAGE, these may overlap with oligomeric/aggregated Tau species. Of greater concern, mid-MW MAP2c isoforms migrate at an apparent MW similar to that of 2N Tau isoforms. As undifferentiated SH-SY5Y cells only express detectable levels of 0N Tau isoforms, this enabled the clear distinction between Tau and mid-MW MAP2c bands. However, caution should be exercised when analysing lysates from cells or tissues that express 2N Tau isoforms, as non-specific MAP2 signals may overlap the Tau bands.

Aligning the amino acid sequences of the 2N4R Tau and MAP2c isoforms highlighted the extent of sequence homology between the C-terminal halves of the two proteins (Supp. Fig. S21a) and raised the possibility that other C-terminal “total” Tau antibodies, as well as some phospho-Tau antibodies, may also cross-react with MAP2, while N-terminal as well as 3R and 4R Tau antibodies are not expected to do so. By combining Tau antibodies and using MAP2 antibodies that do not cross-react with Tau (Supp. Fig. S22b-o), we established that the Tau-46, K9JA, A16097B, pSer199, pSer202 + pThr205, and pSer396 Tau antibodies all reacted with overlapping mid-MW bands that were also immunoreactive with MAP2 antibodies (Supp. Fig. S22e-j’). Furthermore, clones K9JA, A16097B and Tau-46 detected the purified, recombinant MAP2 protein on WB (Supp. Fig. S22p). In contrast, the Tau-13 and SP70 N-terminal antibodies, the C-term ab254256 antibody, and the pSer198 and E178 phospho-Tau antibodies did not cross-react with MAP2 (Supp. Fig. S22k-n, p), consistent with the predictions of sequence alignments (Supp. Fig. S21a). Of note, sequence similarity raises the possibility that other phospho-Tau antibodies (e.g., pThr205, pThr231, pSer262, pSer409, pSer422) may also cross-react with MAP2 (Supp. Fig. S21a), but it was not possible to test this prediction experimentally, as most of these antibodies did not provide a positive signal in SH-SY5Y cell lysates. For phosphorylation-dependent antibodies, whether they detect MAP2 in biological samples will depend on whether the protein is phosphorylated at the relevant residues in the samples being analysed. Taken together, our data demonstrate that several mid-domain and C-terminal Tau antibodies cross-react with MAP2, whereas antibodies whose epitopes lie in the N-terminal half of Tau, as well as 3R- and 4R-targeting antibodies, do not.

As with MAP2, Tau also shares sequence similarities with MAP4, albeit to a lesser extent [45]. Alignment of the human Tau and MAP4 amino acid sequences revealed extensive similarities between the two proteins in the region encoded by Tau residues 251–358, including most of the sequence encoded by the 4R-defining exon 10, with a stretch of 17 identical residues (Gln288-Gly304 in the human 2N4R Tau sequence) (Supp. Fig. S21b). This suggested that, of the antibodies tested, only the polyclonal K9JA “total” Tau antibody and the antibodies targeting pSer262 may potentially cross-react with MAP4, as well as some 4R isoform-specific Tau antibodies, depending on the exact location of their epitope. In SH-SY5Y and HAP1 cell lysates, the K9JA and Ser622 antibodies cross-reacted with a band just below the 250 kDa MW marker (Supp. Fig. S19e, column III; Supp. Fig. S23i), a MW consistent with that of the MAP4 protein. Similar to MAP2, cross-reactivity of 3R Tau antibodies with MAP4 is not expected (Supp. Fig. S21b).

### Performance of PTM-agnostic “total” Tau antibodies by IHC-IF

Of the 12 “total” Tau antibodies tested by IHC-IF, all detected a positive signal in rTg4510 mouse brain sections, except for clone 77E9 (Supp. Fig. S28; Supp. Table S3). Despite the well-documented limitations of the rTg4510 and other human Tau-expressing mouse models, they were included in our antibody testing workflow as a “positive control” i.e. rTg4510 mouse brain sections represented biological samples where Tau is expressed at very high levels (as much as 13-fold higher than endogenous Tau by 2 months of age) [126]. These data, therefore, demonstrated the ability of the respective “total” Tau antibodies to detect human Tau in FFPE tissue sections, at least when the target protein is present at high levels. Dephosphorylation of rTg4510 mouse brain sections did not noticeably alter the pattern or intensity of labelling obtained with most “total” Tau antibodies in IHC-IF (Supp. Fig. S28), except for the Abcam Tau-5 clone, whose labelling intensity increased following λPP treatment (Supp. Fig. S28k-k’, Supp. Fig. S29). These findings are largely consistent with our WB observations reported above. However, in contrast with our WB findings which revealed that dephosphorylation increased immunoreactivity of the 77E9 clone by 11-fold (Supp. Fig. S5e; Supp. Table S2), labelling with 77E9 remained absent in rTg4510 mouse brain sections following λPP treatment (Supp. Fig. S28h-h’). This could be interpreted to suggest that 77E9 is not suitable for IHC applications. Alternatively, lack of 77E9 immunoreactivity in rTg4510 brain sections may result from peculiarities specific to this mouse model, where the development and drivers of pathology are fundamentally different compared to human tauopathies [126]. In support of the latter possibility, labelling human AD brain sections with 77E9 revealed strong immunoreactivity following λPP treatment, with no detectable signal in untreated sections (Supp. Fig. S30a-a’; Supp. Table S3), thus demonstrating its suitability for IHC applications and confirming that prior dephosphorylation of the samples is required for efficient labelling with this reagent.

Through comparing the labelling observed in brain sections from wildtype, *Mapt*^−/−^ and hTau mice, we established that antibodies SP70, Tau-12, Tau-13, HT7, K9JA and Tau-5 detect Tau with high selectivity (Supp. Fig. S31a-f”; Supp. Table S3), although our data further suggest that HT7 and Tau-5 may not be suitable for detecting low level expression of Tau (Supp. Fig. S31d”, f’’). The high selectivity of K9JA and HT7 in IHC-IF was in contrast to the extensive non-selective binding observed for both antibodies by WB (Supp. Fig. S31d-e”; Supp. Table S3; Supp. Fig. S5b; Supp. Fig. S7f; Supp. Table S2), an observation that may be explained by the fact that many more epitopes are exposed and therefore accessible for antibodies to bind in denatured WB samples. Tau-46 displayed non-specific cross-reactivity with a protein localising to the apical dendrites of hippocampal pyramidal neurons (Supp. Figure 31 g-g”; Supp. Table S3), a labelling pattern reminiscent of that previously reported with anti-MAP2 antibodies [79]. Based on knowledge of its epitope, together with our WB data above (Supp. Figs. S21-S22), we propose that the Tau-46 immunoreactivity observed in wildtype, *Mapt*^*−/−*^ and hTau mouse brain sections is a result of antibody cross-reactivity with MAP2, suggesting that even in wildtype brains, the endogenous Tau signal detected with this antibody is comparatively weak.

As the conformational and PTM state of Tau in the mouse brain does not replicate those found in human brain, we assessed the immunoreactivity of the human-specific Tau-12, SP70 and HT7, as well as that of the Tau-5, “total” Tau antibodies in FFPE brain sections from human donors diagnosed with AD, PSP and PiD (Supp. Fig. S32). These three tauopathies are characterised by distinct Tau lesions, including in terms of isoform composition, structural conformations, PTM profiles and affected cell types. Of these, only SP70 labelled Tau lesions in all three tauopathies (Supp. Fig. S32b-b’’), Tau-12 and HT7 did not label Tau in PSP lesions (Supp. Fig. S32a-a’’, c–c’’), while Tau-5 labelled PSP lesions, where it yielded a strong immunoreactive signal, but failed to label Tau in AD and PiD lesions altogether (Supp. Fig. S32d-d’’). The differential immunoreactivity profiles of PHF-Tau in the three tauopathies is likely to arise from a combination of different Tau PTM profiles, Tau conformations and types of protein aggregates.

### Performance of phosphorylation-dependent Tau antibodies by IHC-IF

As a consequence of overexpression of human tau carrying the P301L mutation, rTg4510 mice display accumulation of hyperphosphorylated Tau and develop robust neurofibrillary tangle (NFT)-like pathology at 4–5 months of age [120, 137]. Many of the phospho-Tau antibodies were, therefore, predicted to provide a positive signal in rTg4510 mouse brain sections. Despite the well-established limitations of human Tau-expressing mouse models, rTg4510 mouse brains display robust accumulation of hyperphosphorylated Tau and widespread NFT pathology [126]. Brain tissue from this mouse model was thus employed as a first-pass test towards assessing the ability of phospho-Tau antibodies to detect phosphorylated Tau by IHC-IF in FFPE tissue sections where the protein is present at very high levels. As expected, all 16 phospho-Tau antibodies in our panel yielded clear, positive immunoreactivity in brain sections from rTg4510 mice (Supp. Fig. S31; Supp. Table S3), demonstrating their ability to react with phosphorylated Tau in FFPE tissue sections when the target epitope is present at high levels.

Following λPP treatment, immunoreactivity of most phospho-Tau antibodies in rTg4510 brain sections was either abrogated or diminished, supporting the phosphorylation-dependence of these epitopes, while raising the possibility that several residues were incompletely dephosphorylated under the conditions employed here (Supp. Fig. S33; Supp. Table S3). Unexpectedly, however, signal intensity obtained with pSer199 remained unaltered following λPP treatment (Supp. Fig. S33c-c’), while that obtained with pSer396 and E178 unexpectedly increased (Supp. Fig. S33l-l’, n–n’). These results are in contrast to our WB data, where λPP treatment abrogated immunoreactivity for all three antibodies (Supp. Figs. S10d, S13d-e). We hypothesise that not all phospho-epitopes – most of which are found in PHF-like aggregates in rTg4510 mouse brains, as demonstrated by co-localisation with Thioflavin S (ThS)-positive protein aggregates (Supp. Fig. S34) – are accessible to phosphatases, thus rendering them partially resistant to λPP treatment. While λPP has been used successfully in vitro to dephosphorylate PHF-Tau extracted from AD human brains [71], to the best of our knowledge, it has not previously been used to dephosphorylate Tau in situ and, thus, its efficiency towards different types of Tau aggregates in FFPE tissue sections is yet to be characterised. It is likely that the efficiency of λPP-mediated Tau dephosphorylation will vary depending on the origin of the samples, given the differences in the types of aggregates and Tau conformations between the rTg4510 mouse brain compared to human tauopathies. Indeed, labelling AD human brain sections with the pSer199 antibody, which showed equal immunoreactivity prior to and following dephosphorylation in the rTg4510 mouse brain (Supp. Fig. S33c-c’), resulted in a greatly diminished signal intensity in λPP-treated samples (Supp. Fig. S30c-c’). Similarly, higher efficiency of λPP dephosphorylation was observed in human AD brain FFPE sections at the pSer198 and pSer262 Thermo_1 epitopes, with λPP treatment effectively abrogating the strong signals detected with both antibodies in AD human brain (Supp. Fig. S30b-b’, d-d’), whereas these were diminished but not entirely abrogated in rTg4510 mouse brain (Supp. Fig. S33b-b’, k-k’). Moreover, to follow-up on the intriguing observation that E178 reactivity increased in λPP-treated rTg4510 brain sections, we labelled human brain sections from control individuals and tauopathy patients with E178 and AT8, the latter serving as a “positive control” antibody whose performance in human brain has been extensively characterised. In untreated human brain sections, positive immunolabelling was observed with both antibodies in AD and PiD brains, and, to a smaller extent, in the GGT brain, which originated from a GGT type II patient with low-grade Braak pathology (Supp. Fig. S35a). Similar to our observations in rTg4510 mouse brain sections, treatment with λPP abrogated AT8 labelling in all cases, but increased the labelling intensity obtained with E178 in AD and GGT brains, while E178 labelling in PiD brains was unaffected (Supp. Fig. S35a). We hypothesise that the differential impact of λPP in different diseases may result from differences in the Tau PTM profile, Tau conformation and/or types of the Tau-containing aggregates. Co-labelling with ThS confirmed that AT8 and E178 signals co-localised with ThS-positive protein aggregates present in human AD brains (Supp. Fig. S35b). Altogether, our data suggest that phosphorylation at the pSer396/E178 epitope is resistant to phosphatase treatment in PHF-Tau aggregates. We further advance two potential explanations for the increase in signal intensity observed with the E178 and pSer396 antibodies following λPP treatment: (i) it is possible that, prior to dephosphorylation, binding of these antibodies is inhibited by a nearby phosphorylation event at a λPP-accessible residue, thus resulting in improved binding when phosphorylation at this residue is removed following λPP treatment; (ii) alternatively, removal of phosphate groups from other residues on the protein may drive a conformational change that enhances the immunoreactivity of some pSer396-targetting antibodies.

As Tau lesions from transgenic mouse models differ from pathological Tau deposits found in human brains, we further tested 14 of the 16 phospho-Tau antibodies in FFPE human brains sections from patients diagnosed with advanced-stage AD, PSP or PiD (Supp. Figs. S36-S38). We found that all 14 reagents detected Tau lesions in all three tauopathies, thus establishing these reagents’ ability to detect pathological Tau in clinically relevant samples where the protein is present in disease-specific conformational folds and aggregation states. While differences in signal intensity were observed for some antibodies between the different disease states, such differences may not necessarily directly correlate with the levels of the respective phospho-epitope, as epitopes can be masked or exposed to different degrees depending on the type of protein aggregates and disease-specific Tau conformational folds. Overall, we note that all of the antibodies labelled somatodendritic PHF-Tau in AD, where many also labelled neuropil threads and dystrophic neurites. In PSP, labelling mostly localised to oligodendroglial coiled bodies. Finally, in PiD, Pick’s body inclusions were labelled with all 14 phospho-Tau antibodies, with labelling of globose neurons also observed for several antibodies, e.g. AT100, pThr231, E178, pSer404 (Supp. Fig. S36-S38).

To assess selectivity, 10 of the 16 phospho-antibodies were further tested in brain sections from wildtype, *Mapt*^−/−^ and hTau mice (Supp. Fig. S39; Supp. Table S3). Taken together with IHC data from rTg4510 mouse and human tauopathy brains, lack of detectable signal in *Mapt*^−/−^ brain sections demonstrated selectivity to phosphorylated Tau for all antibodies, with the notable exception of the pSer199 antibody, which cross-reacted with a nuclear phospho-epitope present in CA1 pyramidal neurons, the levels of which appeared to increase in *Mapt*^−/−^ neurons compared to wildtype neurons (Supp. Fig. S39c-c”). Taking our WB data (Supp. Fig. S22h-h’) and previous literature [43, 92, 94, 98] into consideration, one possibility is that the nuclear pSer199 signal in *Mapt*^*−/−*^ and hTau pyramidal neurons represents phosphorylated MAP2, but further testing is required to address this hypothesis.

### Performance of other PTM-dependent Tau antibodies by IHC-IF

A dephosphorylation-dependent antibody, Tau-1 displayed immunoreactivity to neuronal cell bodies in rTg4510 mouse and human AD brain sections following dephosphorylation with λPP, but not in control sections (Supp. Fig. S40a-a’; Supp. Fig. S30e-e’; Supp. Table S3). Tau-1 also provided weak but selective labelling of physiological Tau expressed in wildtype and hTau mouse brains (Supp. Fig. S40h-h”; Supp. Table S3).

Given the accumulation of overt NFT-like pathology in rTg4510 mouse brains by 9 months (the age of animals employed in this study) and the fact that Tau caspase cleavage is an event linked with Tau pathology, D421 epitopes were anticipated to be present at high levels in brain sections from both rTg4510 mice and human tauopathy donors. Labelling rTg4510 mouse brain sections with the D421 antibody, however, produced only weak immunoreactivity (Supp. Fig. S40b; Supp. Table S3), which unexpectedly doubled in intensity following λPP treatment (Supp. Fig. S40b’; Supp. Fig. S41; Supp. Table S3). In contrast, strong labelling of Tau lesions was observed with D421 in PSP and PiD human brain samples, with weaker but clear labelling also present in the AD brain (Supp. Figure 42a-a’’), thus demonstrating that D421 reacts with pathological Tau aggregates in all three diseases. Taken together with our WB findings (Supp. Fig. S15b, column V; Supp. Table S2a), these data suggest that, depending on the origin of and Tau species present in the samples of interest, the Tau phosphorylation status can impact on D421 antibody binding, either directly or through affecting protein conformation. However, given our observation that D421 also detects full-length Tau on WB (Supp. Fig. S15b, column IV; Supp. Table S2a), whether D421-immunoreactivity in biological samples can be used as a reliable readout of Tau cleaved at Asp421 requires further investigation.

### Performance of isoform-specific Tau antibodies by IHC-IF

Knowledge of differential expression of Tau isoforms across the different mouse model brain samples enabled testing of isoform-specific antibodies: (i) rTg4510 mice overexpress the 0N4R human Tau isoform, (ii) 0N4R is the predominant endogenous Tau isoform expressed in adult wildtype mouse brains [103]; and (iii) brains of hTau animals express both 3R and 4R Tau isoforms, with 3R Tau predominating [153]. In line with this, Tau-overexpressing cells in the rTg4510 cortex immunoreacted strongly with the 0N antibody (Supp. Fig. S40c-c’; Supp. Table S3), whereas no signal was detected with the 1N + 2N Tau (#ab109392) antibody (Supp. Fig. S40d-d’; Supp. Table S3), thus supporting the latter reagent’s selectivity but offering no information with regard to its suitability for use in IHC applications.

The ability of the Millipore-RD4 and RD3 antibodies to detect endogenous 4R and 3R Tau isoforms, respectively, was confirmed through their differential immunolabelling of wildtype and hTau mouse brain sections, while absence of immunoreactivity in *Mapt*^−/−^ brain sections confirmed their selectivity (Supp. Fig. S40i-j’’; Supp. Table S3). Unexpectedly, however, the 4R Tau antibody (Millipore-RD4) failed to label Tau-overexpressing cells in the rTg4510 cortex, in both untreated and λPP-treated sections, with immunoreactivity observed instead in neuronal projections and only a small number of cell bodies (Supp. Fig. S40f-f’; Supp. Table S3). In line with conclusions from our WB data that the “Ser622” antibody detects 4R Tau with much higher affinity than 3R Tau (Supp. Fig. S19e-h; Supp. Table S2), its immunoreactivity pattern in rTg4510 mouse brain sections was similar to that obtained with the RD4 antibody (Supp. Fig. S40g-g’; Supp. Table S3). In further agreement with our WB observations, this antibody was found to lack selectivity, as it yielded striking labelling of the apical dendrites of CA1 pyramidal neurons, which persisted in *Mapt*^−/−^ brain (Supp. Fig. S40k-k”; Supp. Table S3). Given that the MTBRs are central to PHF formation [52, 55, 95, 99] and that the majority of Tau-overexpressing cells in rTg4510 brains contain ThS-positive, PHF-like aggregates (Supp. Fig. S34) [56, 80, 125], the pattern of labelling obtained with RD4 and “Ser622″ in these samples may imply that MTBR epitopes are inaccessible in aggregated Tau. Alternatively, it is possible that local conformation or PTM’s overlapping their epitopes may inhibit antibody binding.

Labelling FFPE human AD, PSP, and PiD brain sections using our standard IHC-IF protocol with the RD4 and RD3 antibodies did not yield a detectable signal with either reagent in any of the three tauopathies (Supp. Fig. S42b-c’’). Due to the extensive fixation and further processing that FFPE tissues undergo, epitopes are often masked, thus making the outcome of IHC labelling dependent on the effective retrieval/unmasking of the target epitope. Side-by-side comparison of five antigen retrieval protocols prior to labelling FFPE AD (Braak VI) human brain sections with the pSer214, pSer202 + pThr205, D421 and RD4 antibodies revealed that, while the antigen retrieval protocol influenced labelling outcome, labelling with pSer214, pSer202 + pThr205 and D421 was achieved successfully with several different protocols (Supp. Fig. S43 a-a’’, b-b’’, c–c’’, d-d’’, e-e’’). Of note, proteolytic-induced epitope retrieval (PIER) enhanced pSer214 labelling intensity (Supp. Fig. S43e), but abrogated labelling with the pSer202 + pThr205 and D421 antibodies (Supp. Fig. S43e’-e’’), further highlighting the importance of optimal antigen retrieval. In contrast, no RD4 labelling was observed with any of the five antigen retrieval methods tested (Supp. Fig. S43 a’’’, b’’’, c’’’, d’’’, e’’’).

Although RD3 and RD4 were originally reported to label human brain sections efficiently following a standard antigen retrieval protocol (heat-induced epitope retrieval (HIER) in citrate buffer pH 6.0) [134], very few subsequent studies appear to have obtained immunolabelling with this protocol, e.g. [61], and others have specified that neither antibody labels human tauopathy brain sections in the absence of pre-treatment with formic acid and autoclaving [42]. A literature survey of studies reporting the successful use of RD3 and RD4 in human brain by FFPE-IHC revealed that most had in common the inclusion of formic acid treatment as part of the antigen retrieval protocol [42, 48, 78, 139, 147, 166], although an antigen retrieval approach that combines PIER followed by HIER was also recently reported [40]. Therefore, we next combined a previously-reported optimised RD4 antigen retrieval protocol (HIER citrate pH 6.0 followed by treatment with 99% formic acid) with ultra-sensitive ABC-DAB chromogenic detection. This protocol yielded strong immunolabelling with the Tau-12 antibody (Supp. Fig. S44a), but no detectable signal for the RD3 and RD4 antibodies in FFPE human AD brain sections (Supp. Fig. S44b-c). These findings suggest that the immunoreactivity of RD3 and RD4 antibodies in FFPE-IHC of human brain tissue is highly sensitive to experimental conditions and requires further optimisation. Indeed, we note that a multi-step pre-treatment was required in some studies for enhanced IHC labelling with RD3 and RD4 [48, 72, 149]. Furthermore, Tau deamidation at Asp279 leading to Asn279, an irreversible modification characteristic of AD, abrogates RD4 immunoreactivity and has been reported to be responsible for weak immunoreactivity of the RD4 antibody in human AD brain [42]. Indeed, residual RD4 labelling in the AD brain has been attributed to the presence of a pool of unmodified Tau rather than cross-reactivity of RD4 with the Asn279-Tau [42]. It remains unclear whether other PTMs that overlap the RD4 epitope—such as acetylation at the immediately adjacent Lys280 residue found in AD, PSP, PiD and other tauopathies [75, 76, 106]—may also affect RD4 binding. Potential explanations for the absence of RD3 and RD4 immunolabelling in the samples employed here include technical differences in either the immunolabelling protocol or during the tissue collection, fixation or preservation protocols compared to samples used in previous studies, or underlying biological differences between the samples owing to difference in the Tau PTM profile, Tau conformations or types of aggregates present in different brain regions, at different disease stages and across different donors.

## Discussion

Here, we have used a broad range of sample types and experimental approaches to analyse the performance and establish the utility of Tau antibodies in WB (79 reagents) and IHC-IF (35 reagents) applications. Overall, the number of studies citing use of each antibody clone did not correlate with antibody performance. Indeed, non-selective cross-reactivity with proteins other than Tau was observed for three of the four most highly-cited “total” Tau antibodies, namely HT7 (436 citations), K9JA polyclonal Tau antibody from Dako (354 citations), and Tau-46 (300 citations) [37]. Moreover, binding of the most highly-cited “total” Tau antibody, the Tau-5 clone (703 citations), was found to be partially inhibited by phosphorylation. In contrast, the best-performing antibody clones identified in the course of this work have been used in relatively few studies to date, e.g., Tau-12 (40 citations), Tau-13 (54 citations). This may be explained by historical biases and suggests that use of sub-optimally performing antibodies has been propagated in research studies, likely because of the positive feedback loop created when choice of reagents is driven by the number of citations.

### Antibody performance: ability to detect the target protein and selectivity

Most of the antibodies tested were able to detect Tau when the target is present at high levels, as is the case for overexpression models. We further demonstrate that most of the antibodies also immunoreacted with human brain Tau from patients diagnosed with different tauopathies, both in WB and by IHC-IF on FFPE brain sections. Among these antibodies, however, many failed to detect the protein at lower levels, including widely utilised antibodies such as Tau-5, HT7 and Tau-46, highlighting that choice of primary antibody is critical for the reliable detection of Tau in low-expressing samples, particularly in non-neuronal cells.

In both WB and IHC, only a small proportion of the antibodies tested failed to detect the target Tau species in any of the samples tested. By WB, this was the case for antibodies targeting certain pathological Tau modifications, namely: Tau phosphorylated at Ser238, Tau acetylated at Lys174 and oligomeric Tau (T22 and TOMA-1 antibodies). As the target epitopes for these antibodies have all been reported to be present in AD brain samples, absence of immunoreactivity with AD brain Tau for these reagents may suggest that: (i) the antibody does not detect the target Tau species by WB, under the experimental conditions employed here; or, alternatively, (ii) that the target epitopes were present at low levels below the detection limit of the techniques employed here. In FFPE-IHC, all antibodies labelled Tau in either mouse or human brain sections, but we found that: (i) the 77E9 antibody labelled Tau only in human brain sections and only following dephosphorylation with λPP; while (ii) the RD3 and RD4 antibodies against 3R and 4R Tau isoforms, respectively, labelled physiological Tau in mouse brain sections, but failed to label Tau in human tauopathy brain sections. Performing antigen retrieval using different protocols, including protocols reported to have been optimised for labelling with RD3 and RD4 by FFPE-IHC, did not yield detectable labelling of AD brain Tau with either antibody. Our findings suggest that FFPE-IHC protocols for RD3 and RD4 labelling may require further optimisation to ensure that these reagents can be used reproducibly across different laboratories and on clinical samples from different sources.

Unexpectedly, WB immunoreactivity for 58% (14/24) of the presumed PTM-agnostic “total” Tau antibodies was impacted by phosphorylation, an observation that may be explained either by a direct inhibitor effect of phosphorylation on antibody binding or through an indirect effect of phosphorylation on Tau conformation. In two instances (77E9 and A16097E clones), the impact of protein dephosphorylation on antibody WB performance was even greater than that observed for the Tau-1 clone, an antibody that is well established to bind its mid-Tau epitope only when this is dephosphorylated [17, 111]. A better understanding of these findings was obtained through comparing the epitope regions of 40 of the PTM-agnostic Tau antibodies included in our panel with the phospho-residues present in the human 2N4R Tau sequence, as well as the phosphorylation profiles of human brain Tau, SH-SY5Y Tau and recombinant pTau. This revealed a well-known concentration of phosphorylated residues in the mid-domain and C-term regions of the protein, and established the identity and number of potential phospho-sites overlapping each antibody epitope (Supp. Fig. S45). This analysis further highlights that many of the antibody epitopes overlap or are in very close proximity to sites of increased phosphorylation in the human AD brain (Supp. Fig. S45). Of note, three Ser residues fall within the 77E9 epitope with several other phosho-sites in close proximity, while the BT2 epitope (despite being only five amino acids long), overlaps three phospho-sites, coinciding partly with the Tau-1 epitope (Supp. Fig. S45b). Surprisingly, the epitope of the HT7 clone, which is predicted to fall within that of the D5D8N clone, does not overlap any Ser, Thr or Tyr residues (Supp. Fig. S45b). However, in our experimental data, HT7 immunoreactivity increased following dephosphorylation of the samples, whereas that of the D5D8N clone was unaffected. Such discordant data may suggest that our current understanding of the HT7 and/or D5D8N epitope locations is incomplete. Alternatively, it is possible that the HT7, but not D5D8N, binding affinity is sensitive to protein conformation. Taken together, these data have far-reaching implications for one of the most common uses of Tau antibodies in biomedical research: assessing the levels of phospho-Tau relative to “total” Tau in biological samples. Overall, our findings suggest that, for many antibodies, detection and quantification of “total” Tau can be achieved most accurately if performed on phosphatase-treated samples.

By WB, non-selective cross-reactivity was apparent for many of the antibodies tested, with 62% (42/68) found to react with proteins other than Tau under the conditions tested. In contrast, only 8% (3/38) of the antibodies tested by IHC were judged as non-selective. This disparity is likely multifactorial. Firstly, some of the reagents that were found to perform poorly by WB were not included in the panel for IHC testing. Furthermore, it is possible that some antibodies cross-react with epitopes that become exposed by denaturing proteins during SDS-PAGE sample preparation, but that are not accessible in proteins observed in situ. Finally, unlike the IHC studies, our WB testing included samples that expressed Tau at very low levels, leading to a higher proportion of proteins that are potential sources of non-selective binding relative to target epitopes, thus increasing the likelihood of detecting non-selective reactivity. The latter principle is exemplified by the complementary observation that almost all antibodies appeared highly selective for Tau when tested on samples where the protein was overexpressed, thus highlighting the potential pitfalls associated with the use of overexpression systems to monitor antibody selectivity [47, 88]. Considerations of selectivity, however, should account for the fact that it is not an absolute feature: instead, antibody selectivity must be defined relative to the sample type and with regard to the experimental labelling protocol, particularly with respect to any blocking steps and the choice of detection reagents. For example, Tau-12 and AT8 have previously been reported to display non-selective binding in mouse brain samples [115]. However, both reagents displayed high selectivity under the experimental conditions employed here.

Proteins belonging to the same family may share sequence similarities, giving rise to the possibility that some antibodies may detect several related proteins, depending on their target epitopes. In the case of Tau, its C-terminal half shares extensive sequence homology with that of MAP2 and, to a lesser extent, MAP4 [45], cautioning that some Tau antibodies may cross-react with either or both proteins. Indeed, we demonstrate that several “total” Tau (Tau-46, K9JA, A16097E and A16097B) and, unexpectedly, phospho-Tau antibodies (pSer199, pSer202 + pThr205 and pSer396) cross-react with MAP2. This is of particular concern for any applications where the MW of the protein being detected cannot be ascertained (e.g. IHC, ELISA, co-IP, dot blot). However, it also needs to be accounted for in WB analyses of tissues or cell types that express 2N Tau isoforms, which migrate on SDS-PAGE at a similar apparent MW to MAP2c, thus precluding the clear discrimination between MAP2 and Tau signals.

The T22 antibody clone stood out as a particular case of poor selectivity towards the intended target proteoform, with our WB data revealing that it also binds monomeric Tau, thus demonstrating that T22 reactivity is not restricted to oligomeric Tau, as previously thought. Indeed, binding to monomeric Tau can be observed in previously published data, but has remained largely unacknowledged ([97], Fig. 4F; [13], Fig. 7; [157], Fig. 3; [51], Fig. 3d—see “Tau 0 h” on dot blot; [28], Fig. 4A; [145], Fig. 4C; [18], Fig. 2; [41], Fig. S15; [67], Fig. 4A-C; [77], Fig. 4A; [83], Fig. 3C; [74], Fig. 4A). We further found that a “purified” commercial version of this reagent, which has not been employed widely in the scientific literature to date, did not react with monomeric Tau but neither did it detect oligomeric Tau in our testing. We note that T22 reactivity with monomeric Tau is reminiscent of similar findings previously reported for another presumed “oligomer specific” Tau antibody, the TOC1 antibody clone, which was also found to bind monomeric Tau in denatured samples on WB [158]. Both of these antibody clones have been reported to detect Tau oligomers only under specific non-denaturing conditions, a reactivity pattern that is not dissimilar to that of some “total” Tau antibodies. It remains possible, as has been proposed for TOC1 [158], that such antibodies could detect a linear epitope on Tau that is not exposed in native monomeric Tau, becomes exposed in Tau dimers/trimers due to conformational changes, and becomes inaccessible again in higher order oligomers and mature Tau aggregates. Such a scenario could explain preferential binding to oligomers in samples where Tau retains its native conformation, but detection of Tau monomers under denaturing conditions—note that it remains unclear which category the use of T22 in IHC protocols that incorporate antigen retrieval steps would fall under. However, it is important to note that the experimental conditions originally used to characterise the two oligomeric Tau antibody clones tested here, T22 and TOMA-1 [29, 87], represent neither native WB conditions (SDS was used), nor standard denaturing WB conditions (the effect of denaturation was only reported for “extreme denaturing conditions” in 8 M urea, a very harsh denaturing agent). A better understanding of the characteristics and performance of oligomeric Tau antibodies is needed. In the meantime, these observations caution that findings obtained with the T22 antibody clone should be interpreted carefully, with particular attention to the exact reagent (“purified” or “original” version) and experimental protocol used in each study (in particular sample preparation steps), and highlight the need for orthogonal validation, especially for applications where the MW of the protein(s) being detected is not known (e.g. dot blot, co-IP, IHC, ELISA).

For PTM-dependent antibodies, non-selective reactivity may also arise from binding to the unmodified target protein, with several polyclonal phospho-Tau antibodies previously reported to detect the unphosphorylated form of the protein [50]. In contrast, we found that 96% (23/24; 21 monoclonals) of the phospho-Tau antibodies tested by WB were indeed phosphorylation-specific, as demonstrated by their lack of reactivity with the recombinant Tau ladder or against dephosphorylated samples. In apparent contradiction, however, we also found that IHC immunolabelling of pathological Tau aggregates with some phospho-Tau antibodies was unaltered (pSer199 antibody), or even enhanced (E178 and pSer396 antibodies), following λPP treatment. To explain this perplexing observation, we advance the hypothesis that the enhanced IHC immunoreactivity observed with some phospho-Tau antibodies following dephosphorylation results from: (1) phosphorylation at the relevant epitopes (e.g. phospho-Ser199 and phospho-Ser396) being resistant to λPP in aggregated/PHF-Tau, combined with (2) removal of potential inhibitory phosphorylation events at nearby residues, which may improve epitope accessibility. In support of this hypothesis, a previous study found that the binding of a different pSer396 antibody to a peptide carrying its target epitope was hindered by modifications at nearby residues [50].

### Non-canonical and cleaved Tau isoforms

Here, testing of a large panel of antibodies allowed the identification of “non-canonical” bands that may otherwise have been dismissed as non-specific signals or been attributed to poor sample preservation. Several cleavage events leading to low-MW truncated Tau variants have been reported, each with different characteristics in terms of their microtubule-binding affinity, propensity to aggregate, seeding activity, secretion or enrichment in biological fluids, particularly in the CSF [15, 16, 23, 35, 60, 113, 119]. These have further attracted widespread interest as potential biomarkers, as well as for their purported role in spreading Tau pathology between neuroanatomically connected areas of the brain, but have been studied mostly in the context of pathological conditions. Here, employing a panel of antibodies with epitopes spanning the entire length of the protein enabled us to establish that the human brain Tau pool consists of a mixture of full-length Tau and truncated Tau variants. In the latter category, a prominent fraction of C-term-truncated variants were detected in phosphatase-treated brain extracts as a distinct pattern of four closely spaced bands. As these were observed consistently across control and tauopathy donors, we propose that they represent physiological proteoforms. Strikingly, C-term-truncated Tau variants mirrored the full-length Tau pool in terms of their SDS-PAGE migration pattern and isoform composition, suggesting that they arise from a consistent cleavage/truncation event that affects all full-length isoforms equally. Recently, C-term-truncated Tau fragments were reported in five different brain regions from non-demented control donors of ages 18 to 104 [57], but the technical design of the study did not enable further characterisation of their nature. In contrast to this previous report, our data do not support the existence of the smaller 17–25 kDa C-term-fragments in the brains of control donors, at least not at levels detectable by WB. We note that these previous findings relied on detection with a small number of antibodies (three), of which only two (N-term and mid-domain) would detect the C-term-truncated variants, where: (i) the N-terminal antibody (Abcam, cat. no. ab27162) is recommended by the manufacturer for flow cytometry and ELISA, but has not been formally tested in denaturing WB applications; and (ii) the C-terminal antibody was a goat polyclonal antibody (Santa Cruz, cat. no. sc-1995) of uncharacterised performance that has since been discontinued.

Importantly, the truncated Tau variants identified here are not the same as the C-term-truncated Tau species that have been reported to dominate the human CSF Tau pool [128]. The latter are reportedly truncated at the end of the Proline-rich mid-domain, between amino acids 221–225 [128]. In contrast, as demonstrated by immunoreactivity with 3R and 4R Tau antibodies, the C-term-truncated Tau variants characterised here incorporate sequences up to at least the second MTBR encoded by exon 10, demonstrating that they comprise the Tau sequence up to at least residue 305. While the precise truncation site requires future investigation, detection with 3R and 4R Tau antibodies, but absence of immunoreactivity with A16097B (epitope region: 359–373) and A-10 (epitope region: 399–418), suggest that it likely lies between amino acids 305 to 360 of the human 2N4R Tau sequence. A recently reported truncated Tau variant, Tau-11i, arises through retention of intron 11 and lacks amino acids 333 to 441 [110]. However, we note that—even in total AD brain lysates that have not been subjected to dephosphorylation—Tau-11i was detected as a single, clearly-defined band of ~ 50 kDa [110], both features that distinguish it from the C-term-truncated Tau variants described here.

Caspase-2 cleaves Tau at Asp314 giving rise to Δtau314, the soluble C-term-truncated cleavage products, which are found in the normal human brain and have been found to be elevated in the brains of cognitively impaired individuals [93]. Similar to the C-term-truncated variants that we have identified in human brain extracts by WB, Δtau314 proteoforms derived from all six brain Tau isoforms have been observed [93]. In future, it would be informative to determine whether the C-term-truncated Tau bands described here correspond to Δtau314 cleavage products. Given the complexities and cell type heterogeneity of the human brain, investigating the spatial distribution of the different cleaved Tau variants could offer insights into their origin, localisation and biological functions. In future studies, the information presented here can inform the design of a panel of reliable antibodies, with non-overlapping epitopes, to enable double IF labelling using pairs of N-term/C-term, N-term/mid-term and mid-term/C-term antibodies. Combining such an approach with carefully calibrated quantification protocols could be used to establish whether the different Tau fragments are enriched in or exclusive to specific cell types or brain regions, and whether these patterns are altered in disease.

Tau isoforms of MWs lower than full-length Tau may also arise through unconventional mechanisms, e.g. exon skipping. Indeed, we demonstrate that presumed knockout, *MAPT*-edited HAP1 cell lines express residual Tau protein generated through skipping of the deletion-carrying exon, which give rise to novel low-MW Tau isoforms that are not truncated at either terminus. This finding has broad relevance for attempts to genetically engineer the *MAPT* locus in human cells, as we have recently found that neural progenitor cells derived from presumed “Tau knockout” human induced pluripotent stem cells (hiPSCs), that carry CRISPR–Cas9-induced deletions in *MAPT* exons 1 or 4, also continue to express low levels of residual Tau protein, similar to our findings in HAP1 edited cells [108]. A 26–30 kDa small Tau isoform that may arise via exon skipping was previously described in SH-SY5Y cells [131], and experimental evidence has demonstrated that “constitutively included” *MAPT* exons can be skipped to generate a gene product that integrates both termini [140]. Although residual Tau expression in presumed knockout cells may be surprising, it is worth noting that two independent previous studies utilising different panels of presumed “knockout” CRISPR-Cas9-edited HAP1 cell lines found that ~ 30% of these continued to express the target protein, often in the form of novel variants induced by skipping of the targeted exon [136, 148]. Indeed, gene plasticity, evidenced as skipping of otherwise constitutively included exons, is a widespread phenomenon induced by CRISPR–Cas9 genomic editing reported to occur in a wide variety of cell lines as well as mammalian, insect and plant models [14, 32, 73, 130, 141].

## Limitations

Due to the number of reagents tested, and to enable side-by-side comparisons, we used a standardised immunodetection protocol for most antibodies. Alternative protocols were tested for a small number of reagents, to test the impact of altered experimental conditions on the antibody performance. It is possible that further optimisation of factors, such as antigen retrieval, blocking, detergent concentration and antibody incubation conditions, may improve the performance of some antibodies. Moreover, we did not seek to confirm the identity of each epitope by unequivocal empirical analysis. To mitigate this, we used monoclonal antibodies wherever possible, many of which were raised against well-defined synthetic peptides. For IHC applications, we focused on FFPE tissues, owing to their widespread use and availability. Similar approaches could be employed to validate Tau antibodies for use in IHC on frozen tissue sections. To extend the findings presented here, future studies should also aim to assess the presence of different Tau species, including truncated Tau variants, in a larger number of donors.

## Conclusions

Here, we have established a resource designed to guide informed antibody choice for the study of Tau in protein extracts by WB or in situ by IHC. Our work highlights the importance of preliminary antibody validation performed in complex biological samples representative of the samples of interest in terms of target protein expression levels and species of origin, two factors that can profoundly affect antibody performance. This is especially pertinent given our observations that: (a) many antibodies show non-selective cross-reactivity with proteins other than Tau (the levels of which may vary depending on sample type); (b) not all antibodies are suitable for detecting Tau expressed at low or physiological levels; (c) the binding of many presumed PTM-agnostic antibodies is affected by Tau phosphorylation status; and (d) outcomes obtained with supposedly identical antibody clones can vary depending on the commercial supplier.

Despite the biological and pathological relevance of Tau splice isoforms, our work highlights the unmet need for antibodies that detect the different isoforms, including Big Tau, with high selectivity and sensitivity. Conversely, to the best of our knowledge, no truly “total Tau” antibody exists as yet, since no single antibody can detect all products of proteolytic cleavage. A polyclonal antibody raised against Big/PNS-Tau may achieve this, but its utility would likely be compromised by low selectivity. Currently, if the goal is to detect most Tau species, including cleaved variants, an optimal strategy might employ a combined panel of N-terminal, mid-domain and C-terminal antibodies, with special consideration for C-term antibodies that may cross-react with MAP2 and, potentially, MAP4. Moreover, in light of our findings that phosphorylation can inhibit the binding of some “total” Tau antibodies, dephosphorylation of the samples prior to analysis may be desirable, although we cannot rule out the existence of further PTMs that may also influence binding.

Ultimately, in this study, we identify a panel of high-fidelity Tau antibodies that can be used to reliably detect a wide range of Tau proteoforms in biological samples with high selectivity, not only in neuronal cells but also in tissues or cell types where the protein is expressed at endogenous (often very low) levels. In doing so, this work opens the door to studying this complex protein in non-neuronal cell types and peripheral tissues where its expression and functions remain largely unexplored. For the reliable identification and characterisation of truncated Tau variants in biological studies we propose that multiple antibodies with non-overlapping epitopes should ideally be employed. The detailed understanding of the performance of Tau antibody reagents presented here will improve interpretation of research data, enhance reproducibility of future Tau research studies, and guide the choice of appropriate antibodies for different research questions and sample types, ultimately enabling faster progress towards understanding the many neurodegenerative diseases where the protein has been implicated.

## Additional files

**Supplementary Table** **S1****. List of all antibodies used in this study**. Categories shown for all antibodies are as follows: column **a**, antibody name and clone name (where applicable, shown in square brackets after the antibody name); column** b**, host species; column** c**, clonality; column** d**, isotype; column** e**, commercial supplier; column** f**, catalog number; column** g**, Research Resource Identifier (RRID); column** h**, number of studies citing each antibody according to Citeab; column** i**, antibody concentration as reported by the supplier; column** j**, dilution used for WB applications; column** k**, dilution used for IHC-IF applications. In addition, where applicable and where this information is available, either from the manufacturer or in previous studies, the following information was also included: column** l**, immunogen; column** m**, targeted epitope; column** n**, species reactivity.

**Supplementary Table** **S2****. A-B:** Detailed analysis of antibody performance by WB for “total” Tau antibodies, PTM-dependent antibodies and isoform-specific antibodies. Note that information for Big Tau antibodies can be found in **part B** (separate sheet).

**Supplementary Table** **S3****.** Analysis of antibody performance by IHC-IF.

**Supplementary Table** **S4****.** Comparison of the phospho-Tau antibodies that detected recombinant GSK3beta, DYRK1A and CAMKIIA-phosphorylated Tau with the target sites that each respective kinase has been reported to phosphorylate in recombinant Tau. Data for GSK3beta target sites in recombinant Tau was compiled from [30, 63, 121, 152, 154], data for DYRK1A target sites in recombinant Tau was compiled from [91, 124], and data for CAMKIIA target sites in recombinant Tau was compiled from [30, 89, 152, 154, 167]. As Tau phosphorylation by various kinases is highly interdependent, with some phosphorylation events by one kinase priming further phosphorylation events by a different kinase, only reports that tested phosphorylation of recombinant Tau with the respective kinase individually were considered, given that the recombinant pTau proteins used in this study were phosphorylated with only one kinase at a time.

### Supplementary Information

Below is the link to the electronic supplementary material.

Supplementary file1 (PDF 53390 KB)

Supplementary file2 (XLSX 289 KB)

## Data Availability

All data generated or analyzed during this study are included in this published article.

## Declarations

### Availability of data and materials

All antibodies mentioned in this manuscript are commercially available. The mass spectrometry proteomics data have been deposited to the ProteomeXchange Consortium via the PRoteomics IDEntification Database (PRIDE) [114] partner repository with the dataset identifier PXD041199. All other data on which conclusions are based are included within the manuscript and its additional files, and raw data files are available from the corresponding authors upon reasonable request.
