# Supplementary material for: Identification of high-performing antibodies for the reliable detection of Tau proteoforms by Western blotting and immunohistochemistry
Source: Acta Neuropathol. 2024 May 18;147(1):87. doi: 10.1007/s00401-024-02729-7 (PMC11102361; doi:10.1007/s00401-024-02729-7)
Supplement: Supplementary file 1 — Supplementary file1 (PDF 53390 KB) [file 401_2024_2729_MOESM1_ESM.pdf]

Identification of High-Performing Antibodies for the Reliable Detection of Tau Proteoforms by Western Blotting and Immunohistochemistry

Michael J. Ellis, Christiana Lekka, Katie L. Holden, Hanna Tulmin, Faheem Seedat, Darragh P. O'Brien, Shalinee Dhayal, Marie-Louise Zeissler, Jakob G. Knudsen, Benedikt M. Kessler, Noel G. Morgan, John A. Todd, Sarah J. Richardson, M. Irina Stefana

Supplementary Materials and Methods

Cell culture

Human embryonic kidney (HEK) 293T cells (RRID: CVCL_0063) were maintained in Dulbecco's Modified Eagle's Medium (DMEM, Sigma, cat. no. D6429) supplemented with 10% heat-inactivated foetal bovine serum (hiFBS, Sigma, cat. no. F9665) at 37°C under a 5% CO_2 humidified atmosphere. No antibiotics were used. Cells were split upon reaching 90-95% confluence (approximately every 2-3 days). HEK293T Lenti-X cells (Takara Bio, cat. no. 632180; RRID: CVCL_4401) were maintained in DMEM supplemented with 10% hiFBS, 1x GlutaMAX (Gibco, cat. no. 13462629) and 1 mM sodium pyruvate (Gibco, cat. no. 11360070), at 37°C under a 5% CO_2 humidified atmosphere. No antibiotics were used. Cells were split upon reaching 90-95% confluence (approximately every 2-3 days). SH-SY5Y cells (European Collection of Authenticated Cell Cultures via Sigma-Merck, cat. no. 94030304; RRID: CVCL_0019) were maintained in DMEM:F12 (1:1) medium (Gibco, cat. no. 11330057) supplemented with 10% hiFBS (Sigma, cat. no. F9665) at 37°C under a 5% CO_2 humidified atmosphere. No antibiotics were used. Cells were split upon reaching ~90% confluency (approx. every 5 days) at a ratio of 1:5. Cells were collected for experiments when 85-90% confluent. To inhibit protein dephosphorylation and promote the accumulation of phosphorylated Tau, SH-SY5Y cells were treated with 0.5µM okadaic acid, or DMSO carrier as control, for 2 hours. Parental control and *MAPT*-edited HAP1 cells were purchased from Horizon Discovery (parental control cells cat. no. C631, RRID: CVCL_Y019; 2 bp deletion cells cat. no. HZGHC003277c009, RRID:CVCL_SX09; 14 bp deletion cells cat. no. HZGHC003277c003, RRID:CVCL_SX08). The two *MAPT*-edited cell lines were generated by CRISPR/Cas9 editing using the same guide RNA (sequence: GACCAGCAGCTTCGTCTTCC) and carried either a 2 bp or a 14 bp deletion in *MAPT* exon 4 (exon numbering as shown in **Fig. 1**), in both cases leading to out-of-frame shifts and the introduction of early stop codons. Following the supplier's instructions, cells were maintained in Iscove's Modified Dulbecco's Medium (Sigma, cat. no. I3390)

supplemented with 10% hiFBS and 1% Pen/Strep at 37°C under a 5% CO_2 humidified atmosphere. Cells were split upon reaching 70-75% confluency (approximately every 2-3 days). Cells were collected for experiments when ~70% confluent.

Tau overexpression in HEK293T cells

HEK293T cells were transfected, either with constructs encoding human 0N3R Tau or with control constructs, using Fugene 6 (Promega, cat. no. E2691) according to the manufacturer's instructions. Transfection rates of >90% and high expression of the transgenic construct were confirmed under the fluorescence microscope 48 hours post-transfection. Cells were lysed (as described below) for protein extraction 72 hours post-transfection. Plasmids used for tdTomato-Tau overexpression and the tdTomato-C1 control plasmid were a gift from Michael Davidson: tdTomato-MAPTau-C-10 (RRID: Addgene_58112), tdTomato-MAPTau-N-10 (RRID:Addgene_58113) and tdTomato-C1 (RRID:Addgene_54653). Control tdTomato-N1 plasmid was a gift from Michael Davidson, Nathan Shaner and Roger Tsien (RRID:Addgene_54642) [100].

Expression of transgenic Tau in SH-SY5Y cells

The cDNA sequence of either the 441 amino acids-long human 2N4R Tau isoform or that of the 758 amino acids-long human Big Tau isoform, respectively, was cloned into the pSMPUW-IRES-Bsd vector backbone (Cell Biolabs, cat. no. VPK-219). The empty vector was used as a control. Lentiviral particles were generated by co-transfecting HEK293T Lenti-X cells with packaging vectors – pRSV-Rev, pCMV-VSV-G and pCgpV (Cell Biolabs, ViraSafe Lentiviral Packaging System, cat. no. VPK-206) – and either the empty, the 2N4R-encoding, or the Big Tau-encoding pSMPUW-IRES-Bsd transfer vector, using the PEIPro DNA transfection reagent (Polyplus, cat. no. 115-010) following manufacturer's instructions. The molar ratio of transfer vector to packaging vectors was 3:1:1:1. Media containing lentiviral particles was collected 72 hours post-transfection and the lentiviral particles were precipitated with 3 volumes of Lenti-X Concentrator (Takara, cat. no. 631232) at 4°C overnight, following manufacturer's instructions. The lentiviral stock was aliquoted in single-use aliquots and stored at -80°C. To generate SH-SY5Y cells that stably expressed transgenic Tau, lentiviral stock (either empty or Tau-encoding lentiviral particles) was added to cells in suspension, prior to seeding cells into flasks. Following 20 hours of transduction, the virus was removed, cells were split into fresh flasks and allowed to recover for 24

hours. Transduced cells were then selected with blasticidin (7 µg/mL) for 6 days, until all untransduced, positive-control cells had died. Fresh antibiotic was provided every 2 days during the selection protocol.

Recombinant proteins

An equimolar mixture of recombinant versions of the six common Tau splice isoforms (0N3R, 0N4R, 1N3R, 1N4R, 2N3R and 2N4R), referred to as the Tau ladder (either Sigma cat. no. T7951, or Signal Chem cat. no. T08-07N-250), was used as a control for antibody validation by WB. Note that the Signal Chem Tau ladder contained a seventh Tau isoform of a MW ~10 kDa lower than 0N3R, the smallest of the six common Tau isoforms. Recombinant 2N4R Tau that had been phosphorylated, *in vivo* and *in vitro*, with specific kinases was also used to validate phosphorylation-dependent antibodies and to test the impact of phosphorylation on binding of total and isoform-specific Tau antibodies: GSK3β-phosphorylated Tau (Signal Chem, cat. no. T08-50FN-20), DYRK1A-phosphorylated Tau (Signal Chem, cat. no. T08-50RN-20) and CAMK2A-phosphorylated Tau (Signal Chem, cat. no. T08-50CN-20). Recombinant MAP2c protein (Abcam, #ab114686), carrying a ~26 kDa Glutathione-S-Transferase (GST) tag at the N-terminus, was used to test for cross-reactivity of Tau antibodies with MAP2. Recombinant proteins were resuspended, if applicable, according to manufacturer's instructions, aliquoted and stored at -80°C until use.

Animals and mouse brain samples for WB and IHC

In this study, we used wildtype, *Mapt* knockout (*Mapt*$^{-/-}$; [35]) and humanised Tau (hTau; [125]) mice, all of which were maintained on a C57BL/6J background. hTau mice were generated using P1-derived artificial chromosome (PAC) technology to express the entire 143 kb wildtype human *MAPT* locus (H1 haplotype) in *Mapt*$^{-/-}$ mice. The 143 kb transgene comprises of 9 kb of promoter sequence and 134 kb genomic DNA, including the two alternative polyadenylation sequences at the 3' end of the human *MAPT* locus [91]. This allows the physiological expression of all human Tau isoforms under the control of the endogenous human *MAPT* promoter. Animals were sacrificed at five months of age by cervical dislocation, brains were dissected quickly, and half of the brain was snap-frozen in liquid nitrogen and stored at −80°C until lysis. The other half of the brain was fixed in formalin and embedded in paraffin blocks. Sagittal sections of 4 µm thickness were used for immunohistochemistry. Tissues from female mice were used in this study. rTg4510 mice [95] used in the study were acquired from Eli Lily through

collaboration with Dr Jonathan Brown (Exeter University, UK). Dissected brains were formalin-fixed and embedded in paraffin blocks. 4 µm coronal sections were cut from the forebrain of 9-month old rTg4510 male mice using a Leica RM2235 microtome. All housing and experimental procedures were carried out in compliance with the local ethical review panel of the Universities of Oxford and Exeter, respectively, under UK Home Office project licenses held in accordance with the Animals (Scientific Procedures) Act 1986. Animals were housed under a 12-hour light/dark cycle with *ad libitum* access to food and water.

Preparation of protein samples and Western blotting

For preparation of cell lysates, cells were washed twice with PBS at room temperature (RT) and lysed in 1x radio immunoprecipitation assay buffer (RIPA buffer, Merck Millipore, cat. no. 20-188) containing 0.1% SDS, 1x cOmplete™ EDTA-free Protease Inhibitor Cocktail (Roche, cat. no. 11873580001) and 1x PhosSTOP™ phosphatase inhibitors (Roche, cat. no. 4906837001). Lysis was allowed to proceed on ice for 15 minutes before cells were scraped from the cell culture flask, transferred to low-protein binding microcentrifuge tubes and lysis was allowed to proceed for a further 15 minutes on ice, for a total of 30 minutes. Lysates were then spun down at 20,000 x g for 20 minutes to pellet genomic DNA and cellular debris, and the supernatant was aliquoted into fresh, low-protein binding tubes. Lysates were stored at -80°C.

To extract proteins from mouse brains, frozen tissues were ground in a mortar on dry ice and the resulting pulverised tissue was transferred to low-protein binding microcentrifuge tubes. The pulverised tissues were then sonicated on ice in lysis buffer (1x RIPA buffer containing 0.1% SDS, cOmplete™ EDTA-free Protease Inhibitor Cocktail and PhosSTOP™ phosphatase inhibitors; 16 µL lysis buffer per mg tissue), samples were spun at 20,000 x g for 20 minutes and the supernatant was aliquoted into fresh, low-protein binding tubes. Lysates were stored at -80°C.

For human brain samples, 1g frozen tissue from each donor was ground in liquid nitrogen using a mortar and pestle. The resulting tissue powder was aliquoted into low-protein binding microcentrifuge tubes on dry ice and stored at -80°C until use. To extract proteins, the pulverised tissue was resuspended in lysis buffer (1x RIPA buffer containing 0.1% SDS, cOmplete™ EDTA-free Protease Inhibitor Cocktail and PhosSTOP™ phosphatase inhibitors; 75 µL lysis buffer per mg tissue), the lysate

was passed through a 25G syringe needle 5-7 times until homogenous and any visible clumps were dispersed, and lysis was allowed to proceed on ice for a total of 40 minutes. Lysates were then spun down at 20,000 x g for 30 minutes to pellet genomic DNA and cellular debris, and the supernatant was aliquoted into fresh, low-protein binding tubes. Lysates were stored at -80°C.

To extract proteins from human brain RIPA pellets (resulting from the protein extraction described in the previous paragraph), pellets were resuspended in lysis buffer and treated with DNase I-XT (New England Biolabs, M0570; 2.5 uL per 100 uL lysis buffer) according to manufacturer's instructions for 45 mins at 37°C. DNase-digested pellet samples were then mixed 3:1 with 4x Laemmli buffer (Biorad, cat. no. 1610747) containing β-mercaptoethanol and heated at 95°C for 10 minutes. Boiled pellet samples were spun down briefly (<30 sec) prior to loading on the gel for WB analysis.

Protein concentration was quantified using the Pierce™ BCA Protein Assay Kit (ThermoFisher Scientific, cat. no. 23227) according to the manufacturer's instructions. To prepare samples for WB, lysates were thawed on ice, mixed 3:1 with 4x Laemmli buffer (Biorad, cat. no. 1610747) containing β-mercaptoethanol and heated at 95°C for 10 minutes. Samples were adjusted to the same protein concentration by adding 1x Laemmli buffer containing β-mercaptoethanol. Sodium dodecyl sulfate-polyacrylamide gel electrophoresis (SDS-PAGE) was performed by loading: 6 µg per lane for HEK293T cell lysates, 5 ng/isoform per lane for recombinant Tau ladder (Sigma, cat. no. T7951) on mouse brain blots, 8 µg per lane for mouse brain extracts, 40 µg per lane for SH-SY5Y and HAP1 cell lysates, 20 µg per lane for human brain RIPA lysates, 15 µg per lane for human brain lysates untreated or treated with lambda phosphatase, 50 ng per lane for recombinant phosphorylated Tau, 50 ng/isoform per lane for the recombinant Tau ladder used as a control on the recombinant phospho-Tau blots, 20 ng recombinant MAP2c (Abcam, cat. no. ab114686). Proteins were separated on 4-15% gradient Mini-PROTEAN TGX Stain Free Gels (Bio-Rad, cat. no. 4568084 for 10-well gels, cat. no. 4561086 for 15-well gels) and transferred to Immobilon-FL PVDF membranes (Merck Millipore, cat. no. IPFL00010). Blots were blocked in 5% Amersham™ ECL Prime Blocking Reagent (SLS, cat. no. RPN418) diluted in TBS (blocking buffer, no detergent). Primary antibodies were diluted in blocking buffer containing 0.1% Tween-20 and membranes were incubated with primary antibodies for either 2 hours at RT or overnight at 4°C. For primary antibody details, see **Supp. Table S1**. Secondary antibodies (for details, see in **Supp. Table S1**) were diluted 1:10,000 in blocking buffer containing 0.1% Tween-20 and 0.01%

SDS. Membranes were incubated with secondary antibodies for 1 hour at RT on an orbital shaker. Blots were washed three times with TBS buffer containing 0.1% Tween-20 after the primary and secondary antibody incubation steps and once with TBS buffer without detergent at the end. Membranes were imaged dry on an Odyssey CLx scanner (LI-COR Biosciences), and blot images were visualised and quantified in the Image Studio software (version 5.2.5; LI-COR Biosciences).

When analysing mouse samples by WB, endogenous IgG detection by anti-mouse secondary antibodies can contribute to non-specific signals. For the analysis of Tau proteins, this can be particularly troublesome as IgG heavy chain signals can overlap with monomeric Tau. In the present study, cross-reactivity with bands at ~26 kDa and/or ~55 kDa, consistent with the MWs of the IgG light and heavy chains, respectively, were observed on WB for some but not all mouse antibodies (exceptions include: Tau-12, Tau-13 and 77E9, presumably due to high signal-to-noise ratios). In contrast with previous reports [91] and except for the D421, 0N (BioLegend) and 2N (BioLegend) antibody clones, we find that, even when present, these bands were only faintly detectable.

Tau immunoprecipitation from HAP1 cells

To immunoprecipitate (IP) endogenous Tau from parental and *MAPT*-edited HAP1 cells, we used cytoplasmic extracts prepared using the cytoplasmic extraction reagents (CER) from the NE-PER™ kit (ThermoFisher Scientific, cat. no. 7883; 11 µL CERI and 0.6 µL CERII were used per 1 x 10^6 cells) according to the manufacturer's instructions. cOmplete™ EDTA-free Protease Inhibitor Cocktail and PhosSTOP™ phosphatase inhibitors were added to the CERI extraction buffer. Protein extracts were transferred to low-protein binding tubes, stored on ice and used immediately to quantify protein concentration using the Pierce™ BCA Protein Assay Kit and set up the IP reactions. Protein lysates (1 mg per IP reaction) were incubated in low-protein binding microcentrifuge tubes with Tau antibodies (pThr231 or SP70 rabbit monoclonal antibodies; 0.8 µg antibody/mg protein extract), or an equal amount of rabbit monoclonal IgG control antibody (Abcam, cat. no. ab172730), overnight at 4°C with end-over-end mixing, to allow the formation of antibody/protein immune complexes. After addition of protein G-coated magnetic dynabeads (5 µL beads/µg antibody, ThermoFisher Scientific, cat. no. 13424229), the lysate+antibody+bead mixtures were incubated for 1 hour at 4°C with end-over-end mixing, to allow binding of the antibody/protein complexes to the beads. Beads were then washed three times with 1 mL cold wash buffer (25 mM Tris, 150 mM NaCl, 1 mM EDTA, 1% NP-40, 5% glycerol, pH

7.4), transferred to fresh low-protein binding tubes and proteins were eluted from the beads by incubating in 1.1x Laemmli buffer (containing no reducing agent) at RT for 10 minutes. Eluates were snap-frozen on dry ice and stored at -80°C until further processing. Cytoplasmic extracts, rather than whole-cell RIPA extracts, were used for IP following preliminary data that the efficiency of immunoprecipitation was higher for cytoplasmic extracts compared to RIPA lysates, owing to the more efficient capturing of the IP antibodies on protein G-coated Dynabeads (presumably as a result of the salt and/or detergent composition of the CER buffers being more favourable for this process compared to the composition of the 1x RIPA buffer containing SDS that was employed for protein extractions in the case of WB samples).

Sample processing of Tau IP eluates for proteomic analysis

Eluted proteins were subjected to proteomic processing as follows; all sample volumes were adjusted to 200 µL prior to in-solution digestion. Proteins were reduced in dithiothreitol (5 mM final concentration) and incubated for 45 minutes at RT. Samples were alkylated with iodoacetamide (20 mM final concentration) and incubated for 45 minutes at RT in the dark. Contaminating salts and detergents were removed following methanol/chloroform extraction of proteins. Briefly, to a sample volume of ~200 µL, 600 µL of methanol and 150 µL chloroform were added and vortexed. For protein precipitation, 450 µL of MilliQ H_2O was added, vortexed, and spun down (max. speed, tabletop centrifuge) at RT for 2 minutes. The upper aqueous phase was removed without disrupting the precipitate at the interface. Another 450 µL of methanol was added, vortexed, and centrifuged for 2 minutes at RT. The supernatant was discarded and precipitated protein discs were allowed to briefly air dry. Proteins were resuspended in 50 µL 6 M urea buffer with vortexing and brief sonication on ice. Prior to digestion, urea concentrations were reduced to a final concentration of <1 M by diluting the reaction mixture with 250 µL MilliQ H_2O. Trypsin (Sequencing Grade Modified Trypsin; Promega) was added in a 1:50 w/w ratio of enzyme:total protein and incubated overnight at 37°C. The following day, samples were acidified to 1% trifluoroacetic acid to stop the digestion reaction. Samples were desalted and concentrated using C-18 Sep-Pak (Waters) cartridges according to the manufacturer's instructions. Peptides were eluted in 500 µL buffer B (35% ACN, 65% MilliQ H_2O, 0.1% TFA). Eluted peptides were dried using a vacuum concentrator (Speedvac, Eppendorf) and stored at -20°C until analysis by mass spectrometry (MS). Prior to MS analysis, dried peptides were resuspended in buffer A (98% MilliQ H_2O, 2% ACN, 0.1% TFA).

Liquid chromatography-tandem mass spectrometry (LC-MS/MS) and data analysis

LC-MS/MS analysis was performed using a Dionex Ultimate 3000 nano-ultra high-pressure reverse-phase chromatography coupled on-line to a Q Exactive Orbitrap mass spectrometer (ThermoFisher Scientific). Prior to introduction to the MS, peptides were separated on an EASY-Spray PepMap RSLC C18 column (500 mm × 75 µm, 2 µm particle size, ThermoFisher Scientific) over a 60-minute gradient of 2-35% acetonitrile in 5% dimethyl sulfoxide, 0.1% formic acid at 250 nL/minute. The mass spectrometer was operated in data-dependent acquisition mode for automated switching between MS and MS/MS acquisition. Full MS survey scans were acquired from *m/z* 400-2,000 at a resolution of 70,000 at *m/z* 200 and AGC target of $3e^6$ ions for a maximum injection time of 100 ms. The top 15 most abundant precursor ions were selected for high collision dissociation fragmentation after isolation with a mass window of 1.6 Th and at a resolution of 17,500, with a maximum injection time of 128 ms. The normalised collision energy was 28%.

MS raw data were searched against the UniProtKB human sequence database (fused Uniprot/Trembl, 02/2020) and label-free quantitation (LFQ) was performed using MaxQuant Software (v1.6.10.43). Search parameters were set to include carbamidomethyl (C) as a fixed modification, with oxidation (M) and protein N-terminal acetylation included as variable modifications. A maximum of 2 missed cleavages were allowed, with matching between runs. LFQ was performed using unique peptides only. Label-free interaction data analysis was performed using Perseus (v1.6.10.43).

Sequencing of the PCR-amplified cDNA from HAP1 cells

RNA was extracted from HAP1 cells stored in TRI Reagent® (Sigma, cat. no. 9424) using the Direct-Zol™ RNA purification kit (Zyme Research, cat. no. R2070), following the manufacturer's instructions. cDNA was then synthesised from 1 µg RNA using the LunaScript® RT SuperMix Kit (New England Biolabs, cat. no. E3010), following the manufacturer's instructions. Tau cDNA was then PCR-amplified using OneTaq® Hot Start DNA Polymerase (New England Biolabs, cat. no. M0484), following the manufacturer's instructions. PCR products were separated by agarose gel electrophoresis, and the bands of interest were cut from the gel and purified using the QIAquick® Gel Extraction kit (QIAGEN, cat. no. 28704). The purified PCR products were then Sanger sequenced (Genewiz) using the same primers as were used for the PCR amplification. Primer sequences were as follows:

TGATGGAAGATCACGCTGGG (forward; exon 1) and TGATGGAAGATCACGCTGGG (reverse; exon 7).

Immunofluorescence labelling of FFPE tissue sections

FFPE tissue sections (4 µm thick) were dewaxed and rehydrated using a graded ethanol series (100%, 90%, 70%, methanol for 1 minute; final wash in ddH_2O for 5 minutes). Unless otherwise stated, antigens were unmasked by heat-induced epitope retrieval (HIER) using either 10 mM citrate solution (pH 6.0) and/ or Tris ethylenediaminetetraacetic acid (TE) solution (pH 9.0), prior to antibody labelling. Tissue sections were blocked with 5% normal goat serum in phosphate-buffered saline (PBS; blocking buffer) before incubating with primary antibodies either overnight at 4°C or for 2 hours at RT (for primary antibody details, see **Supp. Table S1**). For human brain tissue sections, all primary antibody incubations were performed overnight at 4°C. The resulting antigen-antibody complexes were detected using Alexa Fluor-conjugated secondary antibodies (1/400; AlexaFluor 488, 555, 647 - secondary antibodies are listed in Supp. **Table S1**). Cell nuclei were labelled with DAPI (1 µg/mL; Invitrogen, UK). Primary and secondary antibodies were diluted in DAKO REAL antibody diluent (Agilent, UK, cat. no. S202230-2). Tissue sections were washed three times in PBS to remove excess antibodies after each antibody incubation step. If applicable, tissue sections were then incubated with 0.5% Thioflavin S (ThS; diluted in water; Sigma Aldrich, cat. no. T1892) for 2 minutes, and were submerged in 100% ethanol 10 times. ThS-labelled slides were washed twice for 5 minutes in ddH_2O before proceeding. For labelling of human brain sections utilised for experiments shown in **Supp. Figs. S38 onwards**, primary and secondary antibodies were diluted in blocking buffer and autofluorescence from non-lipofuscin sources was quenched using the Vector® TrueVIEW® Autofluorescence Quenching Kit (Vector Laboratories, cat. no. SP-8500-15), following manufacturer's instructions. Sections were mounted in fluorescence mounting media (Agilent, UK, cat. no. S302380-2) before imaging. To create autofluorescence control slides for mouse brain tissues, mouse brain sections were labelled as above using the relevant Alexa Fluor-conjugated secondary antibodies (AlexaFluor 488, 555, 647) in the absence of primary antibodies.

Chromogenic immunolabelling of FFPE human brain tissue sections

FFPE tissue sections were dewaxed and rehydrated as above. Antigen retrieval was performed through a combination of heat-induced epitope retrieval (HIER) followed by treatment with formic acid. In brief, tissue sections were first boiled for 5 mins in a pressure cooker in 10 mM citrate solution (pH 6.0), then allowed to cool down and treated with formic acid for 10 mins at RT. Endogenous peroxidases were blocked in 3% H_2O_2 (Sigma, cat. no. H1009) in methanol for 20 mins at RT. Tissue sections were blocked with 5% horse serum in PBS containing 0.05% Tween-20 (blocking buffer) for 1 hr at RT, before incubating with primary antibodies diluted in blocking buffer overnight at 4°C. Sections were then washed three times in PBS containing 0.05% Tween-20 (wash buffer), incubated with biotinylated horse anti-mouse secondary antibodies (Vector Laboratories; cat. no. BA-2000) for 1 hr at RT. Excess secondary antibodies were removed through washing three times in PBS and the immunolabelled antigens were detected using the VECTASTAIN® Elite® ABC-HRP Kit (Vector Laboratories, cat. no. PK-6100) in combination with the ImmPACT® DAB Peroxidase Substrate Kit (Vector Laboratories, cat. no. SK-4105), following manufacturer's instructions. Nuclei were counterstained with hematoxylin (Sigma, cat. no. HHS128) for 1 min. Sections were then washed in ddH_2O, dehydrated, incubated 2x 10 mins in HistoClear and mounted in DPX mounting media (Sigma, cat. no. 06522) before imaging.

Image acquisition, processing and quantifications for IHC-IF data

For mouse brain sections, low-magnification images of whole-tissue sections were obtained through scanning slides on the Akoya PhenoImager HT™ Automated Quantitative Pathology Imaging System (CLS143455). Autofluorescence control slides (described above) were imaged to generate spectral libraries of the endogenous autofluorescence signatures detected in each imaging channel, to enable the removal of autofluorescence prior to image analysis. Regions of interest (ROIs) were re-imaged in high definition using the 40x objective and processed using the InForm™ image analysis platform (Akoya Biosciences, US). The software allows for the generation of unbiased, spectrally unmixed images in which autofluorescence has been removed computationally based on the autofluorescence signature detected for mouse brain tissues in each channel. This is achieved using the autofluorescence slides described above, following the manufacturer's instructions. Labelling was reviewed across the entire brain section with a particular focus on both the cortex and hippocampus. For rTg4510 mouse brains, signal was strongest in cortex (as neuronal loss in the hippocampus is pronounced in rTg4510 mice at this age), whereas images of the CA1 region of the hippocampus are provided for wildtype,

Mapt$^{-/-}$, and hTau mouse brains. . Figures were created using the HALO® (Indica Labs) image analysis platform.

Quantification analysis of the immunofluorescent signal was performed using HALO® (Indica Labs), a gold standard image analysis platform for quantitative analysis of IHC data. The Highplex FL module was used to quantify the signal intensity obtained with Tau antibodies in either untreated (control) or λPP-treated rTg4510 mouse brain sections. For each image, the ROI (cortex) was manually annotated. Efficiency of nuclear detection was assessed visually by comparison with the DAPI nuclear stain. The cytoplasm radius and cell size were set manually. Cells were categorised as either Tau-positive (low, medium or high) or Tau-negative. Statistical analysis to compare control (untreated) and λPP-treated sections was performed using a two-tailed, independent *t-test*.

For human brain sections immunolabelled by IHC-IF, high-magnification images were acquired on an Olympus Spin SR SoRA spinning disc confocal microscope. Images from different slides that were immunolabelled with the same antibody were acquired on the same day, using identical microscope settings. Where possible, microscope acquisition settings (e.g. laser intensity, gain) were kept as low as possible, such that signals detected the 555 nm channel originating from the autofluorescence of lipofuscin particles (which accumulate in the brains of aged individuals) was kept to a minimum. Alternatively, if possible and this was representative of the signals observed across the sample, images were acquired in areas containing fewer lipofuscin accumulations, to allow for a better visualisation of the Tau labelling signal. Secondary only control images were acquired at all different microscope settings employed for imaging the primary antibody-immunolabelled tissues. Images are provided for the highest and lowest combinations of laser/gain microscope settings for both the 488 nm and the 555 nm channels. In addition, areas of high-lipofuscin and low-lipofuscin were imaged for the secondary-only control images in the 555 nm channel, to provide examples of both.

For human brain sections immunolabelled by IHC-DAB (HRP), high-magnification images were acquired using a Leica DMI8 widefield microscope.

Sample dephosphorylation with lambda phosphatase treatment

To generate protein extracts for protein dephosphorylation, PhosSTOP™ phosphatase inhibitors were omitted from the lysis buffer but were added to control samples to protect protein phosphorylation.

Protein extracts were treated with lambda phosphatase (λPP; NEB, cat. no. 0753; 10 U enzyme per µg protein) according to manufacturer's instructions at 30°C for 2 hours. Control reactions were set up in the same manner, except that λPP was omitted and an equivalent volume of 50% molecular biology-grade glycerol (Sigma, cat. no. 356352) in ddH_2O (to mimic the buffer composition that λPP is supplied in) was added instead. Control samples were incubated on ice for 2 hours to protect protein phosphorylation. At the end of the incubation period, the reaction was terminated by adding 4x Laemmli sample buffer to all samples and heating at 95°C for 10 minutes. Samples were used immediately or stored at -20°C until further analysis.

To dephosphorylate WB membranes, membranes were treated with λPP (2000 U enzyme per 1 mL buffer) prepared according to according to manufacturer's instructions for 24 hours at RT on an orbital shaker. Control (untreated) membrane were mock treated in the same manner, except λPP was omitted and an equivalent volume of 50% molecular biology-grade glycerol in water (to mimic the buffer composition that λPP is supplied in) was added instead. Membranes were then blocked, probed and imaged, as described above. Pairs of untreated and treated membranes were probed with the same antibody in parallel and imaged using the same settings, to ensure that the signals are comparable.

To dephosphorylate FFPE tissue slides, following HIER, tissue sections were incubated in the presence (λPP-treated; 10,000 U λPP per mL of reaction buffer) or absence (control sections; equivalent volume of 50% glycerol in ddH_2O added to reaction buffer instead of enzyme) for 24 hours at RT, following manufacturer's instructions. Tissue sections were then washed in PBS, and a second HIER in citrate buffer (pH 6.0) was performed to cease phosphatase activity. Sections were then immunolabelled and mounted in fluorescence mounting media, as described above.

Identification of High-Performing Antibodies for the Reliable Detection of Tau Proteoforms by Western Blotting and Immunohistochemistry

Michael J. Ellis, Christiana Lekka, Katie L. Holden, Hanna Tulmin, Faheem Seedat, Darragh P. O'Brien, Shalinee Dhayal, Marie-Louise Zeissler, Jakob G. Knudsen, Benedikt M. Kessler, Noel G. Morgan, John A. Todd, Sarah J. Richardson, M. Irina Stefana[&]

[&] Affiliation and contact details of the corresponding author:

Centre for Human Genetics
University of Oxford
Roosevelt Drive
Oxford
OX3 7BN
United Kingdom

Email: irina.stefana@well.ox.ac.uk
(alternatively: maria.irina.stefana@gmail.com)

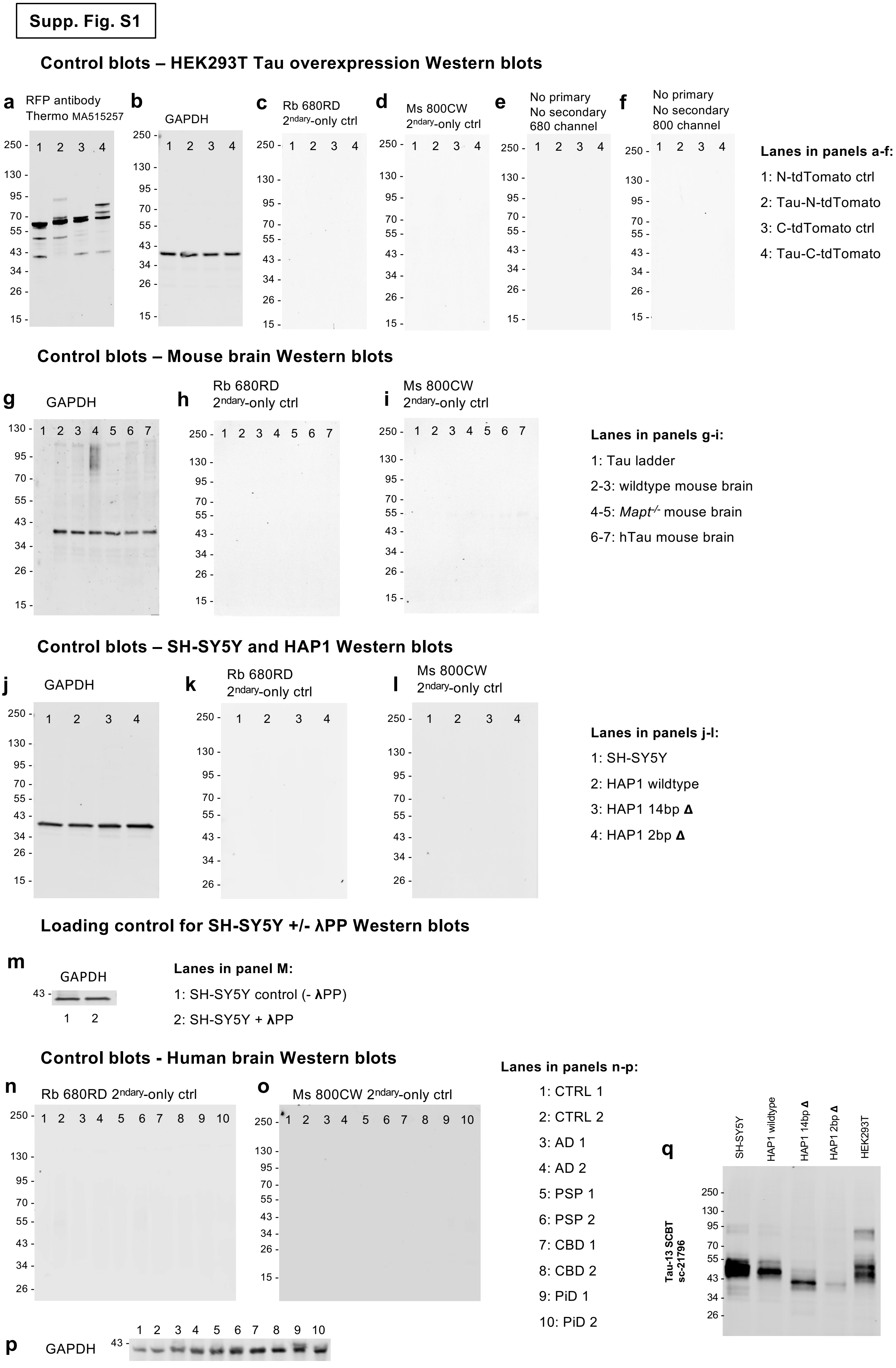
Supp. Fig. S1
Control blots – HEK293T Tau overexpression Western blots
a RFP antibody Thermo MA515257
b GAPDH
c Rb 680RD 2ndary-only ctrl
d Ms 800CW 2ndary-only ctrl
e No primary No secondary 680 channel
f No primary No secondary 800 channel
Lanes in panels a-f:
1: N-tdTomato ctrl
2: Tau-N-tdTomato
3: C-tdTomato ctrl
4: Tau-C-tdTomato
Control blots – Mouse brain Western blots
g GAPDH
h Rb 680RD 2ndary-only ctrl
i Ms 800CW 2ndary-only ctrl
Lanes in panels g-i:
1: Tau ladder
2-3: wildtype mouse brain
4-5: Mapt-/- mouse brain
6-7: hTau mouse brain
Control blots – SH-SY5Y and HAP1 Western blots
j GAPDH
k Rb 680RD 2ndary-only ctrl
l Ms 800CW 2ndary-only ctrl
Lanes in panels j-l:
1: SH-SY5Y
2: HAP1 wildtype
3: HAP1 14bp Δ
4: HAP1 2bp Δ
Loading control for SH-SY5Y +/- λPP Western blots
m GAPDH
Lanes in panel M:
1: SH-SY5Y control (- λPP)
2: SH-SY5Y + λPP
Control blots - Human brain Western blots
n Rb 680RD 2ndary-only ctrl
o Ms 800CW 2ndary-only ctrl
Lanes in panels n-p:
1: CTRL 1
2: CTRL 2
3: AD 1
4: AD 2
5: PSP 1
6: PSP 2
7: CBD 1
8: CBD 2
9: PiD 1
10: PiD 2
p GAPDH
q Tau-13 SCBT sc-21796
SH-SY5Y
HAP1 wildtype
HAP1 14bp Δ
HAP1 2bp Δ
HEK293T

Supplementary Figure S1. Control WB membranes. a: WB membrane of HEK293T cell lysates probed with an anti-RFP antibody to detect tdTomato expression. Note that bands corresponding to tdTomato alone (predicted MW of 54.2 kDa, observed at ~60 kDa) were present not only in control cells, but also in Tau-overexpressing cells, suggesting that a proportion of the tdTomato tag is cleaved off. Indeed, a faint band corresponding to the full-length tdTomato-tagged Tau (predicted MW 90.9 kDa, observed on WB just below 95 kDa) was only observed in tdTomato-N-Tau cells, indicating that most of the transgenic Tau protein is proteolytically processed. **b, g, j, m, p**: WB membranes probed with an anti-GAPDH antibody as a loading control. **c, h, k, n**: WB membranes probed with the anti-rabbit secondary antibody only, in the absence of a primary antibody, to identify non-specific signals that may arise from binding of the secondary antibody. **d, i, l, o**: WB membranes probed with the anti-mouse secondary antibody only, in the absence of a primary antibody, to identify non-specific signals that may arise from binding of the secondary antibody. **e, f**: WB membranes of HEK293T cell lysates were subjected to the same protocol as all other WB membranes shown in this study, with the exception that the primary and secondary antibodies were omitted, in order to assess background autofluorescence in the 700 nm (**e**) and 800 nm (**f**) detection channels, respectively. Order of samples for HEK293T blots (**a-f)**, mouse brain blots (**g-i**), SH-SY5Y and HAP1 blots (**j-l**), SH-SY5Y+/-λPP (**m**) and human brain blots (**n-p**) is the same as that shown in **Fig. 3** and **Supp. Figs. S4-S7, S10-S18**, in columns I, II, III, V and VI, respectively. **q:** To detect endogenous Tau expression in HEK293T cells, protein extracts (50 µg protein/lane) from SH-SY5Y, HAP1 and HEK293T cell lines were loaded on SDS-PAGE. WB membrane was probed with the Tau-13 “total” Tau antibody.

Supp. Fig. S2 – part I – IHC controls

a

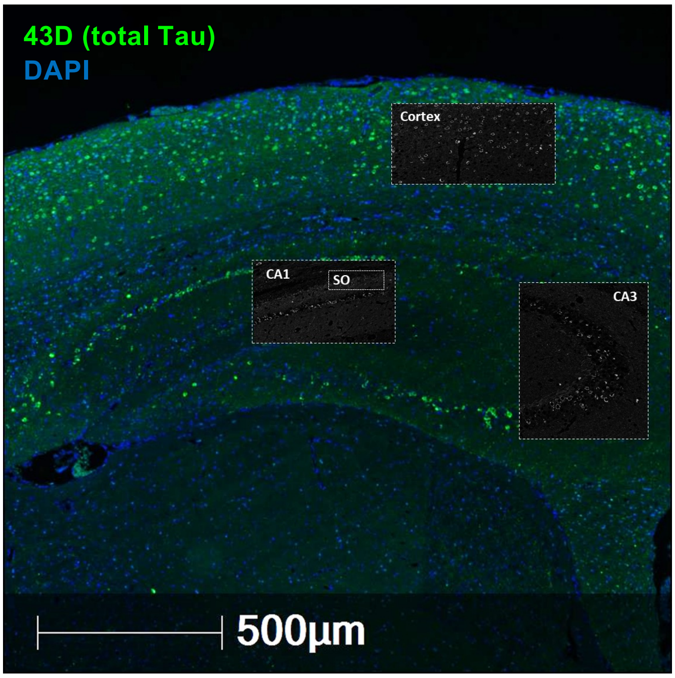

b

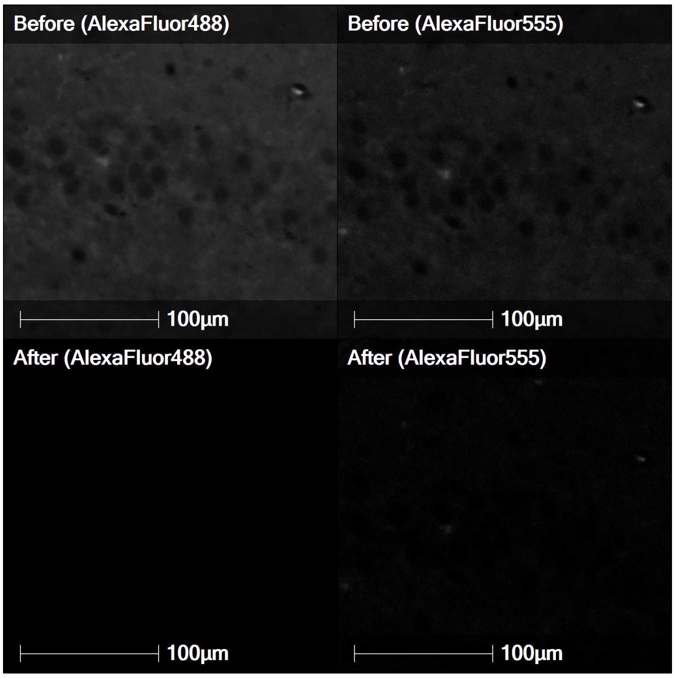

Supplementary Figure S2 (Part I). a: Overview of mouse brain regions (cortex, CA1, CA3, SO - outlined with white dashed line) imaged for the validation of Tau antibodies by IHC. Brain section shown originates from rTg4510 mice, was treated with λPP and was labelled with the 43D “total” Tau antibody (shown in green outside insets; shown in grayscale inside insets). Nuclei are labelled with DAPI (blue, outside insets). Scale bar = 500 μm. CA = *cornu ammonis*, SO = *stratum oriens*. **b:** Micrographs of CA3 brain region from rTg4510 mouse FFPE brain sections immunolabelled with AlexaFluor-conjugated secondary antibodies (AlexaFluor488 - left; AlexaFluor555 - right), in the absence of primary antibodies, before (top row) and after (bottom row) autofluorescence removal. Respective fluorescent signals are shown in grayscale. Scale bars = 100 μm**.**

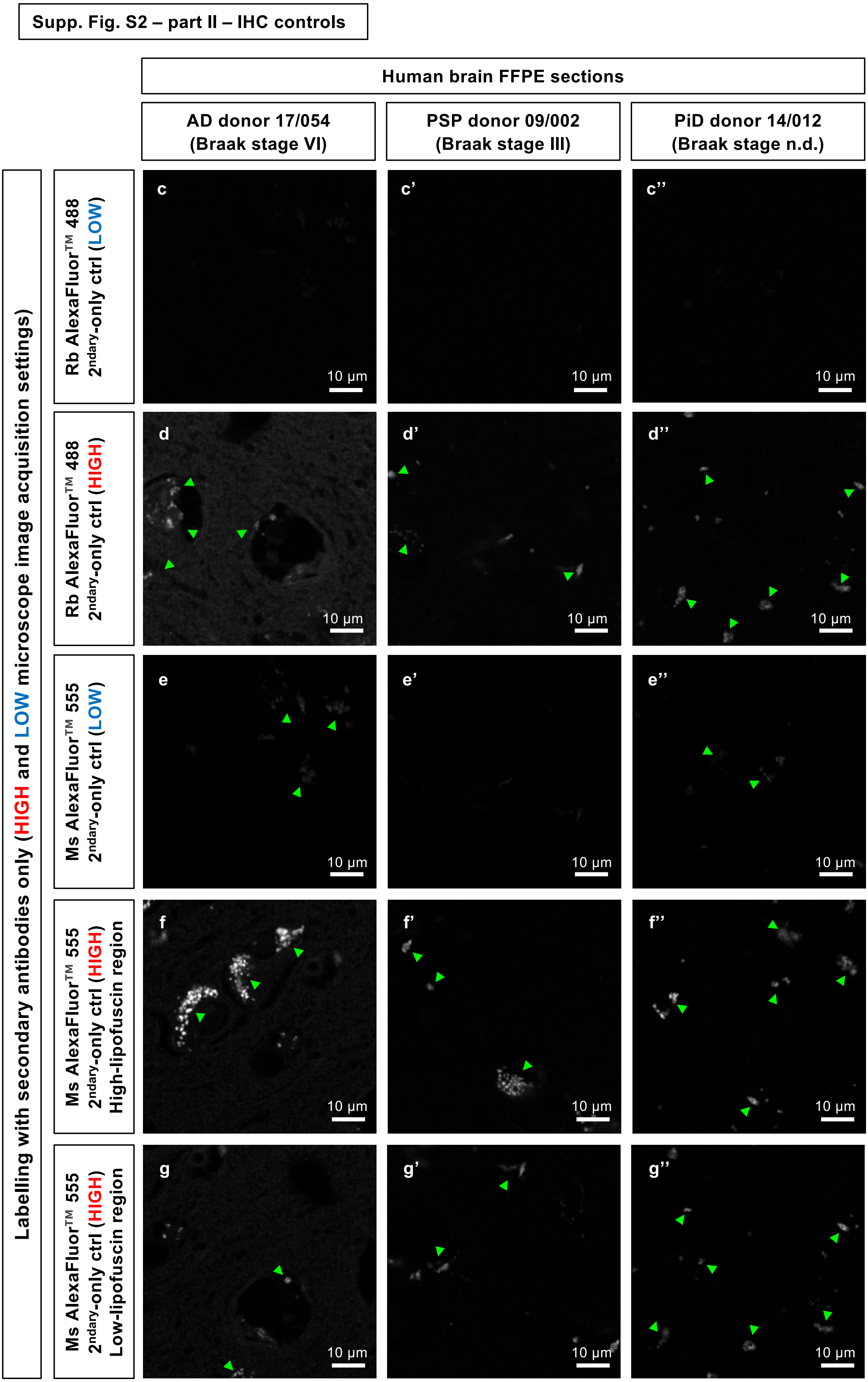
Supp. Fig. S2 – part II – IHC controls
Human brain FFPE sections
AD donor 17/054 (Braak stage VI)
PSP donor 09/002 (Braak stage III)
PiD donor 14/012 (Braak stage n.d.)
Labelling with secondary antibodies only (HIGH and LOW microscope image acquisition settings)
Rb AlexaFluor™ 488 2ndary-only ctrl (LOW)
Rb AlexaFluor™ 488 2ndary-only ctrl (HIGH)
Ms AlexaFluor™ 555 2ndary-only ctrl (LOW)
Ms AlexaFluor™ 555 2ndary-only ctrl (HIGH) High-lipofuscin region
Ms AlexaFluor™ 555 2ndary-only ctrl (HIGH) Low-lipofuscin region
c
c'
c''
d
d'
d''
e
e'
e''
f
f'
f''
g
g'
g''
10 µm

Supplementary Figure S2 (Part II). c-g'': "Secondary only" control experimental data to demonstrate specificity of the Tau immunolabelling observed in FFPE human brain sections from tauopathy donors. In this and **Supp. Figs. S30, S32, S36, S37, S42 and S43**, green arrowheads point to lipofuscin particles, which emit autofluorescence in all channels, with the signal being highest in the red region of the spectrum and, therefore, being detected as brightest in the 555 nm channel. The intensity of the lipofuscin autofluorescent signal depends on the microscope image acquisition settings, with the signal intensity being higher at high laser/gain settings (HIGH) compared to low laser/gain settings (LOW). Even when imaged at HIGH settings, the lipofuscin signal intensity was heterogenous across the cortex with some areas containing neurons with high levels of lipofuscin deposits (high-lipofuscin regions, **f-f''**), while neurons in neighbouring areas may display much lower lipofuscin accumulation (low-lipofuscin regions, **g-g''**). Scale bars = 10 μm. Brain sections originated from the dorsolateral prefrontal cortex (Brodmann areas 9/46).

Supp. Fig. S3

a

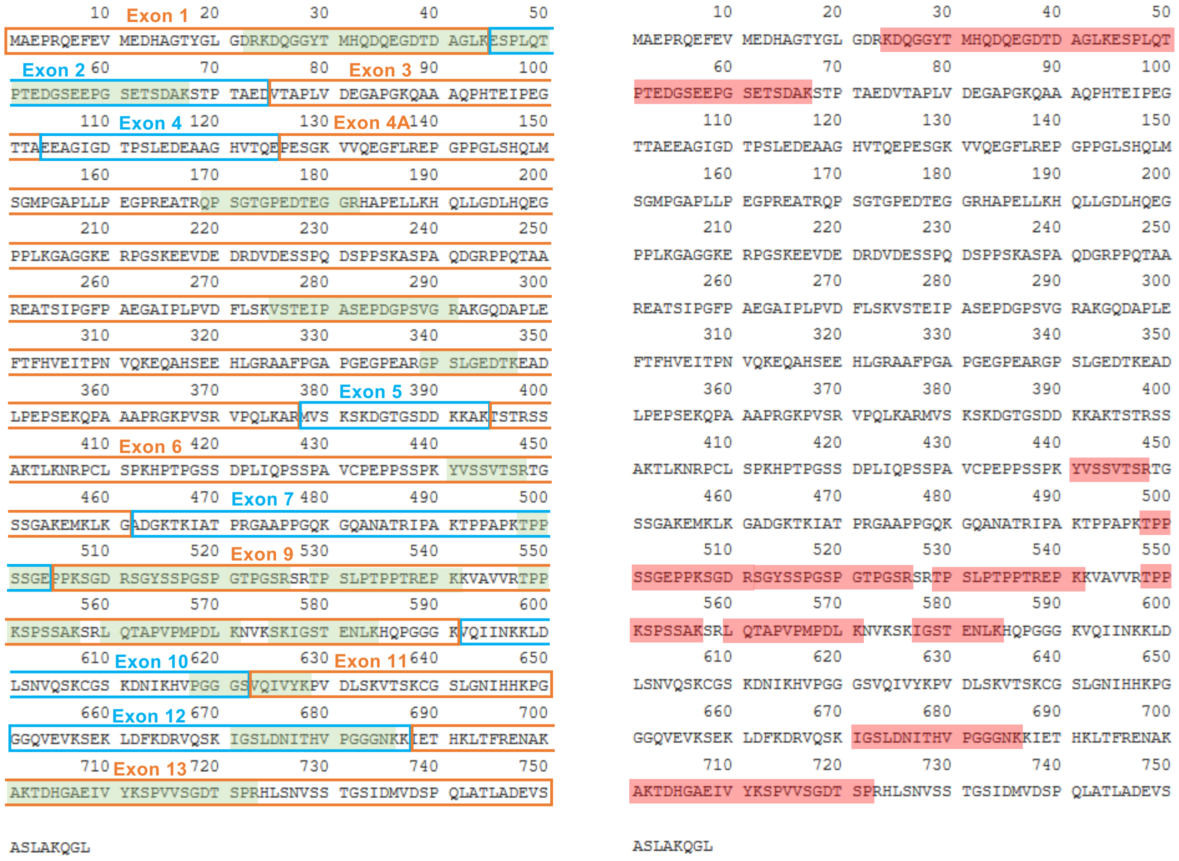

b

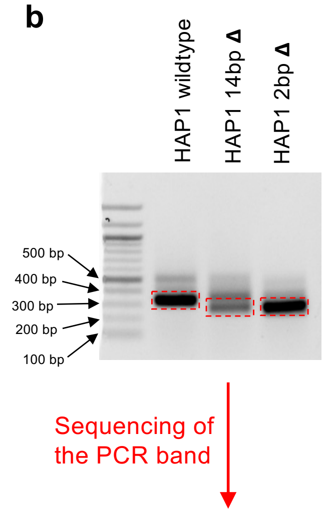

Predicted PCR product length for different transcripts:

Full-length, wildtype transcript encoding 2N Tau isoforms (not expressed in HAP1 cells; includes exons 1, 2, 3, 3, 5 and 7) = **527 bp**

Wildtype transcript encoding 0N Tau isoform (predicted to be the predominant transcript in HAP1 cells; excludes exons 2 and 3 compared to the full-length, wildtype transcript) = **353 bp**

Transcript encoding 0N Tau isoform generated through skipping exon 4 (predicted to be expressed in the CRISPR/Cas9-edited HAP1 cells; excludes exons 2, 3 and 4 compared to the full-length, wildtype transcript) = **291 bp**

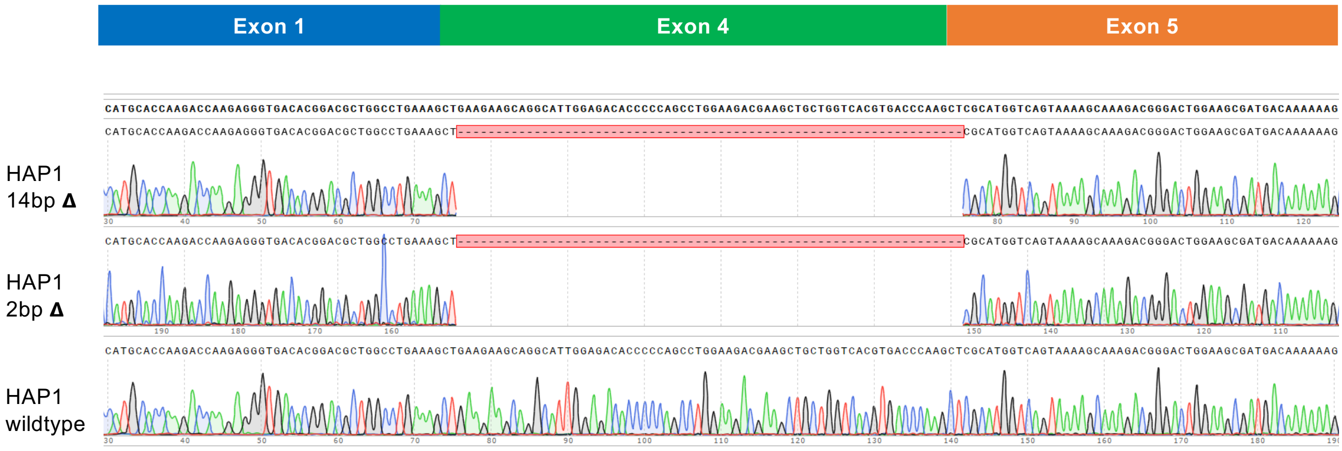

Supplementary Figure S3. Tau peptides detected by mass-spec in cytoplasmic fractions from wildtype and *MAPT*-edited HAP1 cells following immunoprecipitation of Tau. a: Peptides detected in the wildtype/parental cell line (left, detected peptides highlighted in green) and in the 2-bp deletion *MAPT*-edited HAP1 cell line (right, detected peptides highlighted in red) are shown mapped to the amino acid sequence of the canonical Uniprot human Tau isoform (Uniprot ID P10636-1; 758 amino acids). Sequences encoded by the different *MAPT* exons are outlined and labelled on the left in alternating orange/blue colours. **b:** Sequencing of the PCR-amplified region of the *MAPT* cDNA that spans exons 1 to 4 showed that the canonical 0N Tau transcript containing exon 4 is expressed in wildtype cells, whereas exon 4 is skipped in the CRISPR-edited HAP1 cell lines giving rise to a non-canonical 0N Tau transcript where exon 1 is followed by exon 5.

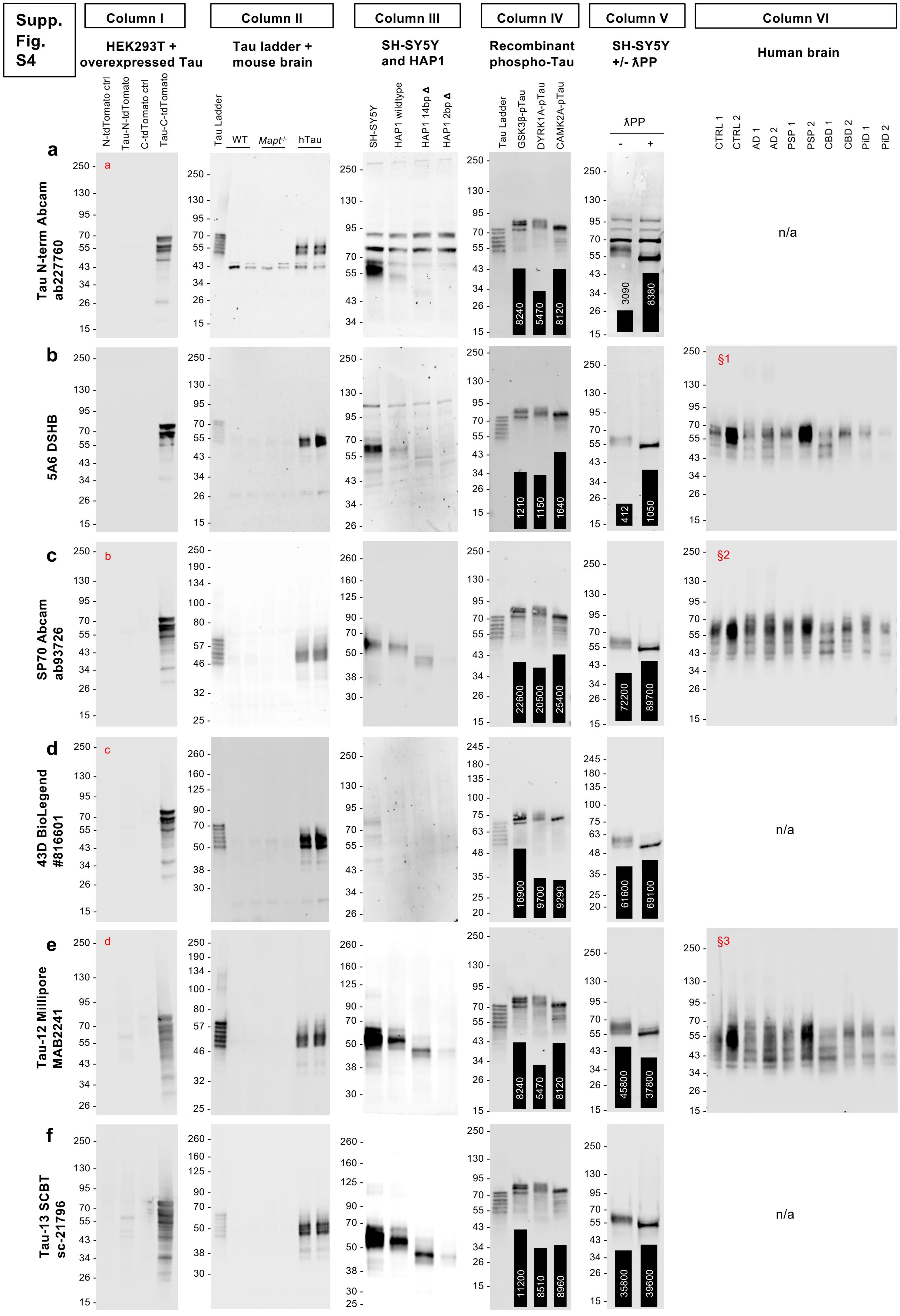

Supp. Fig. S4
Column I
HEK293T + overexpressed Tau
Column II
Tau ladder + mouse brain
Column III
SH-SY5Y and HAP1
Column IV
Recombinant phospho-Tau
Column V
SH-SY5Y +/- λPP
Column VI
Human brain
N-tdTomato ctrl
Tau-N-tdTomato
C-tdTomato ctrl
Tau-C-tdTomato
Tau Ladder
WT
Mapt-/-
hTau
SH-SY5Y
HAP1 wildtype
HAP1 14bp Δ
HAP1 2bp Δ
Tau Ladder
GSK3β-pTau
DYRK1A-pTau
CAMK2A-pTau
λPP
-
+
CTRL 1
CTRL 2
AD 1
AD 2
PSP 1
PSP 2
CBD 1
CBD 2
PiD 1
PiD 2
a
Tau N-term Abcam ab227760
8240
5470
8120
3090
8380
n/a
b
5A6 DSHB
1210
1150
1640
412
1050
§1
c
SP70 Abcam ab93726
22600
20500
25400
72200
89700
§2
d
43D BioLegend #816601
16900
9700
9290
61600
69100
n/a
e
Tau-12 Millipore MAB2241
8240
5470
8120
45800
37800
§3
f
Tau-13 SCBT sc-21796
11200
8510
8960
35800
39600
n/a

Supplementary Figure S4. Validation of PTM-agnostic "total" Tau antibodies by WB (part 1). For this and all subsequent figures up to and including Supp. Fig. S18, WBs shown in columns I to VI are as follows: WB of lysates from HEK293T cells overexpressing 0N3R human Tau and corresponding control cells (**column I**); WB of recombinant human Tau ladder (5 ng/isoform/lane), plus adult mouse brain lysates from wildtype, *Mapt*$^{-/-}$ and hTau mice (**column II**); WB of lysates from SH-SY5Y neuroblastoma cells, plus HAP1 cells: parental (wildtype) and two cell lines carrying either a 14 bp deletion (14 bp Δ) or a 2-bp deletion (2 bp Δ) in *MAPT* exon 4 (**column III**); WB of recombinant human Tau ladder (50 ng/isoform/lane) plus recombinant 2N4R Tau that has been phosphorylated by one of three known Tau kinases: GSK3β, DYRK1A or CAMKIIA (**column IV**); WB of lysates from SH-SY5Y neuroblastoma cells that have been either untreated (-) or treated (+) with λPP (**column V**); WB of RIPA protein extracts from human brain tissue collected from control individuals and patients diagnosed with different tauopathies (AD, PSP, CBD and PiD) (**column VI**). For WBs in columns IV and V, quantifications of the Tau signal intensity for each lane are shown superimposed on each WB image as a bar chart, with the respective value [a.u.] printed on or above each bar of the chart. In this and all subsequent figures, where adjusting the brightness/contrast display settings revealed additional bands or allowed for better visualisation of weak signals, this is indicated in lowercase red lettering displayed in the upper left corner of blots (or the § symbol followed by a numeral, in the case of human brain WBs). The corresponding adjusted WB images are shown in **Supp. Fig. S23** for blots in columns I to V, and in **Supp. Figs. S24-S26** for human brain blots in column VI. In this and all subsequent figures, where applicable, MW markers [kDa] are indicated on the left of each blot. **a-f:** WB membranes shown in each panel (**row**) were probed with a different Tau antibody: N-term Abcam ab227760 (**a**); 5A6 DSHB 5A6 (**b**); SP70 Abcam ab93726 (**c**); 43D BioLegend 816601 (**d**); Tau-12 Merck Millipore MAB2241 (**e**); Tau-13 Santa Cruz sc-21796 (**f**).

Supp. Fig. S5

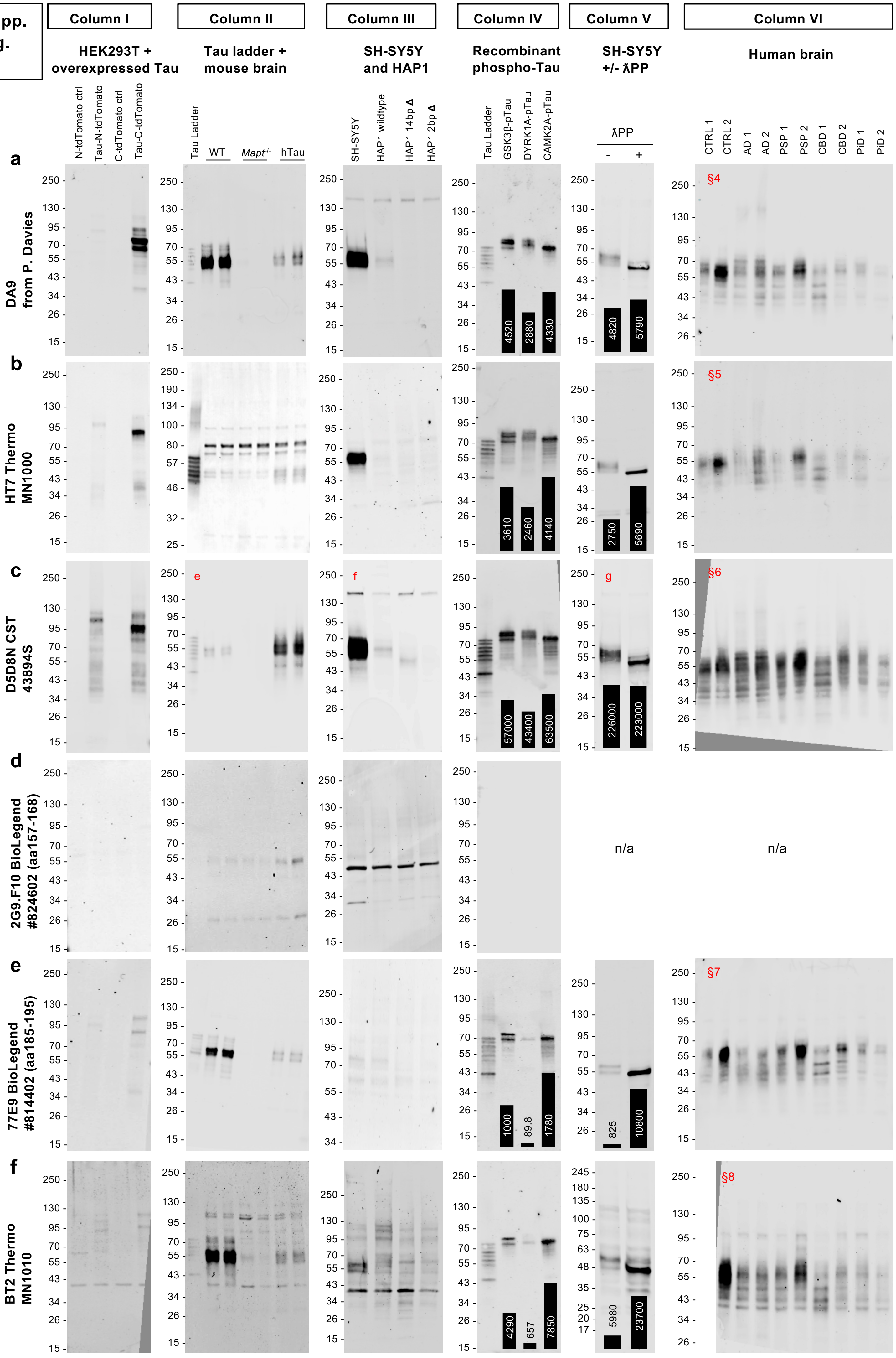
Column I
HEK293T + overexpressed Tau
Column II
Tau ladder + mouse brain
Column III
SH-SY5Y and HAP1
Column IV
Recombinant phospho-Tau
Column V
SH-SY5Y +/- λPP
Column VI
Human brain
N-tdTomato ctrl
Tau-N-tdTomato
C-tdTomato ctrl
Tau-C-tdTomato
Tau Ladder
WT
Mapt-/-
hTau
SH-SY5Y
HAP1 wildtype
HAP1 14bp Δ
HAP1 2bp Δ
Tau Ladder
GSK3β-pTau
DYRK1A-pTau
CAMK2A-pTau
λPP
-
+
CTRL 1
CTRL 2
AD 1
AD 2
PSP 1
PSP 2
CBD 1
CBD 2
PiD 1
PiD 2
a
DA9 from P. Davies
4520
2880
4330
4820
5790
§4
b
HT7 Thermo MN1000
3610
2460
4140
2750
5690
§5
c
D5D8N CST 43894S
e
f
57000
43400
63500
g
226000
223000
§6
d
2G9.F10 BioLegend #824602 (aa157-168)
n/a
n/a
e
77E9 BioLegend #814402 (aa185-195)
1000
89.8
1780
825
10800
§7
f
BT2 Thermo MN1010
4290
657
7850
5980
23700
§8

Supplementary Figure S5. Validation of PTM-agnostic “total” Tau antibodies by WB (part 2). a-f: WB membranes shown in each panel (**row**) were probed with a different Tau antibody: DA9 from P. Davies (**a**); HT7 ThermoFisher Scientific MN1000 (**b**); D5D8N Cell Signalling 43894S (**c**); 2G9.F10 BioLegend 824602 (**d**); clone 77E9 BioLegend 814402 (**e**); BT2 ThermoFisher Scientific MN1010 (**f**).

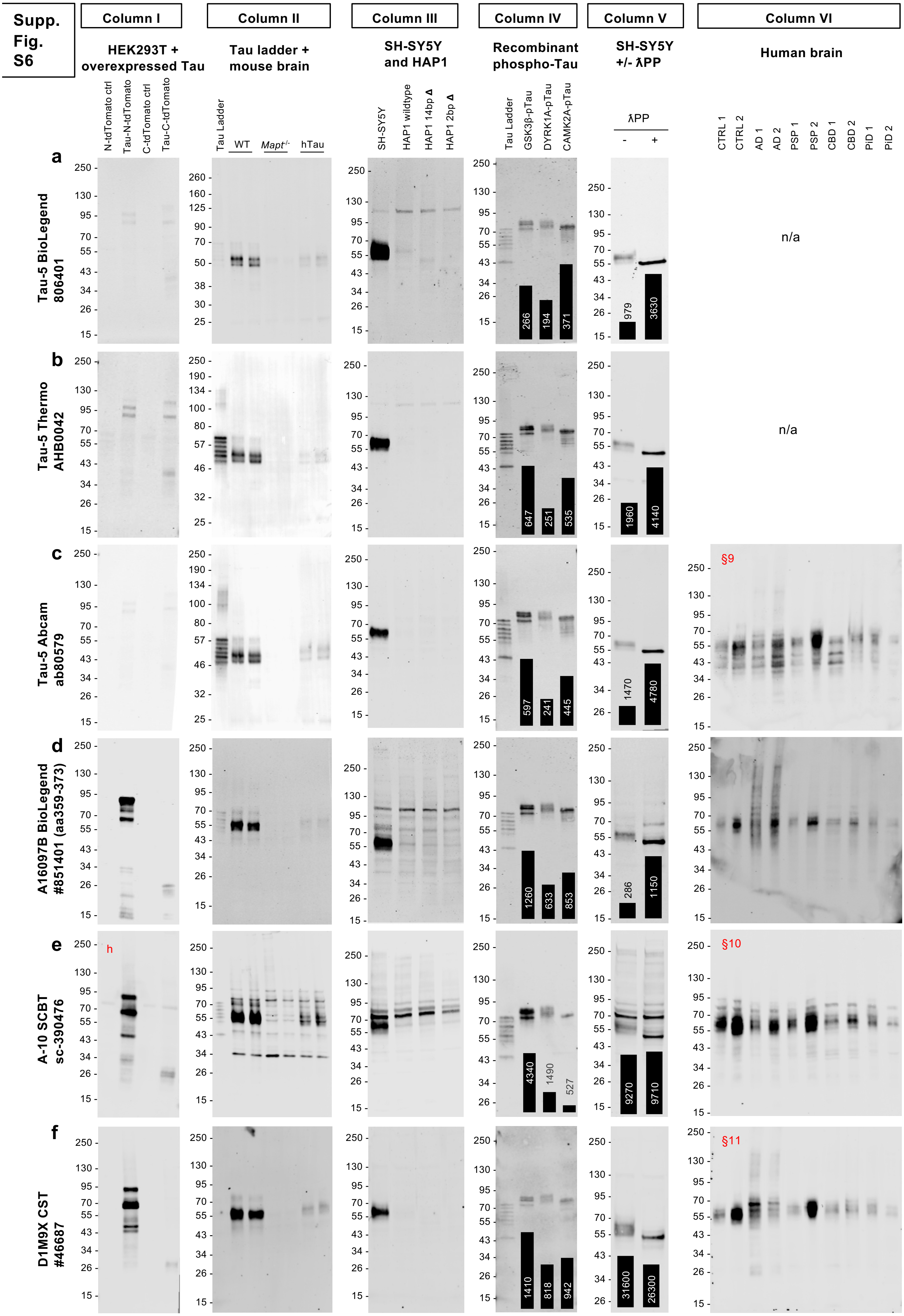

Supp. Fig. S6
Column I
HEK293T + overexpressed Tau
Column II
Tau ladder + mouse brain
Column III
SH-SY5Y and HAP1
Column IV
Recombinant phospho-Tau
Column V
SH-SY5Y +/- λPP
Column VI
Human brain
N-tdTomato ctrl
Tau-N-tdTomato
C-tdTomato ctrl
Tau-C-tdTomato
Tau Ladder
WT
Mapt-/-
hTau
SH-SY5Y
HAP1 wildtype
HAP1 14bp Δ
HAP1 2bp Δ
Tau Ladder
GSK3β-pTau
DYRK1A-pTau
CAMK2A-pTau
λPP
-
+
CTRL 1
CTRL 2
AD 1
AD 2
PSP 1
PSP 2
CBD 1
CBD 2
PiD 1
PiD 2
a
Tau-5 BioLegend 806401
266
194
371
979
3630
n/a
b
Tau-5 Thermo AHB0042
647
251
535
1960
4140
n/a
c
Tau-5 Abcam ab80579
597
241
445
1470
4780
§9
d
A16097B BioLegend #851401 (aa359-373)
1260
633
853
286
1150
e
A-10 SCBT sc-390476
h
4340
1490
527
9270
9710
§10
f
D1M9X CST #46687
1410
818
942
31600
26300
§11

Supplementary Figure S6. Validation of PTM-agnostic “total” Tau antibodies by WB (part 3). a-f: WB membranes shown in each panel (**row**) were probed with a different Tau antibody: Tau-5 BioLegend 806401 (**a**); Tau-5 ThermoFisher Scientific AHB0042 (**b**); Tau-5 Abcam ab80579 (**c**); A16097B BioLegend 851401 (**d**); A-10 Santa Cruz sc-390476 (**e**); D1M9X CST 46687 (**f**).

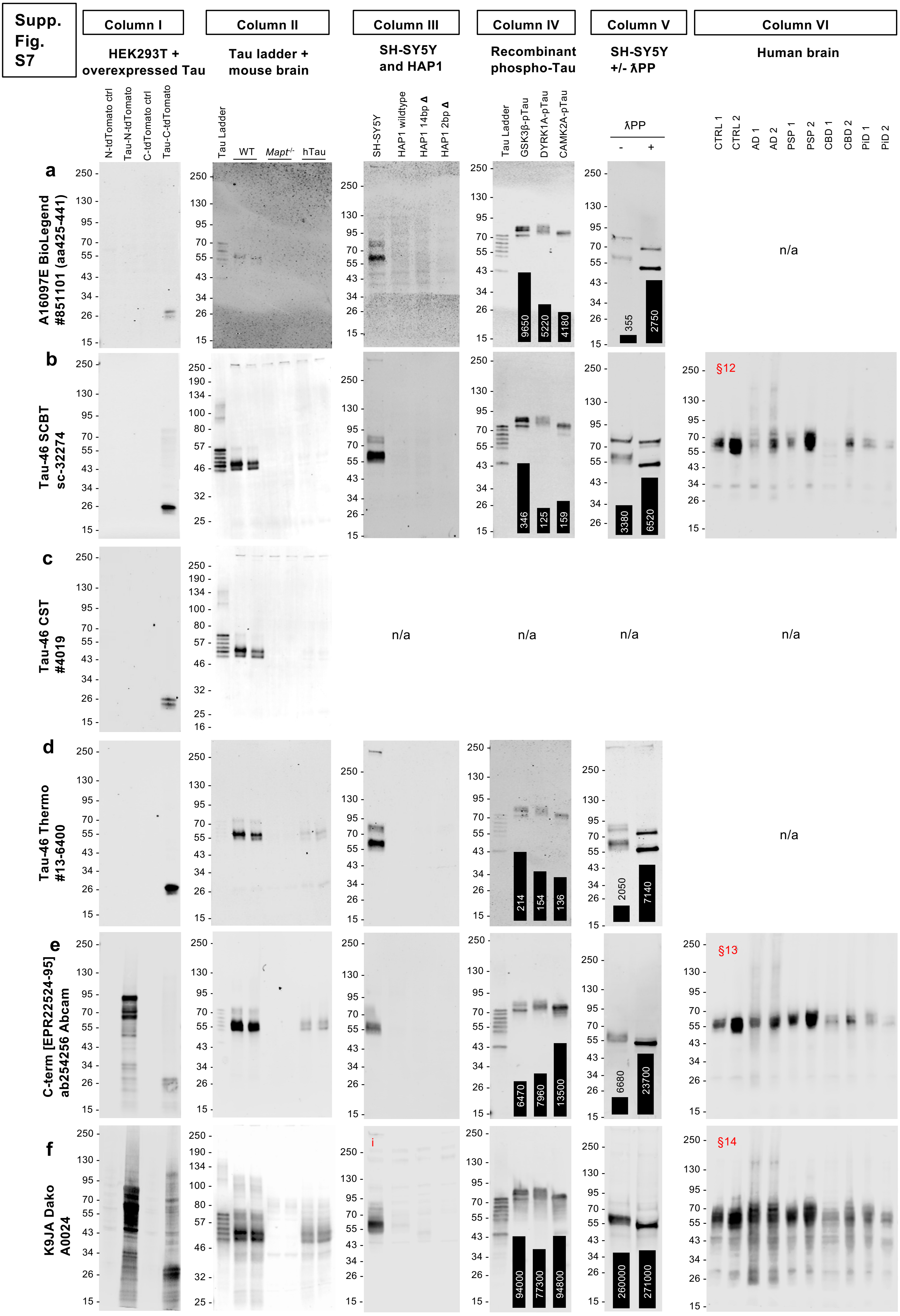

Supp. Fig. S7
Column I
HEK293T + overexpressed Tau
Column II
Tau ladder + mouse brain
Column III
SH-SY5Y and HAP1
Column IV
Recombinant phospho-Tau
Column V
SH-SY5Y +/- λPP
Column VI
Human brain
N-tdTomato ctrl
Tau-N-tdTomato
C-tdTomato ctrl
Tau-C-tdTomato
Tau Ladder
WT
Mapt-/-
hTau
SH-SY5Y
HAP1 wildtype
HAP1 14bp Δ
HAP1 2bp Δ
Tau Ladder
GSK3β-pTau
DYRK1A-pTau
CAMK2A-pTau
λPP
-
+
CTRL 1
CTRL 2
AD 1
AD 2
PSP 1
PSP 2
CBD 1
CBD 2
PiD 1
PiD 2
a
A16097E BioLegend #851101 (aa425-441)
9650
5220
4180
355
2750
n/a
b
Tau-46 SCBT sc-32274
346
125
159
3380
6520
§12
c
Tau-46 CST #4019
n/a
n/a
n/a
n/a
d
Tau-46 Thermo #13-6400
214
154
136
2050
7140
n/a
e
C-term [EPR22524-95] ab254256 Abcam
6470
7960
13500
6680
23700
§13
f
K9JA Dako A0024
i
94000
77300
94800
260000
271000
§14

Supplementary Figure S7. Validation of PTM-agnostic "total" Tau antibodies by WB (part 4). a-f: WB membranes shown in each panel (**row**) were probed with a different Tau antibody: A16097E BioLegend 851101 (**a**); Tau-46 Santa Cruz sc-32274 (**b**); Tau-46 Cell Signalling 4019S (**c**); Tau-46 ThermoFisher Scientific 13-6400 (**d**); EPR22524-95 Abcam ab254256 (**e**); K9JA Dako A0024 (**f**).

Supp. Fig. S8

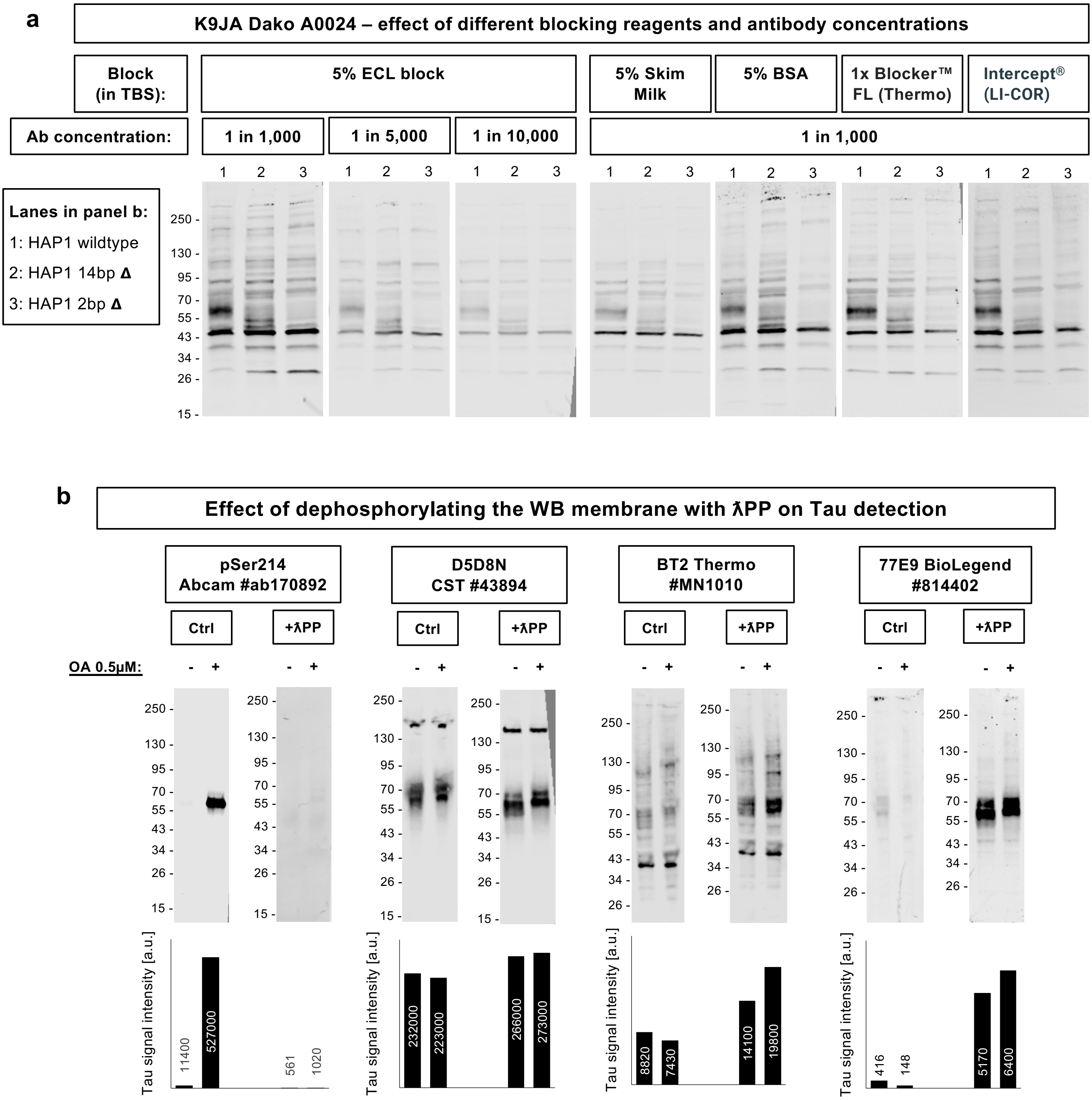
a
K9JA Dako A0024 – effect of different blocking reagents and antibody concentrations
Block (in TBS):
5% ECL block
5% Skim Milk
5% BSA
1x Blocker™ FL (Thermo)
Intercept® (LI-COR)
Ab concentration:
1 in 1,000
1 in 5,000
1 in 10,000
1 in 1,000
Lanes in panel b:
1: HAP1 wildtype
2: HAP1 14bp Δ
3: HAP1 2bp Δ
250
130
95
70
55
43
34
26
15
b
Effect of dephosphorylating the WB membrane with λPP on Tau detection
pSer214 Abcam #ab170892
D5D8N CST #43894
BT2 Thermo #MN1010
77E9 BioLegend #814402
Ctrl
+λPP
OA 0.5µM:
Tau signal intensity [a.u.]
11400
527000
561
1020
232000
223000
266000
273000
8820
7430
14100
19800
416
148
5170
6400

Supplementary Figure S8. a: The effect of different primary antibody concentrations and blocking reagents on non-selective cross-reactivity observed with the polyclonal K9JA antibody (Dako, cat. no. A0024). **b: Treating the WB membrane with λPP increased the immunoreactivity of the BT2 and 77E9 antibodies.** Whole-cell RIPA lysates from SH-SY5Y cells that had been treated with 0.5 μM okadaic acid (OA +), a potent phosphatase inhibitor, or an equal volume of DMSO as a control (OA -) were separated by SDS-PAGE and transferred onto PVDF membrane. Pairs oe WB membrane were then either dephosphorylated through treatment with λPP (+λPP) or were incubated in buffer alone (ctrl), prior to probing with one of four primary antibodies: pSer214, D5D8N, BT2 or 77E9. OA treatment dramatically increased Tau phosphorylation, as detected with the pSer214 antibody and this signal was abrogated following λPP treatment of the WB membrane. However, OA treatment did not affect overall Tau protein levels, as detected with the PTM-agnostic "total" Tau antibody D5D8N, which yielded comparable immunoreactive signals prior and following λPP treatment. In contrast, immunoreactivity of both the BT2 and 77E9 antibodies was enhanced following λPP treatment of the WB membrane, with the fold-increase in signal intensity being higher for the OA-treated samples compared to lysates from cells that had not been treated with OA. Membranes were scanned using the same settings and are displayed at the same brightness/contrast settings. Quantifications of the Tau signal intensity [a.u.] detected in each lane are shown below the blots.

Supp. Fig. S9

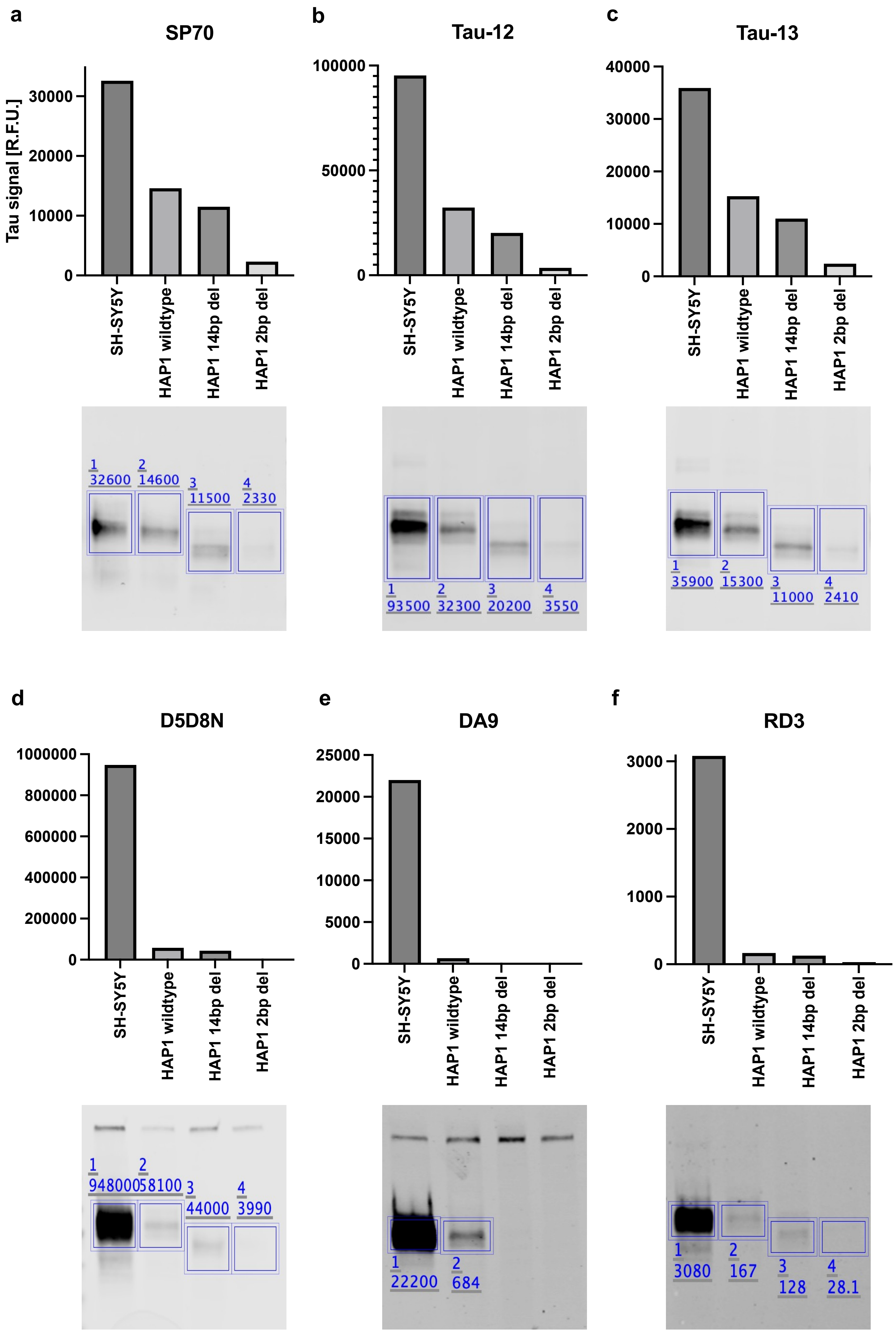
a
SP70
Tau signal [R.F.U.]
30000
20000
10000
0
SH-SY5Y
HAP1 wildtype
HAP1 14bp del
HAP1 2bp del
1 32600
2 14600
3 11500
4 2330
b
Tau-12
100000
50000
0
1 93500
2 32300
3 20200
4 3550
c
Tau-13
40000
30000
20000
10000
0
1 35900
2 15300
3 11000
4 2410
d
D5D8N
1000000
800000
600000
400000
200000
0
1 948000
2 58100
3 44000
4 3990
e
DA9
25000
20000
15000
10000
5000
0
1 22200
2 684
f
RD3
3000
2000
1000
0
1 3080
2 167
3 128
4 28.1

Supplementary Figure S9. Tau protein expression levels in SH-SY5Y and HAP1 cell lines. Quantifications of the Tau band intensities detected by WB in lysates from SH-SY5Y, HAP1 wildtype, HAP1 14 bp Δ and HAP1 2 bp Δ cells as detected with the SP70 (**a**), Tau-12 (**b**), Tau-13 (**c**), D5D8N (**d**), DA9 (**e**) and RD3 (**f**) antibodies. Shown below each graph is the respective WB membrane with the areas used for quantifications marked by rectangles and the detected signal intensity value (R.F.U.) shown above each rectangle. The WB membranes that were used for quantifications and are shown in this figure are the same ones as those shown in the third column in **Supp. Figs. 4c, 4e, 4f, 5c, 5a and 18a**. Raw signal intensity values are reported as relative fluorescence units (R.F.U.).

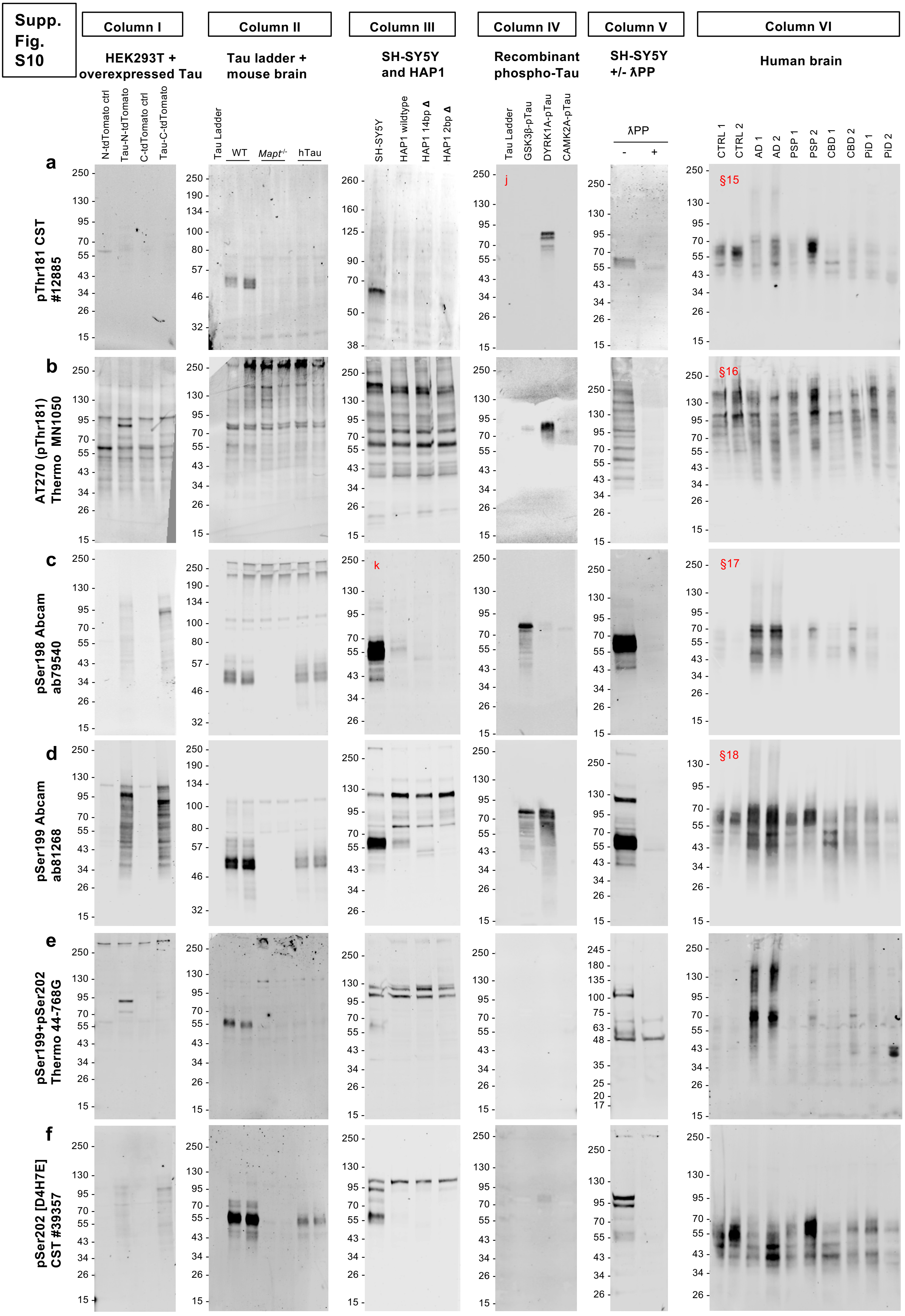

Supp. Fig. S10
Column I
HEK293T + overexpressed Tau
Column II
Tau ladder + mouse brain
Column III
SH-SY5Y and HAP1
Column IV
Recombinant phospho-Tau
Column V
SH-SY5Y +/- λPP
Column VI
Human brain
N-tdTomato ctrl
Tau-N-tdTomato
C-tdTomato ctrl
Tau-C-tdTomato
Tau Ladder
WT
Mapt-/-
hTau
SH-SY5Y
HAP1 wildtype
HAP1 14bp Δ
HAP1 2bp Δ
Tau Ladder
GSK3β-pTau
DYRK1A-pTau
CAMK2A-pTau
λPP
-
+
CTRL 1
CTRL 2
AD 1
AD 2
PSP 1
PSP 2
CBD 1
CBD 2
PiD 1
PiD 2
a
pThr181 CST #12885
j
§15
b
AT270 (pThr181) Thermo MN1050
§16
c
pSer198 Abcam ab79540
k
§17
d
pSer199 Abcam ab81268
§18
e
pSer199+pSer202 Thermo 44-768G
f
pSer202 [D4H7E] CST #39357

Supplementary Figure S10. Validation of phospho-Tau antibodies by WB (part 1). a-f: WB membranes shown in each panel (**row**) were probed with a different Tau antibody: pThr181 Cell Signalling 12885S (**a**); AT270 ThermoFisher Scientific MN1050 (**b**); pSer198 Abcam ab79540 (**c**); pSer199 Abcam ab81268 (**d**); pSer199+pSer202 ThermoFisher Scientific 44-768G (**e**); pSer202 Cell Signalling 39357 (**f**).

Supp. Fig. S11

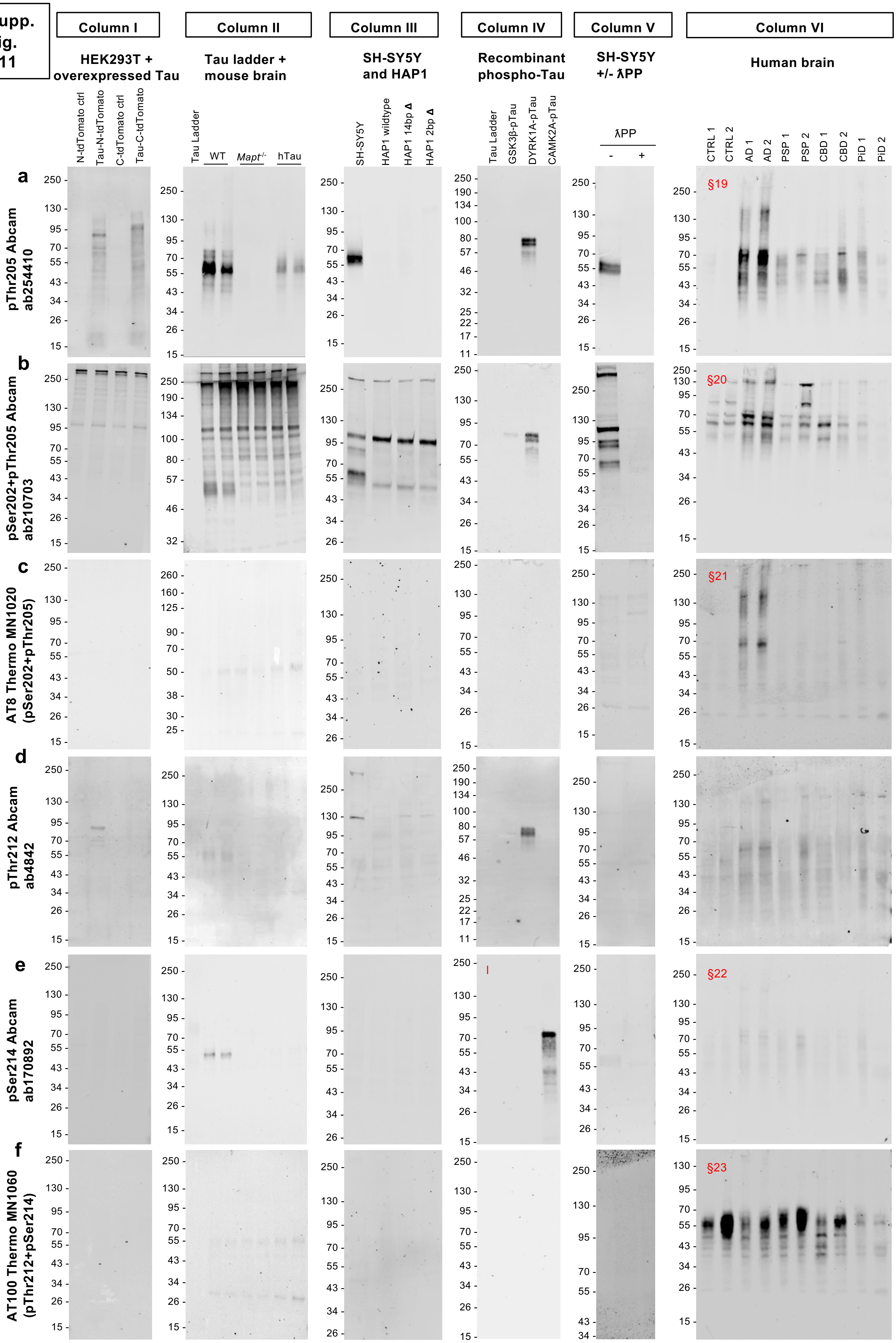
Column I
HEK293T + overexpressed Tau
N-tdTomato ctrl
Tau-N-tdTomato
C-tdTomato ctrl
Tau-C-tdTomato
Column II
Tau ladder + mouse brain
Tau Ladder
WT
Mapt-/-
hTau
Column III
SH-SY5Y and HAP1
SH-SY5Y
HAP1 wildtype
HAP1 14bp Δ
HAP1 2bp Δ
Column IV
Recombinant phospho-Tau
Tau Ladder
GSK3β-pTau
DYRK1A-pTau
CAMK2A-pTau
Column V
SH-SY5Y +/- λPP
λPP
- +
Column VI
Human brain
CTRL 1
CTRL 2
AD 1
AD 2
PSP 1
PSP 2
CBD 1
CBD 2
PiD 1
PiD 2
a
pThr205 Abcam ab254410
b
pSer202+pThr205 Abcam ab210703
c
AT8 Thermo MN1020 (pSer202+pThr205)
d
pThr212 Abcam ab4842
e
pSer214 Abcam ab170892
f
AT100 Thermo MN1060 (pThr212+pSer214)
§19
§20
§21
§22
§23
I

Supplementary Figure S11. Validation of phospho-Tau antibodies by WB (part 2). a-f: WB membranes shown in each panel (**row**) were probed with a different Tau antibody: pThr205 Abcam ab254410 (**a**); pSer202+pThr205 Abcam ab210703 (**b**); AT8 ThermoFisher Scientific MN1020 (**c**); pThr212 Abcam ab4842 (**d**); pSer214 Abcam ab170892 (**e**); AT100 ThermoFisher Scientific MN1060 (**f**).

Supp. Fig. S12

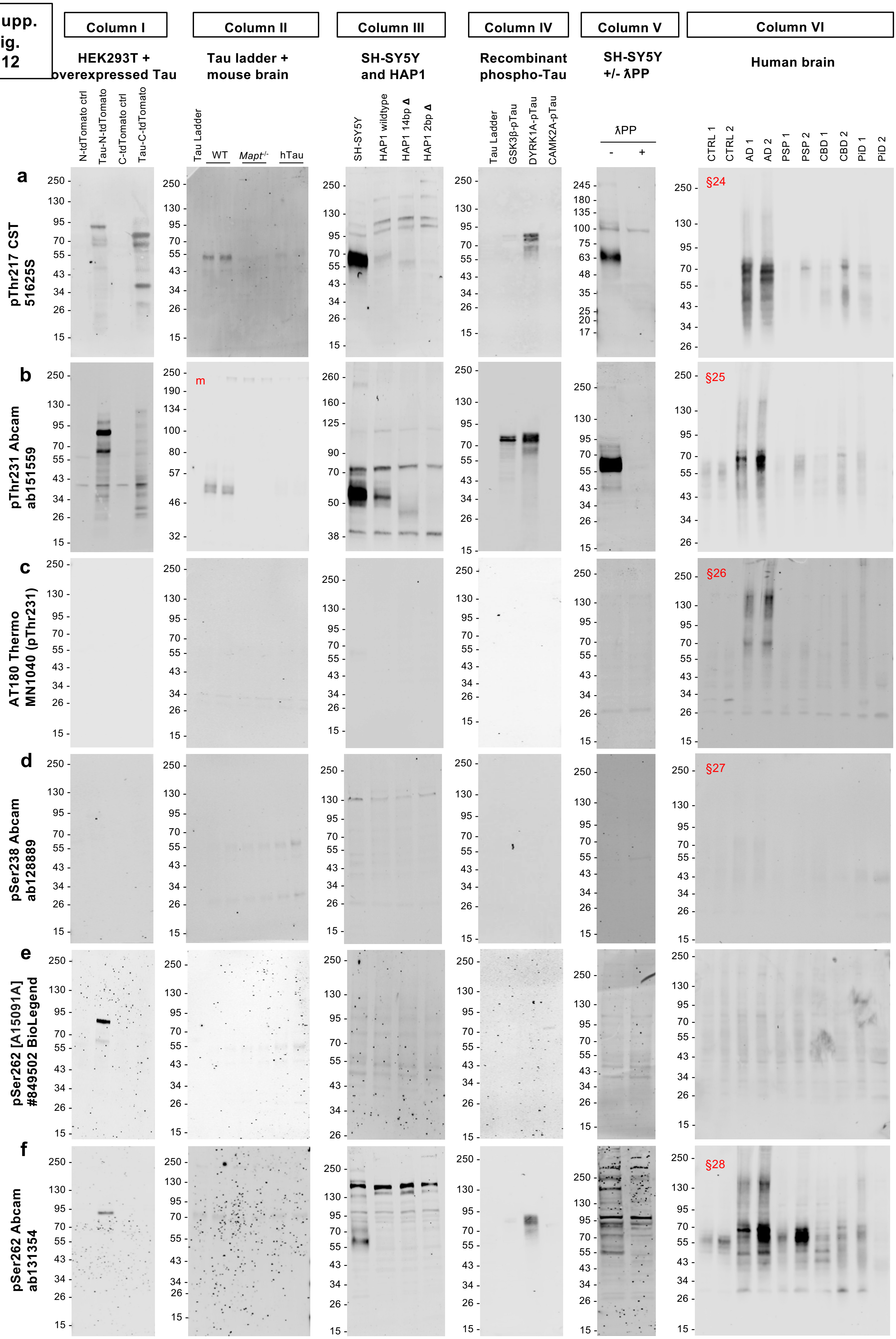

Column I
HEK293T + overexpressed Tau
N-tdTomato ctrl
Tau-N-tdTomato
C-tdTomato ctrl
Tau-C-tdTomato
Column II
Tau ladder + mouse brain
Tau Ladder
WT
Mapt-/-
hTau
Column III
SH-SY5Y and HAP1
SH-SY5Y
HAP1 wildtype
HAP1 14bp Δ
HAP1 2bp Δ
Column IV
Recombinant phospho-Tau
Tau Ladder
GSK3β-pTau
DYRK1A-pTau
CAMK2A-pTau
Column V
SH-SY5Y +/- λPP
λPP
-
+
Column VI
Human brain
CTRL 1
CTRL 2
AD 1
AD 2
PSP 1
PSP 2
CBD 1
CBD 2
PiD 1
PiD 2
a
pThr217 CST 51625S
§24
b
pThr231 Abcam ab151559
m
§25
c
AT180 Thermo MN1040 (pThr231)
§26
d
pSer238 Abcam ab128889
§27
e
pSer262 [A15091A] #849502 BioLegend
f
pSer262 Abcam ab131354
§28

Supplementary Figure S12. Validation of phospho-Tau antibodies by WB (part 3). a-f: WB membranes shown in each panel (**row**) were probed with a different Tau antibody: pThr217 Cell Signalling 51625S (**a**); pThr231 Abcam ab151559 (**b**); AT180 ThermoFisher Scientific MN1040 (**c**); pSer238 Abcam ab128889 (**d**); pSer262 BioLegend 849502 (**e**); pSer262 Abcam ab131354 (**f**).

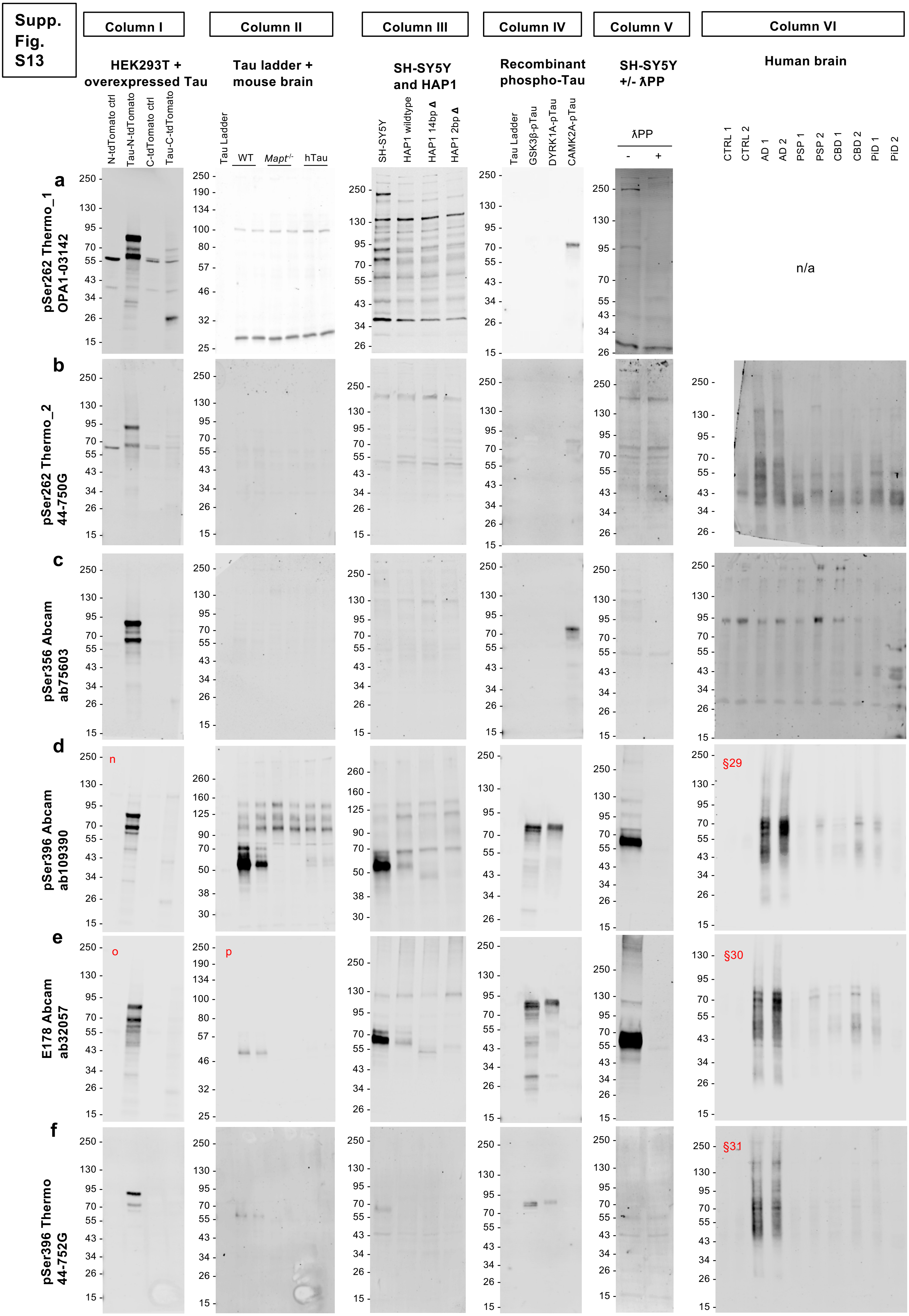

Supp. Fig. S13
Column I
Column II
Column III
Column IV
Column V
Column VI
HEK293T + overexpressed Tau
Tau ladder + mouse brain
SH-SY5Y and HAP1
Recombinant phospho-Tau
SH-SY5Y +/- λPP
Human brain
N-tdTomato ctrl
Tau-N-tdTomato
C-tdTomato ctrl
Tau-C-tdTomato
Tau Ladder
WT
Mapt-/-
hTau
SH-SY5Y
HAP1 wildtype
HAP1 14bp Δ
HAP1 2bp Δ
Tau Ladder
GSK3β-pTau
DYRK1A-pTau
CAMK2A-pTau
λPP
-
+
CTRL 1
CTRL 2
AD 1
AD 2
PSP 1
PSP 2
CBD 1
CBD 2
PiD 1
PiD 2
a
pSer262 Thermo_1 OPA1-03142
n/a
b
pSer262 Thermo_2 44-750G
c
pSer356 Abcam ab75603
d
pSer396 Abcam ab109390
n
§29
e
E178 Abcam ab32057
o
p
§30
f
pSer396 Thermo 44-752G
§31

Supplementary Figure S13. Validation of phospho-Tau antibodies (part 4) by WB. a-f: WB membranes shown in each panel (**row**) were probed with a different Tau antibody: pSer262 ThermoFisher Scientific OPA1-03142 (**a**); pSer262 ThermoFisher Scientific 44-750G (**b**); pSer356 Abcam ab75603 (**c**); pSer396 Abcam ab109390 (**d**); E178 (pSer396) Abcam ab32057 (**e**); pSer396 ThermoFisher Scientific 44-752G (**f**).

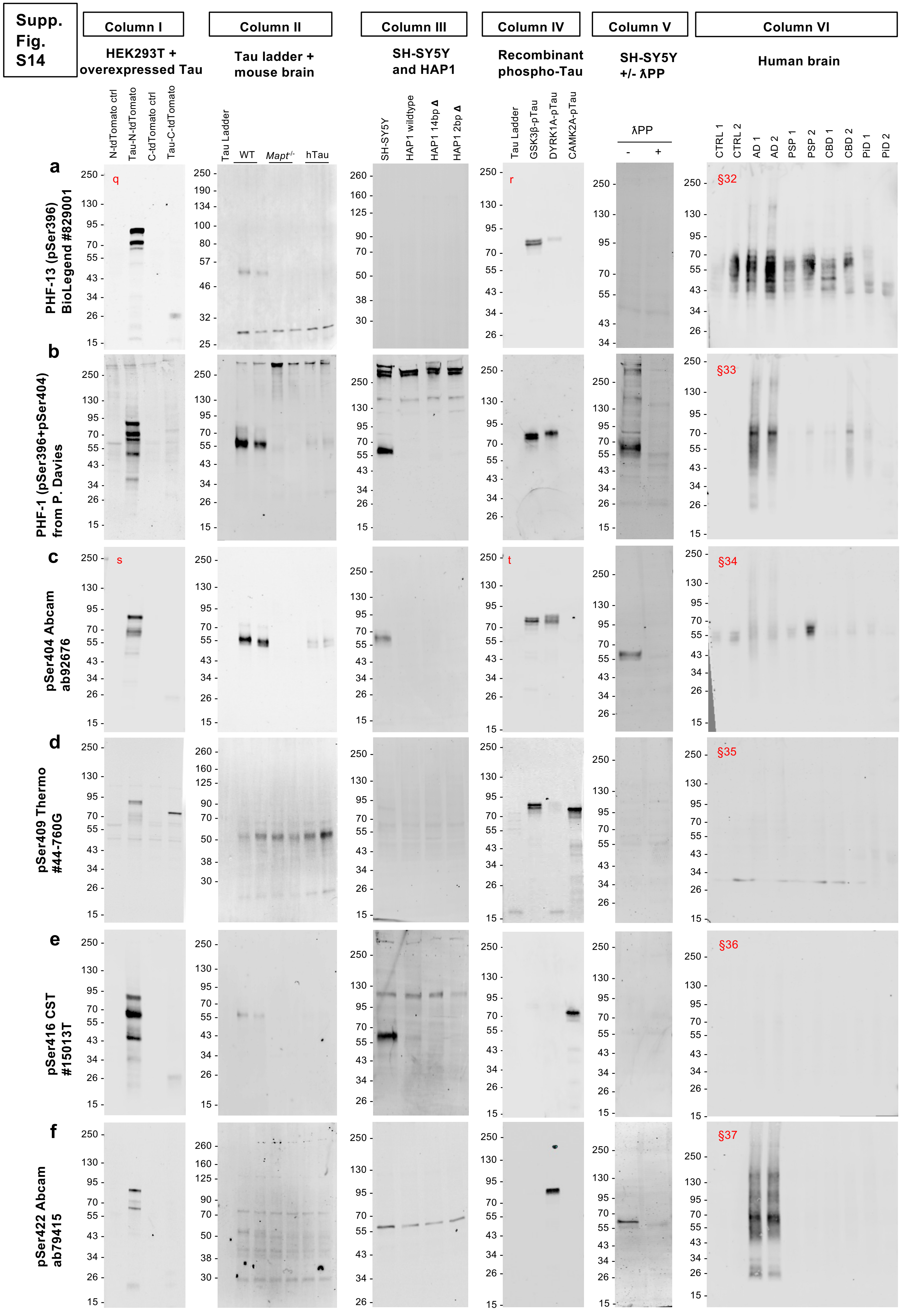
Supp. Fig. S14
Column I
HEK293T + overexpressed Tau
Column II
Tau ladder + mouse brain
Column III
SH-SY5Y and HAP1
Column IV
Recombinant phospho-Tau
Column V
SH-SY5Y +/- λPP
Column VI
Human brain
N-tdTomato ctrl
Tau-N-tdTomato
C-tdTomato ctrl
Tau-C-tdTomato
Tau Ladder
WT
Mapt-/-
hTau
SH-SY5Y
HAP1 wildtype
HAP1 14bp Δ
HAP1 2bp Δ
Tau Ladder
GSK3β-pTau
DYRK1A-pTau
CAMK2A-pTau
λPP
-
+
CTRL 1
CTRL 2
AD 1
AD 2
PSP 1
PSP 2
CBD 1
CBD 2
PiD 1
PiD 2
a
PHF-13 (pSer396) BioLegend #829001
q
r
§32
b
PHF-1 (pSer396+pSer404) from P. Davies
§33
c
pSer404 Abcam ab92676
s
t
§34
d
pSer409 Thermo #44-760G
§35
e
pSer416 CST #15013T
§36
f
pSer422 Abcam ab79415
§37

Supplementary Figure S14. Validation of phospho-Tau antibodies (part 5) by WB. a-f: WB membranes shown in each panel (**row**) were probed with a different Tau antibody: PHF-13 (pSer396) BioLegend 829001 (**a**); PHF-1 (pSer396+pSer404) from P. Davies (**b**); pSer404 Abcam ab92676 (**c**); pSer409 ThermoFisher Scientific 44-760G (**d**); pSer416 Cell Signalling 15013T (**e**); pSer422 Abcam ab79415 (**f**).

Supp. Fig. S15

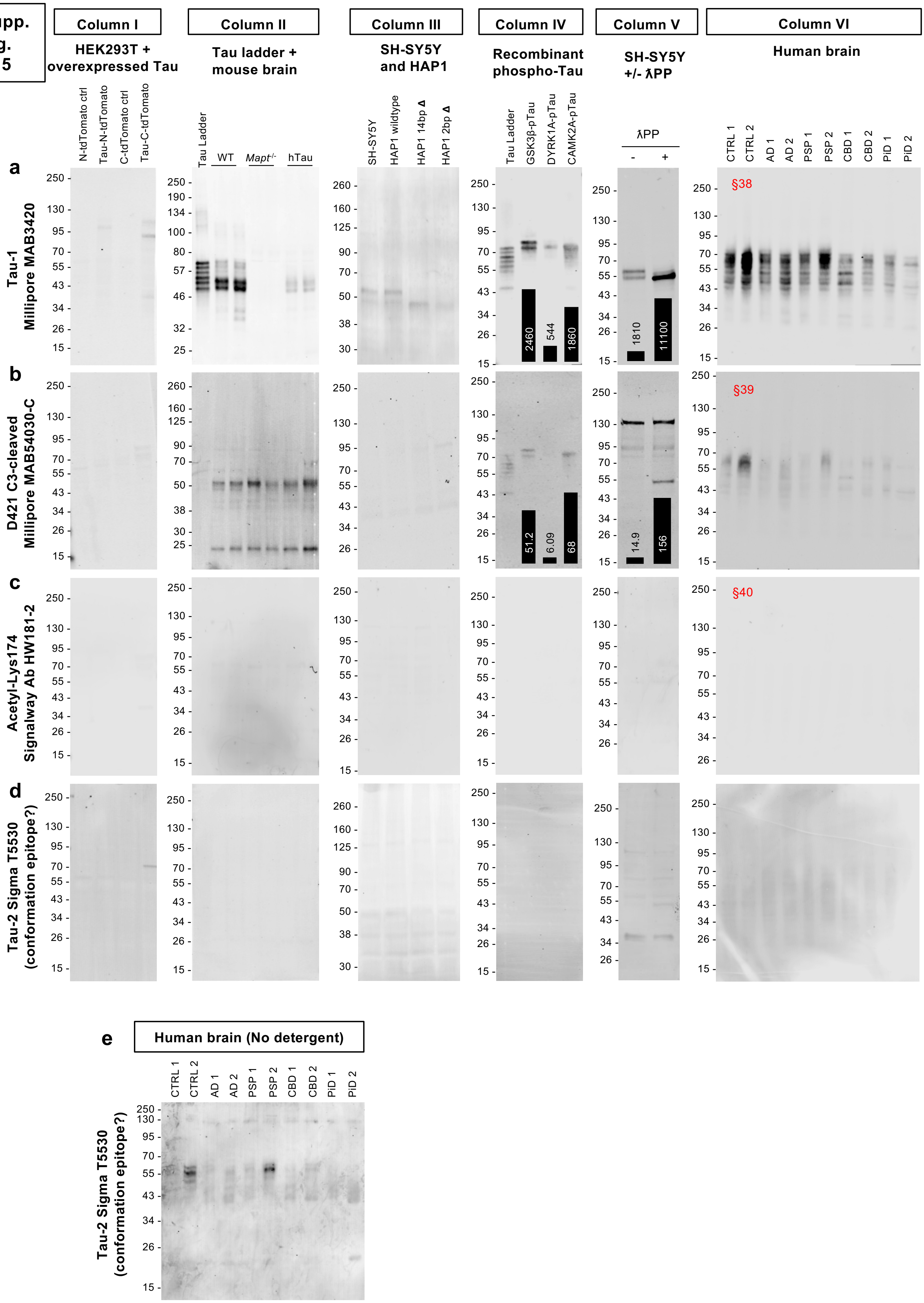

Column I
HEK293T + overexpressed Tau
N-tdTomato ctrl
Tau-N-tdTomato
C-tdTomato ctrl
Tau-C-tdTomato
Column II
Tau ladder + mouse brain
Tau Ladder
WT
Mapt-/-
hTau
Column III
SH-SY5Y and HAP1
SH-SY5Y
HAP1 wildtype
HAP1 14bp Δ
HAP1 2bp Δ
Column IV
Recombinant phospho-Tau
Tau Ladder
GSK3β-pTau
DYRK1A-pTau
CAMK2A-pTau
Column V
SH-SY5Y +/- λPP
λPP
-
+
Column VI
Human brain
CTRL 1
CTRL 2
AD 1
AD 2
PSP 1
PSP 2
CBD 1
CBD 2
PiD 1
PiD 2
a
Tau-1
Millipore MAB3420
2460
544
1860
1810
11100
§38
b
D421 C3-cleaved
Millipore MAB54030-C
51.2
6.09
68
14.9
156
§39
c
Acetyl-Lys174
Signalway Ab HW181-2
§40
d
Tau-2 Sigma T5530
(conformation epitope?)
e
Human brain (No detergent)
Tau-2 Sigma T5530
(conformation epitope?)

Supplementary Figure S15. Validation of other PTM-dependent antibodies by WB. a-d: WB membranes shown in each panel (**row**) were probed with a different Tau antibody: Tau-1 [clone PC1C6] Merck Millipore MAB3420 (**a**); D421 Tau-C3 Merck Millipore MAB54030-C (**b**); Acetyl-Lys174 Signalway Antibody HW181-2 (**c**); Tau-2 Sigma T5530 (**d**). **e:** WB membrane of human brain protein extracts probed with the Tau-2 (Sigma, cat. no T5530) antibody where detergent has been omitted from all buffers (i.e. block, antibody incubation and wash buffers).

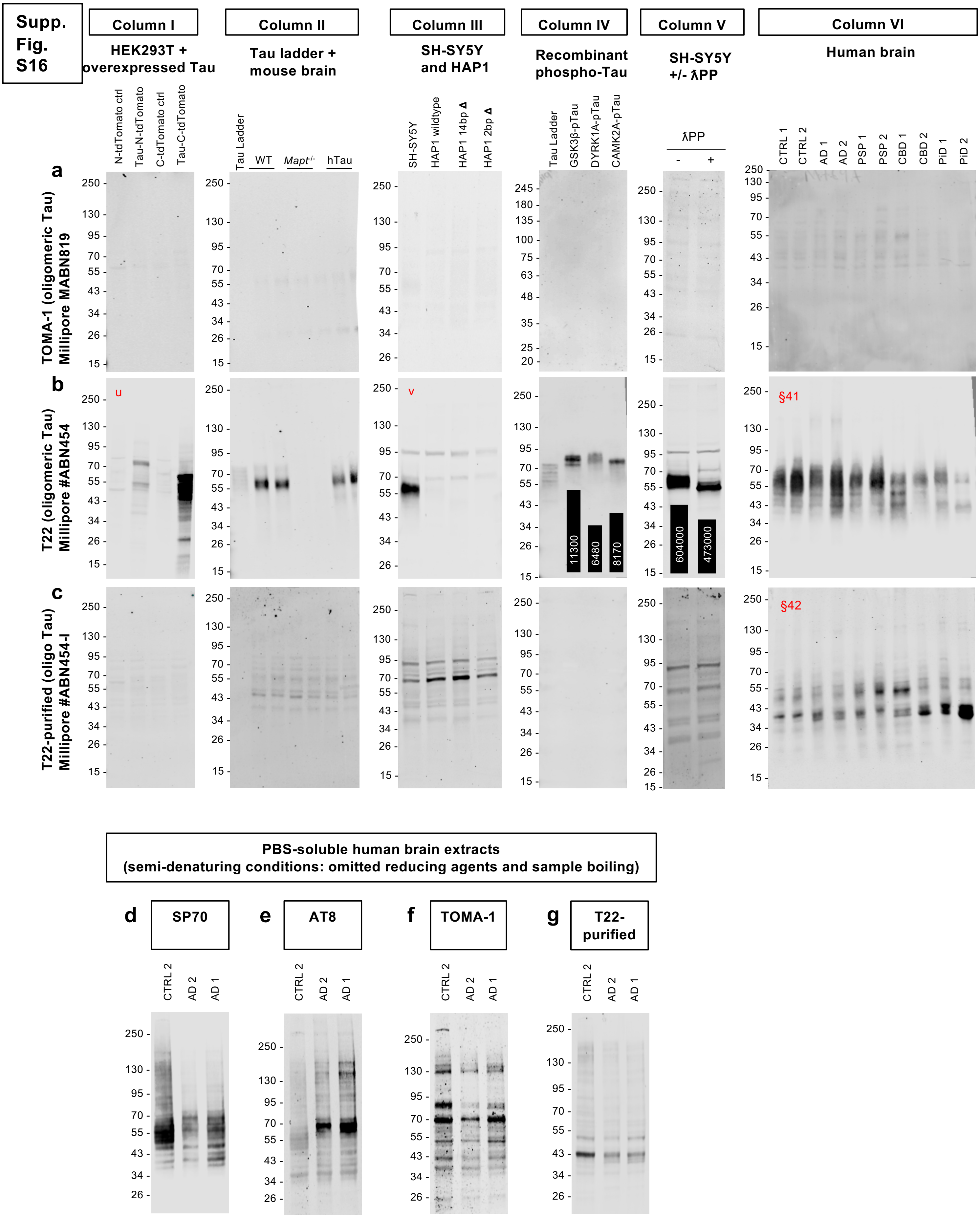
Supp. Fig. S16
Column I
HEK293T + overexpressed Tau
N-tdTomato ctrl
Tau-N-tdTomato
C-tdTomato ctrl
Tau-C-tdTomato
Column II
Tau ladder + mouse brain
Tau Ladder
WT
Mapt-/-
hTau
Column III
SH-SY5Y and HAP1
SH-SY5Y
HAP1 wildtype
HAP1 14bp Δ
HAP1 2bp Δ
Column IV
Recombinant phospho-Tau
Tau Ladder
GSK3β-pTau
DYRK1A-pTau
CAMK2A-pTau
Column V
SH-SY5Y +/- λPP
λPP
-
+
Column VI
Human brain
CTRL 1
CTRL 2
AD 1
AD 2
PSP 1
PSP 2
CBD 1
CBD 2
PiD 1
PiD 2
a
TOMA-1 (oligomeric Tau) Millipore MABN819
b
T22 (oligomeric Tau) Millipore #ABN454
u
v
§41
11300
6480
8170
604000
473000
c
T22-purified (oligo Tau) Millipore #ABN454-I
§42
250
130
95
70
55
43
34
26
15
245
180
135
100
75
63
48
35
25
20
PBS-soluble human brain extracts
(semi-denaturing conditions: omitted reducing agents and sample boiling)
d
SP70
e
AT8
f
TOMA-1
g
T22-purified
CTRL 2
AD 2
AD 1

Supplementary Figure S16. Validation of “oligomeric Tau” antibodies by WB. a-c: WB membranes shown in each panel (**row**) were probed with a different Tau antibody: TOMA-1 Merck Millipore MABN819 (**a**); T22 Merck Millipore ABN454 (**b**); T22 "purified" Merck Millipore ABN454-I (**c**). **d-g:** PBS-soluble proteins extracted from human brain samples were separated by SDS-PAGE under semi-denaturing conditions (reducing agents and boiling of the samples were omitted). WB membranes were probed with: SP70 “total” Tau antibody (**d**), AT8 phospho-Tau antibody (**e**), TOMA-1 antibody (**f**), T22-purified antibody (**g**).

Supp. Fig. S17

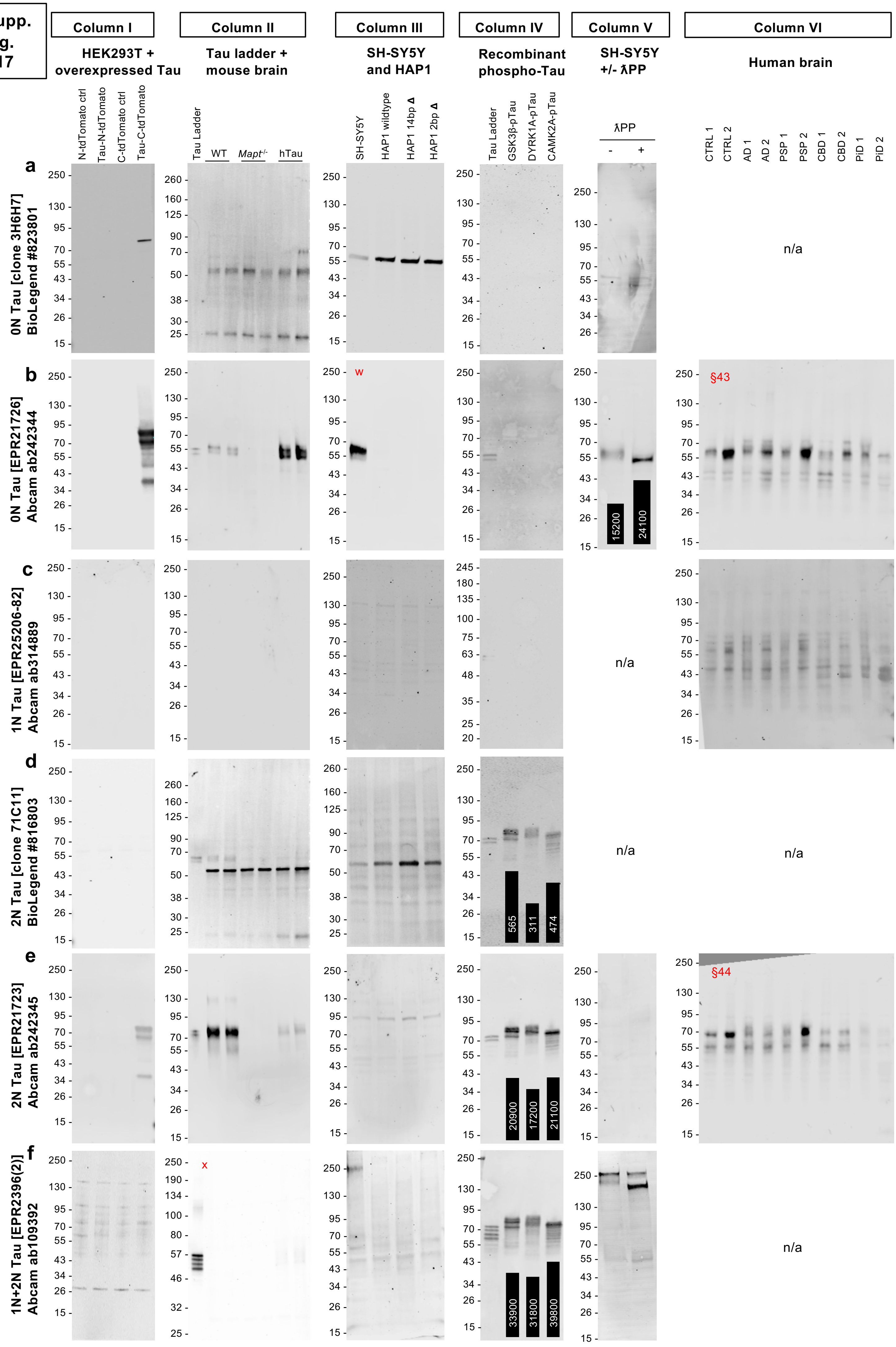

Column I
HEK293T + overexpressed Tau
Column II
Tau ladder + mouse brain
Column III
SH-SY5Y and HAP1
Column IV
Recombinant phospho-Tau
Column V
SH-SY5Y +/- λPP
Column VI
Human brain
N-tdTomato ctrl
Tau-N-tdTomato
C-tdTomato ctrl
Tau-C-tdTomato
Tau Ladder
WT
Mapt-/-
hTau
SH-SY5Y
HAP1 wildtype
HAP1 14bp Δ
HAP1 2bp Δ
Tau Ladder
GSK3β-pTau
DYRK1A-pTau
CAMK2A-pTau
λPP
- +
CTRL 1
CTRL 2
AD 1
AD 2
PSP 1
PSP 2
CBD 1
CBD 2
PiD 1
PiD 2
a
0N Tau [clone 3H6H7] BioLegend #823801
n/a
b
0N Tau [EPR21726] Abcam ab242344
w
15200
24100
§43
c
1N Tau [EPR25206-82] Abcam ab314889
n/a
d
2N Tau [clone 71C11] BioLegend #816803
565
311
474
n/a
n/a
e
2N Tau [EPR21723] Abcam ab242345
20900
17200
21100
§44
f
1N+2N Tau [EPR2396(2)] Abcam ab109392
x
33900
31800
39800
n/a

Supplementary Figure S17. Validation of isoform-specific Tau antibodies by WB (part 1). a-f: WB membranes shown in each panel (**row**) were probed with a different Tau antibody: 0N Tau BioLegend 823801 (**a**); 0N Tau Abcam ab242344 (**b**); 1N Tau Abcam ab314889 (**c**); 2N Tau BioLegend 816803 (**d**); 2N Tau Abcam ab242345 (**e**); 1N Tau Abcam ab109392 (**f**).

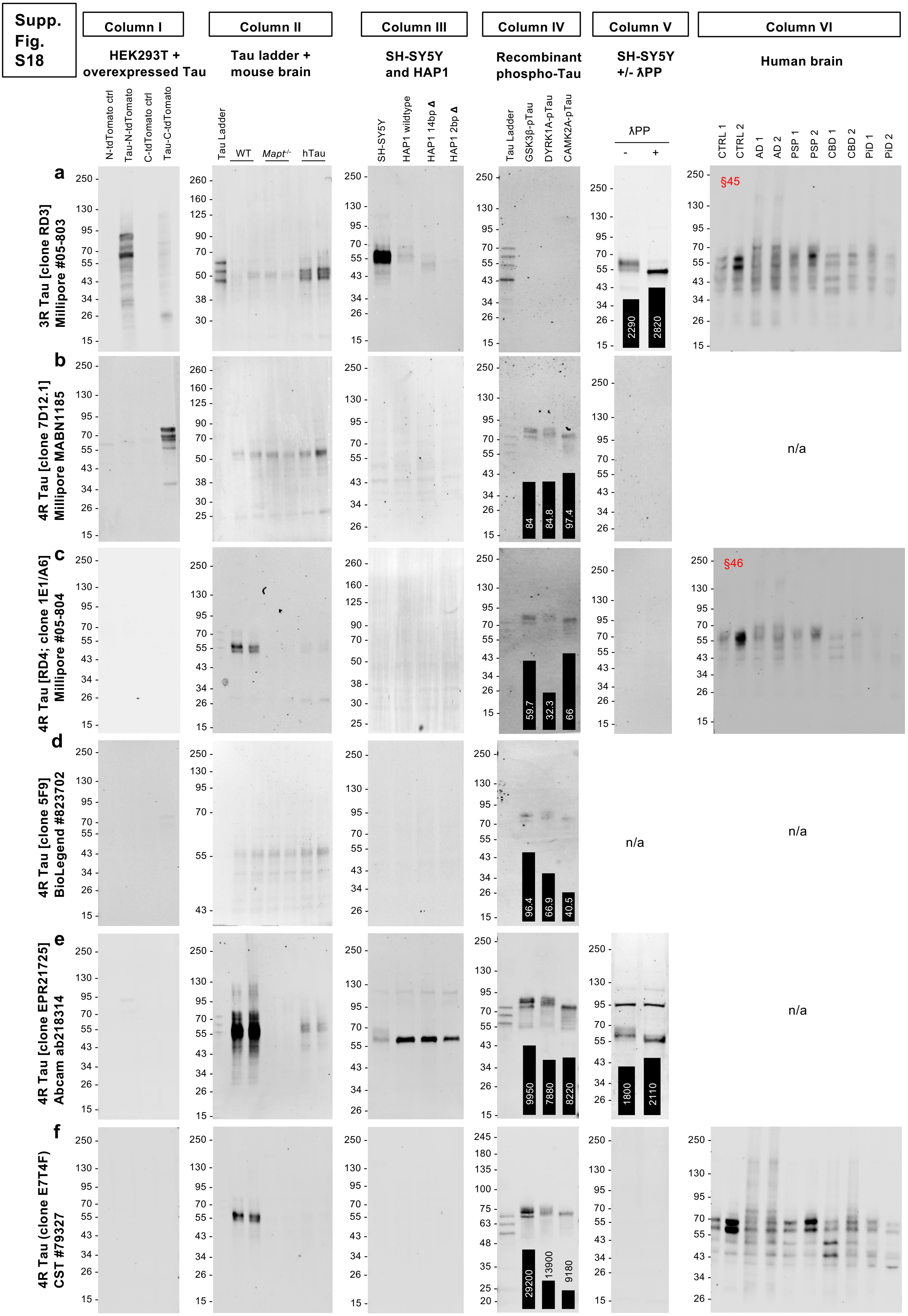
Supp. Fig. S18
Column I
HEK293T + overexpressed Tau
N-tdTomato ctrl
Tau-N-tdTomato
C-tdTomato ctrl
Tau-C-tdTomato
Column II
Tau ladder + mouse brain
Tau Ladder
WT
Mapt-/-
hTau
Column III
SH-SY5Y and HAP1
SH-SY5Y
HAP1 wildtype
HAP1 14bp Δ
HAP1 2bp Δ
Column IV
Recombinant phospho-Tau
Tau Ladder
GSK3β-pTau
DYRK1A-pTau
CAMK2A-pTau
Column V
SH-SY5Y +/- λPP
λPP
-
+
Column VI
Human brain
CTRL 1
CTRL 2
AD 1
AD 2
PSP 1
PSP 2
CBD 1
CBD 2
PiD 1
PiD 2
a
3R Tau [clone RD3] Millipore #05-803
§45
2290
2820
b
4R Tau [clone 7D12.1] Millipore MABN1185
84
84.8
97.4
n/a
c
4R Tau [RD4; clone 1E1/A6] Millipore #05-804
59.7
32.3
66
§46
d
4R Tau [clone 5F9] BioLegend #823702
96.4
66.9
40.5
n/a
n/a
e
4R Tau [clone EPR21725] Abcam ab218314
9950
7880
8220
1800
2110
n/a
f
4R Tau (clone E7T4F) CST #79327
29200
13900
9180

Supplementary Figure S18. Validation of isoform-specific Tau antibodies by WB (part 2). a-f: WB membranes shown in each panel (**row**) were probed with a different Tau antibody: RD3 (3R Tau) Merck Millipore 05-803 (**a**); 4R Tau Merck Millipore MABN1185 (**b**); RD4 4R Tau Merck Millipore 05-804 (**c**); 4R BioLegend 823702 (**d**); 4R Abcam ab218314 (**e**); 4R Cell Signalling 79327 (**f**).

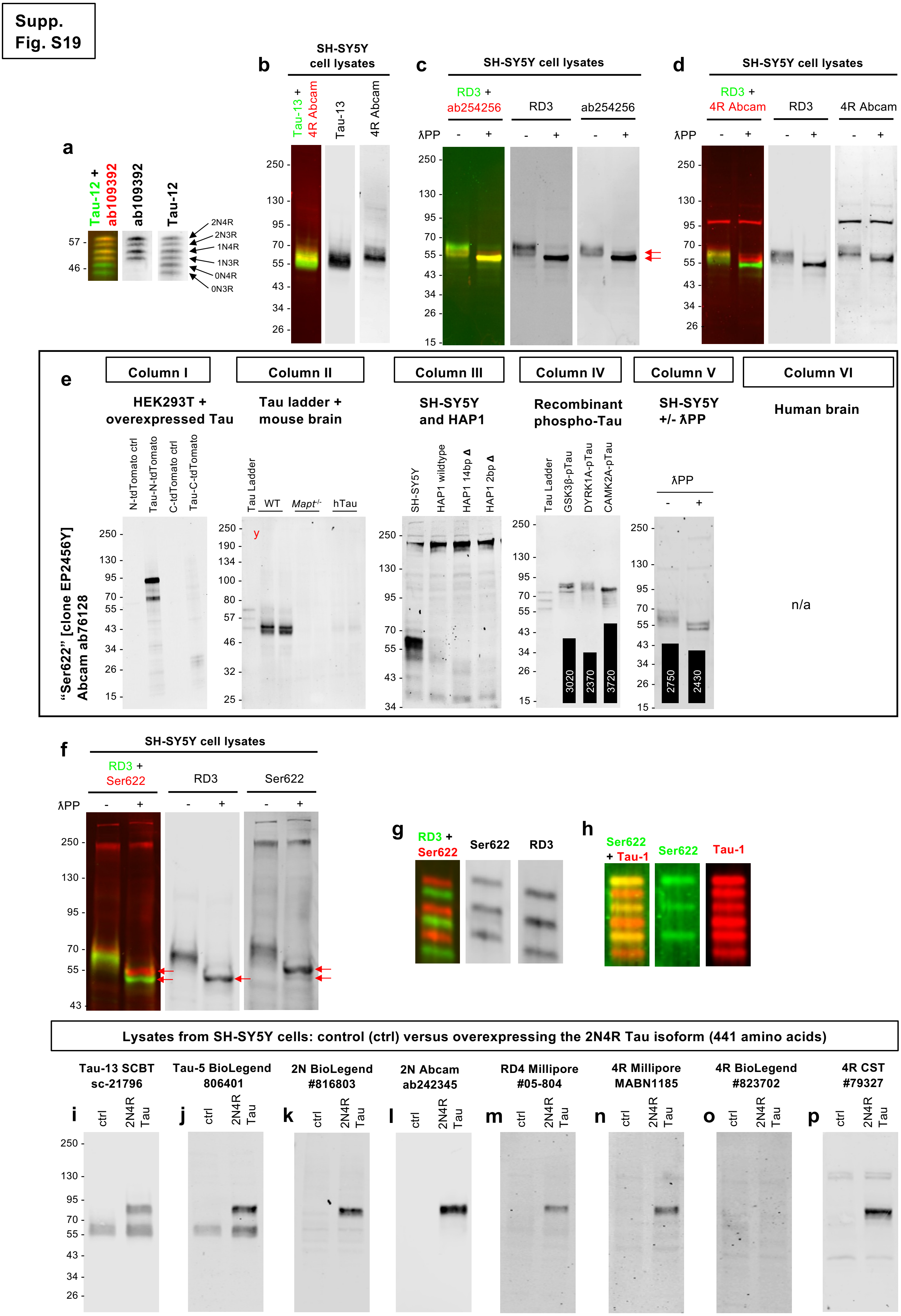
Supp. Fig. S19
a
Tau-12 + ab109392
ab109392
Tau-12
2N4R
2N3R
1N4R
1N3R
0N4R
0N3R
b
SH-SY5Y cell lysates
Tau-13 + 4R Abcam
Tau-13
4R Abcam
c
SH-SY5Y cell lysates
RD3 + ab254256
RD3
ab254256
λPP
d
SH-SY5Y cell lysates
RD3 + 4R Abcam
RD3
4R Abcam
e
Column I
HEK293T + overexpressed Tau
N-tdTomato ctrl
Tau-N-tdTomato
C-tdTomato ctrl
Tau-C-tdTomato
Column II
Tau ladder + mouse brain
Tau Ladder
WT
Mapt-/-
hTau
Column III
SH-SY5Y and HAP1
SH-SY5Y
HAP1 wildtype
HAP1 14bp Δ
HAP1 2bp Δ
Column IV
Recombinant phospho-Tau
Tau Ladder
GSK3β-pTau
DYRK1A-pTau
CAMK2A-pTau
3020
2370
3720
Column V
SH-SY5Y +/- λPP
λPP
2750
2430
Column VI
Human brain
n/a
"Ser622" [clone EP2456Y] Abcam ab76128
f
SH-SY5Y cell lysates
RD3 + Ser622
RD3
Ser622
λPP
g
RD3 + Ser622
Ser622
RD3
h
Ser622 + Tau-1
Ser622
Tau-1
Lysates from SH-SY5Y cells: control (ctrl) versus overexpressing the 2N4R Tau isoform (441 amino acids)
i Tau-13 SCBT sc-21796
j Tau-5 BioLegend 806401
k 2N BioLegend #816803
l 2N Abcam ab242345
m RD4 Millipore #05-804
n 4R Millipore MABN1185
o 4R BioLegend #823702
p 4R CST #79327
ctrl
2N4R Tau

Supplementary Figure S19. Validation of the "Ser622" Tau antibody and additional data for the validation of isoform-specific Tau antibodies by WB (related to Supp. Figs. S17 and S18). **a**: Recombinant Tau ladder was detected with the Tau-12 "total" Tau antibody and the #ab109392 antibody, which only detects four of the six Tau isoforms that make up the ladder. The bands detected by #ab109392 correspond to 1N and 2N splice isoforms. **b**-**d**: Fluorescent WB membranes of SH-SY5Y cell lysates, that were either untreated (panel **b**, marked with "-" in panels **c** and **d**) or treated (marked with "+" in panels **c** and **d**) with λPP, were co-probed with different combinations of mouse and rabbit monoclonal Tau antibodies: Tau-13 "total" Tau antibody + 4R Abcam Tau antibody (**b**), RD3 3R Tau antibody + #ab254256 "total" Tau antibody (**c**), RD3 3R Tau antibody + 4R Abcam Tau antibody (**d**). **e**: WB validation data for the "Ser622" antibody (Abcam, ab76128). **f**: Fluorescent WB membrane of SH-SY5Y cell lysates, that were either untreated ("-") or treated ("+") with λPP, co-probed with "Ser622" (red) and RD3 (green) antibodies shows that the main "Ser622"-immunoreactive band is a Tau isoform of a higher MW compared to that detected by RD3. Red arrows in panels **c** and **f** indicate the Tau bands detected in λPP-treated SH-SY5Y lysates that correspond to 3R (bottom band) and 4R (top band) Tau isoforms, respectively. **g-h**: WBs of recombinant human Tau ladder were co-probed with the Ser622 antibody (red in **g**, green in **h**) and either the RD3 3R Tau antibody clone (**g**, green) or the Tau-1 antibody clone (**h**, red). **i-p**: WBs of lysates from SH-SY5Y cells overexpressing 2N4R human Tau or control (ctrl) cells were probed with different Tau antibodies: clone Tau-13 Santa Cruz Biotechnology #sc-21796 (**i**), clone Tau-5 BioLegend #806401 (**j**), clone 71C11 (2N Tau) BioLegend #816803 (**k**), 2N Abcam #ab242345 (**l**), clone RD4 Millipore #05-804 (**m**), clone 7D12.1 (4R Tau) Millipore #MABN1185 (**n**), clone 5F9 (4R Tau) BioLegend #823702 (**o**), clone E7T4F (4R Tau) CST #79327 (**p**).

Supp. Fig. S20

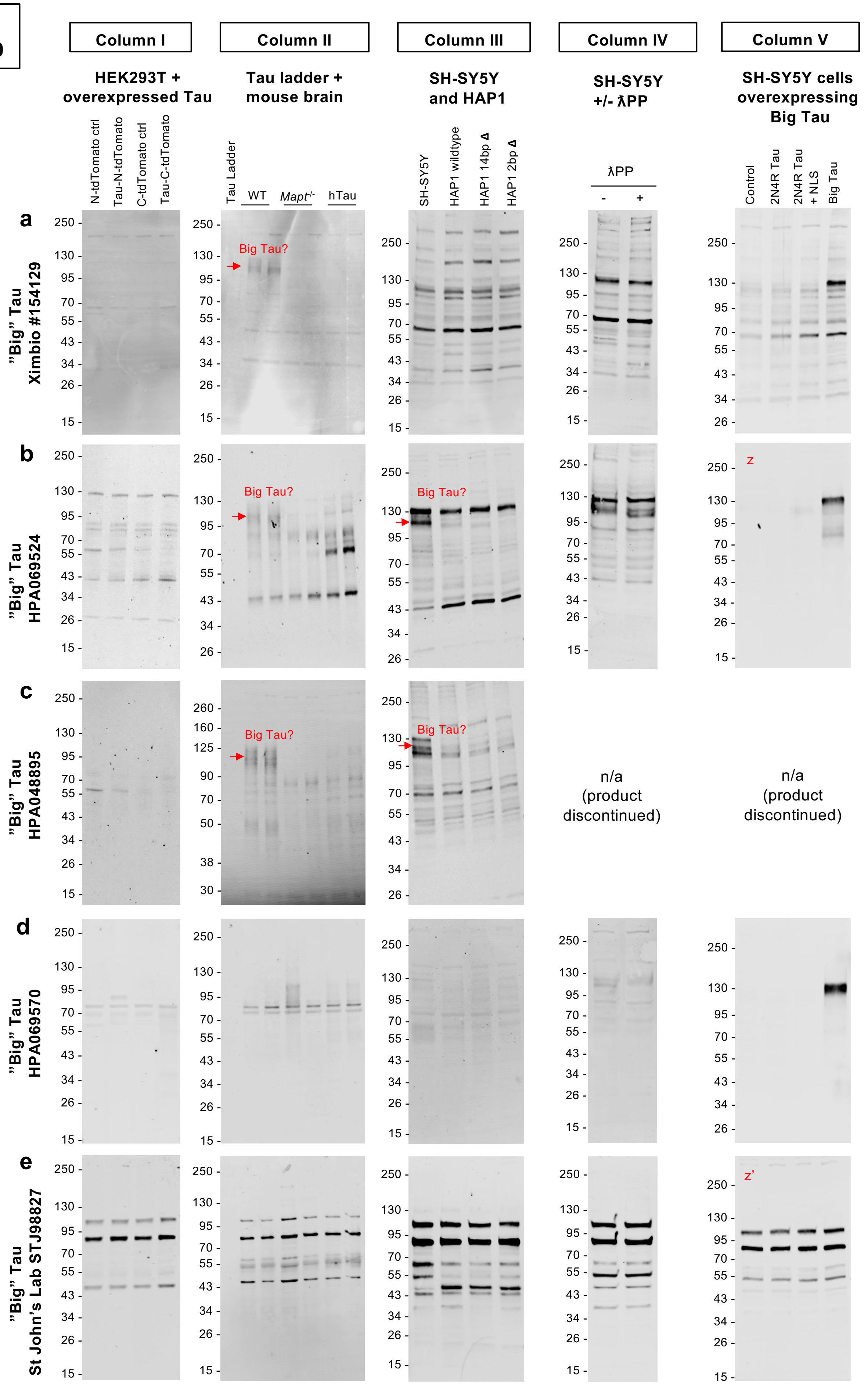
Column I
HEK293T + overexpressed Tau
N-tdTomato ctrl
Tau-N-tdTomato
C-tdTomato ctrl
Tau-C-tdTomato
Column II
Tau ladder + mouse brain
Tau Ladder
WT
Mapt-/-
hTau
Column III
SH-SY5Y and HAP1
SH-SY5Y
HAP1 wildtype
HAP1 14bp Δ
HAP1 2bp Δ
Column IV
SH-SY5Y +/- λPP
λPP
-
+
Column V
SH-SY5Y cells overexpressing Big Tau
Control
2N4R Tau
2N4R Tau + NLS
Big Tau
a
"Big" Tau Ximbio #154129
b
"Big" Tau HPA069524
c
"Big" Tau HPA048895
d
"Big" Tau HPA069570
e
"Big" Tau St John's Lab STJ98827
Big Tau?
z
z'
n/a (product discontinued)
n/a (product discontinued)

Supplementary Figure S20. Validation of Big Tau antibodies by WB. a-e: WBs shown in columns I to V are as follows: WB of lysates from HEK293T cells overexpressing 0N3R human Tau and corresponding control cells (**column I**); WB of recombinant human Tau ladder (5 ng/isoform/lane), plus adult mouse brain lysates from wildtype, *Mapt*$^{-/-}$ and hTau mice (**column II**); WB of lysates from SH-SY5Y neuroblastoma cells, plus HAP1 cells: parental (wildtype) and two cell lines carrying either a 14 bp deletion (14 bp Δ) or a 2-bp deletion (2 bp Δ) in *MAPT* exon 4 (**column III**); WB of lysates from SH-SY5Y neuroblastoma cells that have been either untreated (-) or treated (+) with λPP (**column IV**); and WB of lysates from SH-SY5Y cells overexpressing either 2N4R human Tau (+/- nuclear localisation signal, NLS) or human Big Tau and corresponding control cells (**column V**). WB membranes shown in each panel (**row**) were probed with a different Big Tau antibody: Ximbio #154129 (**a**), HPA069524 (**b**), HPA048895 (**c**), HPA069570 (**d**), St. John's Laboratories #STJ98827 (**e**).

Supp. Fig. S21

a **Human Tau (top) versus human MAP2 (bottom) amino acid sequences**

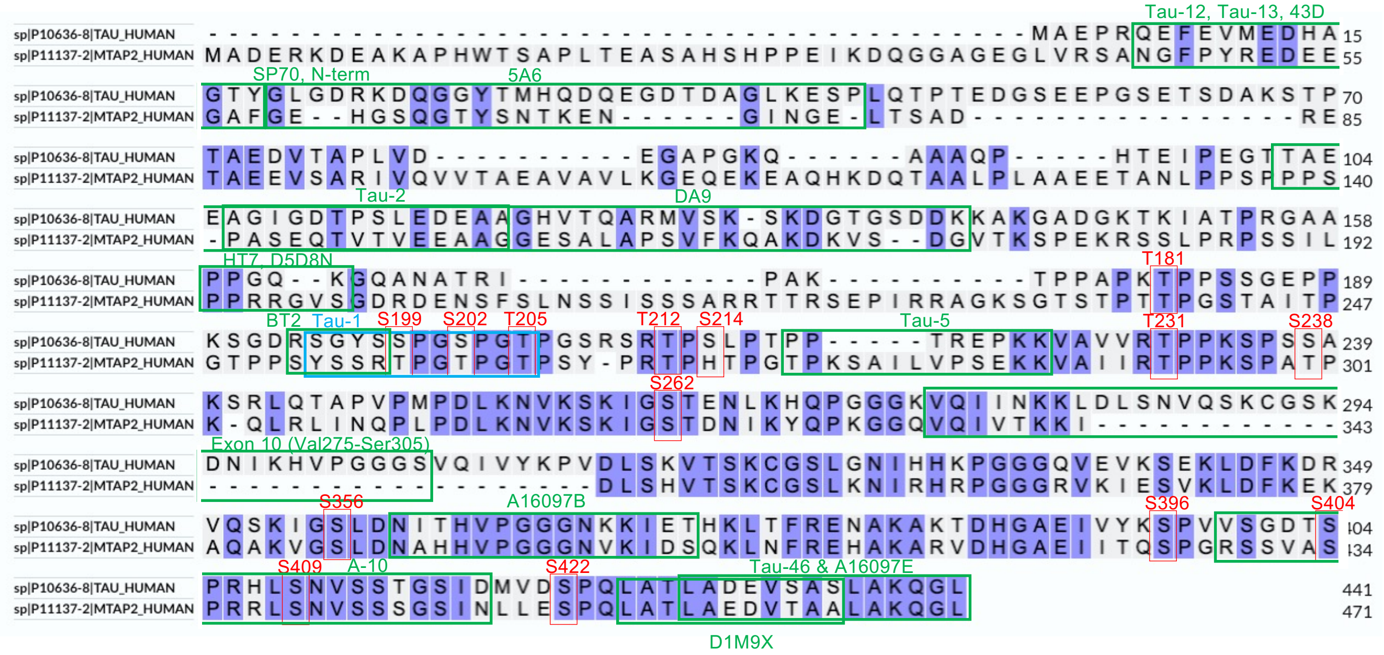

b **Human Tau (top) versus human MAP4 (bottom) amino acid sequences**

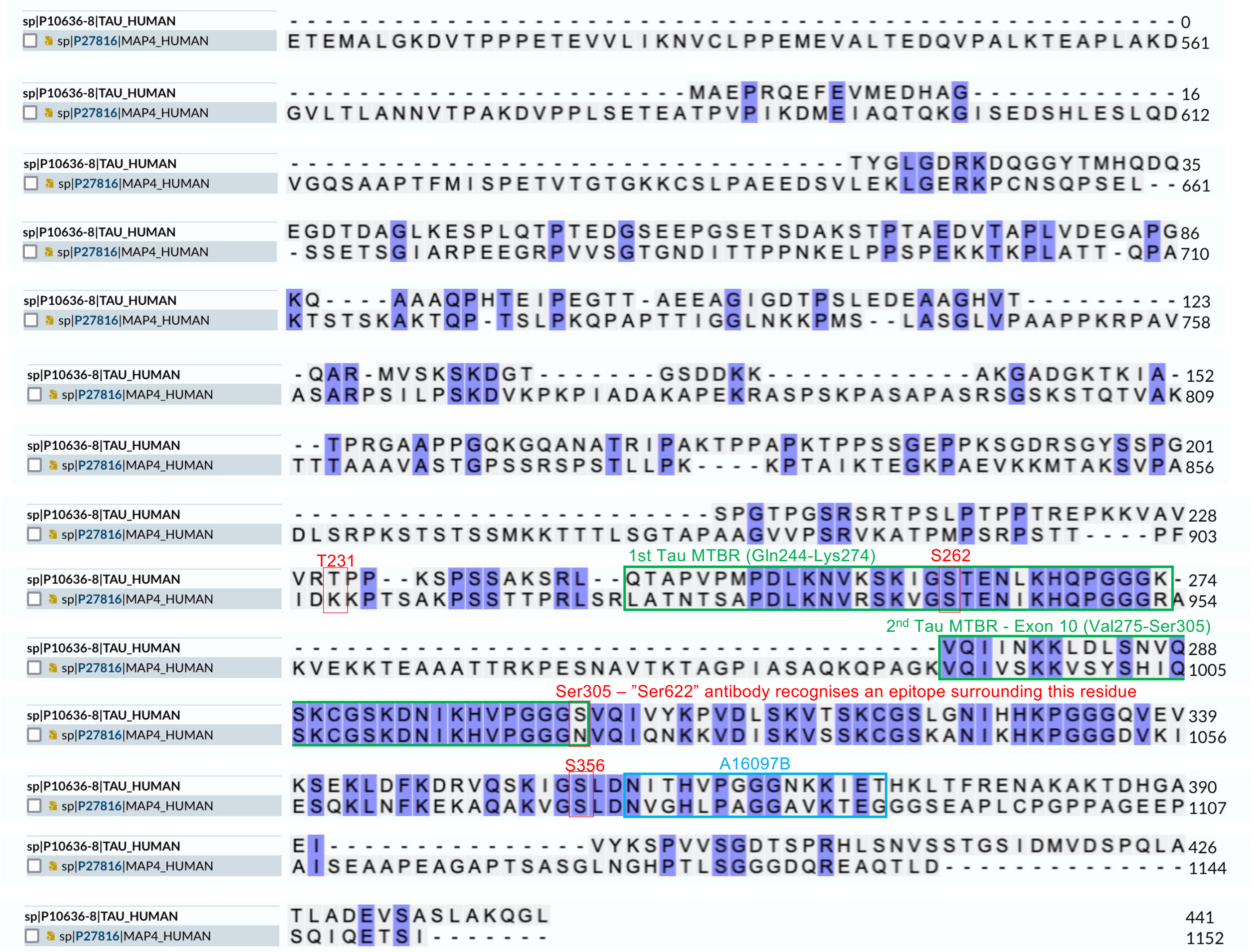

Supplementary Figure S21. Alignment of protein sequences highlights similarities between Tau and the related microtubule-associated proteins, MAP2 and MAP4. a: CLUSTAL Omega alignment of the 2N4R human Tau protein sequence (441 amino acids, Uniprot ID P10636-8) with the human MAP2c protein isoform sequence (471 amino acids, Uniprot ID P11137-2). Phosphorylateable residues targeted by different phospho-Tau antibodies are highlighted in red. The epitope regions of different “total” Tau antibodies are highlighted in green and that of Tau-1 in blue. **b**: CLUSTAL Omega alignment of the 2N4R human Tau protein sequence (441 amino acids, Uniprot ID P10636-8) with the human MAP4 protein sequence (1,152 amino acids, Uniprot ID P27816). Human Tau residues Thr231, Ser262 and Ser305 are highlighted in red. The sequences of the first and second Tau MTBRs (the latter is encoded by the alternatively-spliced exon 10 and is only present in 4R Tau isoforms) are highlighted in green. Epitope region of the A16079B “total” Tau antibody clone is highlighted in blue.

Supp. Fig. S22

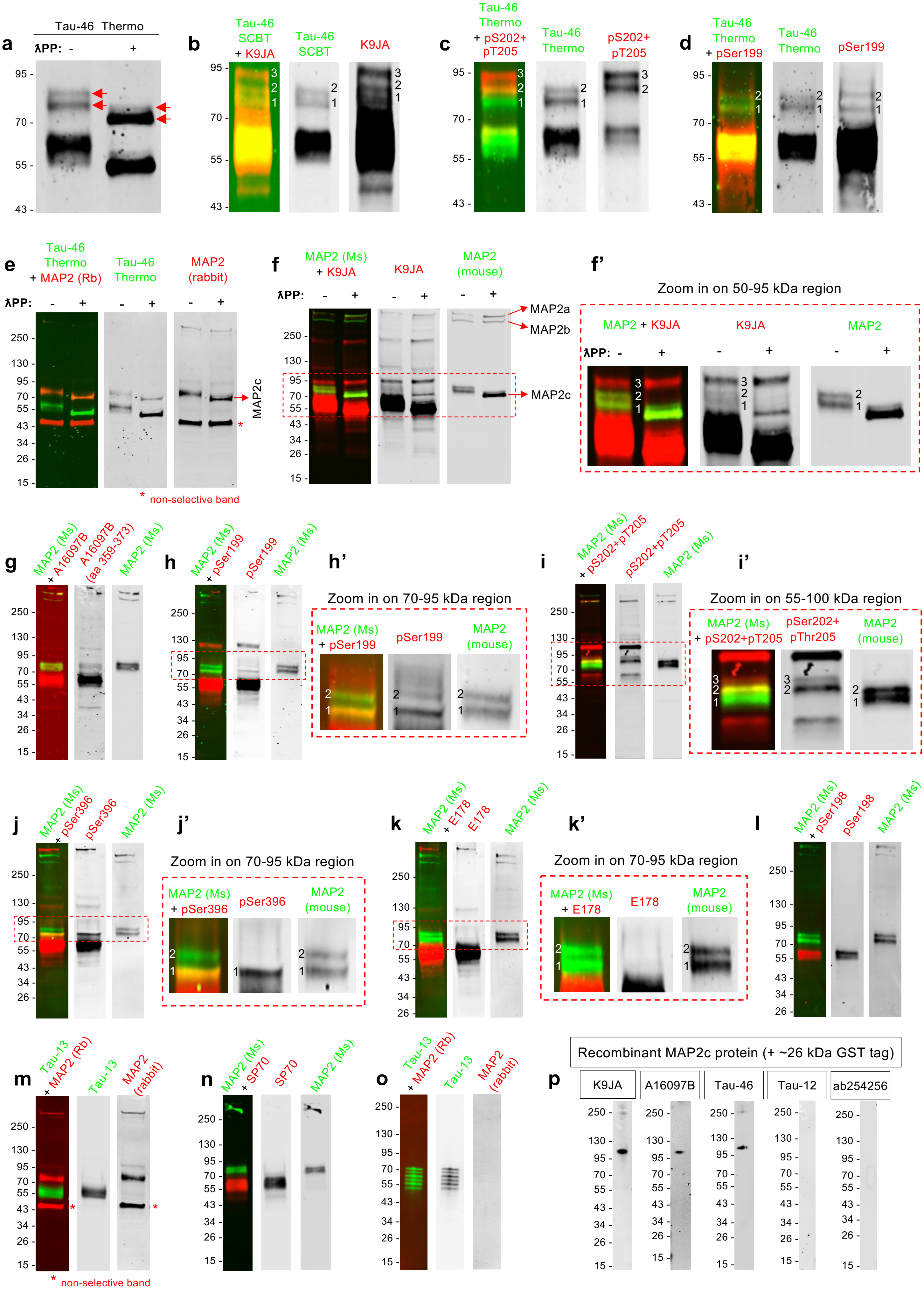

a
Tau-46 Thermo
λPP: - +
b
Tau-46 SCBT + K9JA
Tau-46 SCBT
K9JA
c
Tau-46 Thermo + pS202+pT205
Tau-46 Thermo
pS202+pT205
d
Tau-46 Thermo + pSer199
Tau-46 Thermo
pSer199
e
Tau-46 Thermo + MAP2 (Rb)
Tau-46 Thermo
MAP2 (rabbit)
MAP2c
* non-selective band
f
MAP2 (Ms) + K9JA
K9JA
MAP2 (mouse)
MAP2a
MAP2b
MAP2c
f'
Zoom in on 50-95 kDa region
MAP2 + K9JA
K9JA
MAP2
g
MAP2 (Ms) + A16097B
A16097B (aa 359-373)
MAP2 (Ms)
h
MAP2 (Ms) + pSer199
pSer199
MAP2 (Ms)
h'
Zoom in on 70-95 kDa region
MAP2 (Ms) + pSer199
pSer199
MAP2 (mouse)
i
MAP2 (Ms) + pS202+pT205
pS202+pT205
MAP2 (Ms)
i'
Zoom in on 55-100 kDa region
MAP2 (Ms) + pS202+pT205
pSer202+ pThr205
MAP2 (mouse)
j
MAP2 (Ms) + pSer396
pSer396
MAP2 (Ms)
j'
Zoom in on 70-95 kDa region
MAP2 (Ms) + pSer396
pSer396
MAP2 (mouse)
k
MAP2 (Ms) + E178
E178
MAP2 (Ms)
k'
Zoom in on 70-95 kDa region
MAP2 (Ms) + E178
E178
MAP2 (mouse)
l
MAP2 (Ms) + pSer198
pSer198
MAP2 (Ms)
m
Tau-13 + MAP2 (Rb)
Tau-13
MAP2 (rabbit)
* non-selective band
n
MAP2 (Ms) + SP70
SP70
MAP2 (Ms)
o
Tau-13 + MAP2 (Rb)
Tau-13
MAP2 (rabbit)
p
Recombinant MAP2c protein (+ ~26 kDa GST tag)
K9JA
A16097B
Tau-46
Tau-12
ab254256

Supplementary Figure S22. Several Tau antibodies cross-react with MAP2-immunoreactive bands on WB. Fluorescent WB membranes probed with different Tau and MAP2 antibodies. SH-SY5Y protein extracts were employed for all WBs shown in panels **a-n**. For panels **o** and **p**, recombinant proteins were used: recombinant Tau ladder (**o**) and recombinant GST-tagged MAP2c protein (**p**). **a**: Magnified view of the 43-100 kDa region of the SH-SY5Y +/-λPP WB membrane shown in Supp. Fig. S7d, to highlight mid-MW Tau-46-immunoreactive bands (red arrows). **b-e**: WB membranes co-probed with Tau-46 and either: K9JA (**b**), pSer202+pThr205 (**c**), pSer199 (**d**), or MAP2 (**e**) antibodies. **f-n**: WB membranes co-probed with a MAP2 antibody and one of several Tau antibodies: K9JA (**f, f'**), A16097B (**g**), pSer199 (**h, h'**), pSer202+pThr205 (**i, i'**), pSer396 (**j, j'**), E178 (**k, k'**), pSer198 (**l**), Tau-13 (**m**), or SP70 (**n**). **o**: WB membrane of recombinant Tau ladder co-probed with the Tau-13 and MAP2 antibodies, to demonstrate lack of reactivity of the MAP2 antibody with Tau. **p:** WB membranes of recombinant MAP2c (carrying a ~26 kDa GST tag; migrates on SDS-PAGE to an apparent MW of ~100 kDa) probed with one of five different Tau antibodies, as indicated (K9JA, A16097B, Tau-46, Tau-12 or ab254256) to directly test for cross-reactivity of Tau antibodies with the MAP2 protein. In panels a, e, f and f', protein lysates were either untreated (-) or treated (+) with λPP. Bands marked with an asterisk in panels e and m represent non-specific cross-reactivity of the rabbit MAP2 antibody. Magnified views of the regions outlined with red dashed line in panels f, h, i, j and k are provided in panels f', h', i', j' and k', respectively. Bands corresponding to different MAP2 protein isoforms are indicated with red arrows in panels e and f.

Supp. Fig. S23

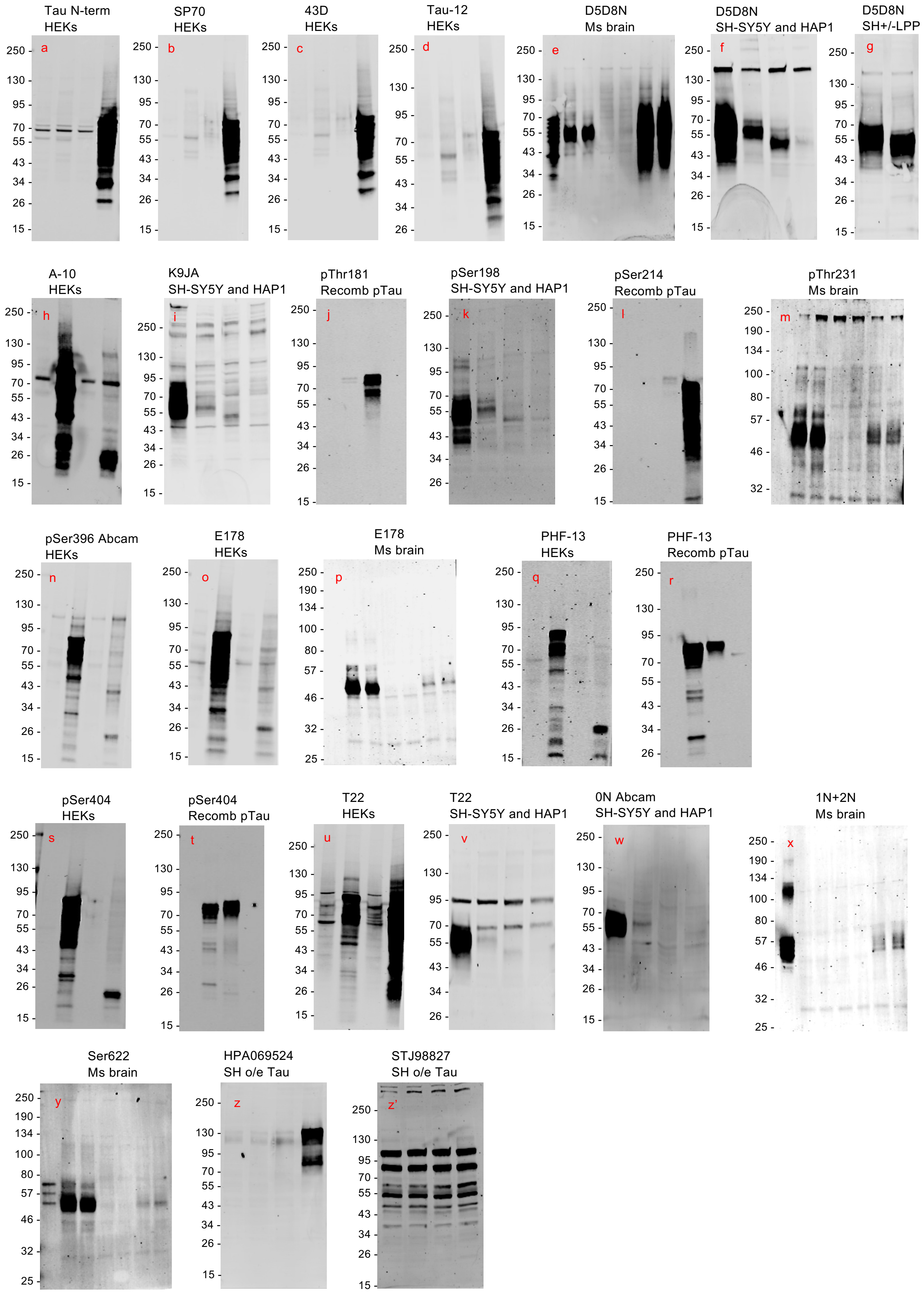
Tau N-term HEKs
a
SP70 HEKs
b
43D HEKs
c
Tau-12 HEKs
d
D5D8N Ms brain
e
D5D8N SH-SY5Y and HAP1
f
D5D8N SH+/-LPP
g
A-10 HEKs
h
K9JA SH-SY5Y and HAP1
i
pThr181 Recomb pTau
j
pSer198 SH-SY5Y and HAP1
k
pSer214 Recomb pTau
l
pThr231 Ms brain
m
pSer396 Abcam HEKs
n
E178 HEKs
o
E178 Ms brain
p
PHF-13 HEKs
q
PHF-13 Recomb pTau
r
pSer404 HEKs
s
pSer404 Recomb pTau
t
T22 HEKs
u
T22 SH-SY5Y and HAP1
v
0N Abcam SH-SY5Y and HAP1
w
1N+2N Ms brain
x
Ser622 Ms brain
y
HPA069524 SH o/e Tau
z
STJ98827 SH o/e Tau
z'

Supplementary Figure S23. Images of WB membranes probed with different Tau antibodies where adjusting the brightness/contrast display settings led to the oversaturation of the “main” Tau signal but revealed additional bands or allowed for better visualisation of weaker signals. Brightness/contrast-adjusted blot images are shown in this figure. Red lettering shown in the upper left corner links each blot to the corresponding one shown in **Supp. Figs. S4-S20**.

Supp. Fig. S24

Human brain – increased brightness/contrast to visualise faint bands

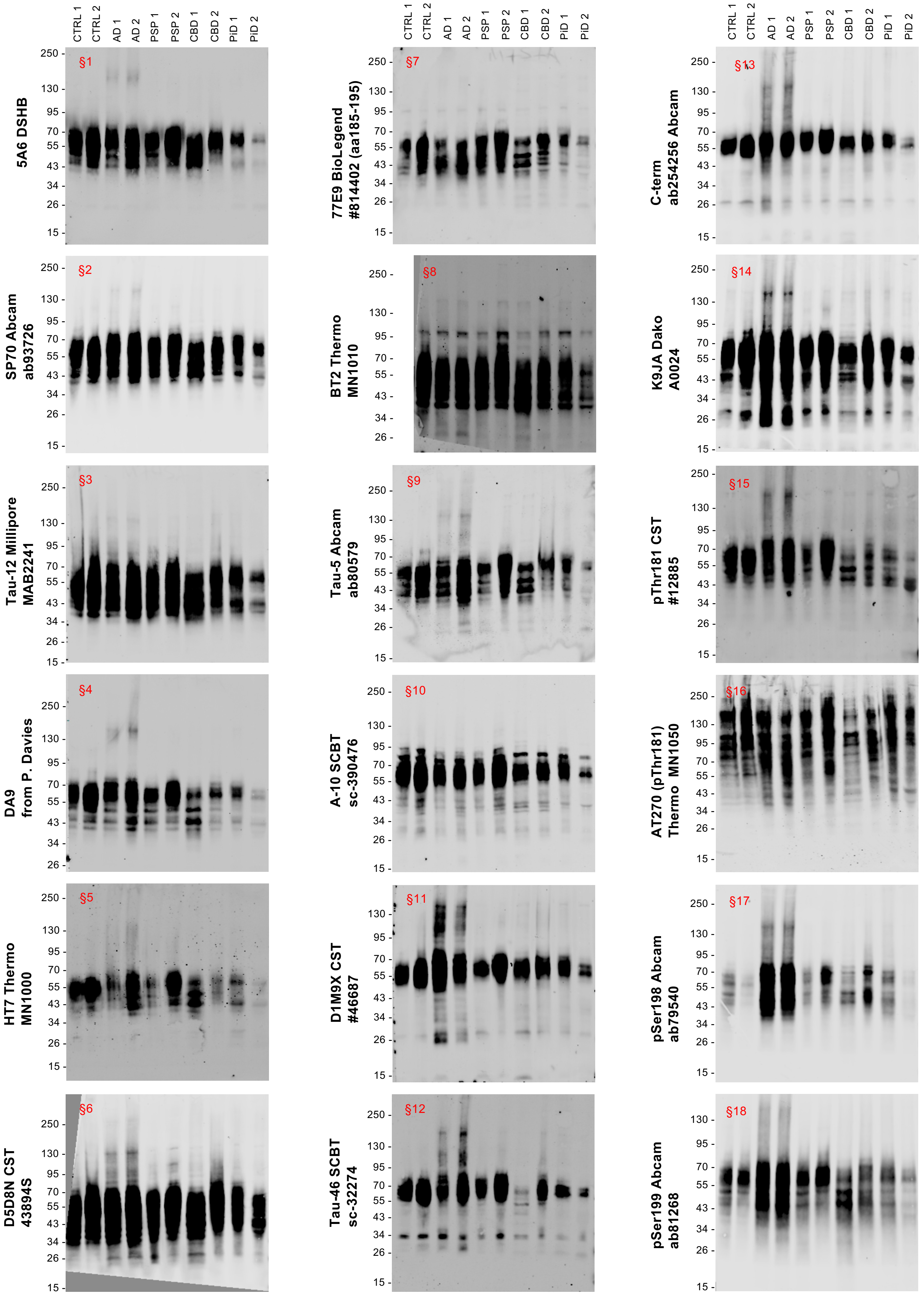

Supp. Fig. S25

Human brain – increased brightness/contrast to visualise faint bands

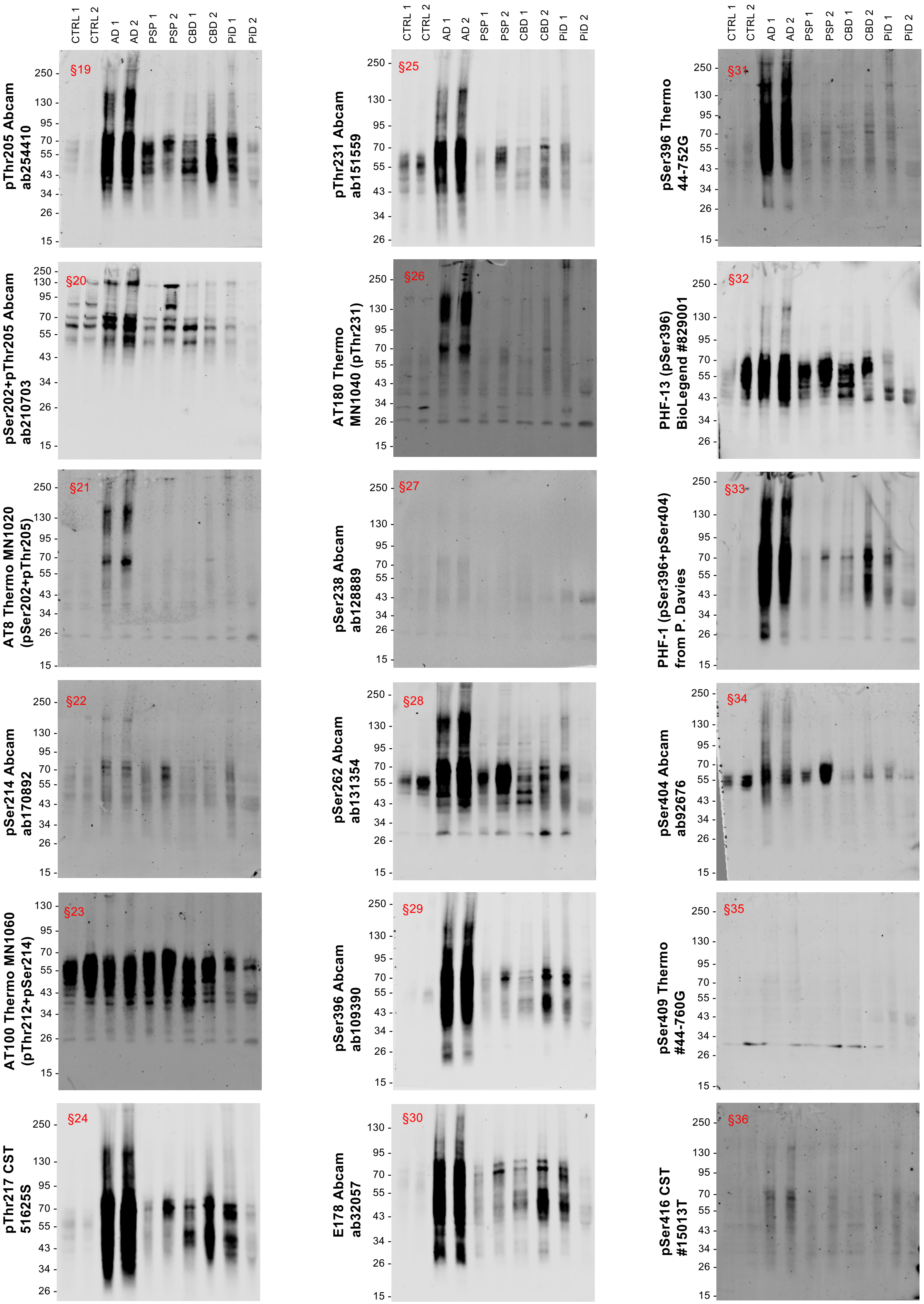

Supp. Fig. S26

Human brain – increased brightness/contrast to visualise faint bands

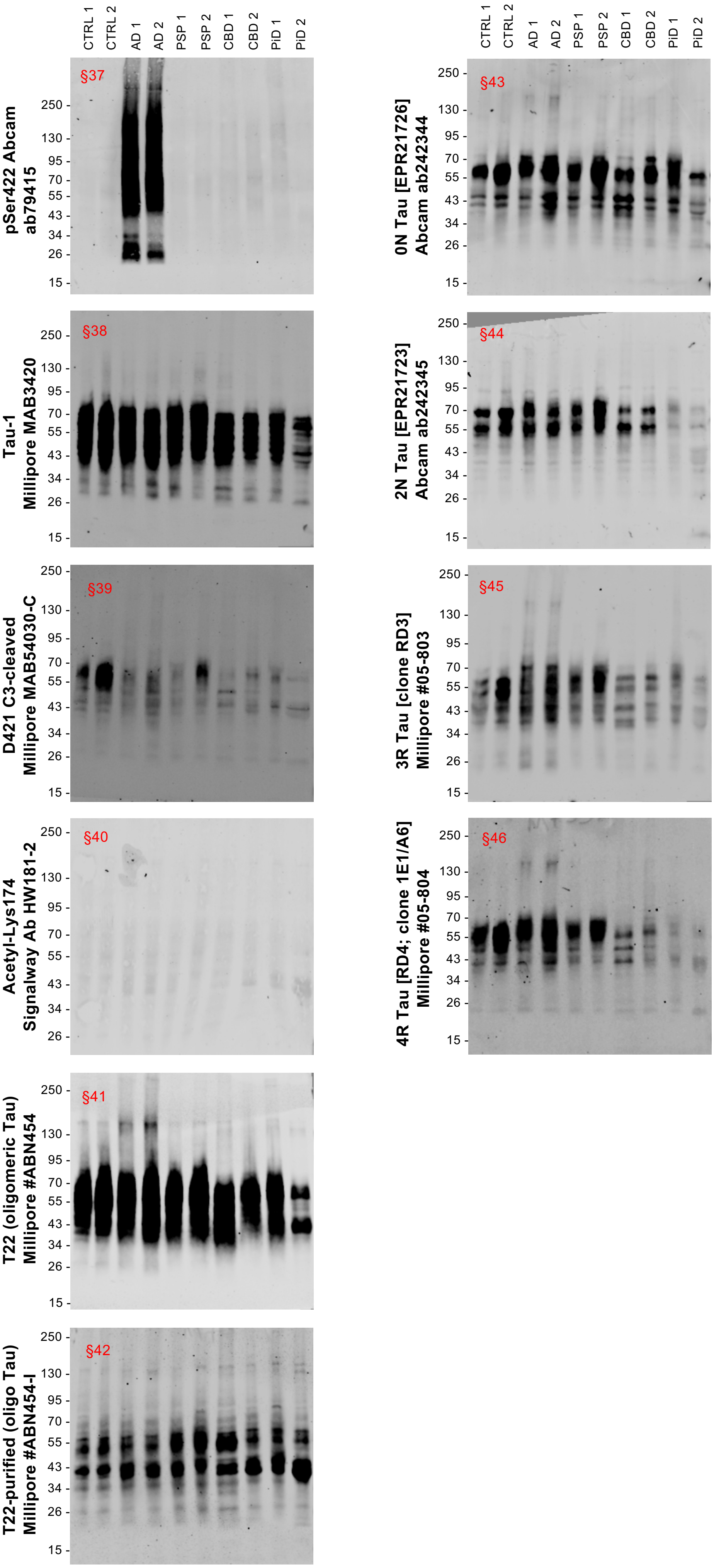

Supplementary Figure S24-S26. Images of human brain WB membranes probed with different Tau antibodies where adjusting the brightness/contrast display settings led to the oversaturation of the "main" Tau signal but allowed for better visualisation of weaker signals. Brightness/contrast-adjusted blot images are shown in this figure. The "§" symbol followed by a numeral shown in red in the upper left corner of each blot links it to the corresponding one shown in **Supp. Figs. S4-S18**.

Supp. Fig. S27

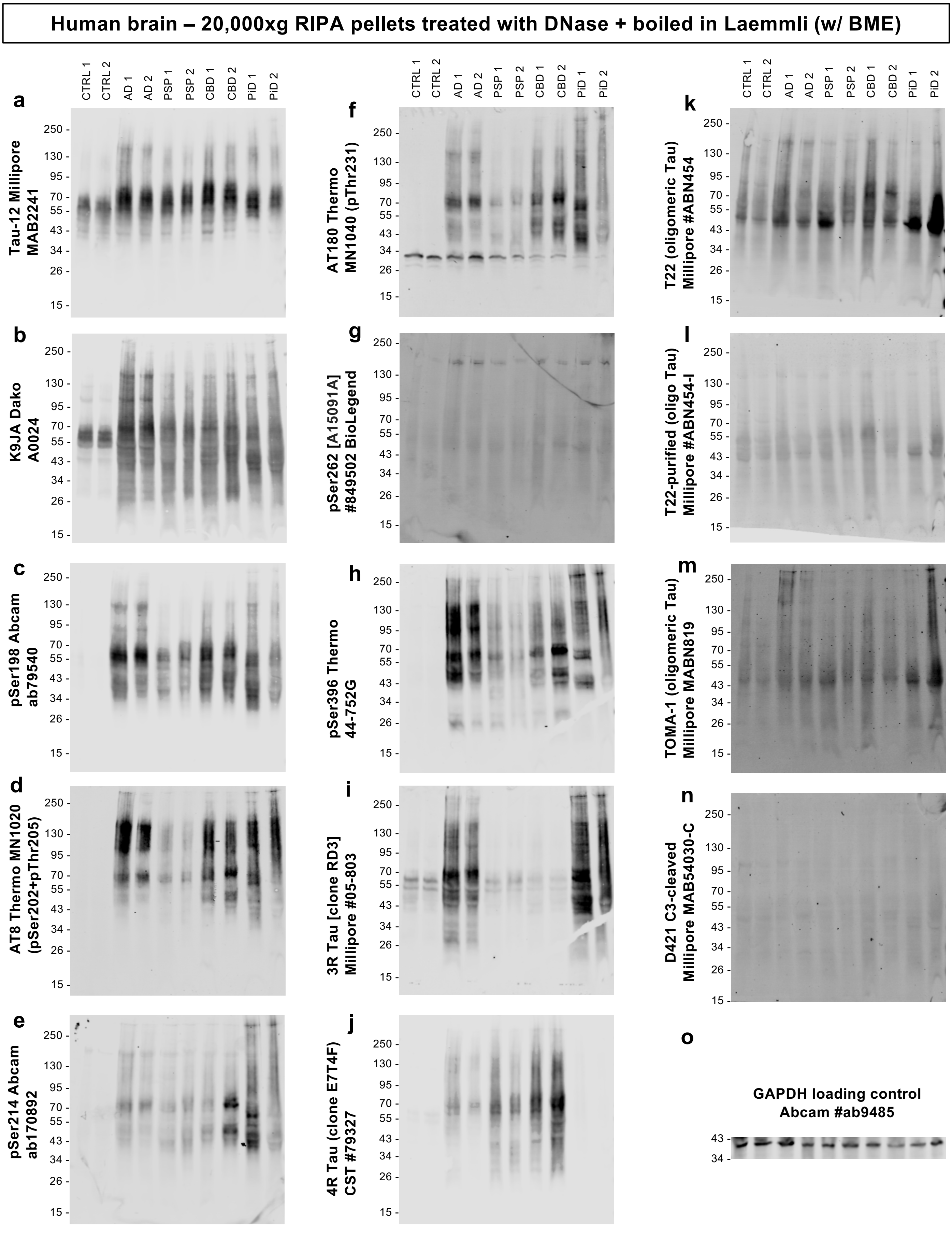
Human brain – 20,000xg RIPA pellets treated with DNase + boiled in Laemmli (w/ BME)
CTRL 1
CTRL 2
AD 1
AD 2
PSP 1
PSP 2
CBD 1
CBD 2
PiD 1
PiD 2
a
Tau-12 Millipore MAB2241
b
K9JA Dako A0024
c
pSer198 Abcam ab79540
d
AT8 Thermo MN1020 (pSer202+pThr205)
e
pSer214 Abcam ab170892
f
AT180 Thermo MN1040 (pThr231)
g
pSer262 [A15091A] #849502 BioLegend
h
pSer396 Thermo 44-752G
i
3R Tau [clone RD3] Millipore #05-803
j
4R Tau (clone E7T4F) CST #79327
k
T22 (oligomeric Tau) Millipore #ABN454
l
T22-purified (oligo Tau) Millipore #ABN454-I
m
TOMA-1 (oligomeric Tau) Millipore MABN819
n
D421 C3-cleaved Millipore MAB54030-C
o
GAPDH loading control Abcam #ab9485
250
130
95
70
55
43
34
26
15

Supplementary Figure S27. WBs on human brain RIPA pellets. WBs of proteins extracted from the pellets arising from the 20,000 xg spin following lysis of frozen human brain samples in 1x RIPA buffer as detailed in the Methods section. a-n: WB membranes of human brain proteins extracted from the low-speed RIPA pellet were probed with different Tau antibodies: Tau-12 Merck Millipore MAB2241 (**a**); K9JA Dako A0024 (**b**); pSer198 Abcam ab79540 (**c**); AT8 ThermoFisher Scientific MN1020 (**d**); pSer214 Abcam ab170892 (**e**); AT180 ThermoFisher Scientific MN1040 (**f**); pSer262 BioLegend 849502 (**g**); pSer396 ThermoFisher Scientific 44-752G (**h**); RD3 (3R Tau) Merck Millipore 05-803 (**i**); 4R Tau CST #79327 (**j**); T22 Merck Millipore ABN454 (**k**); T22 "purified" Merck Millipore ABN454-I (**l**); TOMA-1 Merck Millipore MABN819 (**m**); D421 Tau-C3 Merck Millipore MAB54030-C (**n**); GAPDH loading control antibody Abcam ab9485 (**o**).

Supp. Fig. S28

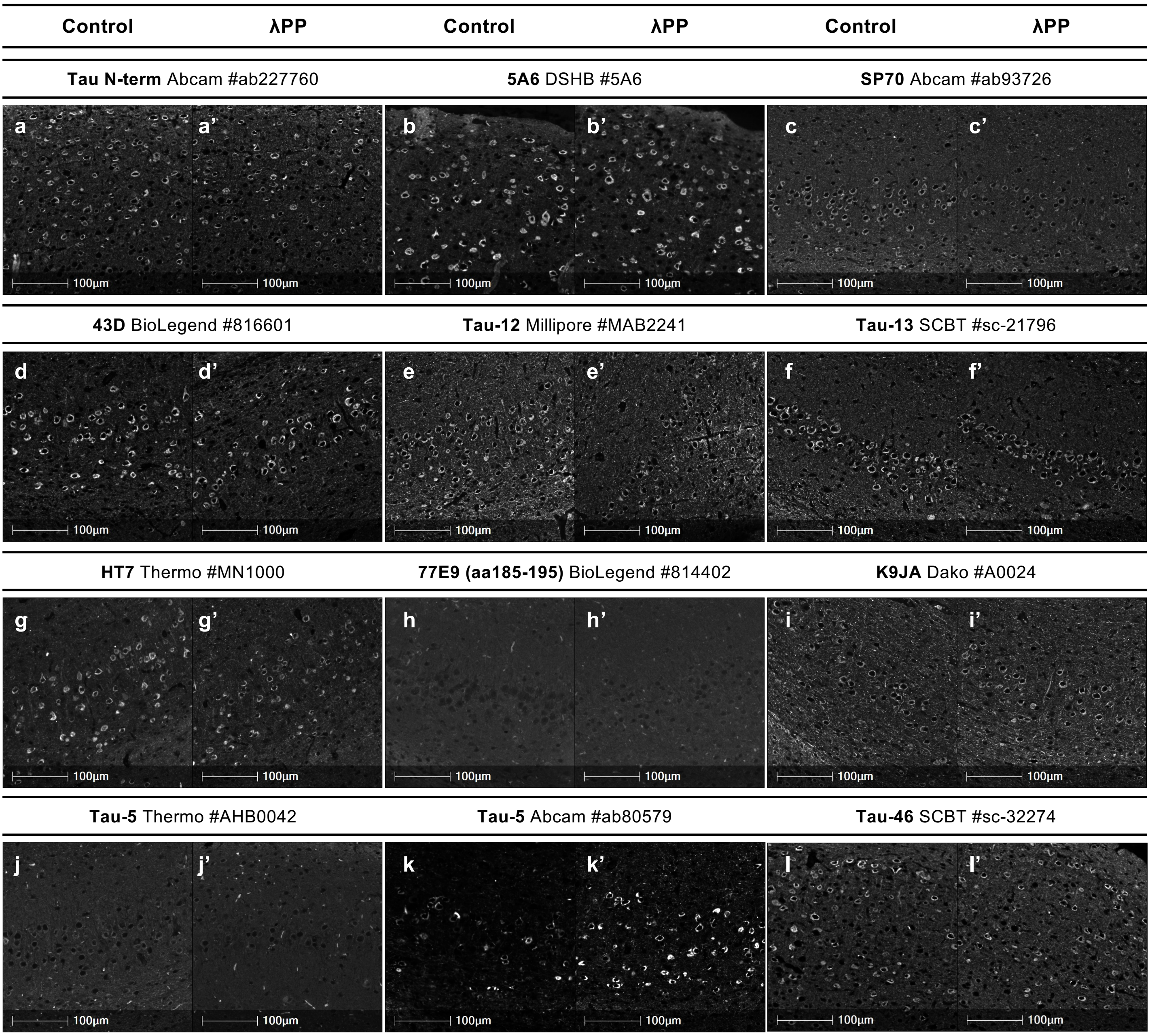

Control
λPP
Control
λPP
Control
λPP
Tau N-term Abcam #ab227760
5A6 DSHB #5A6
SP70 Abcam #ab93726
a
a'
b
b'
c
c'
43D BioLegend #816601
Tau-12 Millipore #MAB2241
Tau-13 SCBT #sc-21796
d
d'
e
e'
f
f'
HT7 Thermo #MN1000
77E9 (aa185-195) BioLegend #814402
K9JA Dako #A0024
g
g'
h
h'
i
i'
Tau-5 Thermo #AHB0042
Tau-5 Abcam #ab80579
Tau-46 SCBT #sc-32274
j
j'
k
k'
l
l'
100μm

Supplementary Figure S28. Validation of "total" Tau antibodies by IHC-IF (part 1). a-l': Fluorescence micrographs of serial FFPE brain sections from 9-month old rTg4510 mice, either untreated (control; **a, b, c, d, e, f, g, h, i, j, k and l**) or treated (λPP; **a', b', c', d', e', f', g', h', i', j', k' and l'**) with λPP, immunolabelled with Tau antibodies: N-terminal-targeting antibody Abcam #227760 (**a, a'**), clone 5A6 DSHB (**b, b'**), clone SP70 Abcam #ab93726 (**c, c'**), clone 43D BioLegend #816601 (**d, d'**), clone Tau-12 Millipore #MAB2241 (**e, e'**), clone Tau-13 Santa Cruz Biotechnology #sc-21796 (**f, f'**), clone HT7 ThermoFisher Scientific #MN1000 (**g, g'**), clone 77E9 BioLegend #814402 (**h, h'**), clone K9JA Dako #A0024 (**i, i'**), clone Tau-5 ThermoFisher Scientific #AHB0042 (**j, j'**), clone Tau-5 Abcam #ab80579 (**k, k'**), clone Tau-46 Santa Cruz Biotechnology #sc-32274 (**l, l'**). Brain region: cortex. Tau labelling is shown in grayscale. Scale bars = 100μm.

Supp. Fig. S29

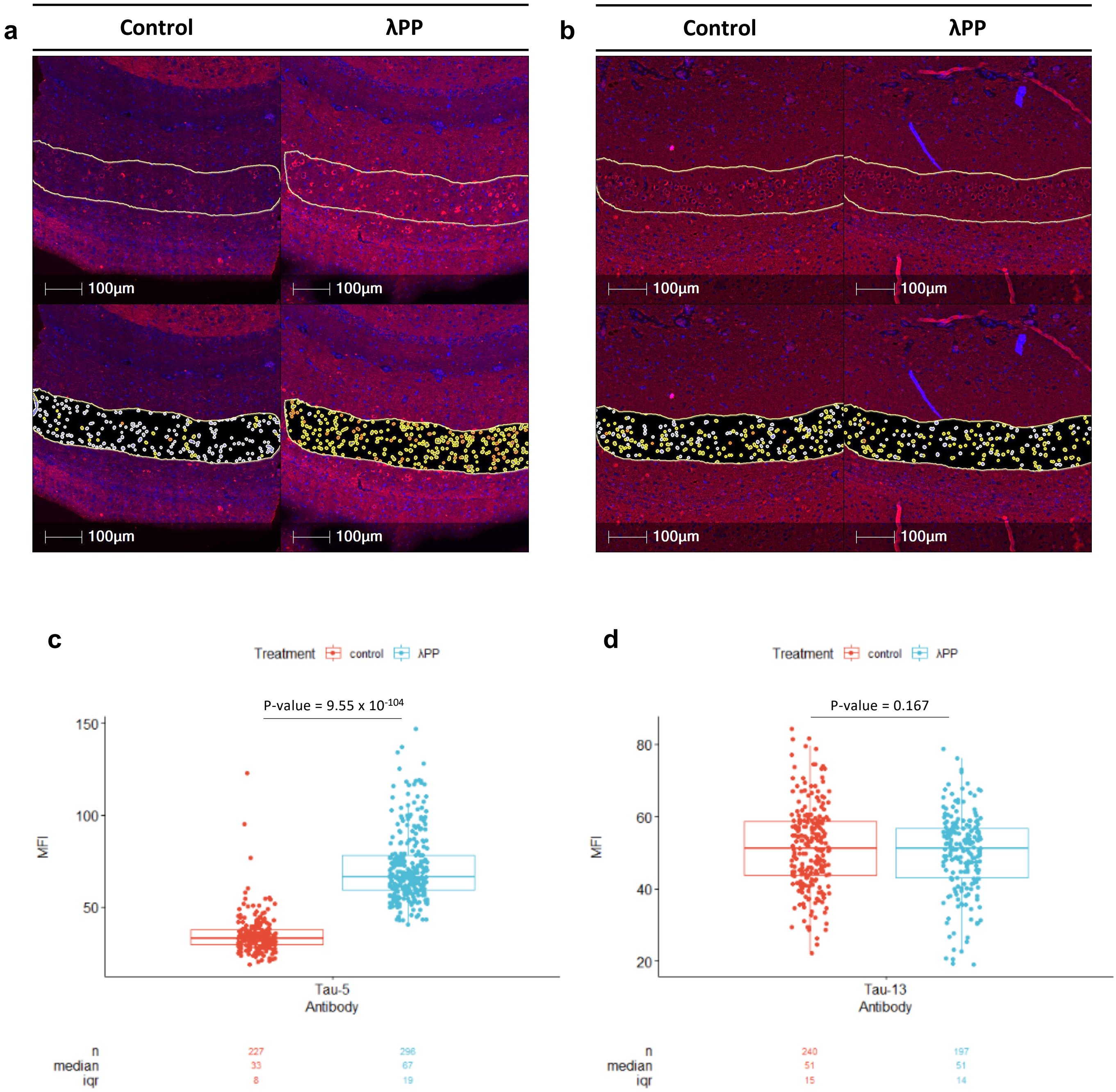

a
Control
λPP
100µm
100µm
100µm
100µm
b
Control
λPP
100µm
100µm
100µm
100µm
c
Treatment
control
λPP
P-value = 9.55 x 10-104
150
100
50
MFI
Tau-5
Antibody
n
median
iqr
227
33
8
296
67
19
d
Treatment
control
λPP
P-value = 0.167
80
60
40
20
MFI
Tau-13
Antibody
n
median
iqr
240
51
15
197
51
14

Supplementary Figure S29. Phosphorylation partially inhibits the binding of the Tau-5, but not Tau-13, antibody clone in FFPE-IHC applications. micrographs (**a, b**) and quantifications (**c, d**) of the signal mean fluorescence intensity (MFI) obtained in rTg4510 mouse brain cortex labelled with the Tau-5 (**a, c**) or Tau-13 (**b, d**) antibodies, before (**a, b** - left; **c, d** - red) and after (**a, b** - right; **c, d** - blue) λPP treatment. Tau signal is shown in red (**a, b**). Nuclei are labelled with DAPI (blue) (**a, b**). In **a** and **b**, the region used for quantifications is outlined and images in the bottom row highlight all the cells identified by the automated image analysis pipeline. Tau-negative cells are coloured in white (**a, b** - bottom row). Tau-positive cells are coloured in yellow and orange, indicating moderate and high Tau labelling signal intensity, respectively (**a, b** - bottom row). Scale bars = 100 μm (**a, b**). n= total number of cells analysed, iqr = inter-quartile range (**c, d**). p-values were calculated using a two-tailed, independent *t test*.

Supp. Fig. S30

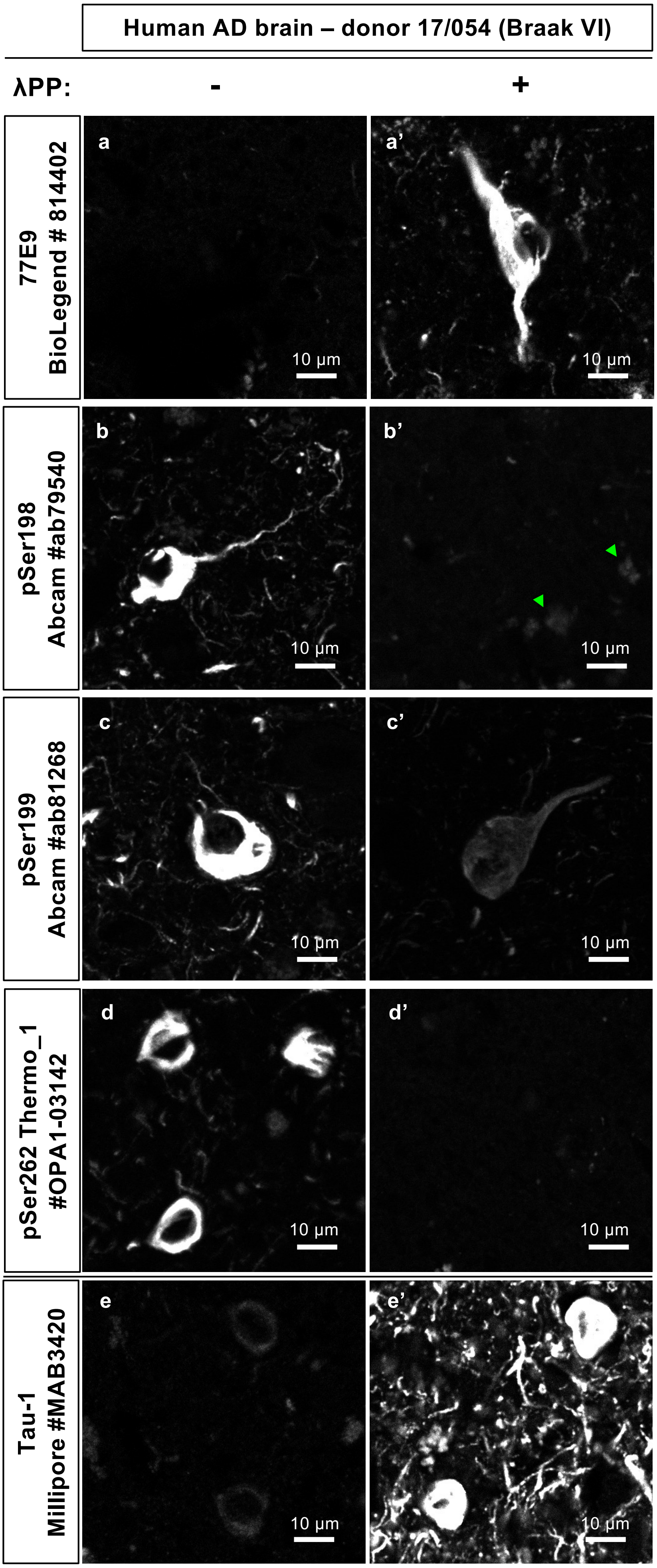
Human AD brain – donor 17/054 (Braak VI)
λPP:
-
+
77E9
BioLegend # 814402
pSer198
Abcam #ab79540
pSer199
Abcam #ab81268
pSer262 Thermo_1
#OPA1-03142
Tau-1
Millipore #MAB3420
a
a’
b
b’
c
c’
d
d’
e
e’
10 µm

Supplementary Figure S30. IHC-IF immunolabelling of FFPE human AD brain sections with the Tau-1 and 77EE9 antibody clones is dramatically enhanced following dephosphorylation of the tissue sections. Micrographs of cortical neurons from human AD brain (Braak stage VI) labelled with different Tau antibodies before (left) and after (right) λPP treatment. FFPE human brain frontal cortex sections were labelled with the following Tau antibodies (as indicated on the left): 77E9 BioLegend #814402 (**a**); pSer198 Abcam #79540 (**b**); pSer199 Abcam #81268 (**c**); and pSer262 ThermoFisher Scientific #OPA1-03142 (**d**); Tau-1 Millipore #MAB3420 (**e**). Tau signal is shown in grayscale. All images were acquired on the same day and microscope imaging settings were kept consistent for all slides. Adjacent tissue sections from the same brain sample were used for each antibody. Scale bars = 10 µm. Brain sections originated from the dorsolateral prefrontal cortex (Brodmann areas 9/46). Green arrowheads indicate highly autofluorescent lipofuscin particles (see **Supp. Fig. S2**).

Supp. Fig. S31

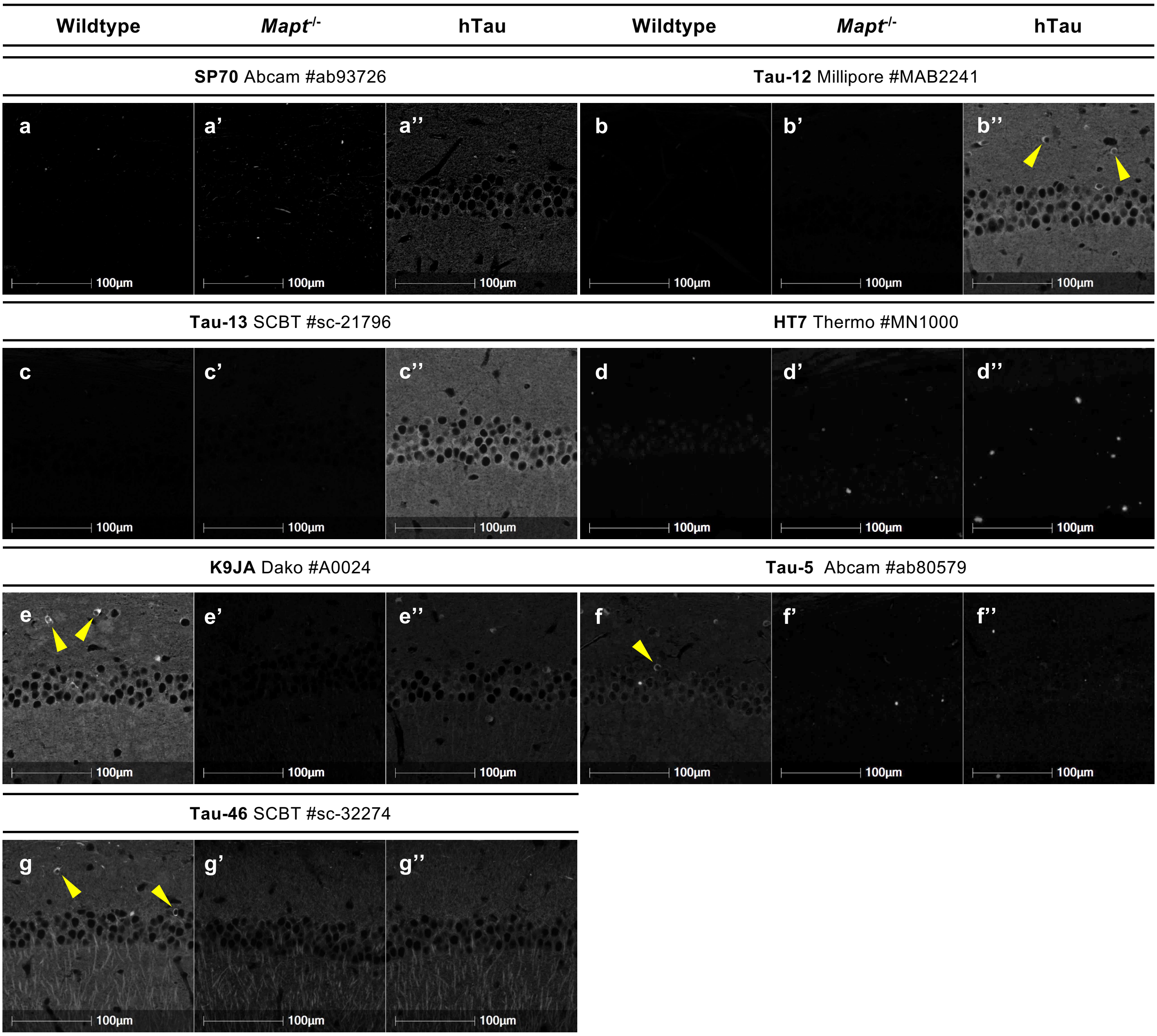
Wildtype
$Mapt^{-/-}$
hTau
Wildtype
$Mapt^{-/-}$
hTau
SP70 Abcam #ab93726
Tau-12 Millipore #MAB2241
Tau-13 SCBT #sc-21796
HT7 Thermo #MN1000
K9JA Dako #A0024
Tau-5 Abcam #ab80579
Tau-46 SCBT #sc-32274
a
a'
a''
b
b'
b''
c
c'
c''
d
d'
d''
e
e'
e''
f
f'
f''
g
g'
g''
100µm

Supplementary Figure S31. Validation of "total" Tau antibodies by IHC-IF (part 2). a-g": Fluorescence micrographs of FFPE brain sections from 5-month old wildtype (**a, b, c, d, e, f and g**), *Mapt*$^{-/-}$ (**a', b', c', d', e', f' and g'**) and hTau (**a'', b'', c'', d'', e'', f'' and g''**) mice immunolabelled with Tau antibodies: SP70 Abcam #ab93726 (**a-a''**), clone Tau-12 Millipore #MAB2241 (**b-b''**), clone Tau-13 Santa Cruz Biotechnology #sc-21796 (**c-c''**), clone HT7 ThermoFisher Scientific #MN1000 (**d-d''**), clone K9JA Dako #A0024 (**e-e''**), clone Tau-5 Abcam #ab80579 (**f-f''**), clone Tau-46 Santa Cruz Biotechnology #sc-32274 (**g-g''**). Yellow arrowheads indicate Tau-positive cell bodies located in *stratum oriens*. Brain region: CA1 region of the hippocampus. Tau labelling is shown in grayscale. Scale bars = 100µm.

Supp. Fig. S32

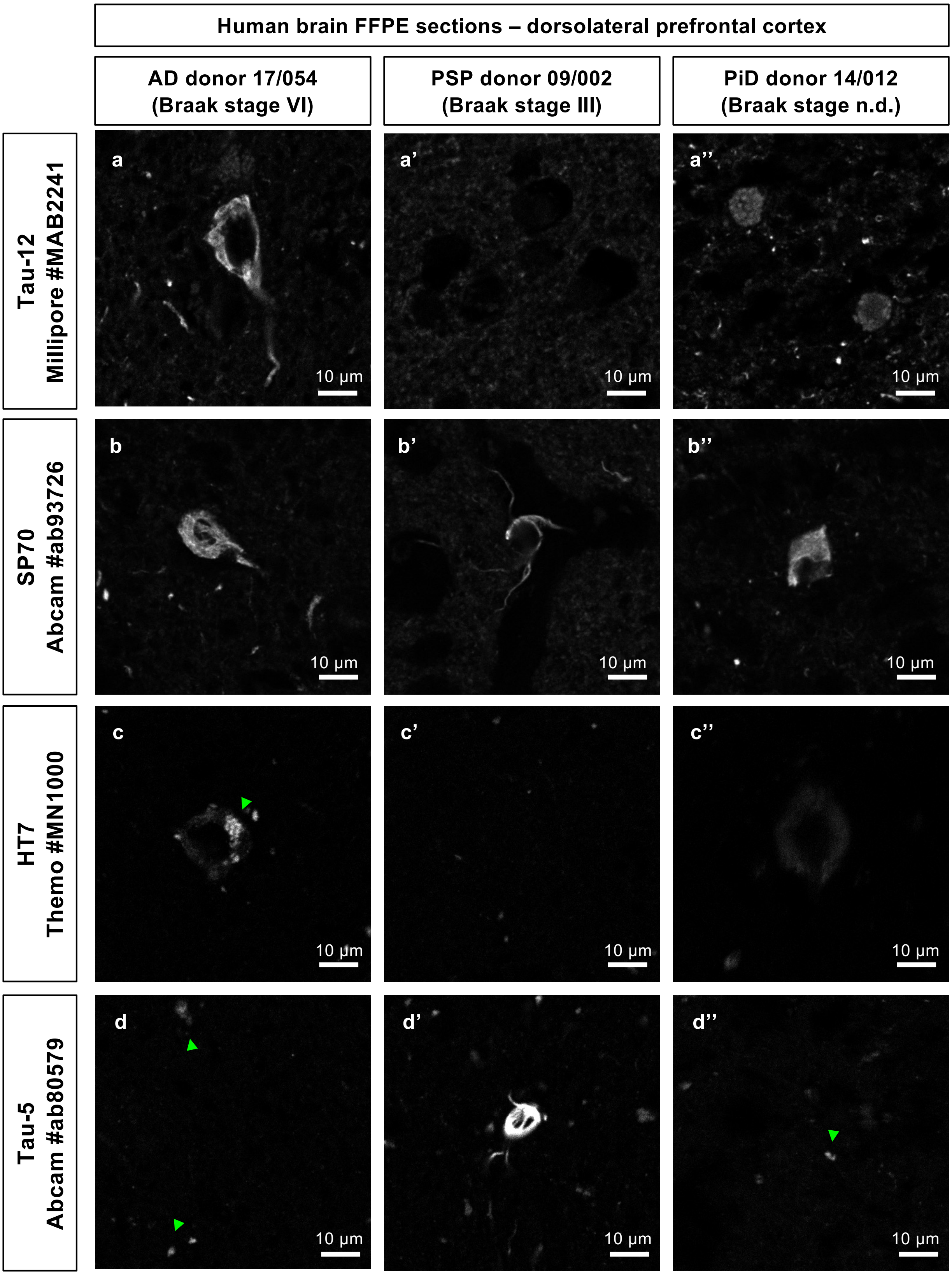

Human brain FFPE sections – dorsolateral prefrontal cortex
AD donor 17/054
(Braak stage VI)
PSP donor 09/002
(Braak stage III)
PiD donor 14/012
(Braak stage n.d.)
Tau-12
Millipore #MAB2241
SP70
Abcam #ab93726
HT7
Themo #MN1000
Tau-5
Abcam #ab80579
a
a'
a''
b
b'
b''
c
c'
c''
d
d'
d''
10 µm

Supplementary Figure S32. IHC-IF immunolabelling of FFPE human brain sections from tauopathy donors with "total" Tau antibodies. a-d: Tau-12 Merck Millipore MAB2241 (**a**); SP70 Abcam ab93726 (**b**); HT7 ThermoFisher Scientific MN1000 (**c**); Tau-5 Abcam ab80579 (**d**). Scale bars = 10 μm. Brain sections originated from the dorsolateral prefrontal cortex (Brodmann areas 9/46). Green arrowheads indicate highly autofluorescent lipofuscin particles (see **Supp. Fig. S2**).

Supp. Fig. S33

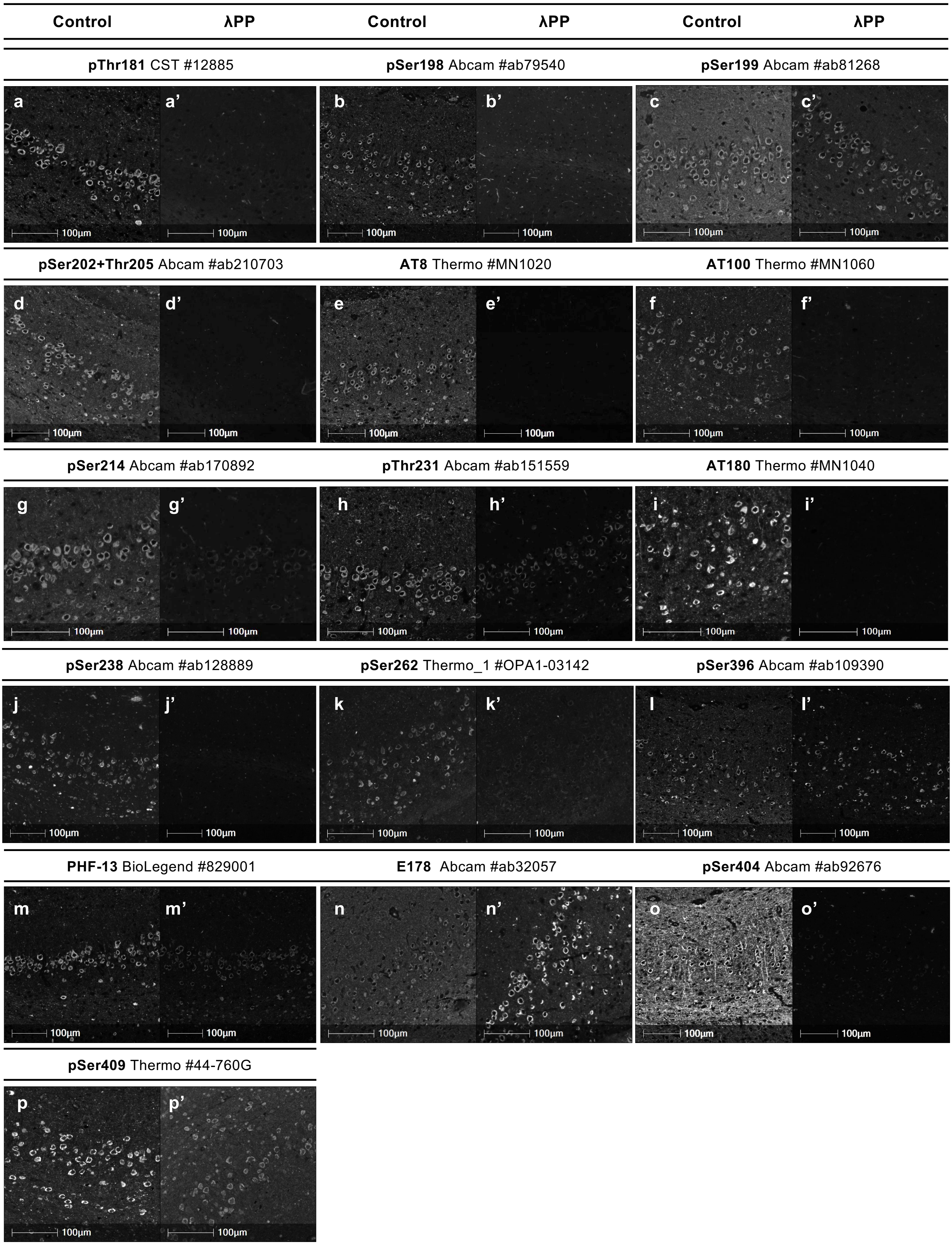
Control
λPP
Control
λPP
Control
λPP
pThr181 CST #12885
pSer198 Abcam #ab79540
pSer199 Abcam #ab81268
a
a’
b
b’
c
c’
pSer202+Thr205 Abcam #ab210703
AT8 Thermo #MN1020
AT100 Thermo #MN1060
d
d’
e
e’
f
f’
pSer214 Abcam #ab170892
pThr231 Abcam #ab151559
AT180 Thermo #MN1040
g
g’
h
h’
i
i’
pSer238 Abcam #ab128889
pSer262 Thermo_1 #OPA1-03142
pSer396 Abcam #ab109390
j
j’
k
k’
l
l’
PHF-13 BioLegend #829001
E178 Abcam #ab32057
pSer404 Abcam #ab92676
m
m’
n
n’
o
o’
pSer409 Thermo #44-760G
p
p’
100µm

Supplementary Figure S33. Validation of phospho-Tau antibodies by IHC-IF (part 1). a-p': Fluorescence micrographs of serial FFPE brain sections from 9-month old rTg4510 mice, either untreated (control; **a, b, c, d, e, f, g, h, i, j, k, l, m, n, o and p**) or treated (λPP; **a', b', c', d', e', f', g', h', i', j', k', l', m', n', o' and p'**) with λPP, immunolabelled with Tau antibodies: pThr181 Cell Signalling Technologies #12885 (**a, a'**), pSer198 Abcam #ab79540 (**b, b'**), pSer199 Abcam #81268 (**c, c'**), pSer202+pThr205 Abcam #210703 (**d, d'**), AT8 ThermoFisher Scientific #MN1020 (**e, e'**), AT100 ThermoFisher Scientific #MN1060 (**f, f'**), pSer214 Abcam #ab170892 (**g, g'**), pThr231 Abcam #ab151559 (**h, h'**), AT180 ThermoFisher Scientific #MN1040 (**i, i'**), pSer238 Abcam #ab128889 (**j, j'**), pSer262 ThermoFisher Scientific #OPA1-03142 (**k, k'**), pSer396 Abcam #ab109390 (**l, l'**), clone PHF-13 BioLegend #829001 (**m, m'**), clone E178 Abcam #ab32057 (**n, n'**), pSer404 Abcam #ab92676 (**o, o'**), Ser409 ThermoFisher Scientific #44-760G (**p, p'**). Brain region: cortex. Tau labelling is shown in grayscale. Scale bars = 100 µm.

Supp. Fig. S34

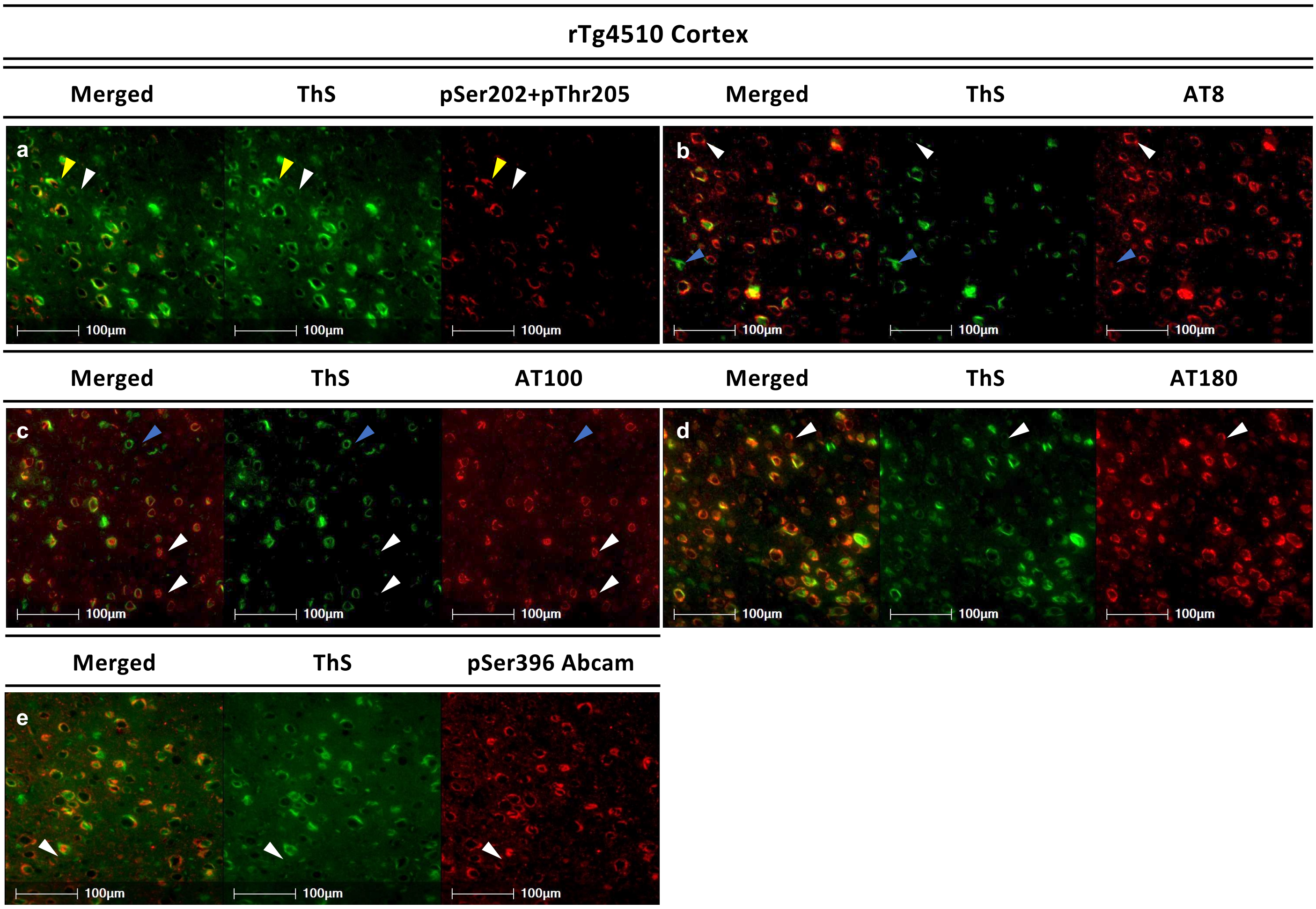
rTg4510 Cortex
Merged
ThS
pSer202+pThr205
Merged
ThS
AT8
a
b
100µm
Merged
ThS
AT100
Merged
ThS
AT180
c
d
Merged
ThS
pSer396 Abcam
e

Supplementary Figure S34. Majority of phosphorylated Tau in rTg4510 cortex is found in protein aggregates. FFPE rTg4510 mouse brain sections co-labelled with Thioflavin S (ThS, green) and phospho-Tau antibodies (red): pSer202+pThr205 (**a**), AT8 (**b**), AT100 (**c**), AT180 (**d**), pSer396 (**e**). Cortex region outlined in Supp. Fig. 2a is shown. Scale bars = 100 µm. A small subset of cells were positive for phospho-Tau but negative for ThS (white arrowheads), or positive for ThS but negative for the respective phospho-Tau antibody (blue arrowheads).

Supp. Fig. S35a

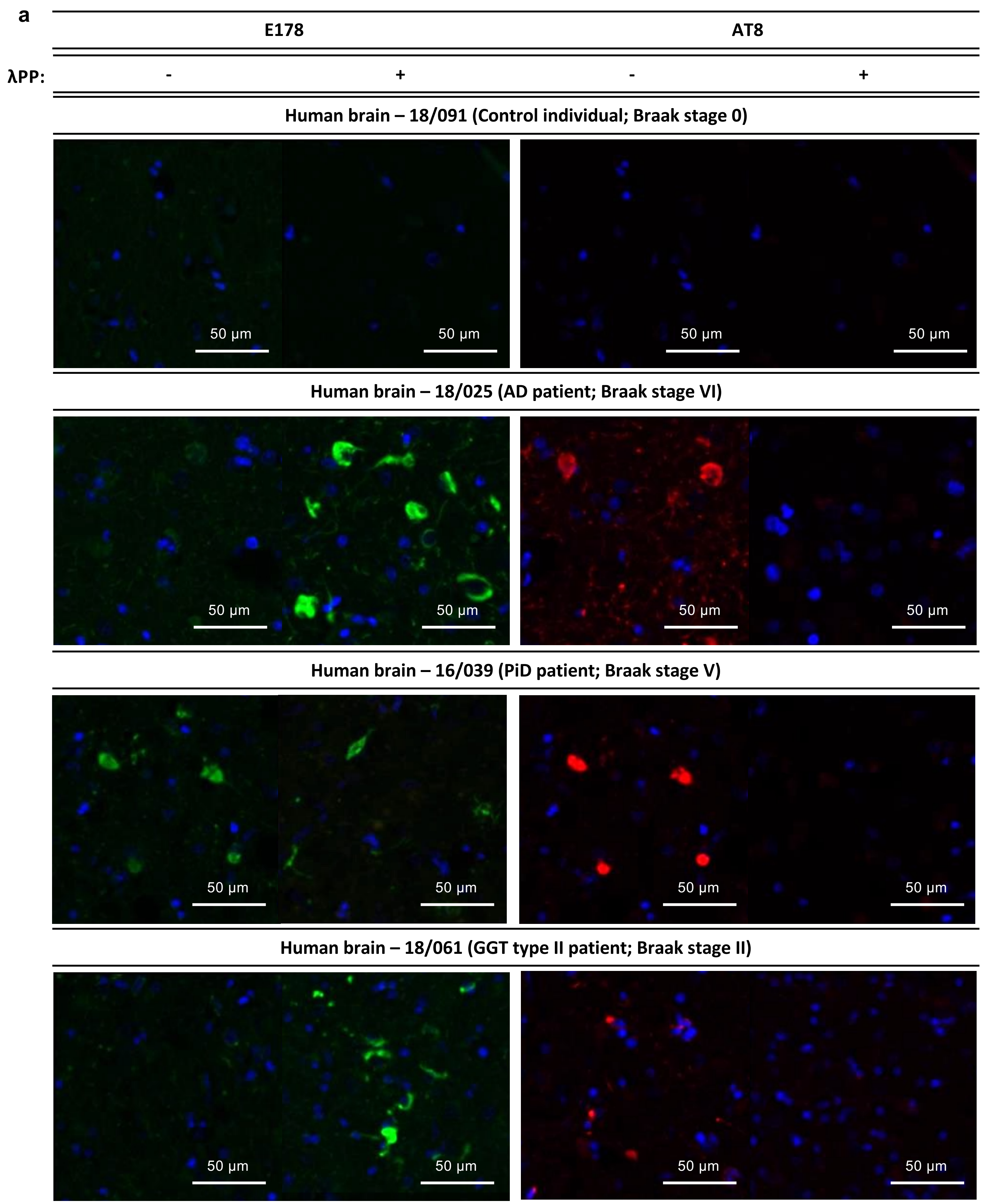

Supplementary Figure S35. E178 immunoreactivity to pathological PHF-Tau present in human brain sections is enhanced, rather than abrogated, following λPP treatment. a: Serial FFPE brain sections from control, AD, PiD and GGT individuals were either untreated (control) or treated (λPP) with λPP prior to immunolabelling with two different phospho-Tau antibodies: E178 (green) and AT8 (red). Nuclei were labelled with DAPI (blue). Scale bars = 50μm.

Supp. Fig. S35b (cont.)

b

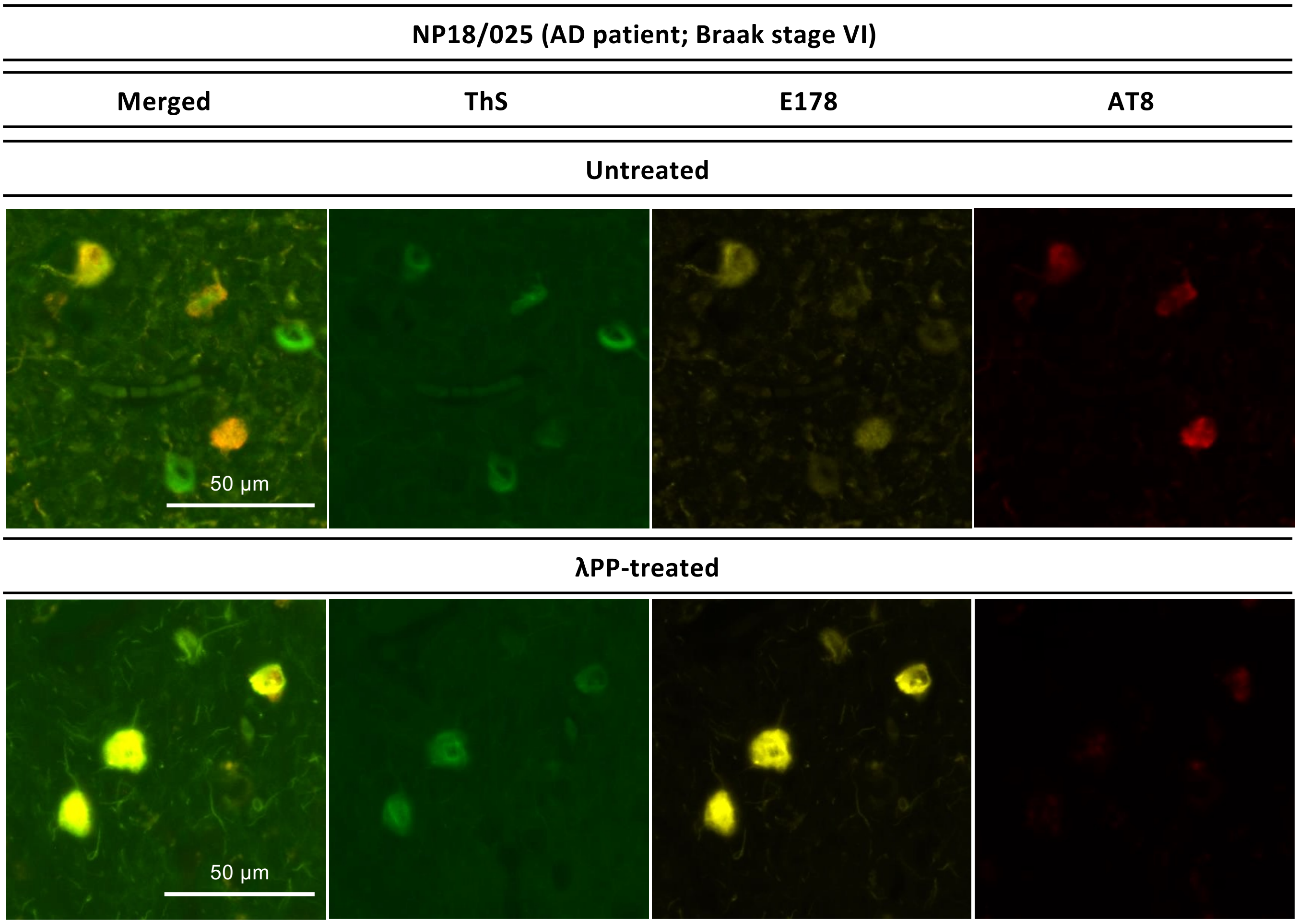

Supplementary Figure S35. E178 immunoreactivity to pathological PHF-Tau present in human brain sections is enhanced, rather than abrogated, following λPP treatment. b: Serial FFPE brain sections from an AD patient were pre-incubated with λPP (bottom row) or left untreated (top row), before immunolabelling with two different phospho-Tau antibodies: E178 (red) and AT8 (cyan). Sections were then labelled with ThS (green) to identify aggregated proteins. Areas that appear positive for ThS labelling but negative for phospho-Tau immunolabelling are presumed to represent amyloid deposits. Scale bars = 50µm. For the control, AD and PiD donors, brain sections originated from the dorsolateral prefrontal cortex (Brodmann areas 9/46). For the GGT donor, sections were taken from the M1 area (motor cortex).

Supp. Fig. S36

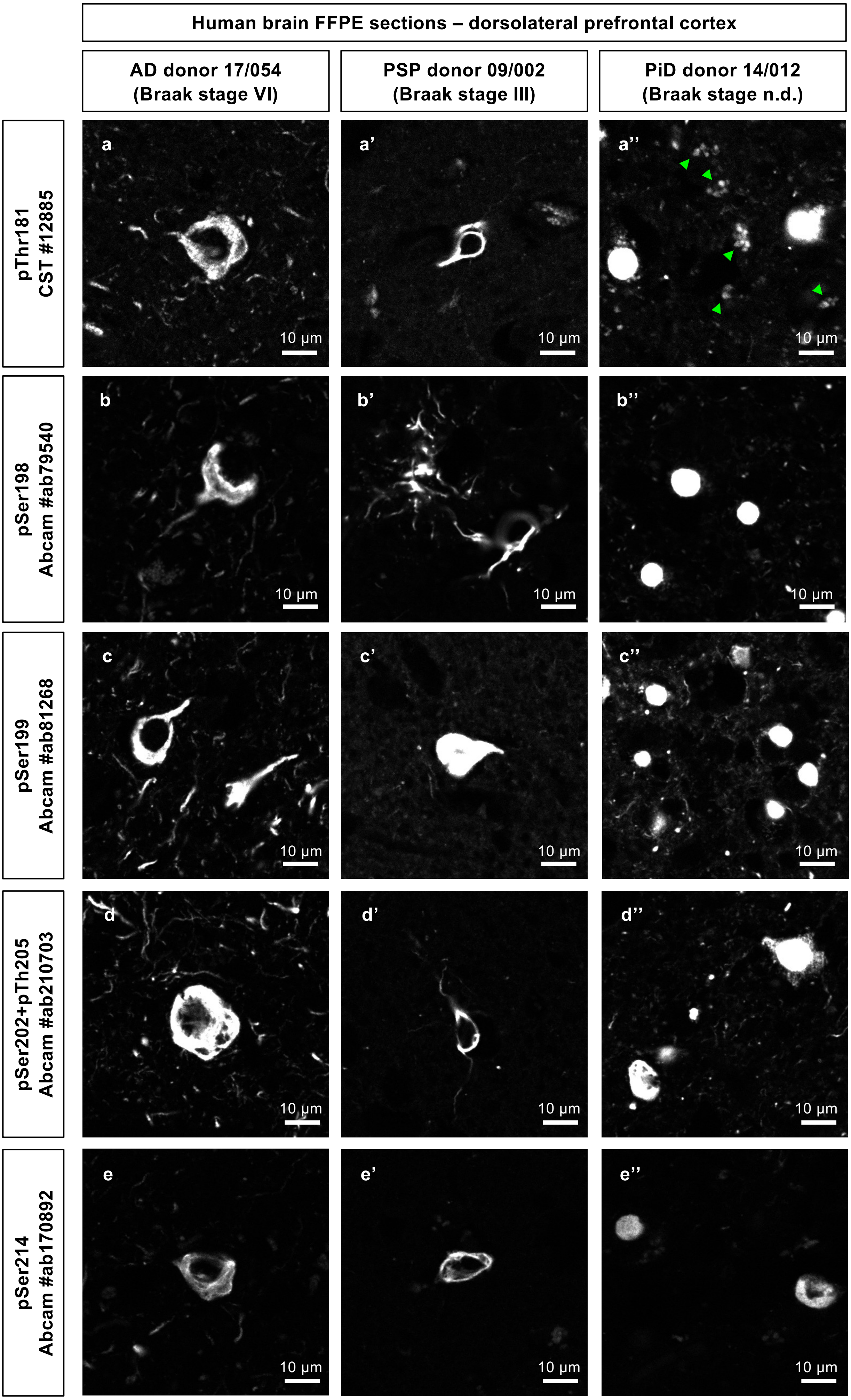
Human brain FFPE sections – dorsolateral prefrontal cortex
AD donor 17/054
(Braak stage VI)
PSP donor 09/002
(Braak stage III)
PiD donor 14/012
(Braak stage n.d.)
pThr181
CST #12885
pSer198
Abcam #ab79540
pSer199
Abcam #ab81268
pSer202+pTh205
Abcam #ab210703
pSer214
Abcam #ab170892
a
a’
a’’
b
b’
b’’
c
c’
c’’
d
d’
d’’
e
e’
e’’
10 µm

Supplementary Figure S36. IHC-IF immunolabelling of FFPE human brain sections from tauopathy donors with phospho-Tau antibodies (part I). a-e: pThr181 Cell Signalling 12885S (**a**); pSer198 Abcam ab79540 (**b**); pSer199 Abcam ab81268 (**c**); pSer202+pThr205 Abcam ab210703 (**d**); pSer214 Abcam ab170892 (**e**). Scale bars = 10 µm. Brain sections originated from the dorsolateral prefrontal cortex (Brodmann areas 9/46). Green arrowheads indicate highly autofluorescent lipofuscin particles (see **Supp. Fig. S2**)

Supp. Fig. S37

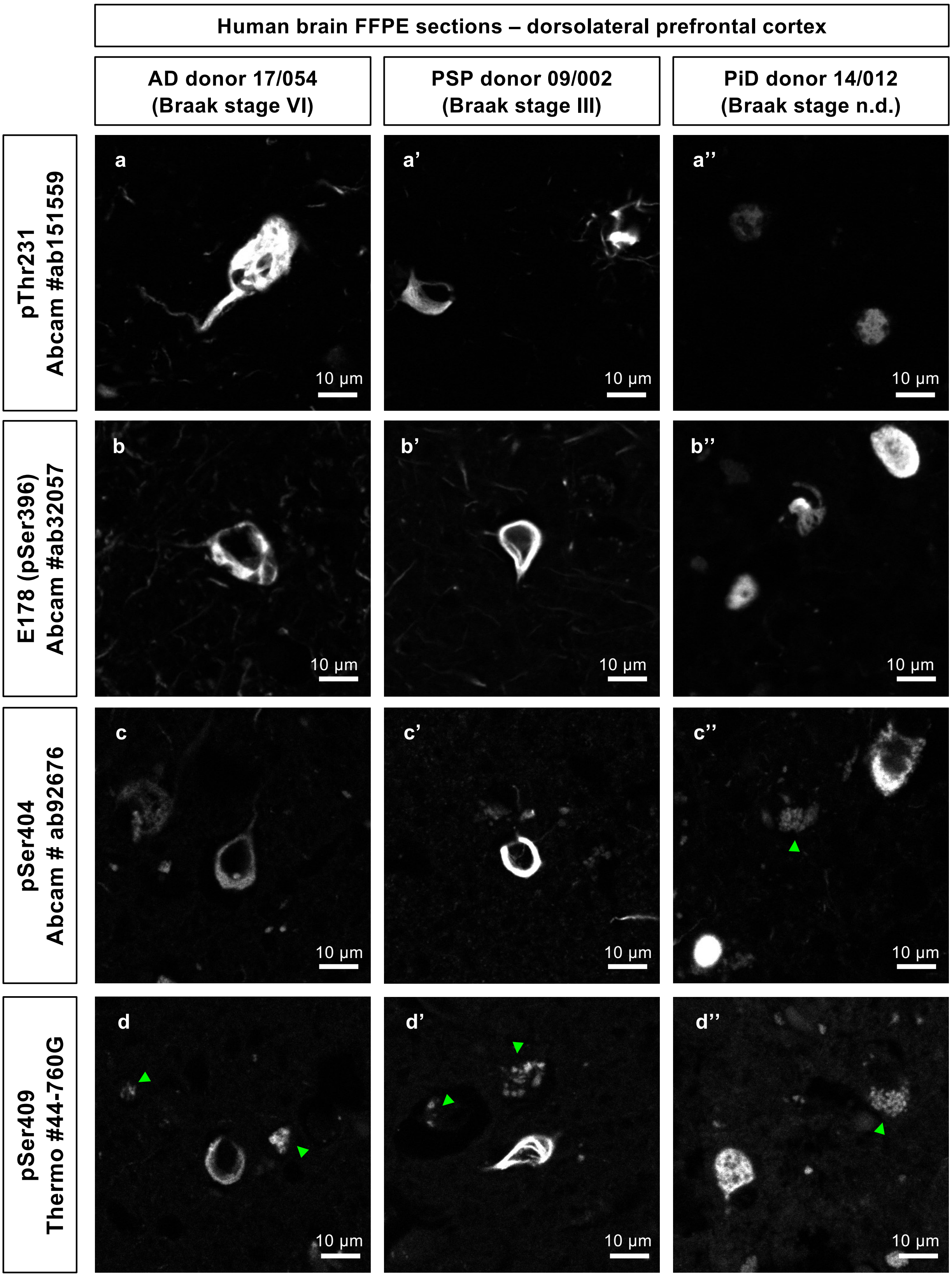
Human brain FFPE sections – dorsolateral prefrontal cortex
AD donor 17/054
(Braak stage VI)
PSP donor 09/002
(Braak stage III)
PiD donor 14/012
(Braak stage n.d.)
pThr231
Abcam #ab151559
E178 (pSer396)
Abcam #ab32057
pSer404
Abcam # ab92676
pSer409
Thermo #44-760G
a
a'
a''
b
b'
b''
c
c'
c''
d
d'
d''
10 µm

Supplementary Figure S37. IHC-IF immunolabelling of FFPE human brain sections from tauopathy donors with phospho-Tau antibodies (part II). a-d: pThr231 Abcam ab151559 (**a**); E178 (pSer396) Abcam ab32057 (**b**); pSer404 Abcam ab92676 (**c**); pSer409 ThermoFisher Scientific 44-760G (**d**). Scale bars = 10 µm. Brain sections originated from the dorsolateral prefrontal cortex (Brodmann areas 9/46). Green arrowheads indicate highly autofluorescent lipofuscin particles (see **Supp. Fig. S2**)

Supp. Fig. S38

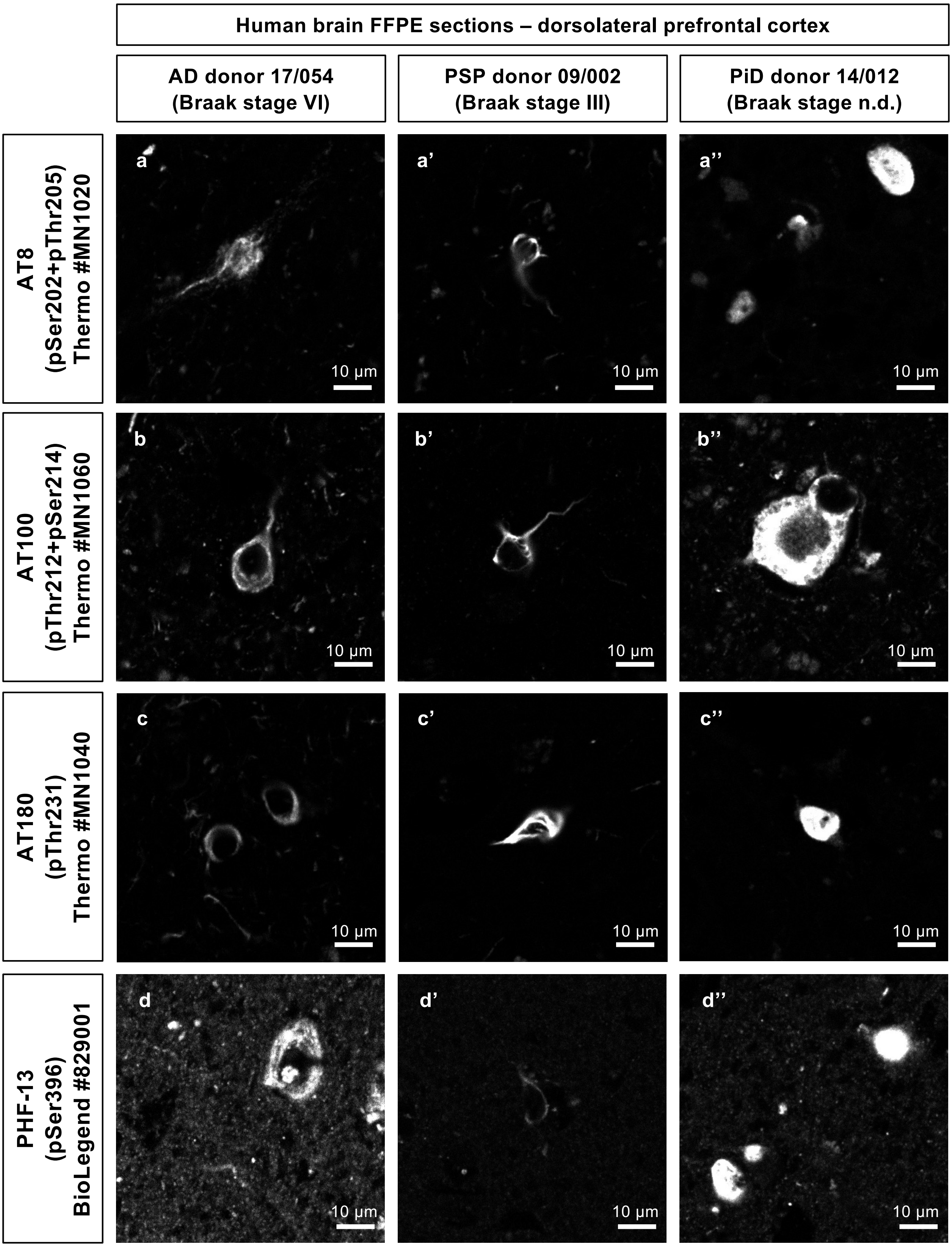
Human brain FFPE sections – dorsolateral prefrontal cortex
AD donor 17/054 (Braak stage VI)
PSP donor 09/002 (Braak stage III)
PiD donor 14/012 (Braak stage n.d.)
AT8 (pSer202+pThr205) Thermo #MN1020
AT100 (pThr212+pSer214) Thermo #MN1060
AT180 (pThr231) Thermo #MN1040
PHF-13 (pSer396) BioLegend #829001
a
a’
a’’
b
b’
b’’
c
c’
c’’
d
d’
d’’
10 µm

Supplementary Figure S38. IHC-IF immunolabelling of FFPE human brain sections from tauopathy donors with phospho-Tau antibodies (part III). a-d: AT8 ThermoFisher Scientific MN1020 (**a**); AT100 ThermoFisher Scientific MN1060 (**b**); AT180 ThermoFisher Scientific MN1040 (**c**); PHF-13 (pSer396) BioLegend 829001 (d). Scale bars = 10 μm. Brain sections originated from the dorsolateral prefrontal cortex (Brodmann areas 9/46).

Supp. Fig. S39

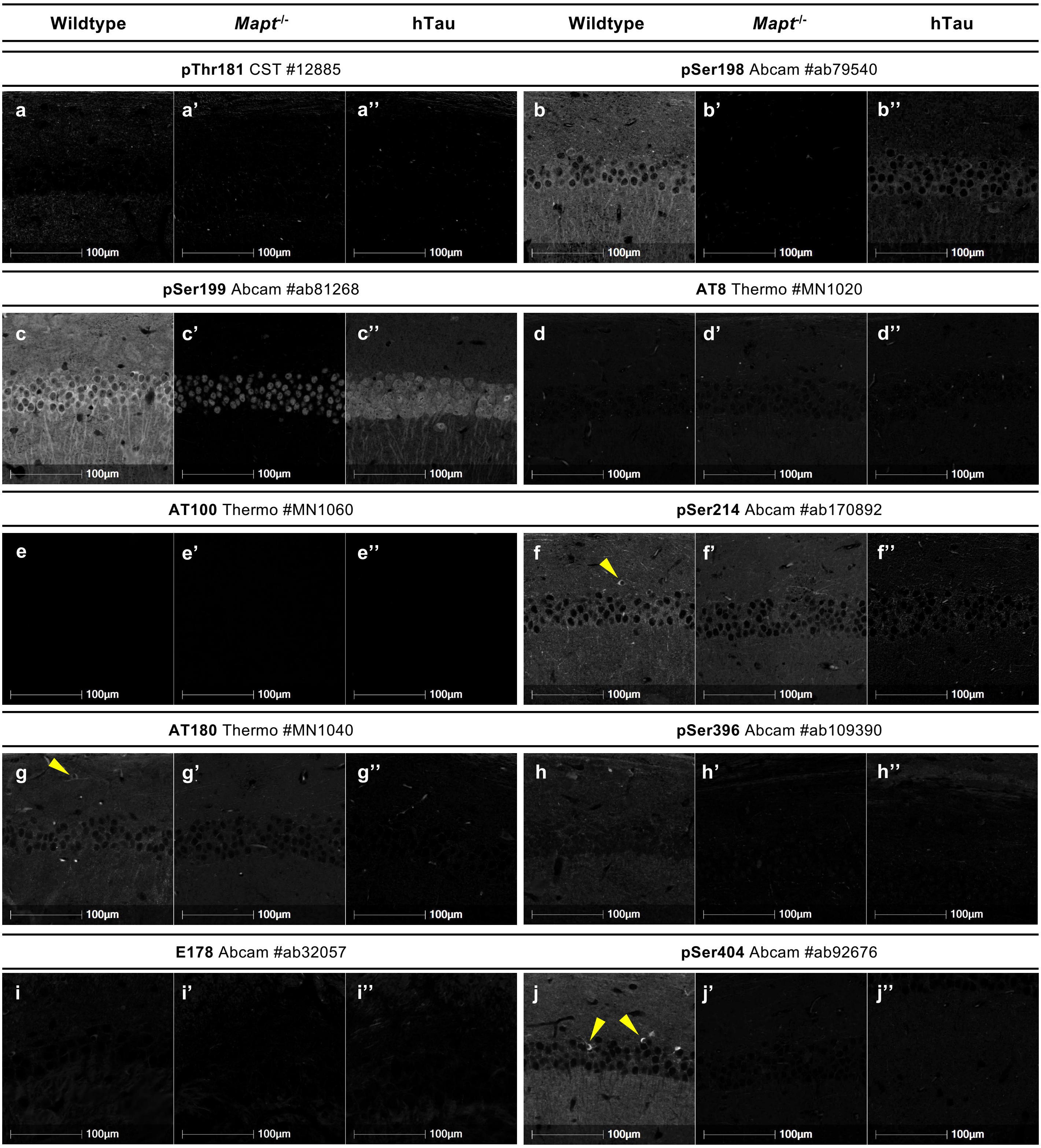
Wildtype
Mapt-/-
hTau
Wildtype
Mapt-/-
hTau
pThr181 CST #12885
pSer198 Abcam #ab79540
a a' a''
b b' b''
pSer199 Abcam #ab81268
AT8 Thermo #MN1020
c c' c''
d d' d''
AT100 Thermo #MN1060
pSer214 Abcam #ab170892
e e' e''
f f' f''
AT180 Thermo #MN1040
pSer396 Abcam #ab109390
g g' g''
h h' h''
E178 Abcam #ab32057
pSer404 Abcam #ab92676
i i' i''
j j' j''
100µm

Supplementary Figure S39. Validation of phospho-Tau antibodies by IHC-IF (part 2). a-j”: Fluorescence micrographs of FFPE brain sections from 5-month old wildtype (**a, b, c, d, e, f, g, h, i and j**), *Mapt*$^{-/-}$ (**a’, b’, c’, d’, e’, f’, g’, h’, i’ and j**’) and hTau (**a”, b”, c”, d”, e”, f”, g”, h”, i” and j”**) mice immunolabelled with Tau antibodies: pThr181 Cell Signalling Technologies #12885 (**a-a”**), pSer198 Abcam #ab79540 (**b-b”**), pSer199 Abcam #81268 (**c-c”**), AT8 ThermoFisher Scientific #MN1020 (**d-d”**), AT100 ThermoFisher Scientific #MN1060 (**e-e”**), pSer214 Abcam #ab170892 (**f-f”**), AT180 ThermoFisher Scientific #MN1040 (**g-g”**), pSer396 Abcam #ab109390 (**h-h”**), clone E178 Abcam #ab32057 (**i-i”**), pSer404 Abcam #ab92676 (**j-j”**). Yellow arrowheads indicate Tau-positive cell bodies located in *stratum oriens*. Brain region: CA1 region of the hippocampus. Tau labelling is shown in grayscale. Scale bars = 100 µm.

Supp. Fig. S40

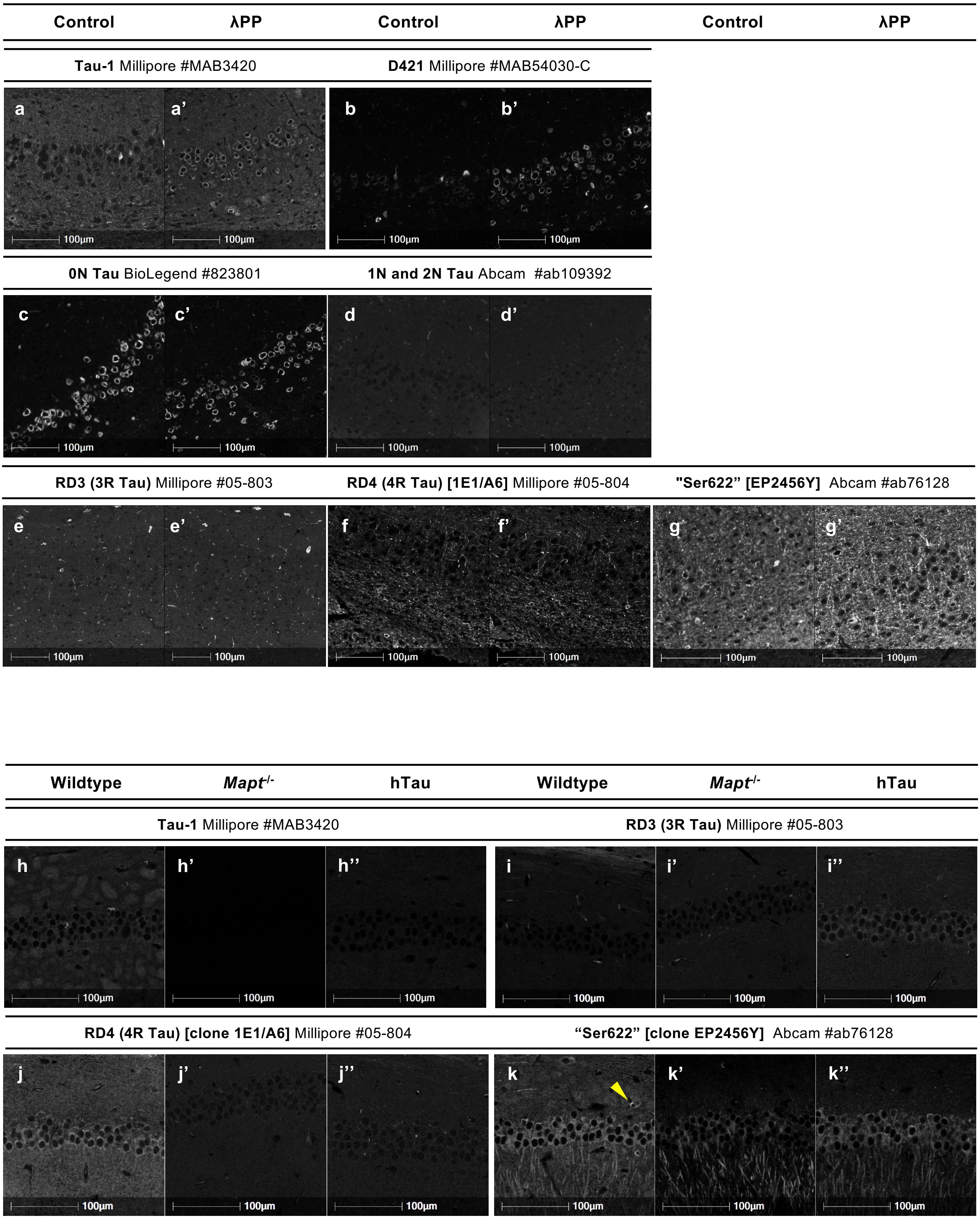
Control
λPP
Control
λPP
Control
λPP
Tau-1 Millipore #MAB3420
D421 Millipore #MAB54030-C
a
a’
b
b’
100µm
0N Tau BioLegend #823801
1N and 2N Tau Abcam #ab109392
c
c’
d
d’
RD3 (3R Tau) Millipore #05-803
RD4 (4R Tau) [1E1/A6] Millipore #05-804
"Ser622" [EP2456Y] Abcam #ab76128
e
e’
f
f’
g
g’
Wildtype
Mapt-/-
hTau
Wildtype
Mapt-/-
hTau
Tau-1 Millipore #MAB3420
RD3 (3R Tau) Millipore #05-803
h
h’
h’’
i
i’
i’’
RD4 (4R Tau) [clone 1E1/A6] Millipore #05-804
“Ser622” [clone EP2456Y] Abcam #ab76128
j
j’
j’’
k
k’
k’’

Supplementary Figure S40. Validation of isoform-specific Tau antibodies and other PTM-dependent Tau antibodies by IHC. a-g': Fluorescence micrographs of serial FFPE brain sections from 9-month old rTg4510 mice, either untreated (control; **a, b, c, d, e, f and g**) or treated (λPP; **a', b', c', d', e', f' and g'**) with λPP, immunolabelled with Tau antibodies: clone Tau-1 Millipore #MAB3420 (**a, a'**), clone D421 Millipore #MAB54030-C (**b, b'**) 0N Tau BioLegend #823801 (**c, c'**), 1N and 2N Tau Abcam #ab109392 (**d, d'**), clone RD3 (3R Tau) Millipore #05-803 (**e, e'**), clone RD4 (4R Tau) Millipore #05-804 (**f, f'**), "Ser622" Abcam #ab76128 (**g, g'**). Brain region: cortex. Tau labelling is shown in grayscale. Scale bars = 100 µm. **h-k":** Fluorescence micrographs of FFPE brain sections from 5-month old wildtype (**h, i, j and k**), *Mapt*$^{-/-}$ (**h', i', j' and k'**) and hTau (**h'', i'', j'' and k''**) mice immunolabelled with Tau antibodies: clone Tau-1 Millipore #MAB3420 (**h-h''**), clone RD3 (3R Tau) Millipore #05-803 (**i-i''**), clone RD4 (4R Tau) Millipore #05-804 (**j-j''**), "Ser622" Abcam #ab76128 (**k-k''**). Yellow arrowheads indicate Tau-positive cell bodies located in *stratum oriens*. Brain region imaged: CA1 region of the hippocampus. Tau labelling is shown in grayscale. Scale bars = 100 µm.

Supp. Fig. S41

a

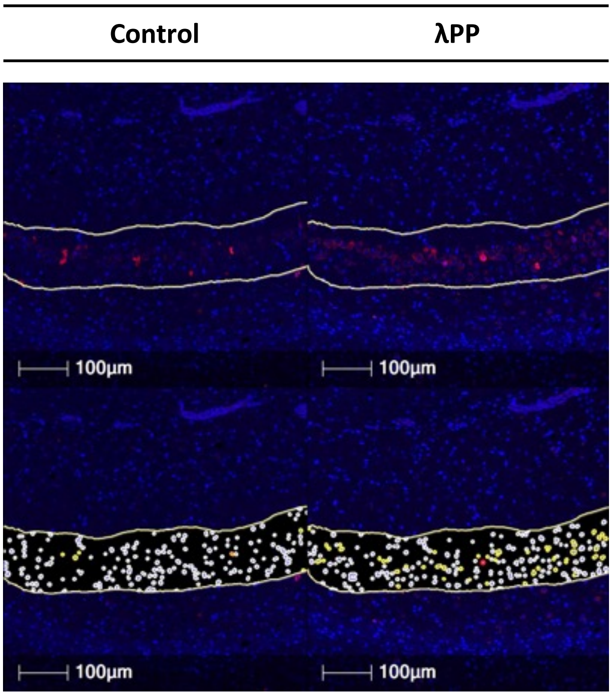

b

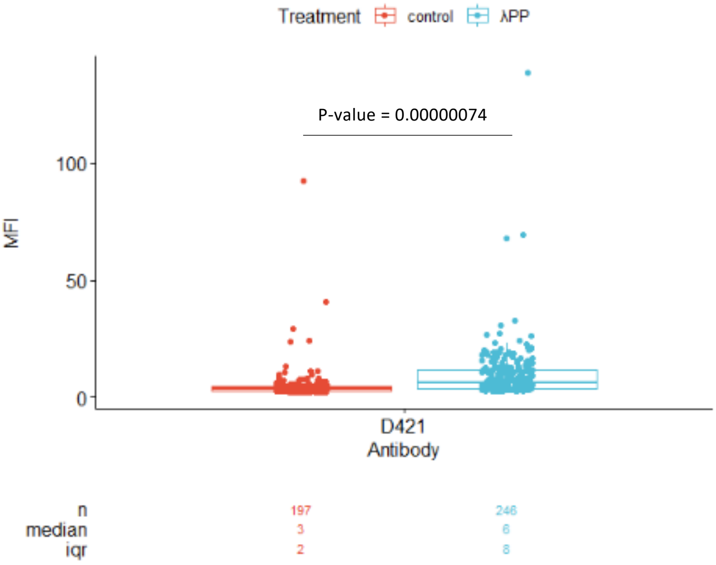

Supplementary Figure S41. FFPE-IHC immunolabelling with the D421 antibody clone is enhanced following dephosphorylation of the tissue sections. Micrographs (**a**) and quantifications (**b**) of the signal mean fluorescence intensity (MFI) obtained in rTg4510 mouse brain cortex labelled with the D421 antibody, before (**a** - left; **b** - red) and after (**a** - right; **b** - blue) λPP treatment. Tau signal is shown in red (**a**). Nuclei are labelled with DAPI (blue) (**a**). The area used for quantifications is outlined (**a**). In **a**, bottom rows show all the cells identified by the image analysis pipeline. Tau-negative cells are coloured in white (**a** - bottom row). Tau-positive cells are coloured in yellow and orange, indicating moderate and high Tau labelling signal intensity, respectively (**a** - bottom row). Scale bars = 100 µm. (**a**). n = total number of cells of cells analysed, iqr = inter-quartile range (**b**). p-values were calculated using a two-tailed, independent *t test*.

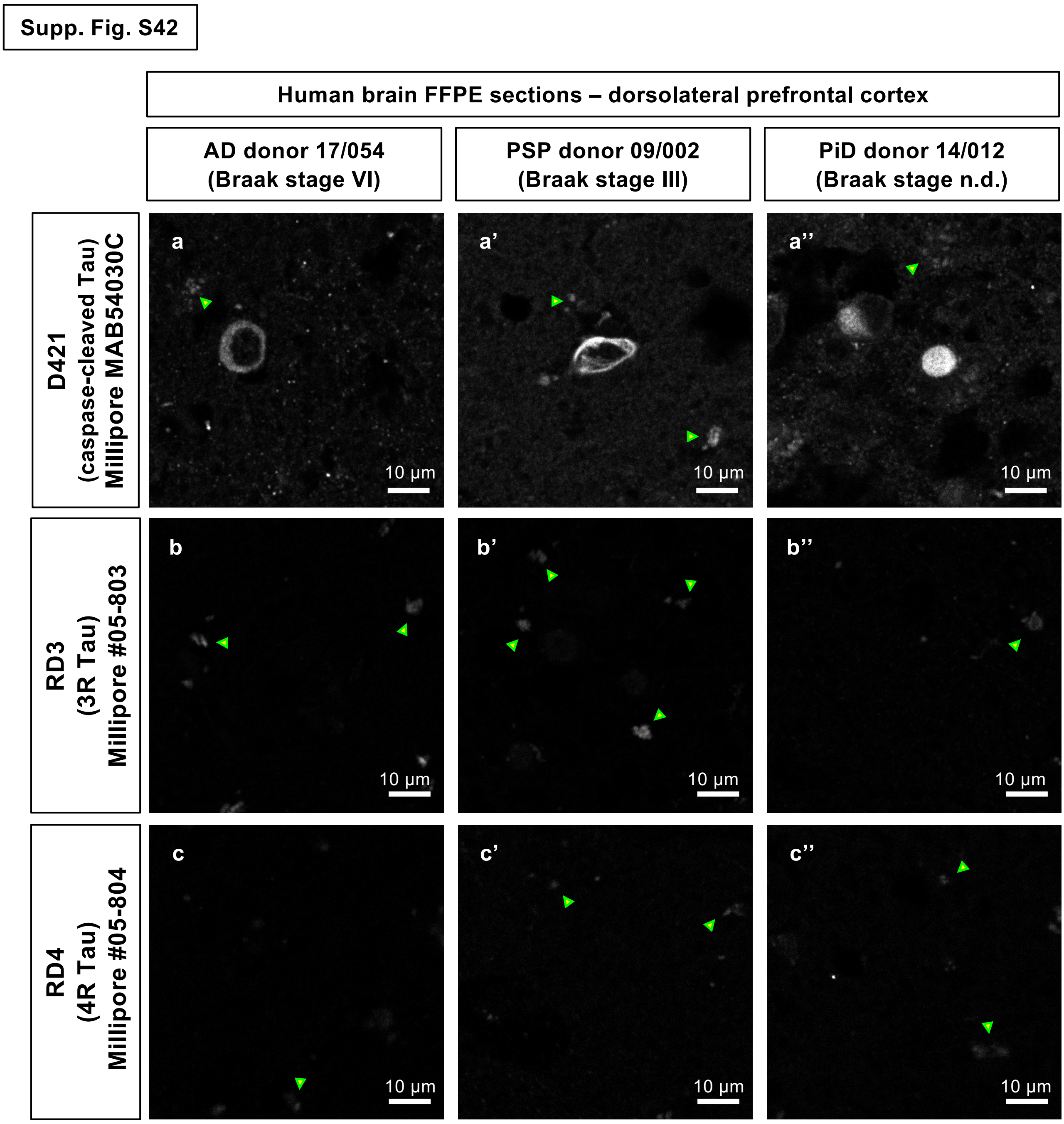
Supp. Fig. S42
Human brain FFPE sections – dorsolateral prefrontal cortex
AD donor 17/054
(Braak stage VI)
PSP donor 09/002
(Braak stage III)
PiD donor 14/012
(Braak stage n.d.)
D421
(caspase-cleaved Tau)
Millipore MAB54030C
RD3
(3R Tau)
Millipore #05-803
RD4
(4R Tau)
Millipore #05-804
a
a'
a''
b
b'
b''
c
c'
c''
10 µm

Supplementary Figure S42. IHC-IF immunolabelling of FFPE human brain sections from tauopathy donors with variant-specific Tau antibodies. a-c: D421 Tau-C3 Merck Millipore MAB54030-C (**a-a''**) RD3 (3R Tau) Merck Millipore 05-803 (**b-b''**); RD4 4R Tau Merck Millipore 05-804 (**c-c''**). Scale bars = 10 µm. Brain sections originated from the dorsolateral prefrontal cortex (Brodmann areas 9/46). Green arrowheads indicate highly autofluorescent lipofuscin particles (see **Supp. Fig. S2**)

Supp. Fig. S43

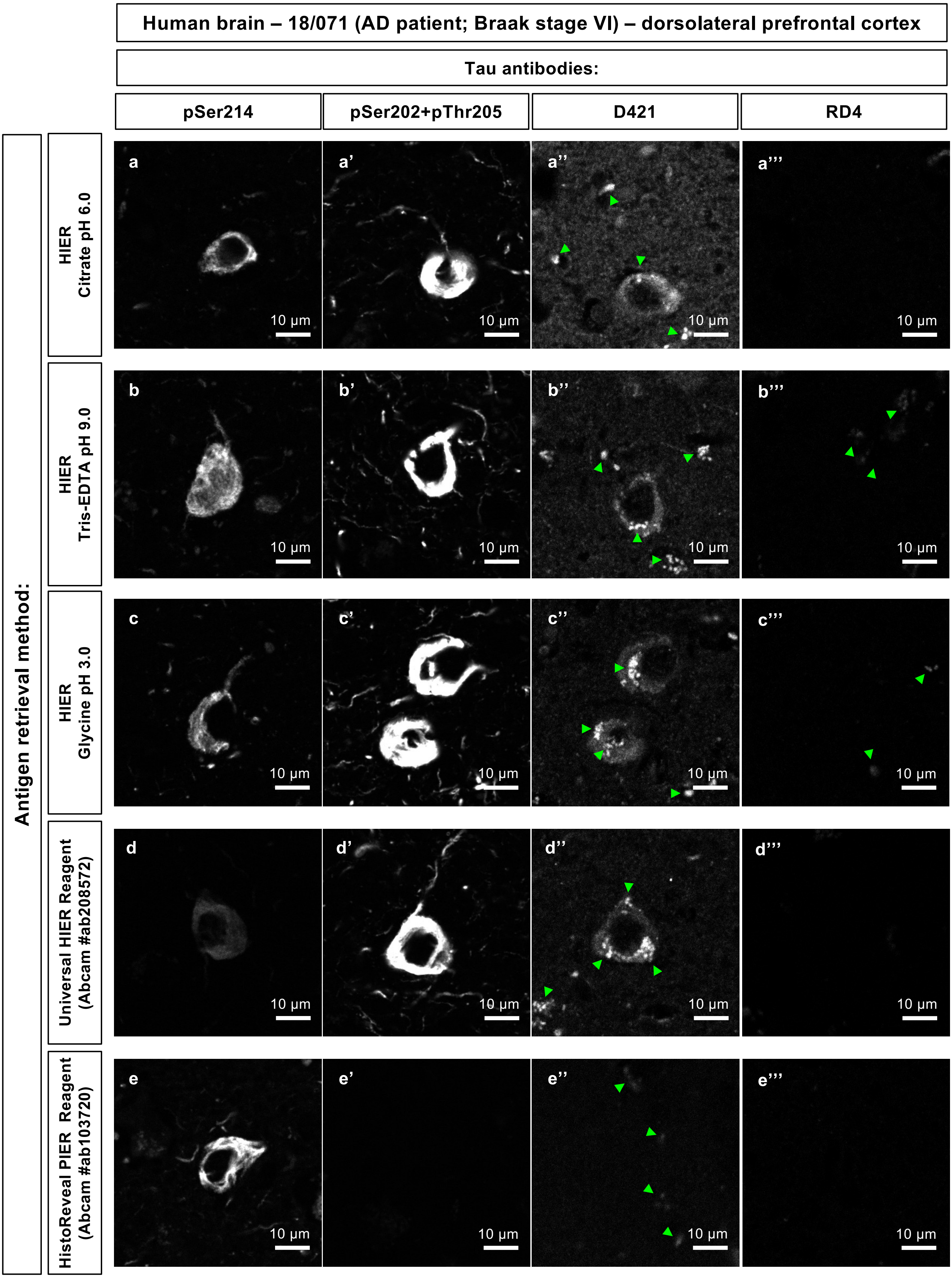

Human brain – 18/071 (AD patient; Braak stage VI) – dorsolateral prefrontal cortex
Tau antibodies:
pSer214
pSer202+pThr205
D421
RD4
Antigen retrieval method:
HIER Citrate pH 6.0
HIER Tris-EDTA pH 9.0
HIER Glycine pH 3.0
Universal HIER Reagent (Abcam #ab208572)
HistoReveal PIER Reagent (Abcam #ab103720)
a
a'
a''
a'''
b
b'
b''
b'''
c
c'
c''
c'''
d
d'
d''
d'''
e
e'
e''
e'''
10 µm

Supplementary Figure S43. Effect of different antigen retrieval protocols on IHC-IF labelling of FFPE human brain sections with different Tau antibodies. HIER = heat-induced epitope retrieval. PIER = proteolytic-induced epitope retrieval. IHC-IF data illustrating the effect of the following antigen retrieval protocols: HIER citrate pH 6.0 (**a-a'''**), HIER Tris-EDTA pH9.0 **(b-b'''**), HIER Glycine pH 3.0 (**c-c'''**), Universal HIER reagent Abcam #ab208572 (**d-d'''**), HistoReveal PIER reagent Abcam #ab103720 (**e-e'''**). Each antigen retrieval method was tested in combination with the following antibodies: pSer214 Abcam #ab170892 (**a, b, c, d, e**), pSer202+pThr205 Abcam ab210703 (**a', b', c', d' e'**), D421 Tau-C3 Merck Millipore MAB54030-C (**a'', b'', c'', d'', e''**), RD4 4R Tau Merck Millipore 05-804 (**a''', b''', c''', d''', e'''**). Brain sections originated from the dorsolateral prefrontal cortex (Brodmann areas 9/46). Green arrowheads indicate highly autofluorescent lipofuscin particles (see **Supp. Fig. S2**), which are particularly bright in the red region of the spectrum (555 nm channel) and are visible in the D421 images due to the low signal intensity obtained when tissue sections were labelled with this antibody. This required microscope acquisition settings (e.g. gain and laser intensity) to be increased, which also increased the signal emitted by lipofuscin particles. Lipofuscin particles were distinguishable from genuine Tau labelling due to their punctate/vesicular staining pattern and the fact that they emitted fluorescence in all channels.

Supp. Fig. S44

Human brain – 18/071 (AD patient; Braak stage VI) – dorsolateral prefrontal cortex

Antigen retrieval: Citrate pH 6.0 HIER + formic acid treatment

DAB chromogenic detection

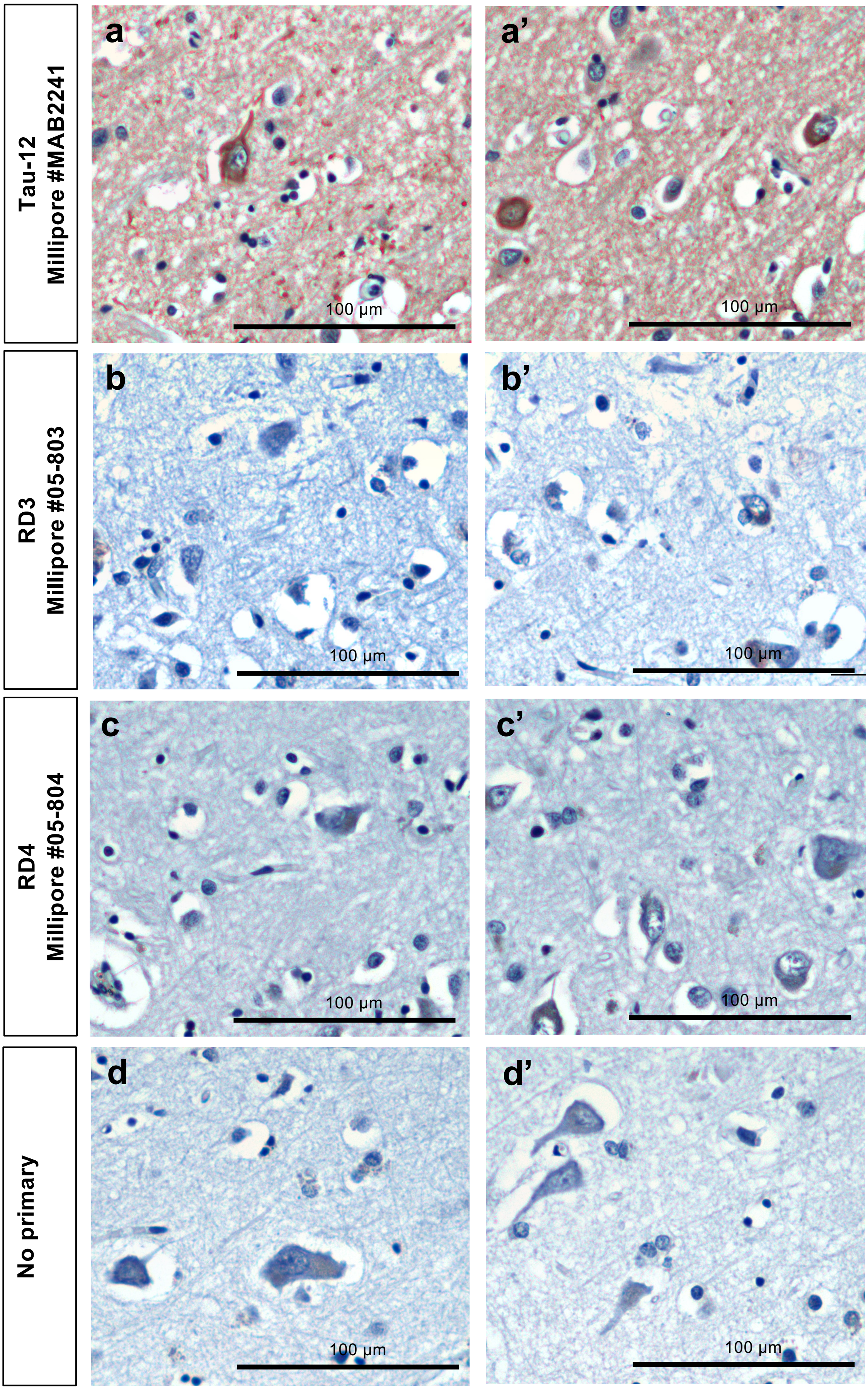

Supplementary Figure S44. IHC-DAB immunolabelling of FFPE human brain sections from tauopathy donors with variant-specific Tau antibodies.

Supp. Fig. S45 (part I) – Phospho-sites overlapping antibody epitopes

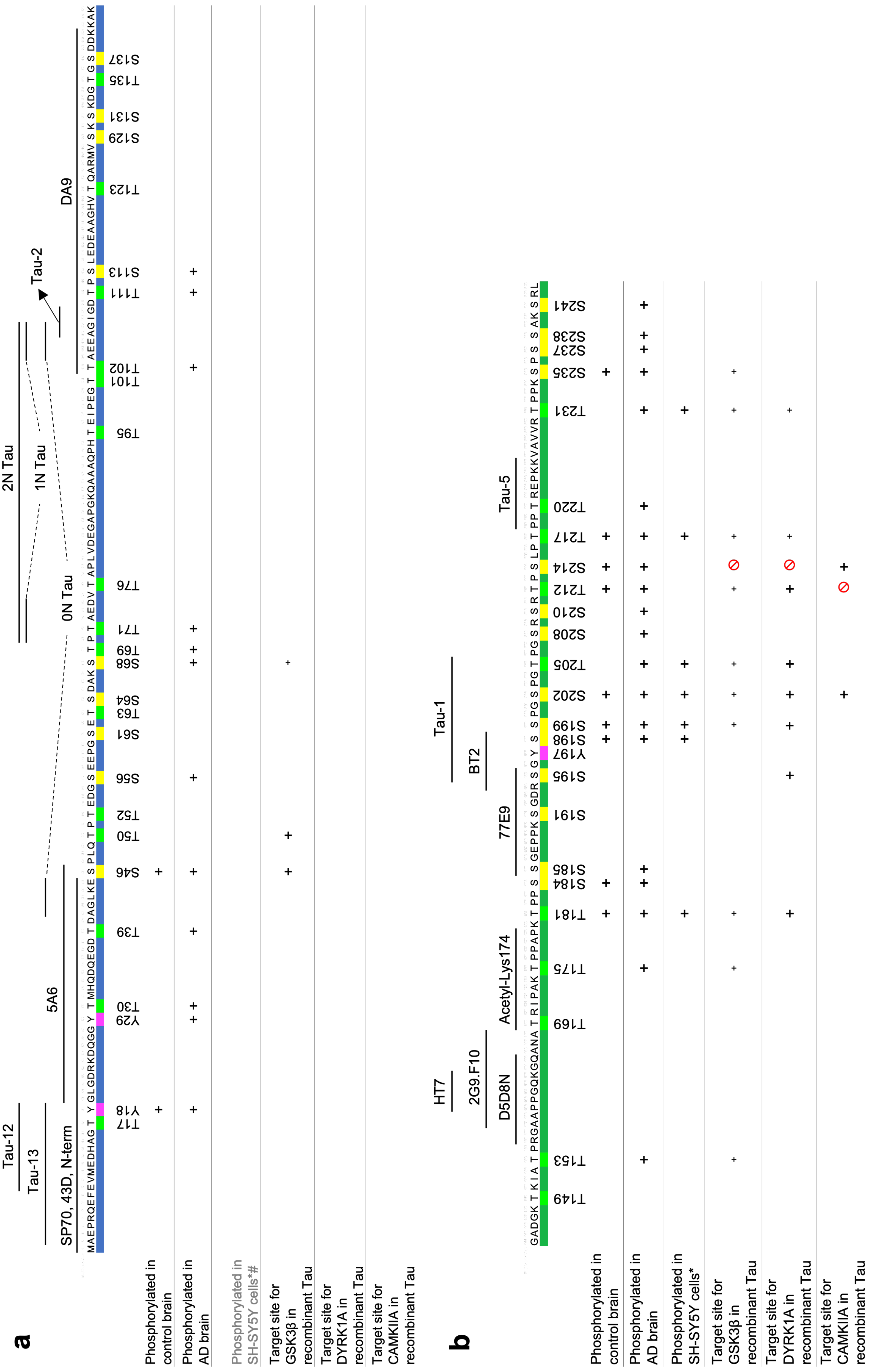

* as suggested by detection with phospho-Tau antibodies in the present study
\# none of the phospho-Tau antibodies included in this study target residues located in the N-terminal domain

Supp. Fig. S45 (part II) – Phospho-sites overlapping antibody epitopes

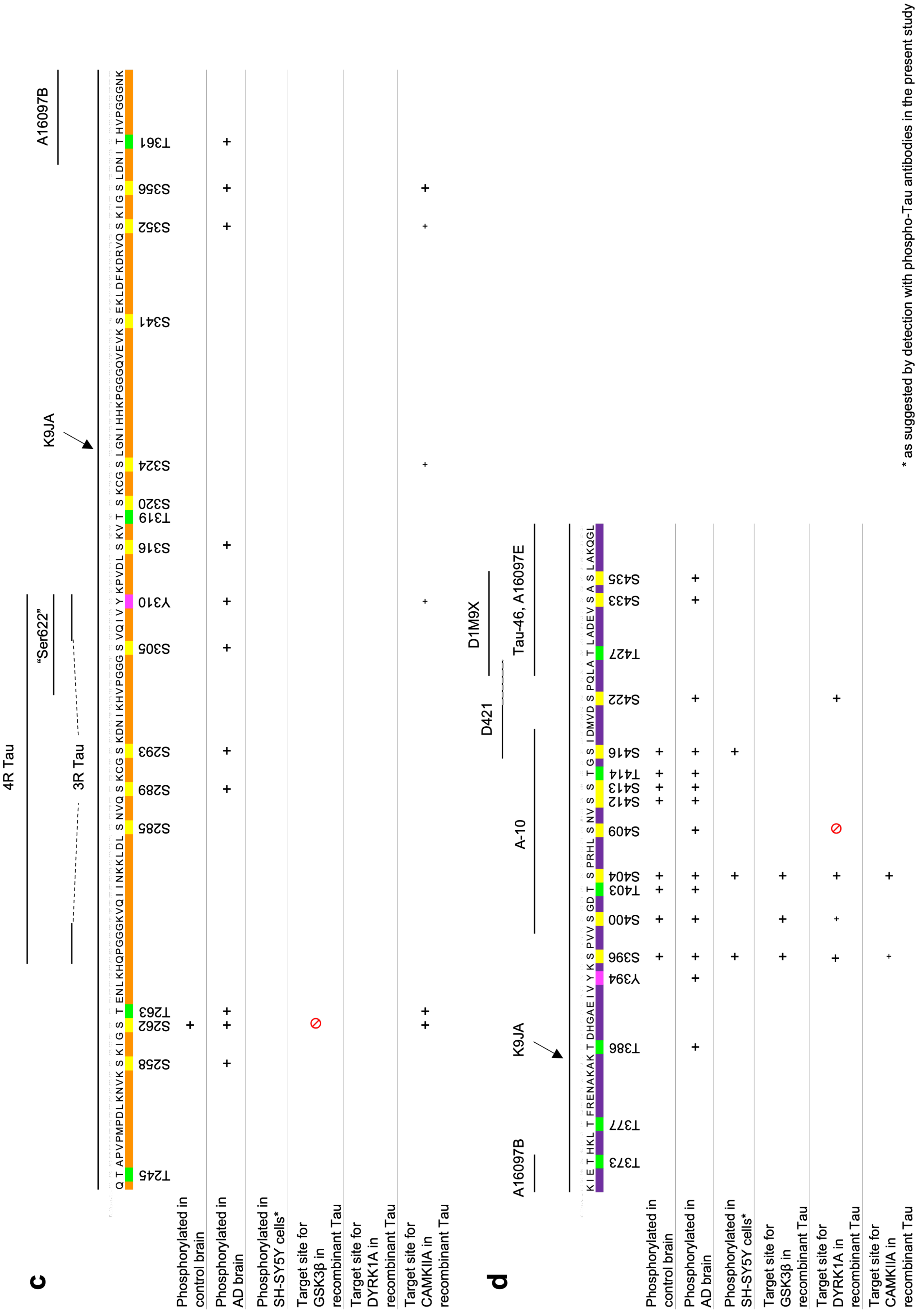

Supplementary Figure S45. Potential Tau phosphorylation sites overlapping antibody epitopes. Diagrams showing the potential phosphorylation sites against the amino acid sequences of the main protein domains of 2N4R human Tau: N-term domain (**a**), proline-rich domain (**b**), MTBR (**c**), and C-term domain (**d**). The regions containing the epitopes of the phosphorylation-independent antibodies tested in this study (plus Tau-1) are shown above the diagram. In the case of isoform-specific antibodies whose target isoforms are defined by the exclusion of specific exons (i.e. 0N, 1N and 3R Tau antibodies), their epitopes are presumed to span the resulting exon-exon junction. The exact epitope is, therefore, likely to lie within the sequences encoded within the five amino acids on either side of these junctions. In the case of 2N and 4R Tau antibodies, their epitopes may lie anywhere with exons 3 and 10, respectively, or at the exon-exon junctions of exons 3 or 10 with their neighbouring exons. The region covering the epitopes of 2N and 4R Tau antibodies is therefore marked as the entire sequence of exons 3 and 10, respectively, +/- five amino acids. In the case of antibodies whose epitope is reported as "surrounding residue x" (e.g. D5D8N, Ser622, D1M9X), their epitopes are presumed to lie within the region +/- five amino acids of the respective residue. Marked below the diagram are the residues known to be phosphorylated in control human brain, AD human brain and SH-SY5Y cells, as well as those predicted to be phosphorylated in each of the three recombinant phospho-Tau variants used in this study. Where a kinase has been explicitly reported to not phosphorylate Tau at a certain residue, this in indicated on the diagram as ⊘. Brain phosphorylation sites were compiled from [14, 162, 165]. Recombinant pTau phosphorylation sites were compiled from: [29, 62, 119, 150, 152] for GSK3β, [89, 122] for DYRK1A, and [29, 87, 150, 153, 168] for CAMKIIA.
